# Visible‐Light‐Promoted Metal‐Free Synthesis of (Hetero)Aromatic Nitriles from C(sp^3^)−H Bonds**

**DOI:** 10.1002/anie.202011815

**Published:** 2020-12-01

**Authors:** Kathiravan Murugesan, Karsten Donabauer, Burkhard König

**Affiliations:** ^1^ Faculty of Chemistry and Pharmacy University of Regensburg Germany

**Keywords:** ammoxidation, C−H functionalization, methylarenes, nitriles, photoredox catalysis

## Abstract

The metal‐free activation of C(sp^3^)−H bonds to value‐added products is of paramount importance in organic synthesis. We report the use of the commercially available organic dye 2,4,6‐triphenylpyrylium tetrafluoroborate (TPP) for the conversion of methylarenes to the corresponding aryl nitriles via a photocatalytic process. Applying this methodology, a variety of cyanobenzenes have been synthesized in good to excellent yield under metal‐ and cyanide‐free conditions. We demonstrate the scope of the method with over 50 examples including late‐stage functionalization of drug molecules (celecoxib) and complex structures such as l‐menthol, amino acids, and cholesterol derivatives. Furthermore, the presented synthetic protocol is applicable for gram‐scale reactions. In addition to methylarenes, selected examples for the cyanation of aldehydes, alcohols and oximes are demonstrated as well. Detailed mechanistic investigations have been carried out using time‐resolved luminescence quenching studies, control experiments, and NMR spectroscopy as well as kinetic studies, all supporting the proposed catalytic cycle.

## Graphical abstract

C(sp^3^)−H bonds have been converted to cyano derivatives under metal‐ and cyanide‐free condition in the presence of visible light and an organic dye. Potential applications of this method in the late‐stage transformation of complex drug molecules, steroid derivatives, and gram‐scale reactions have been described.

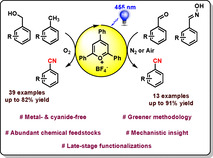

## Introduction

Transition‐metal based catalysts have an indispensable role in several chemical reactions^[1]^ industrial production,[^1a^, ^1b^, ^1c^, ^1d^, ^1e^, ^1h^] fine and bulk chemical synthesis.^[1t]^ During the last two decades, several transition‐metal based catalysts[^1q^, ^6^] have been developed for the activation of C−H bonds in *sp* ^3^ centers via thermal and photochemical pathways.[^1q^, ^2^] One of the most important transformations is the conversion of petroleum by‐products into fine chemicals,[^1a^, ^1b^] for example, the synthesis of benzonitrile from toluene.[^1b^, ^1c^] Industrially, benzonitrile is produced by ammoxidation of toluene using a transition‐metal catalyst (vanadium) and applying a high NH_3_ and O_2_ pressure at 300–500 °C.[^1b^, ^1c^] Since the nitrile moiety is an essential functional group in various drugs and bioactive compounds,^[3]^ as well as an important building block for the preparation of fine and bulk chemicals,^[4]^ novel, milder, (transition)metal catalyzed methods starting from alcohols,^[5]^ amines,^[6]^ aldehydes^[7]^ or more conveniently from simple CH_3_ have been developed in recent years.[^7f^, ^8^] On example comprises the palladium‐catalyzed cyanation of methylarenes using *t*BuONO as nitrogen source reported by Wang and co‐workers in 2013.^[8a]^

**Scheme 1:**
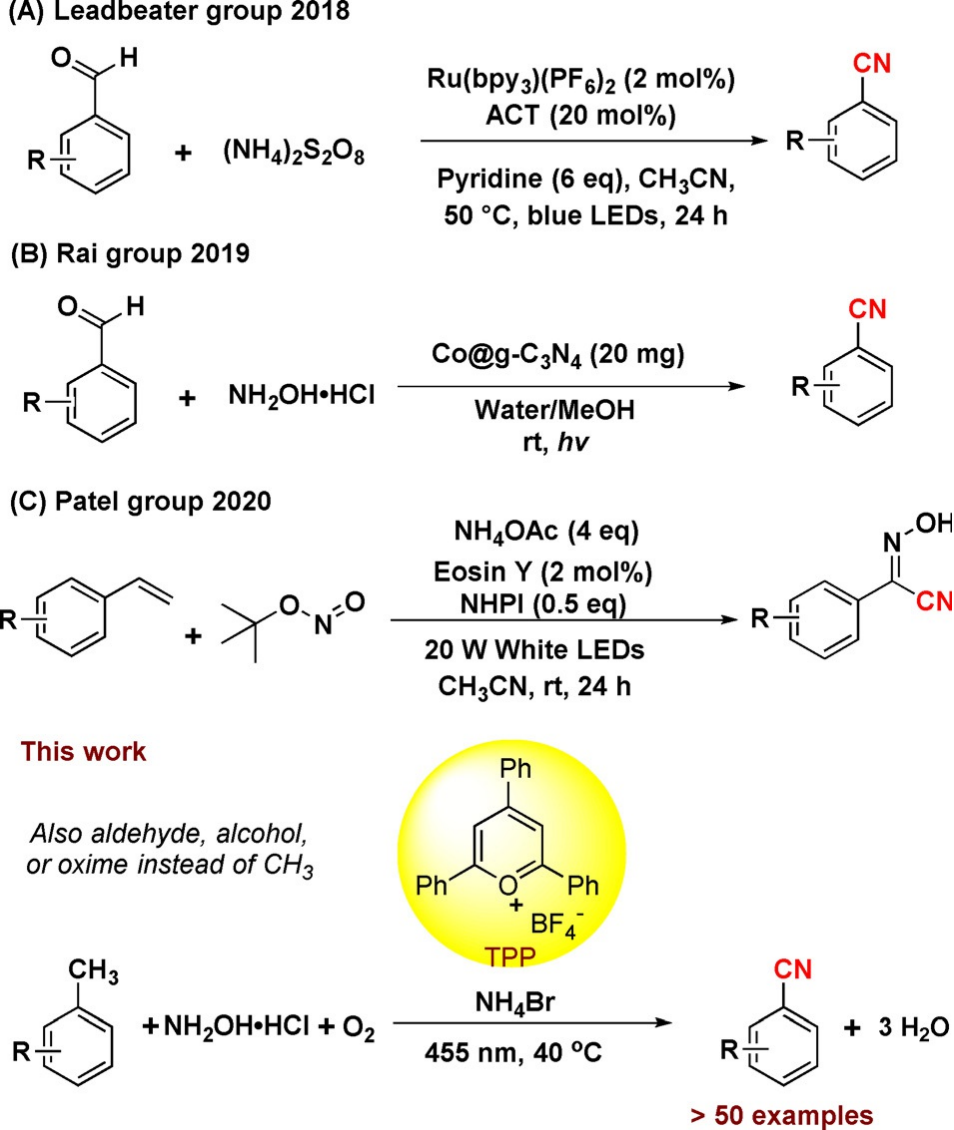
Photocatalytic nitrile formations using ammonium salts.

While these methods carry a remarkable impact and are highly valuable, they operate in most cases under elevated temperature and employ hazardous reagents or (transition)metals. However, owing to the high price,^[9]^ low abundance^[10]^ or toxicity,^[11]^ of (transition)metals, the interest in using alternative metal‐free catalysts is growing within the scientific community, especially for the late‐stage functionalization of inert C−H bonds.^[12]^ An intriguing development in this regard is the activation of aromatic compounds by an organic photocatalyst to form nitriles from C(*sp* ^*2*^)−H bonds reported by Nicewicz^[13]^ and others.^[14]^ Yet, this method employs toxic cyanide as stochiometric reagent, which can be a drawback, espcially in a bulk‐scale synthesis. Besides the use of a toxic cyanide source, a few photochemical methods for the synthesis of nitriles have been developed using a greener ammonium salt.[^7d^, ^7e^, ^14a^] These include the conversion of aldehydes to nitriles using a ruthenium photocatalyst with ammonium persulfate as nitrogen source (Scheme 1 A)^[7d]^ or a heterogeneous Co@g‐C_3_N_4_ photocatalyst together with NH_2_OH⋅HCl reported by the Rai group (Scheme 1 B).^[7e]^ Another interesting transformation in this regard is the functionalization of styrenes to N‐hydroxybenzimidoyl cyanides (Scheme 1 C).^[14a]^ Most photocatalytic methods furnishing nitriles in absence of cyanide use pre‐functionalized starting materials. However, the conversion of methylarenes to nitriles analogous to the industrial ammoxidation illustrates a simpler and more efficient retrosynthetic pathway.

Considering all factors, we wondered if a photocatalytic, metal‐ and cyanide free one‐step procedure from toluene derivatives to nitriles can be developed. As activation method, we envisioned that methylarene substrates (*E*_1/2_=+2.36 V vs. SCE for toluene)^[15]^ may be activated by a highly oxidizing photocatalysts or by a hydrogen atom abstaction at the benzylic position. Herein we describe the use of the commercially available organic dye 2,4,6‐triphenylpyrylium tetrafluoroborate (TPP)^[16]^ for the synthesis of nitriles from methylarenes. In addition to this, a detailed mechanistic investigation was carried out to support our mechanistic hypothesis. All experimental results and spectroscopic analyses support the proposed catalytic cycle. To the best of our knowledge, this is the first example for a photocatalytic ammoxidation of methylarenes using abundant feedstock materials, ammonium salts and molecular oxygen without the use of metals and toxic reagents.

## Results and Discussion

At the start of our investigation, we screened commercially available organic dyes for the desired transformation (Table 1 and S1). Gratifyingly, the use of DDQ (PC‐1) and acridinium‐based photocatalysts (PC‐2 and PC‐3) gave the desired product with NH_2_OH⋅HCl as ammonia source and NH_4_Br as additive, however only in a low yield of 12–26 % (Table 1, entries 1–3). Interestingly, the more sensitive photocatalyst TPP (PC‐4) (*E*_1/2_=+2.55 V vs. SCE),^[17]^ gave methyl 4‐cyanobenzoate (**2**) in a good GC‐yield of 76 % (Table 1, entry 4). Screening different ammonium salts, merely hydroxylamine hydrochloride gave the desired product **2**, while all other tested ammonium sources, such as ammonium acetate and aqueous ammonia failed to render the desired product (Table 1, entries 4–6 and Table S1, entries 5–6). Possible reasons for this observation are that the oxidation of simple ammonia (*E*_1/2_=+0.63 V vs. SCE)^[18]^ might be faster than the required oxidation of the substrate and the potential degradation of PC‐4 in the presence of a base or nucleophiles. Crucially, control experiments revealed the necessity of light, photocatalyst, O_2_ and NH_2_OH⋅HCl for the formation of the desired product (Table S4, entries 2–4 and 7).

**Table 1:** Photocatalyst and ammonia source screening. 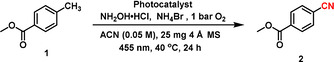

Entry	Photocatalyst (PC)	Ammonia source	Yield of 2 (%)
1^[a]^	PC‐1	NH_2_OH⋅HCl	26
2^[a]^	PC‐2	NH_2_OH⋅HCl	12
3^[a]^	PC‐3	NH_2_OH⋅HCl	14
4^[a]^	PC‐4	NH_2_OH⋅HCl	76
5^[a]^	PC‐4	NH_4_(OAc)	NR
6^[a]^	PC‐4	aq. NH_3_	NR

Reaction conditions: [a] 0.1 mmol substrate, 20 mol % PC, 3 equiv. ammonia source, 2.5 equiv. NH_4_Br, 25 mg 4 Å MS, 1 bar O_2_, 2 mL acetonitrile (0.05 M), 455 nm, 40 °C, 24 h, yields were determined by GC using n‐decane as standard. 
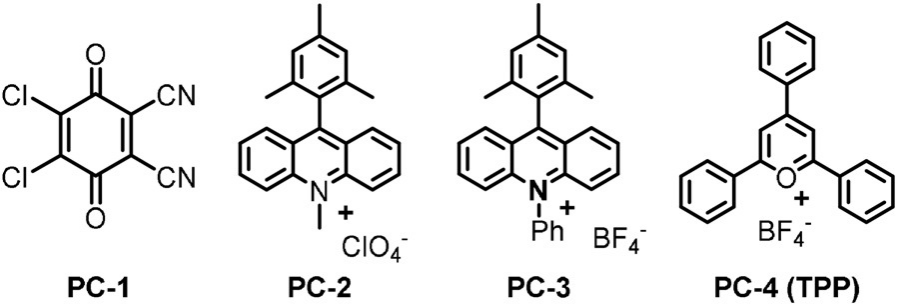

**Figure 1:**
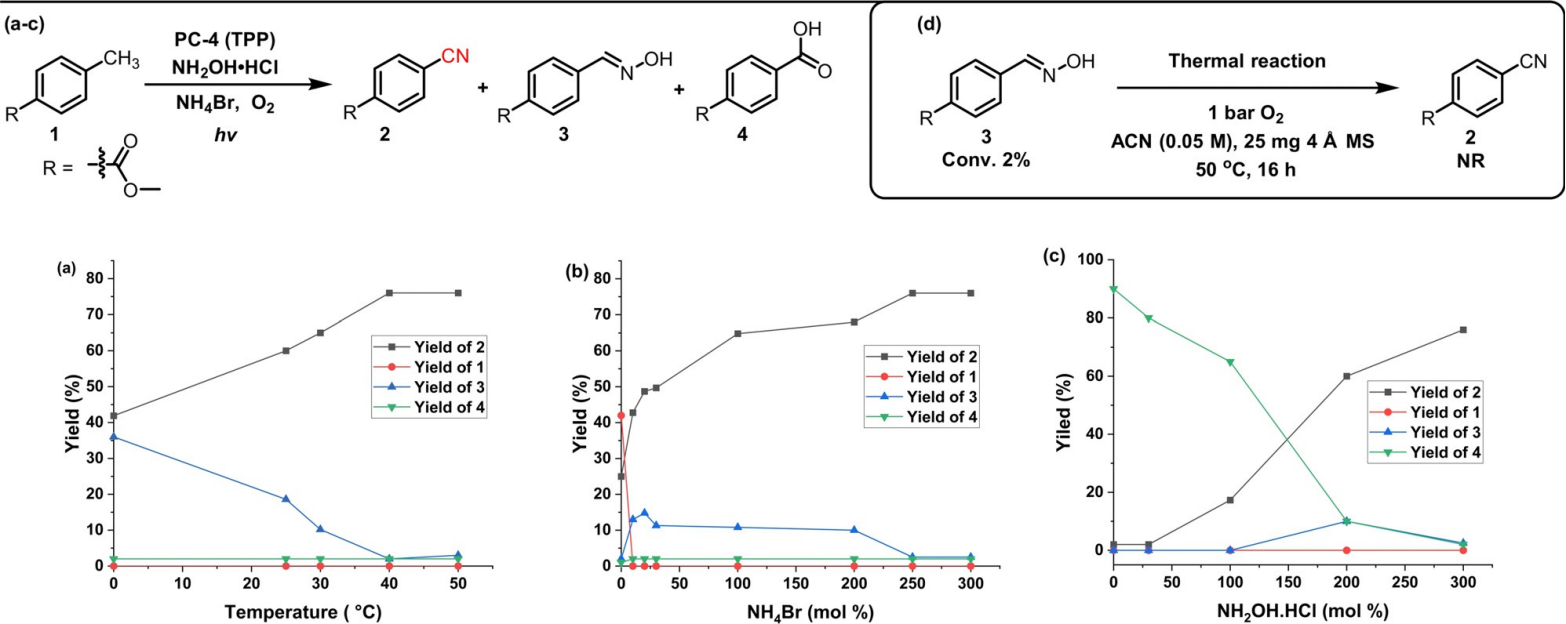
Influence of temperature and NH_4_Br and NH_2_OH⋅HCl loading. Reactions performed under standard reaction conditions (Table 1, entry 4) with (a) varying temperature, (b) varying NH_4_Br loading, (c) varying NH_2_OH⋅HCl loading and (d) starting from oxime **3** in absence of light, photocatalyst and NH_4_Br at 50 °C.

Having identified a potential photocatalyst (PC‐4, TPP), the effect of temperature (a), ammonium bromide (b) and hydroxylamine loading (c) was investigated in more detail, including the formation of by‐products (Figure 1). Most decisively, a lower reaction temperature seems to hamper the water elimination from the intermediate oxime **3** to form nitrile **2** (Figure 1 a), while the presence of NH_4_Br is crucial for a satisfactory conversion of **1** (Figure 1 b). As expected, the absence of a suitable ammonium source leads to the over‐oxidation to the corresponding acid **4** (Figure 1 c). Notably, the conversion of oxime **3** to nitrile **2** only proceeds under photocatalytic conditions and not in the dark under thermal conditions (Figure 1 d). The complete optimization process including time, solvent, additive, catalyst loading and wavelength variation is given in the supporting information (Table S1–S3 and Figure S3).

### Mechanistic Hypothesis

**Figure 2:**
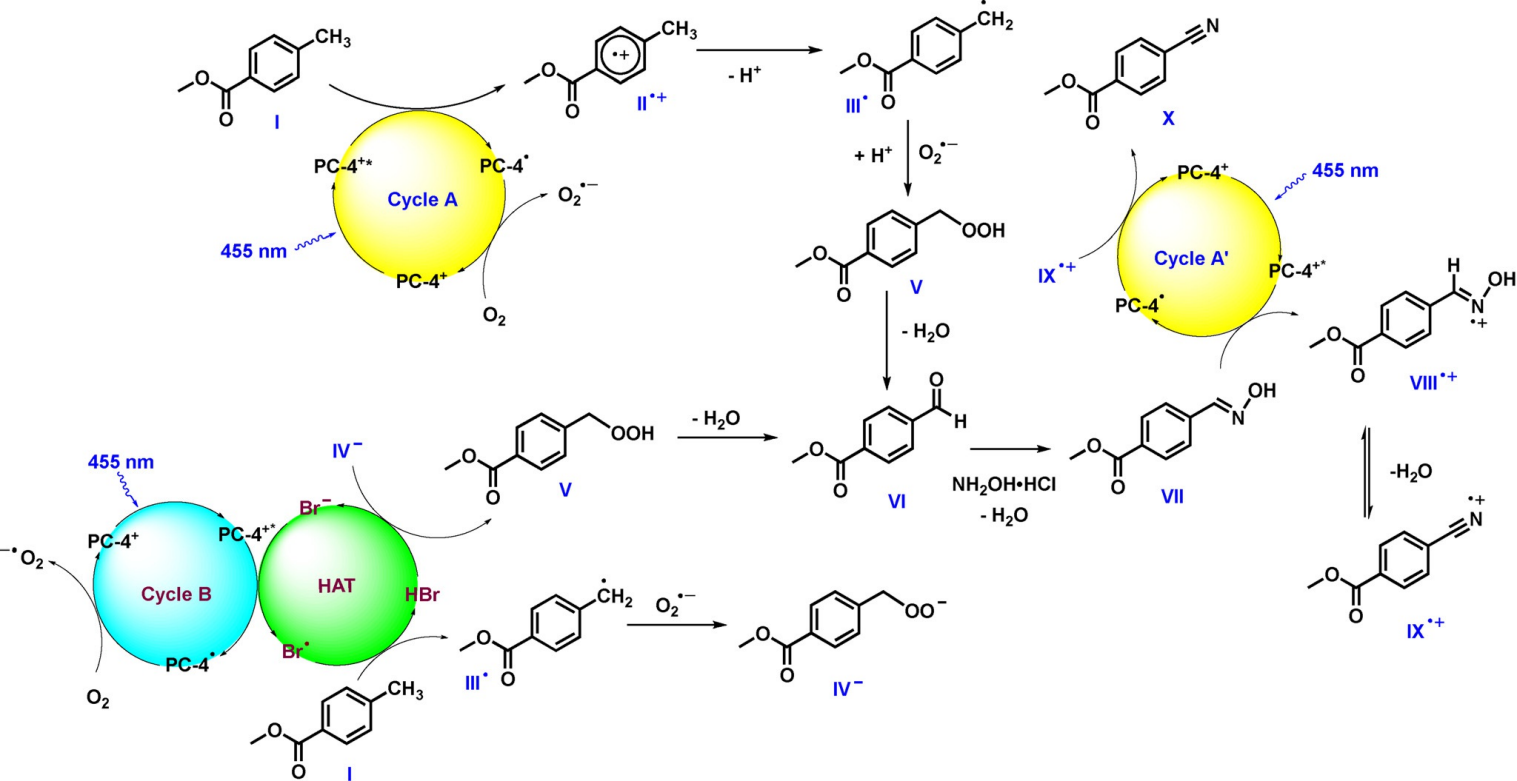
Proposed catalytic cycles for the ammoxidation of methylarenes.

The initial results suggest that the oxime is one of the key intermediates, as its depletion is accompanied by the formation of the product (Figure 1). Further, the control experiment yielding no product in the absence of O_2_ indicates that the oxime might be formed from the corresponding aldehyde. Based on this we envisioned the following catalytic cycle (Figure 2; Cycle A): The substrate (*E*_1/2_ (**1**/**1^.^** ^+^)=+2.45 V vs. SCE, see SI) is oxidised by the excited photocatalyst (*E*_1/2_ (PC‐4^+^*/ PC‐4^.^)=+2.55 V vs. SCE)^[17]^ generating radical cation **II^.^** ^+^ and the reduced photocatalyst species (PC‐4^.^).

The photocatalytic cycle is closed by single electron transfer from PC‐4^.^ to O_2_, forming a superoxide anion (O_2_^.−^).^[19]^ Intermediate **II^.^** ^+^ loses a proton to form the more stable benzylic radical (**III^.^**), which combines with the superoxide anion to peroxide derivative **V** after protonation. Alternatively, benzyl radical **III^.^** can directly add to O_2_ to give a peroxide radical, which may accept an electron from PC‐4^.^ to close the photocatalytic cycle and form intermediate **V**.^[20]^ Elimination of water from **V** leads to the corresponding aldehyde intermediate (**VI**), which yields oxime **VII** upon condensation with NH_2_OH⋅HCl. As the oxime is not converted to the final product in the absence of light (Figure 1 d), a following photocatalytic cycle is proposed (Figure 2, Cycle A′). Here, the oxime (**VII**) is oxidised to **VIII^⋅^** ^+^ via reductive quenching^[7e]^ of PC‐4^+^* followed by elimination of water to give intermediate **IX^.^** ^+^. The reduced PC undergoes SET with **IX^.^** ^+^ to render the desired product **X**, closing the photocatalytic cycle and completing a redox‐neutral process. As shown in Figure 1, the presence of NH_4_Br is crucial to obtain a satisfactory yield. Thus, another catalytic cycle may be operative, yielding the same reaction product (Figure 2, Cycle B). Bromide anions are known hydrogen atom transfer (HAT) catalysts.^[21]^ In presence of visible‐light, the excited PC can oxidise Br^**−**^ to generate the Br^.^ radical, which is capable to abstract the H‐atom from the methylarene to yield a benzyl radical (**III^.^**) and HBr. The combination of superoxide anion and **III^.^** yields intermediate **IV^−^**, which deprotonates HBr to regenerate Br^**−**^ and form intermediate **V**. After its formation, **V** follows the same mechanistic pathway as described above (Cycle A and A′).

To support this proposed hypothesis, we performed several mechanistic experiments, starting with spectroscopic investigations. Time‐resolved luminescence quenching experiments indicated that methyl 4‐methylbenzoate (**1**) can be oxidized by the excited TPP (PC‐4) (Figure S4a,b), as a slight decrease of the luminescence lifetime with increasing concentration of **1** was observed (*K_SV_*=0.283 M^−1^). Further, no life‐time quenching was detected with product **2** or proposed aldehyde intermediate **VI** (Figure S4c). On the other hand, the oxime intermediate (**3**) showed a superior quenching to methyl 4‐methylbenzoate (**1**) (Figure S4c, *K_SV_*=25.9 M^−1^), indicating an interaction of the excited photocatalyst with the oxime intermediate to form the desired product under the applied reaction conditions (Cycle A′). To support that an electron transfer is feasible, cyclic voltammetry measurements revealed a potential of (*E*_1/2_(**3^.^** ^+^/**3**)=+1.80 V vs. SCE, see SI) for the oxime, which lies within the oxidation window of PC‐4.

The quenching efficiencies of NH_2_OH⋅HCl and NH_4_Br were investigated as well, with the former (*K_SV_*=23.5 M^−1^) exhibiting a poorer quenching than the later one (*K_SV_*=75 M^−1^) (Figure S6), which suggests that Cycle B can be active as well. Comparing the luminescence lifetime quenching of NH_4_Br and the methyl 4‐methylbenzoate starting material (**1**), NH_4_Br seems to decrease the lifetime much more effectively (Figure S7). However, an electron‐rich substrate (4‐methyl anisole) opposed to electron‐poor substrate **1** proved to be a potent quencher as well (Figure S8, *K_SV_*=83.7 M^−1^), showing a slightly superior quenching ability to NH_4_Br. Thus, based on the time‐resolved luminescence quenching experiments, both proposed catalytic cycles A and B can be operative, with their respective importance likely being dependent on the electronic nature of the starting material.

Next, several mechanistic control experiments were performed to directly or indirectly detect reaction intermediates vital for the mechanistic process (Scheme S2). The model reaction was performed under standard reaction condition in presence of TEMPO, yielding no product, which indicates a radical pathway (Scheme S2, Experiment‐1). Further, the radical cation intermediate (II^.+^) could be trapped in the presence of pyrazole or 4‐cholorpyrazole as nucleophiles under standard reaction condition, in a similar reaction as reported by Nicewicz,^[22]^ supporting its formation (Scheme S2, Experiment‐2). Looking at the more stable intermediates, the methylarene is proposed to be oxidized to the corresponding aldehyde (**VI**). The generation of an aldehyde intermediate is supported by its detection when avoiding an ammonium source (Scheme S2, Experiment‐3).

Further, aldehydes as well as oximes could be used as starting materials for the synthesis of the final nitrile product (Scheme S2, Experiment‐5 & 6). In both cases, product **2** was formed with a yield of 91 % and 80 %, respectively. Notably, the presence of O_2_ is not required in both cases, which is in accordance to the proposed reaction mechanism (Figure 2, Cycle A′). The reaction progress could be followed by NMR as well (Figure S15–18), clearly showing the formation of the oxime followed by the product formation under the applied conditions. As mentioned above, the oxime could not be converted to the nitrile under thermal conditions. To indicate the necessity of more than one photon for the formation of one product molecule, the product yield dependent on the irradiation intensity was investigated (see SI, Figure S19).^[23]^ Collectively, the described control experiments and spectroscopic investigations all support the proposed photocatalytic cycles. In addition, the catalyst deactivation pathway was also studied under the standard reaction conditions, suggesting 2,4,6‐triphenylpyridine as the degradation product, which could further be observed in the scale‐up batch as minor by‐product supported by HRMS (Scheme‐S3).

**Scheme 2:**
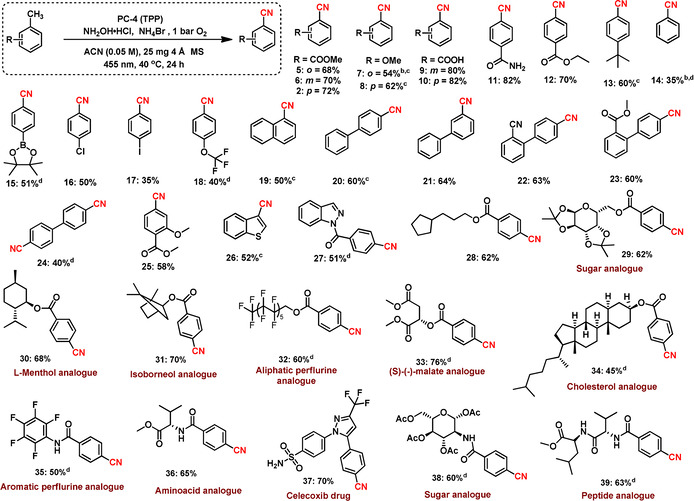
Substrate scope for the synthesis of functionalized cycanobenzenes. Reactions conditions: ^a^ 0.1 mmol substrate, 20 mol % PC, 3 equiv. NH_2_OH⋅HCl, 2.5 equiv. NH_4_Br, 25 mg 4 Å MS, 1 bar O_2_, 2 mL acetonitrile (0.05 M), 455 nm, 40 °C, 24 h, isolated yields. ^b^ GC yields using n‐decane as standard. ^c^ Same as “a” using 1 equiv. of PTSCl. ^d^ Same as “a” 48 h instead of 24 h.

**Scheme 3:**
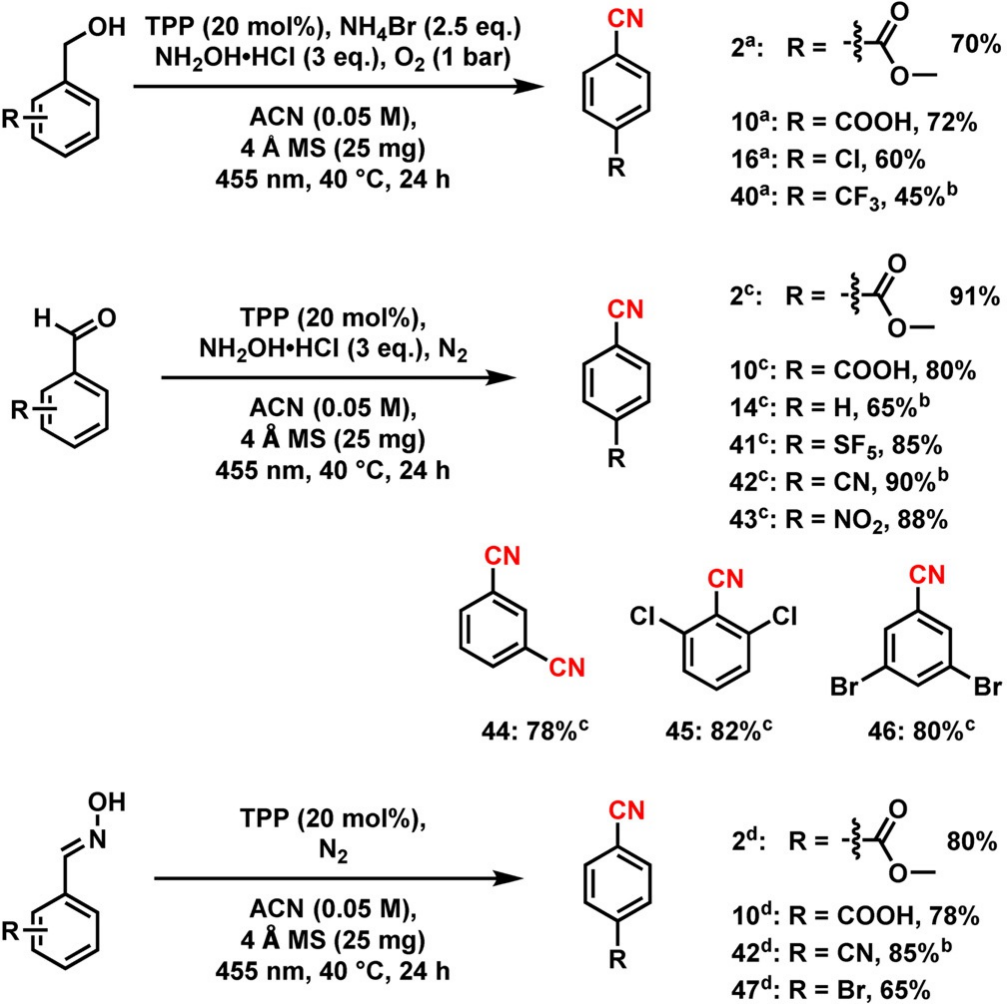
Synthesis of nitriles from alcohols, aldehydes, and oximes. Reaction conditions: ^a^ 0.1 mmol substrate, 20 mol % PC, 3 equiv. NH_2_OH⋅HCl, 2.5 equiv. NH_4_Br, 25 mg 4 Å MS, 1 bar O_2_, 2 mL acetonitrile (0.05 M), 455 nm, 40 °C, 24 h, isolated yields of nitriles. ^b^ GC yields of nitriles using n‐decane as standard. ^c^ Under N_2_ atmosphere and in absence of NH_4_Br. ^d^ Under N_2_ atmosphere and in absence of NH_4_Br and NH_2_OH⋅HCl.

With the successful conditions in hand, we explored the C(*sp* ^*3*^)‐H functionalization of different methylarenes (Scheme 2). Substrates bearing electron‐donating and ‐withdrawing groups, as well as heterocycles, gave the respective products in good to excellent yields (Scheme 2, **5**–**39**). Functional groups such as carboxylic acid, ester, amide, halogens, cyanide and boronic ester were untouched during the reaction (Scheme 2, **5**–**6**, **9**–**12**, **15**–**18** and **22**–**23**). Applying this methodology, a dinitrile could also be synthesized in a one‐pot procedure with a moderate yield (**24**). Structurally complex, bioactive‐ and drug‐ molecules could be employed for a selective cyanation as well, rendering the desired product in good to excellent yields up to 76 % (Scheme 2, **29**–**39**). Delightfully, substrates bearing multiple oxygen atoms, which are usually not stable under photochemical conditions in the presence of oxygen, are viable, too.^[24]^ Using the developed protocol, sugar derivatives **29** and **38** were obtained in 62 % and 60 % yield, respectively. Further, more challenging substrates such as cholesterol, isoborneol, amino acid and peptide derivatives gave the corresponding products in 45–70 % yield. Interestingly, anti‐inflammatory drug (Celecoxib, **37**) gave the desired product in 70 % yield. For the example of a bulk‐scale preparation, compound **2** and Celecoxib were cyanated in a 1 g scale to give the desired product in 65 and 60 % yield, respectively (Scheme S1). After the screening of methylarenes, we were interested to apply this methodology using alcohols, aldehydes and oximes as starting materials for the synthesis of aromatic nitriles as well (Scheme 3). Similar to methylarenes, alcohols, aldehydes and oximes gave good to excellent yields up to 91 %. Notably, isophthalaldehyde (Scheme 3) was cyanided twice to isophthalonitrile (**44**) in 78 % yield. Sterically crowded 2,6‐dichlorobenzaldehyde (Scheme 3) was a viable substrate, too, giving 2,6‐dichlorobenzonitrile (DCBN, **45**) in 82 % yield, which is used as herbicide and regarded as a potential intermediate for pesticides and agrochemicals.

## Conclusion

In conclusion, we present the first metal‐ and cyanide‐free visible‐light‐induced photocatalytic ammoxidation of C(*sp* ^3^)−H bonds using an abundant ammonia source and molecular oxygen. A detailed mechanistic investigation was carried out to support the proposed mechanistic hypothesis including various spectroscopy experiments. Applying this methodology, more than 50 aromatic and heteroaromatic substrates, as well as steroids and existing drug molecules containing methyl groups could be converted to the nitrile in good to excellent yields. In addition to this, the method could be executed on gram scale and alcohols, aldehydes and oximes could be used as starting materials for their conversion to nitriles in up to 91 % yield in the same manner.

## Conflict of interest

The authors declare no conflict of interest.

## Supporting information

As a service to our authors and readers, this journal provides supporting information supplied by the authors. Such materials are peer reviewed and may be re‐organized for online delivery, but are not copy‐edited or typeset. Technical support issues arising from supporting information (other than missing files) should be addressed to the authors.

Supplementary
