# Supplementary material for: Visible‐Light‐Promoted Metal‐Free Synthesis of (Hetero)Aromatic Nitriles from C(sp3)−H Bonds
Source: Angew Chem Int Ed Engl. 2020 Dec 1;60(5):2439–45. doi: 10.1002/anie.202011815 (PMC7898869; doi:10.1002/anie.202011815)
Supplement: Supplementary file 1 — Supplementary [file ANIE-60-2439-s001.pdf]

Angewandte Chemie
Eine Zeitschrift der Gesellschaft Deutscher Chemiker

Supporting Information

Visible-Light-Promoted Metal-Free Synthesis of (Hetero)Aromatic Nitriles from $C(sp^3)$–H Bonds**

*Kathiravan Murugesan, Karsten Donabauer, and Burkhard König**

anie_202011815_sm_miscellaneous_information.pdf

Table of contents

1. General information

2. Synthetic procedure

2.1 General procedure for synthesis of starting materials

2.2 General procedure for synthesis of oximes

2.3 General procedure for synthesis of nitriles from methylarenes or alcohols

2.4 General procedure for synthesis of nitriles from aldehydes

2.5 General procedure for synthesis of nitriles from oximes

2.6 General procedure for gram scale reactions

3. Reaction optimization

4. Mechanistic Investigation

4.1 Kinetic studies

4.2 Time-resolved Luminescence Quenching studies

4.3 Mechanistic experiments

4.4 Trapping experiments

4.5 NMR studies

4.6 The product yield on irradiation density

4.7 Catalyst deactivation

4.8 Cyclic Voltammetry measurement

5. Characterization of prepared compounds

6. NMR spectra

7. References

1. General information

All required fine chemicals were purchased from commercial suppliers (abcr, Acros, Alfa Aesar, Fluka, Fluorochem, Merck, Sigma Aldrich, TCI) and were used directly without purification unless stated otherwise. All air and moisture sensitive reactions were carried out under nitrogen atmosphere using standard Schlenk manifold technique. Extra dry anhydrous acetonitrile was purchased from Acros organics.

All NMR spectra were measured at room temperature using a Bruker Avance 300 (300 MHz for ^{1}H, 75 MHz for ^{13}C, 282 MHz for ^{19}F) or a Bruker Avance 400 (400 MHz for ^{1}H, 101 MHz for ^{13}C, 376 MHz for ^{19}F)[1] NMR spectrometer. All chemical shifts are reported in δ-scale as parts per million [ppm] (multiplicity, coupling constant *J*, number of protons) relative to the solvent residual peaks as the internal standard.[2] Coupling constants *J* are given in Hertz [Hz]. Abbreviations used for signal multiplicity: ^{1}H-NMR: b = broad, s = singlet, d = doublet, t = triplet, q = quartet, p = quintet, and m = multiplet.

GC measurements were performed on a GC 7890 from Agilent Technologies. Data acquisition and evaluation was done with Agilent ChemStation Rev.C.01.04. GC/MS measurements were performed on a 7890A GC system from Agilent Technologies with an Agilent 5975 MSD Detector. Data acquisition and evaluation was done with MSD ChemStation E.02.02.1431. A capillary column HP-5MS/30 m x 0.25 mm/0.25 μM film and helium as carrier gas (flow rate of 1 mL/min) were used. The injector temperature (split injection: 40:1 split) was 280 °C, detection temperature 300 °C (FID). GC measurements were made and investigated *via* integration of the signal obtained. The GC oven temperature program was adjusted as follows: initial temperature 40 °C was kept for 3 minutes, the temperature was increased at a rate of 15 °C/min over a period of 16 minutes until 280 °C was reached and kept for 5 minutes, the temperature was again increased at a rate of 25 °C/min over a period of 48 seconds until the final temperature (300 °C) was reached and kept for 5 minutes. *n*-Decane was used as an internal standard.

Analytical TLC was performed on silica gel coated aluminium sheets (Merck, TLC Silica gel 60 F_{254}). Compounds were visualized by exposure to UV-light (254 or 366 nm) or by dipping the plates in staining solutions (permanganate stain, bromocresol green stain, ceric ammonium molybdate stain) followed by heating. Purification by column chromatography was performed with silica gel 60 M (40-63 μm, 230-440 mesh, Merck) or with a pre-packed Biotage® Snap Ultra

HP-Sphere™ 25 *u*m column on a Biotage® Isolera™ Spektra One device. All mixed solvent eluents are reported as v/v solutions.

High resolution mass spectrometry (HRMS) were performed at the Central Analytical Laboratory of the University of Regensburg. Mass spectra were recorded on a Finnigan MAT 95, ThermoQuest Finnigan TSQ 7000, Finnigan MAT SSQ 710 A or Agilent Q-TOF 6540 UHD instrument and a Waters Acquity UPLC system equipped with Waters PDA, sample manager, sample organiser, column oven and Waters Xevo QTOF mass spectrometer. Photoreactions in regular scale were irradiated with blue LEDs (OSRAM Oslon SSL 80 royal-blue, λ = 455 nm (± 15), average radiant flux 232 ± 23 mW, 2.9 V, 350 mA) or green LEDs (λ = 535 nm, average radiant flux, 29 ± 5 mW) and were exposed to light from the flat bottom side of the vial. The temperature of the reaction mixtures was controlled by a water-cooling circuit consisting of an aluminium cooling block connected to a thermostat (Fig. S1). An exemplary reaction in larger scale was carried out in a custom-built glass reactor which upon vigorous stirring generates a thin film of the reaction mixture between the reaction vessel and an attached cold finger. The reaction vessel was surrounded by blue LED arrays (OSRAM Oslon SSL 80 LT-2010, λ = 451 nm, 700 mA) generating a total radiant flux of 12 W (Fig. S2). Luminescence measurements were performed on a Horiba® Scientific FluoroMax-4 instrument using the above-mentioned quartz cells. Luminescence lifetime measurements were performed on a Horiba® Scientific DeltraPro™ fluorescence lifetime system using a 452 nm laser diode from Horiba® Scientific DeltaDiode™ as excitation source and above-mentioned quartz cells. The instrument response function (IRF) was determined prior to measurements by using colloidal silica (LUDOX®) in water.

CV measurements were performed with the three-electrode potentiostat galvanostat PGSTAT302N from Metrohm Autolab using a glassy carbon working electrode, a platinum wire counter electrode, a silver wire as a reference electrode and TBATFB 0.1 M as supporting electrolyte. The control of the measurement instrument, the acquisition and processing of the cyclic voltammetric data were performed with the software Metrohm Autolab NOVA 1.10.4. The measurements were carried out as follows: a 0.1 M solution of TBATFB in acetonitrile was added to the measuring cell and the solution was degassed by argon purge for 5 min. After recording the baseline the electroactive compound was added (0.01 M) and the solution was again degassed a stream of argon for 5 min. The cyclic voltammogram was recorded with one to three scans.

Afterwards ferrocene (2.20 mg, 12.0 μmol) was added to the solution which was again degassed by argon purge for 5 min and the final measurement was performed with three scans.

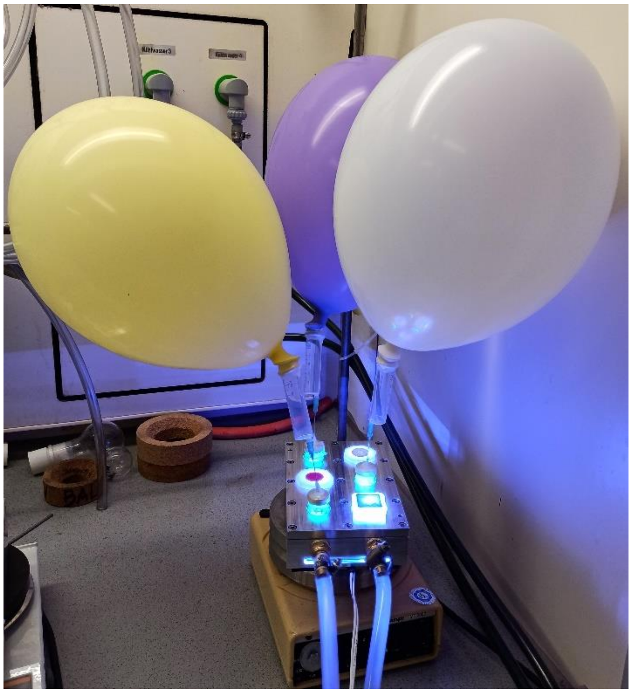

Fig. S1: Typical set-up for small scale synthesis

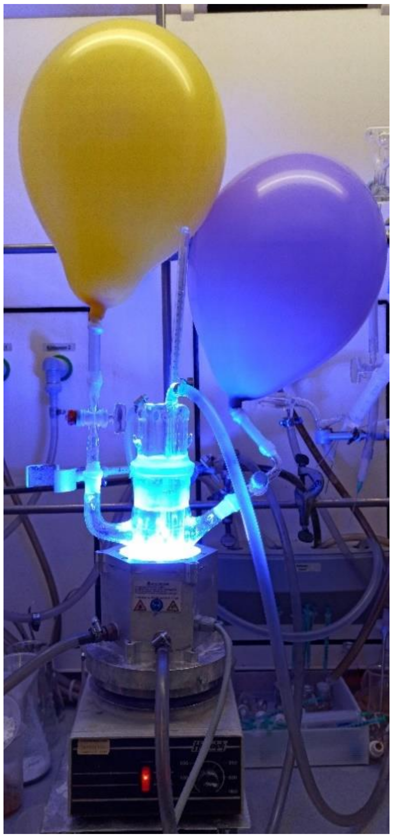

Fig. S2: Custom-built glass reactor for upscaling of the photocatalytic ammoxidation reaction.

2. Synthetic procedure

2.1 General procedure for the synthesis of methylarenes

Methylarenes were synthesized according to a literature procedure.[3]

General conditions: To a stirred solution of 4-methyl benzoyl chloride (7.5 mmol, 1.5 eq) in DCM (10 mL) and added an alcohol or amine nucleophile (5 mmol 1.0 eq), DMAP (0.5 mmol, 0.1 eq) and Et_3N (10 mmol, 2.0 eq) in DCM (10 mL). The reaction was allowed to stir overnight at room temperature. Then, the mixture was quenched upon addition of NH_4Cl (aq. 10%), and extracted with DCM (3x). The organic phase was washed with brine, concentrated and purified by silica gel flash chromatography to give the corresponding substituted methylarenes. If the HCl salt of the amine nucleophile was employed, 4 eq of Et_3N were used.

2.2 General procedure for the synthesis of oximes

Oximes were synthesized according to a literature procedure.[4]

A 100 mL round bottom flask equipped with a magnetic stirring bar was loaded with NH_2OH•HCl (1.2 eq for 1.0 g of substrate) in water (25 mL), followed by the addition of $NaHCO_3$ (1.75 eq for 1.0 g of substrate) at 0° C. A solution of the corresponding aldehyde (1.0 g, 1 eq) in MeOH was added to the above mixture and stirred at RT for 6h. The reaction progress was monitored by TLC (PE:EA). After completion of the reaction, MeOH was removed under reduced pressure. The precipitated solid was filtered off, washed with water and dried under high vacuum. The thus obtained product was used for following steps without further purification.

2.3 General procedure for the synthesis of nitriles from methylarenes or alcohols

A 5 mL crimp cap vial equipped with a magnetic stirring bar was loaded with TPP (20 μmol, 8.0 mg, 20 mol%), NH_2OH•HCl (0.3 mmol, 20.7 mg, 3 eq), NH_4Br (0.25 mmol, 25.0 mg, 2.5 eq), 4 Å molecular sieves (25 mg), the corresponding methylarene or alcohol (0.1 mmol, 1 eq.) and dry ACN (2 mL, 0.05M). In doing so, all solid compounds were added before capping the vial, whereas all liquid compounds were added *via* syringe after setting the capped vial under an O_2 atmosphere (highly viscous liquids were added before capping the vial as well). The reaction mixture was stirred under 1 bar of O_2 using an O_2-filled balloon and under light irradiation using a 455 nm (± 15 nm) LED for 24 h at 40 °C using a cryostat. Four reaction batches were combined, filtered and concentrated under reduced pressure. The crude product was

purified by automated flash column chromatography using a petrolether/ethyl acetate mixture. Note: in order to get better yields, dry acetonitrile and freshly activated 4 Å molecular sieves are mandatory.

2.4 General procedure for the synthesis of nitriles from aldehydes

A 5 mL crimp cap vial equipped with a magnetic stirring bar was loaded with TPP (20 μmol, 8.0 mg, 20 mol%), NH_2OH•HCl (0.3 mmol, 20.7 mg, 3 eq), NH_4Br (0.25 mmol, 25.0 mg, 2.5 eq), 4 Å molecular sieves (25 mg), the corresponding aldehyde (0.1 mmol, 1 eq.) and dry ACN (2 mL, 0.05M). In doing so, all solid compounds were added before capping the vial, whereas all liquid compounds were added *via* syringe after setting the capped vial under N_2 atmosphere (highly viscous liquids were added before capping the vial as well). The reaction mixture was stirred under light irradiation using a 455 nm (± 15 nm) LED for 24 h at 40 °C using a cryostat. Four reaction batches were combined, filtered and concentrated under reduced pressure. The crude product was purified by automated flash column chromatography using a petrolether/ethyl acetate mixture.

2.5 General procedure for the synthesis of nitriles from oximes

A 5 mL crimp cap vial equipped with a magnetic stirring bar was loaded with TPP (20 μmol, 8.0 mg, 20 mol%), 4 Å molecular sieves (25 mg), the corresponding oxime (0.1 mmol, 1 eq.) and dry ACN (2 mL, 0.05M). In doing so, all solid compounds were added before capping the vial, whereas all liquid compounds were added *via* syringe after setting the capped vial under a N_2 atmosphere (highly viscous liquids were added before capping the vial as well). The reaction mixture was stirred under light irradiation using a 455 nm (± 15 nm) LED for 24 h at 40 °C using a cryostat. Four reaction batches were combined, filtered and concentrated under reduced pressure. The crude product was purified by automated flash column chromatography using a petrolether/ethyl acetate mixture.

2.6 General procedure for gram scale reactions

A 200 mL glass reactor equipped with a magnetic stirring bar was loaded with TPP (20 mol% for 1 g of substrate), NH_2OH•HCl (3 eq for 1 g of substrate), NH_4Br (2.5 eq for 1 g of substrate), 4 Å molecular sieves (25 mg for 0.1 mmol of substrate), the corresponding methylarene (1 g, 1 eq.) and dry ACN (0.05 M). In doing so, all solid compounds were added first to the glass reactor, whereas all liquid compounds were added at the end under O_2 or air atmosphere. The reaction setup was connected to two balloons holding

each 1 bar of oxygen (please see Fig. S2) and the mixture was stirred under light irradiation using a 455 nm (± 15 nm) LED for 36 h at 40 °C using a cryostat. The reaction progress was monitored by GC-FID. After the completion of the reaction, the reaction mixture was filtered over a celite bed and concentrated under reduced pressure. The crude product was purified by automated flash column chromatography using a petrolether/ethyl acetate mixture.

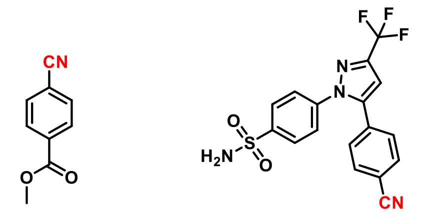

2: 65% (1 g scale) 38: 60% (1 g scale)

Scheme S1. Gram-scale synthesis

Reaction conditions: [a]1 g substrate, 20 mol% PC, 3 eq. NH_2OH•HCl, 2.5 eq. NH_4Br, 25 mg 4 Å MS for 0.1 mmol, 1 bar O_2, acetonitrile (0.05 M), 455 nm, 40 °C, 36 h, isolated yields.

3. Reaction optimization

Table S1. Screening of different photocatalysts and ammonia source

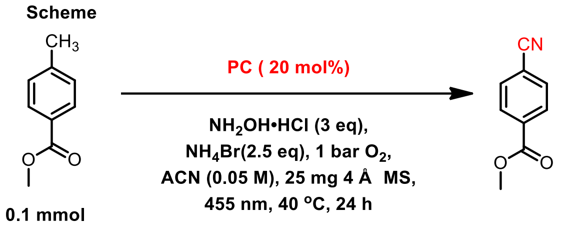

Entry	Photocatalyst (PC)	Ammonia source	Yield of product (%)
**1**	**PC-4**	$NH_2OH{\bullet}HCl$	**76**
2	PC-5	$NH_2OH{\bullet}HCl$	15
3	PC-6	$NH_2OH{\bullet}HCl$	35
4	PC-7	$NH_2OH{\bullet}HCl$	12
5	PC-4	$HCOONH_4$	NR
6	PC-4	7N NH_3 in MeOH	NR

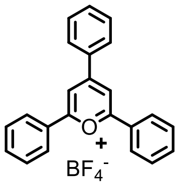

PC-4 (TPP)

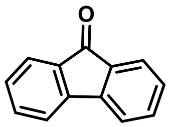

PC-5

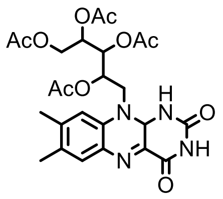

PC-6

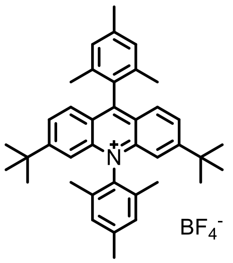

PC-7

Reaction conditions: [a]0.1 mmol substrate, 20 mol% PC, 3 eq. $NH_2OH{\bullet}HCl$, 2.5 eq. NH_4Br, 25 mg 4 Å MS, 1 bar O_2, 2 mL solvent (0.05 M), 455 nm, 40 °C, 24 h, GC yields using n-decane as standard.

Table S2. Screening of different solvents

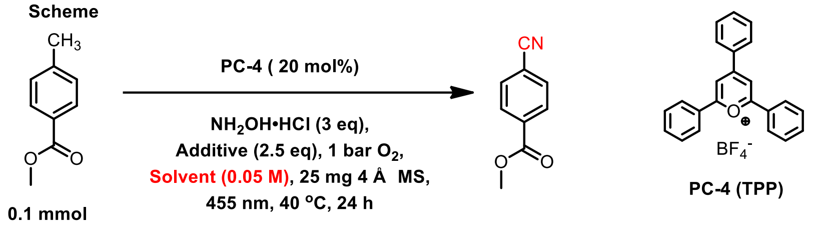

Entry	Solvent (0.05M conc.)	Yield of product (%)
**1**	**ACN**	**76**
2	Benzene	22
3	DMF	NR
4	DMSO	NR
5	DCM	3
6	TFE	12
7	Acetic acid	NR
8	ACN (0.1 M)	63
9	ACN (0.2 M)	39
10	ACN + 100 µL H_2O (without molecular sieves)	10

Reaction conditions: [a]0.1 mmol substrate, 20 mol% PC, 3 eq. NH_2OH•HCl, 2.5 eq. NH_4Br, 25 mg 4 Å MS, 1 bar O_2, 2 mL solvent (0.05 M), 455 nm, 40 °C, 24 h, GC yields using n-decane as standard.

Table S3. Screening of different additives

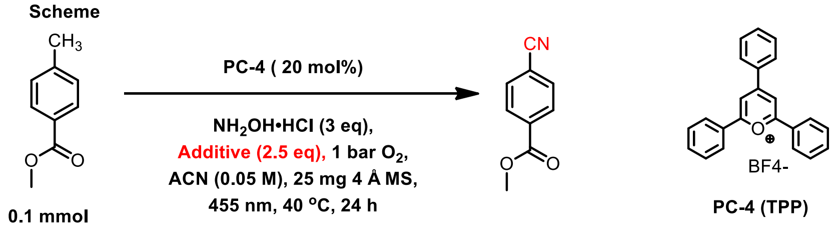

Entry	Ammonia source (3 eq)	Additive(2.5 eq)	Yield of product (%)
1	NH_2OH•HCl	NH_4Br	76
2	NH_2OH•HCl	TBABr	10
3	NH_2OH•HCl	$Sc(OTf)_3$	35
4	NH_2OH•HCl	TFA	10
5	NH_2OH•HCl	NH_4F	4
6	NH_2OH•HCl	NH_4Cl	32
7	NH_2OH•HCl	NH_4I	NR
8	NH_2OH•HCl	CBr_4	33
9	NH_2OH•HCl	KBr	36
10	NH_2OH•HCl	$LiNO_3$	18
11	NH_2OH•HCl	NH_4NO_3	30
12	NH_2OH•HCl	HBr in acetic acid	10

Reaction conditions: [a]0.1 mmol substrate, 20 mol% PC, 3 eq. NH_2OH•HCl, 2.5 eq. additive, 25 mg 4 Å MS, 1 bar O_2, 2 mL acetonitrile (0.05 M), 455 nm, 40 °C, 24 h, GC yields using n-decane as standard.

Table S4. Control experiments

Scheme

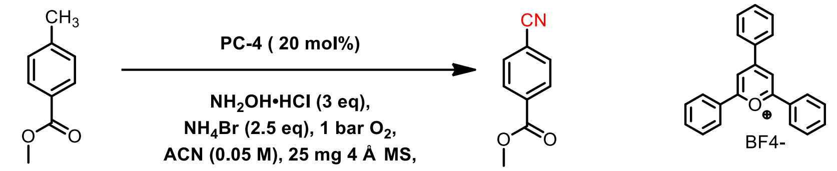

0.1 mmol

PC-4 (TPP)

Entry	PC-4 (20 mol%)	Light (455 nm)	NH_2OH•HCl (3 eq)	NH_4Br (2.5 eq)	4 Å MS (25 mg)	O_2 (1 bar)	Yield of product (%)
**1**	**YES**	**YES**	**YES**	**YES**	**YES**	**YES**	**76**
2	NO	YES	YES	YES	YES	YES	1
3	YES	NO	YES	YES	YES	YES	NR
4	YES	YES	NO	YES	YES	YES	2
5	YES	YES	YES	NO	YES	YES	25
6	YES	YES	YES	YES	NO	YES	51
7	YES	YES	YES	YES	YES	NO	NR

Reaction conditions: [a]0.1 mmol substrate, 20 mol% PC, 3 eq. NH_2OH•HCl, 2.5 eq. NH_4Br, 25 mg 4 Å MS, 1 bar O_2, 2 mL acetonitrile (0.05 M), 455 nm, 40 °C, 24 h, GC yields using n-decane as standard.

4. Mechanistic Investigation

4.1 Kinetic investigation

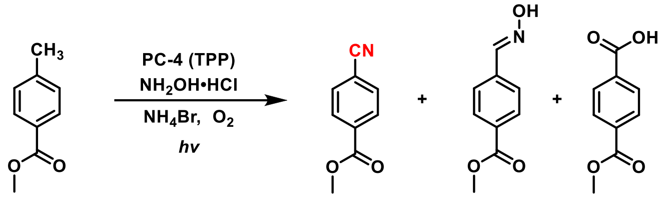

1 2 3 4

The kinetic investigations on this system were performed, examining the effect of (a) temperature, (b) ammonium bromide concentration, (c) hydroxylamine to substrate ratio, (d) reaction time, (e) concentration of the catalyst, and (f) wavelength (Fig. 1 and S3). The reaction temperature had a significant effect on the product yield, as shown in Fig. 1a. At 0 °C, product **2** together with **3** were observed in 42% and 38%, respectively. The oxime to nitrile conversion was increased by elevating the reaction temperature, revealing 40 °C as the optimum. A catalytic amount of ammonium bromide was sufficient for the complete conversion of starting material. However, to reach the maximum yield an excess of ammonium bromide is required (Fig. 1b). The absence or low concentration of hydroxylamine hydrochloride led to an increased amount of by-product **4** (Fig. 1c), whereas a loading of 3 eq. gave the product in a good yield. Regarding the reaction time (Fig. S3d), 30% of the starting material was converted after 2 h, with the major product being the oxime intermediate 3 (16%) and only small amounts of product (2). After 8 h, the starting material was almost completely converted. At this time, the oxime (3) concentration is beginning to decrease slowly, while the yield of the desired product 2 is steadily increasing. Varying the catalyst loading (Fig. S3e), the starting material was predominant with 2 mol%. For 5 mol% catalyst, product **2**, **3** and starting material **1** were almost at an equal level (28-38%) and the maximum product yield of 76% was obtained when the photocatalyst loading was raised to 20 mol%. When investigating the effect of different wavelength, we found that 365 nm and 455 nm LEDs are efficient light sources for this transformation (Fig. S3f).

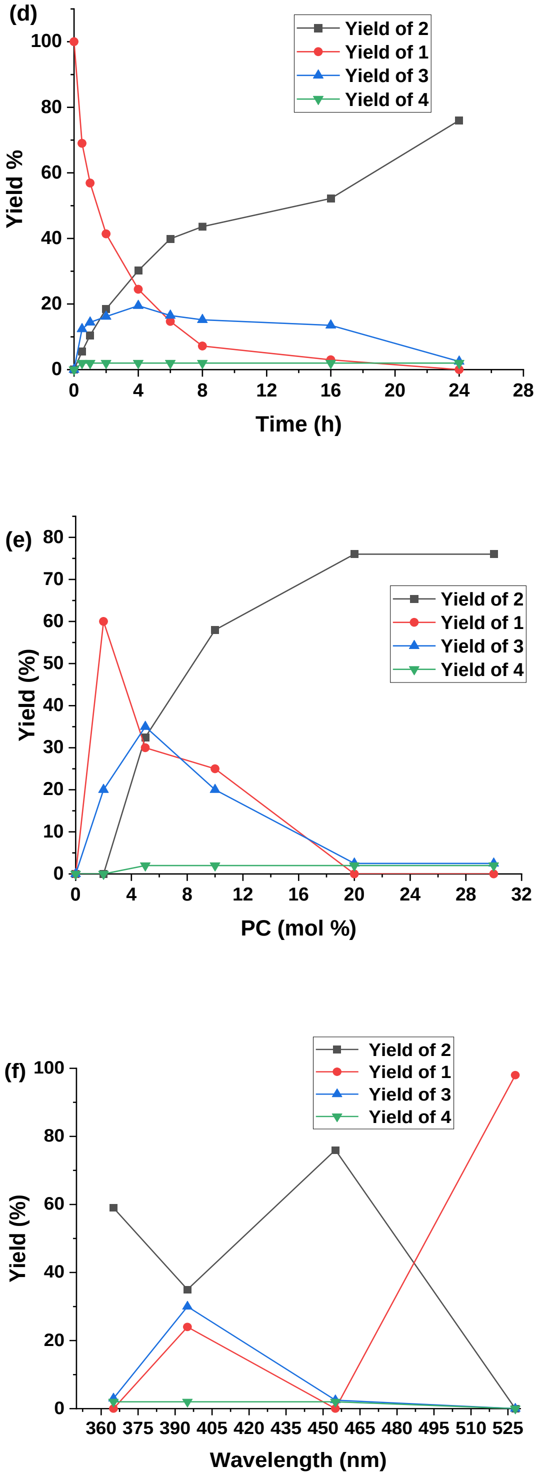

Fig. S3: Kinetic investigation on the photocatalytic ammoxidation**.** (d) Yield vs time, (e) yield vs concentration of TPP, (f) yield vs wavelength. Reaction conditions: For Fig. S3 (d): 0.1 mmol substrate, 20 mol% PC, 3 eq. NH_2OH•HCl, 2.5 eq. NH_4Br, 25 mg 4 Å MS, 1 bar O_2, 2 mL acetonitrile (0.05 M), 455 nm, 40 °C, 0-24 h. Fig.

S3 (e): 0.1 mmol substrate, 2-20 mol% PC, 3 eq. NH_2OH•HCl, 2.5 eq. NH_4Br, 25 mg 4 Å MS, 1 bar O_2, 2 mL acetonitrile (0.05 M), 455 nm, 40 °C, 24 h. Fig. S3 (f): 0.1 mmol substrate, 20 mol% PC, 3 eq. NH_2OH•HCl, 2.5 eq. NH_4Br, 25 mg 4 Å MS, 1 bar O_2, 2 mL acetonitrile (0.05 M), 365-528 nm, 40 °C, 24 h. Yields were determined by GC using n-decane as standard.

4.2 Time-resolved Luminescence Quenching studies

A linear correlation between concentration of quencher [Q] and $\tau_0 \times \tau^{-1}$ indicates a dynamic luminescence quenching. The luminescence lifetime was recorded in dry ACN using a quartz cuvette (1×1 cm) with septum screw cap. The cuvette was degassed *in vacuo* and backfilled with N_2 (5×) before the stock solution of quencher and the catalyst solution were added *via* syringe. For excitation of the sample, a 452 nm laser diode was used and an optical longpass filter (cut-on wavelength 500 nm) was installed before the detection unit. The time range for the measurement was set to 100 ns. The experimental data were fitted with a mono-exponential function. For liquid quenchers, the substrate was added as such. For solid quenchers, a stock solution in ACN with defined concentration was prepared. In case of NH_2OH•HCl and NH_4Br 1 mmol was taken in 1 mL ACN and stirred at 40° C for 15 minutes, then passed through a filter pad and the clear solution was used for quenching studies. We suspect that the resulting non-linear behavior is due to the poor solubility of ammonium salts in acetonitrile (1 mg of ammonium salt dissolves in 10 mL of acetonitrile). In order to support this, we performed the quenching studies of NH_4Br and NH_2OH•HCl using an ACN/H_2O (1:1) mixture as solvent. The thus obtained data showed a linear correlation between the life-time ratio (τ_0/τ) and the quencher concentration.

The corresponding graphs and/or Stern-Volmer plots of the conducted quenching experiments are given below (Fig S4-10). In case of a linear relation, the Stern-Volmer constant (K_{SV}) was determined from the slope of the linear fit:

$$\frac{\tau_0}{\tau} - 1 = K_{SV} \cdot [Q]$$

With τ_0 being the luminescence lifetime in absence of the quencher, τ the luminescence lifetime in presence of the quencher and [Q] the quencher concentration.

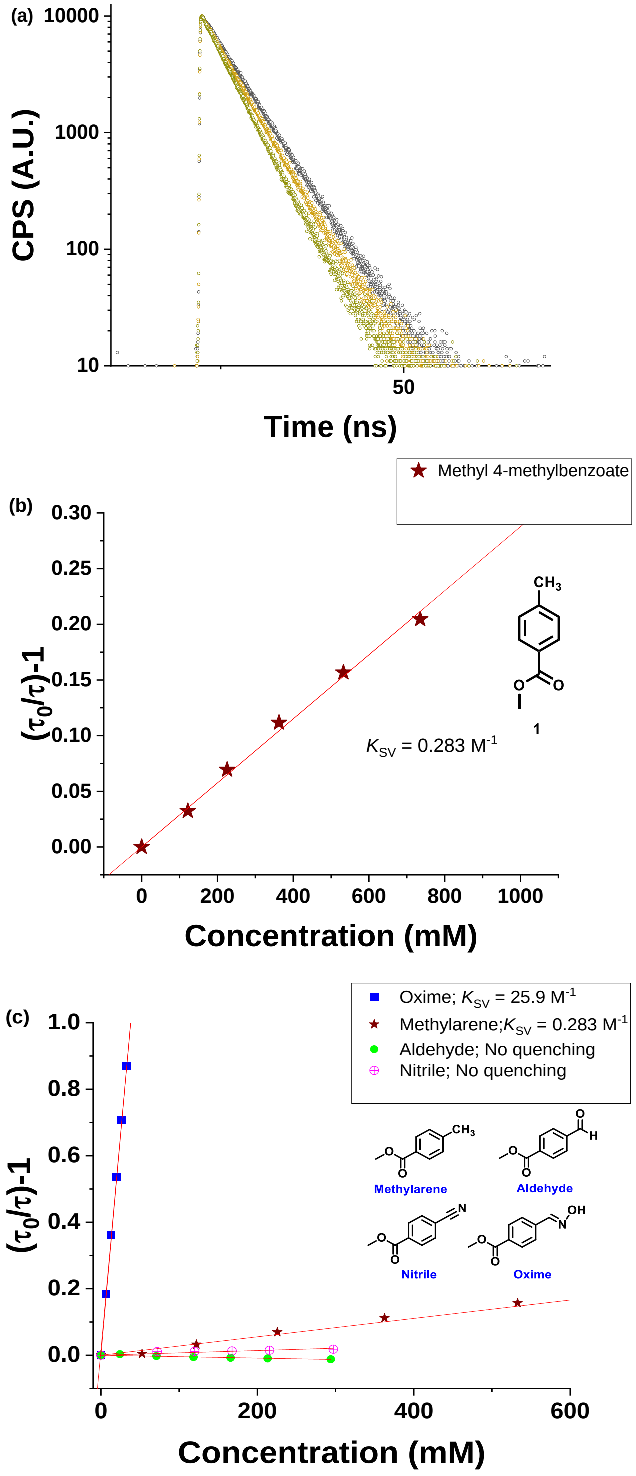

Fig. S4: Quenching studies. (a) Time-resolved luminescence quenching decay of TPP in presence of methyl 4-methylbenzoate at different concentrations; (b) Stern-Volmer plot for the TPP quenching with methyl 4-methylbenzoate; (c) Stern-Volmer plot comparing potential TPP quencher.

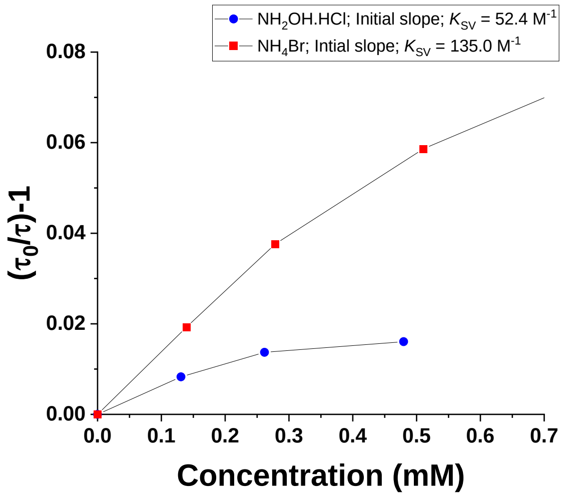

Fig. S5: Stern-Volmer plot developed with data obtained from time-resolved quenching experiments of TPP (PC-4) with NH_2OH•HCl and NH_4Br in ACN.

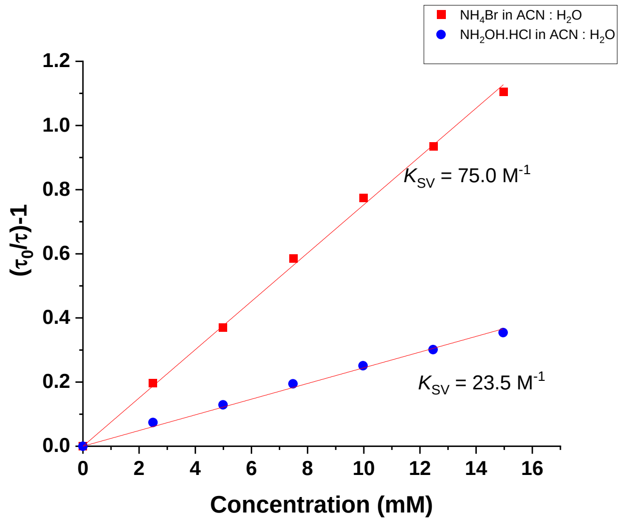

Fig. S6: Stern-Volmer plot developed with data obtained from time-resolved quenching experiments of TPP (PC-4) with NH_2OH•HCl and NH_4Br in ACN:Water.

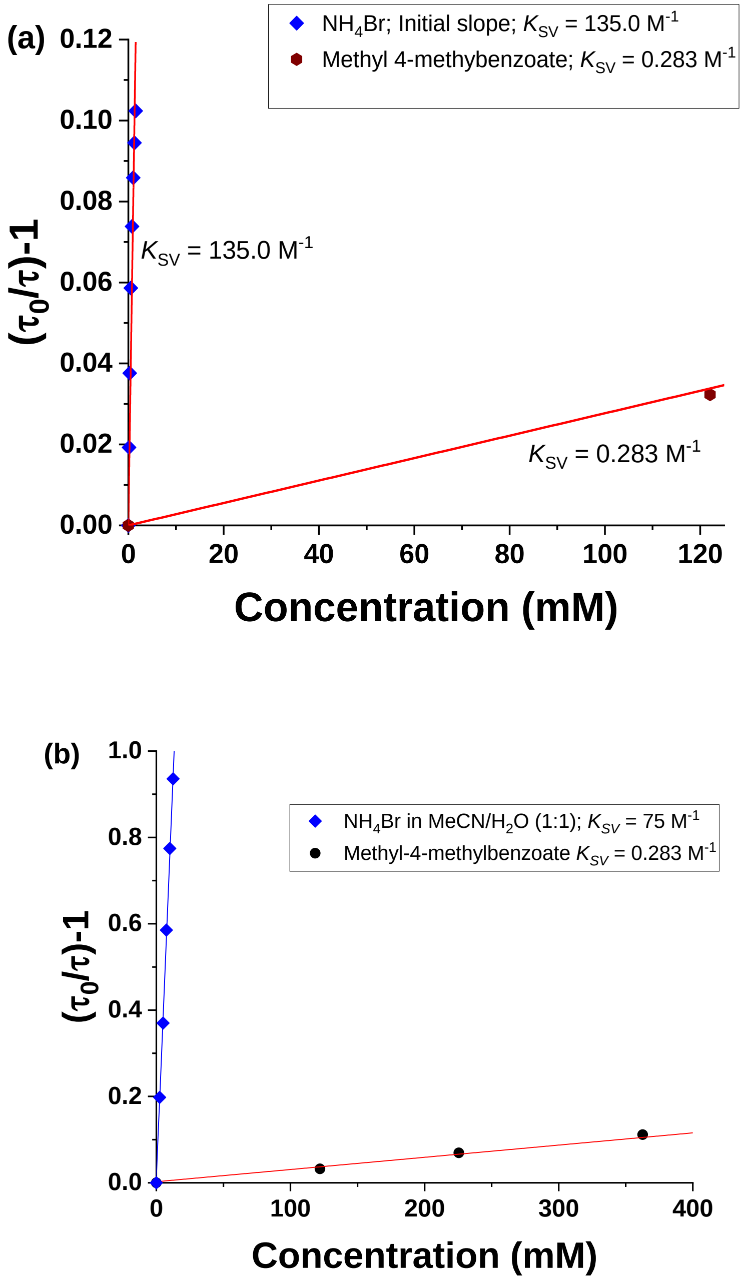

Fig. S7: Stern-Volmer plot developed with data obtained from time-resolved quenching experiments of TPP (PC-4) with (a) methyl-4-methylbenzoate in ACN and NH_4Br in ACN and (b) methyl-4-methylbenzoate in ACN and NH_4Br in ACN:H_2O (1:1).

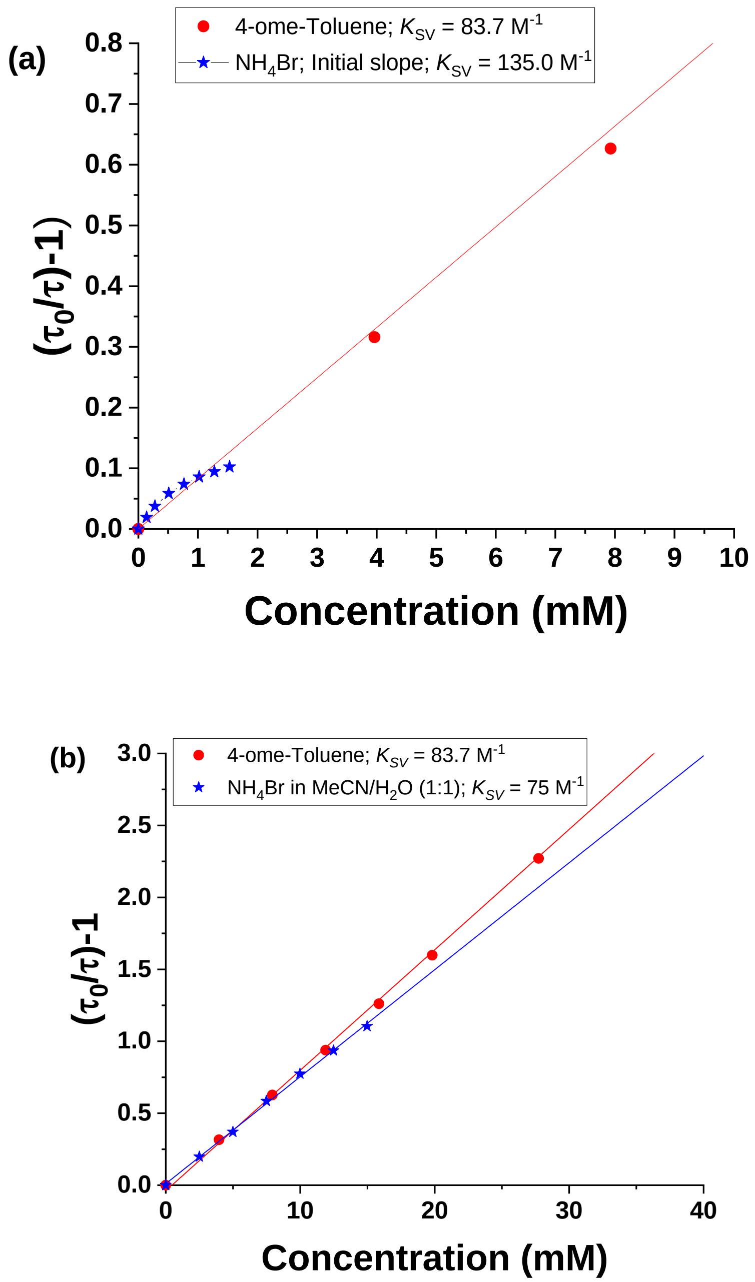

Fig. S8: Stern-Volmer plot developed with data obtained from time-resolved quenching experiments of TPP (PC-4) with (a) 4-methoxy toluene in ACN and NH_4Br in ACN and (b) 4-methoxy toluene in ACN and NH_4Br in ACN:H_2O (1:1).

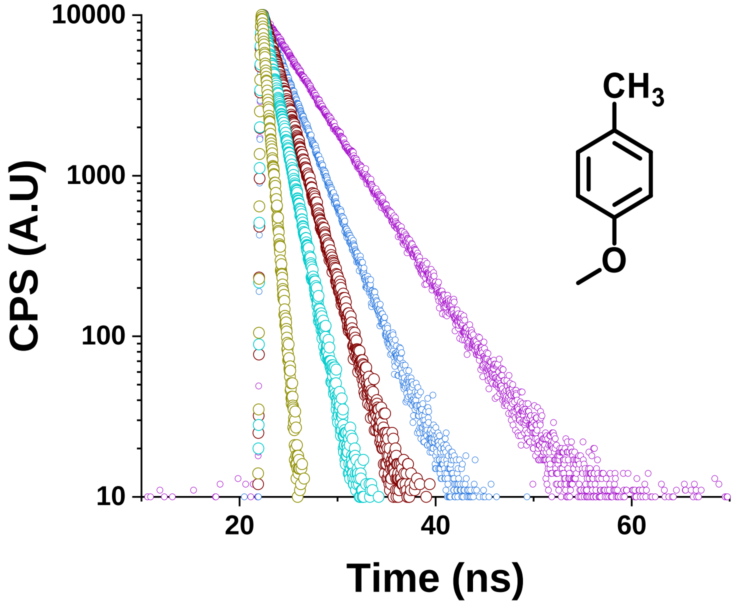

Fig. S9: Luminescence decay of 4-methoxy toluene with TPP (PC-4). The lifetime was determined by employing an exponential fit function.

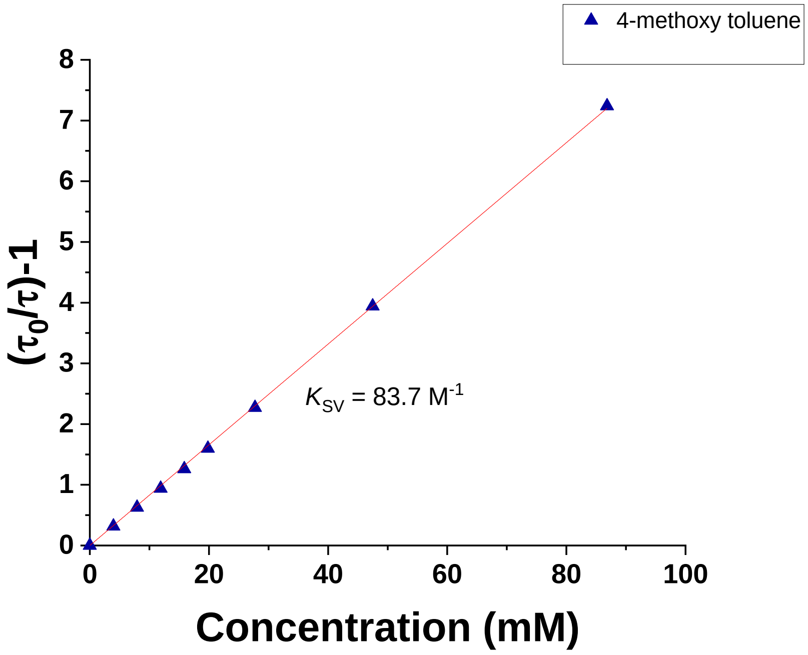

Fig. S10: Stern-Volmer plot developed with data obtained from time-resolved quenching experiments of TPP (PC-4) with 4-methoxy toluene.

4.3 Mechanistic experiments

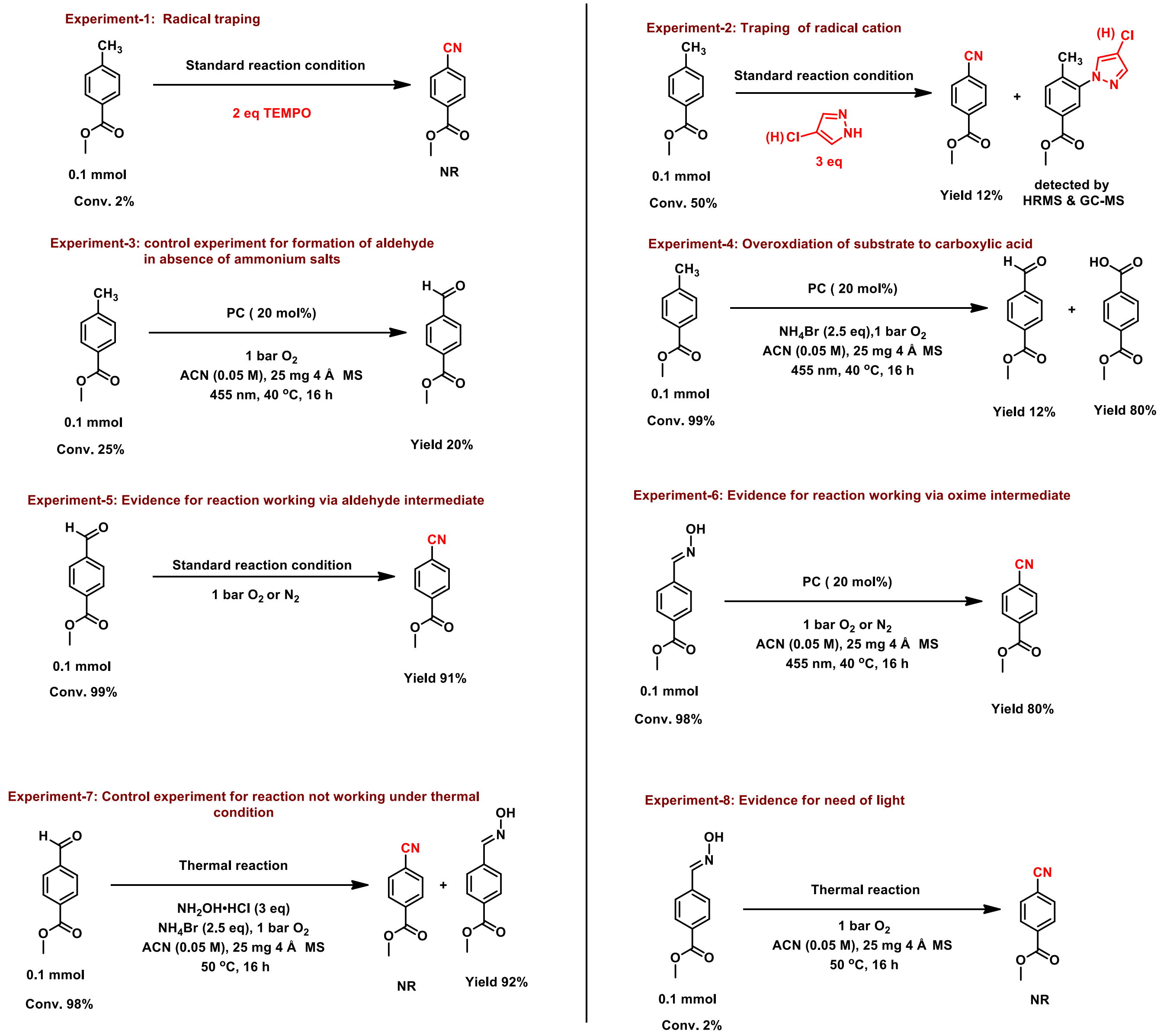

Scheme S2. Mechanistic experiments to support the proposed catalytic hypothesis

4.4 Trapping experiments[5]

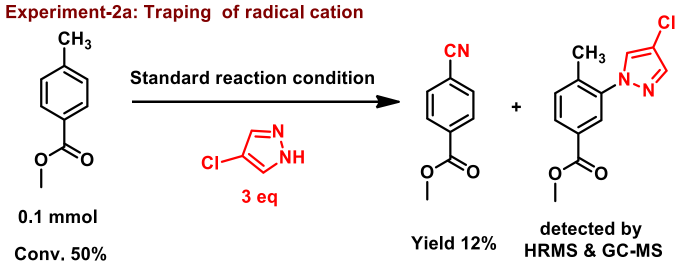

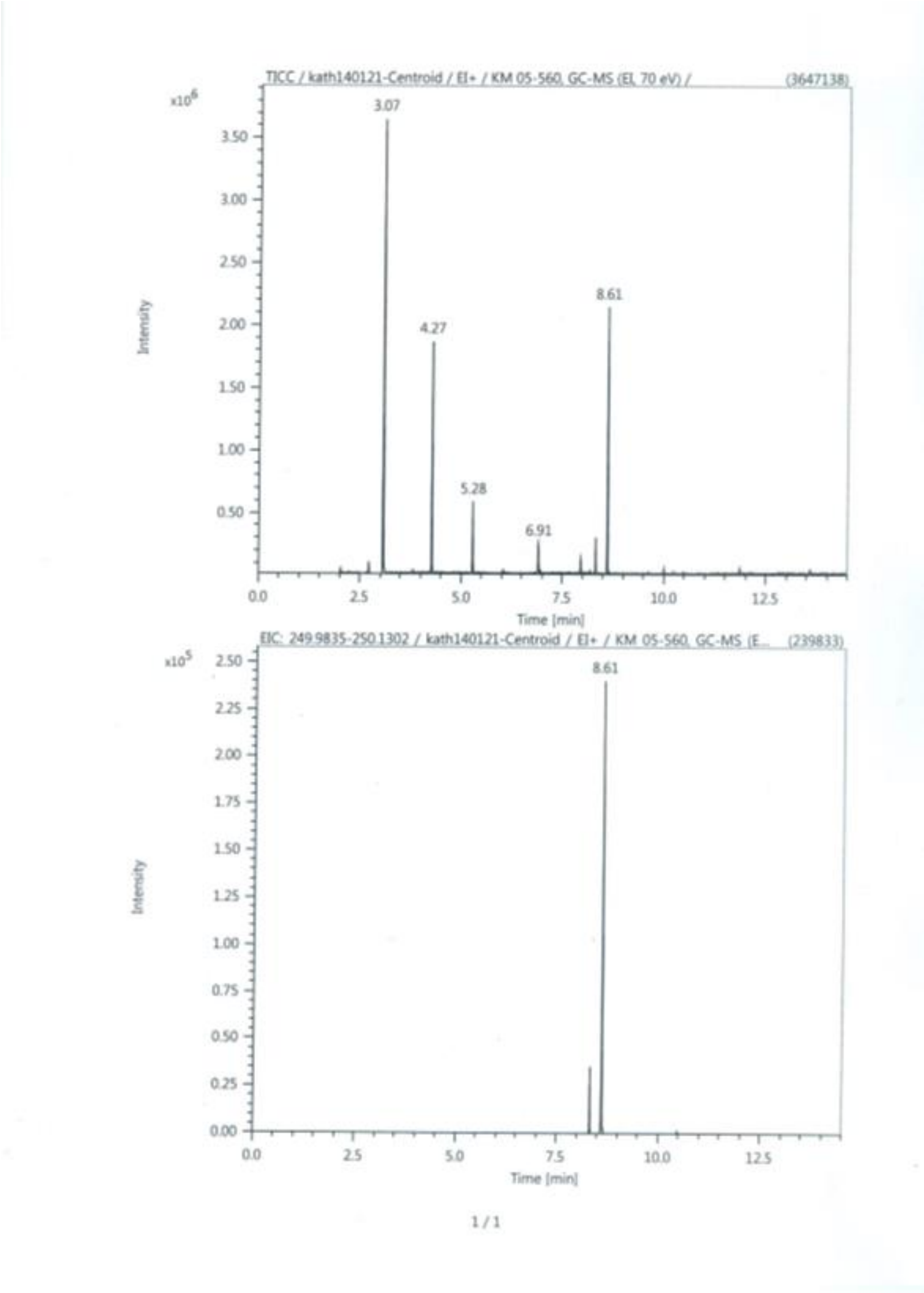

Spectrum

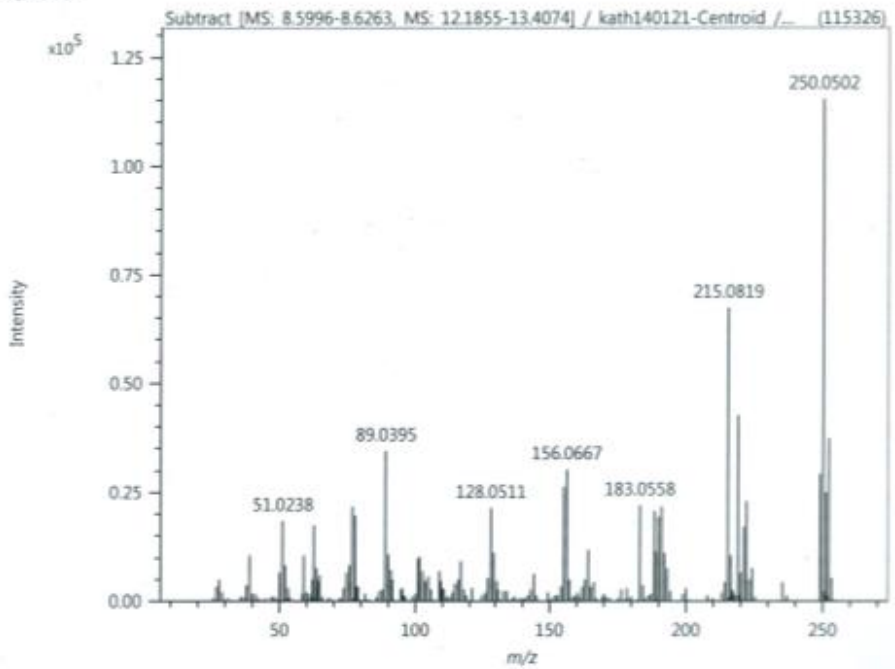

Elemental Composition

Parameters	
Tolerance:	15.00 ppm
Electron:	Odd/Even
Charge:	+1
DBE:	-1.5 - 50.0

Elements:

Symbol	C	H	N	O	S	Cl	B	Bx
Min	1	1	0	0	0	0	0	0
Max	100	100	3	3	0	3	0	0

Symbol	F
Min	0
Max	0

Results

Mass	Intensity	Formula	Calculated Mass	Mass Difference [mDa]	Mass Difference [ppm]	DBE
249.04264	29291.78	C12 H10 N2 O2 Cl	249.04253	0.11	0.45	8.5
		C9 H13 N3 O Cl2	249.04302	-0.38	-1.51	4.0
		C15 H7 N O3	249.04204	0.60	2.40	13.0
		C11 H15 O2 Cl2	249.04436	-1.72	-6.90	3.5
		C18 H5 N2	249.04472	-2.08	-8.36	17.5
		C8 H18 N O Cl3	249.04485	-2.21	-8.86	-1.0
250.05017	115325.67	C12 H11 N2 O2 Cl	250.05036	-0.19	-0.74	8.0
		C15 H8 N O3	250.04987	0.30	1.20	12.5
		C9 H14 N3 O Cl2	250.05084	-0.67	-2.69	3.5
		C11 H16 O2 Cl2	250.05219	-2.02	-8.06	3.0
		C18 H6 N2	250.05255	-2.38	-9.51	17.0

1 / 1

Fig. S11: GC-MS and HRMS spectra of 4-chloro pyrazole trapped product under standard reaction condition

Experiment-2b: Traping of radical cation

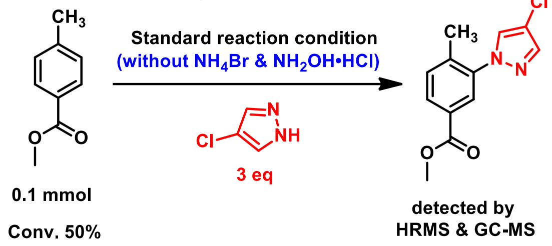

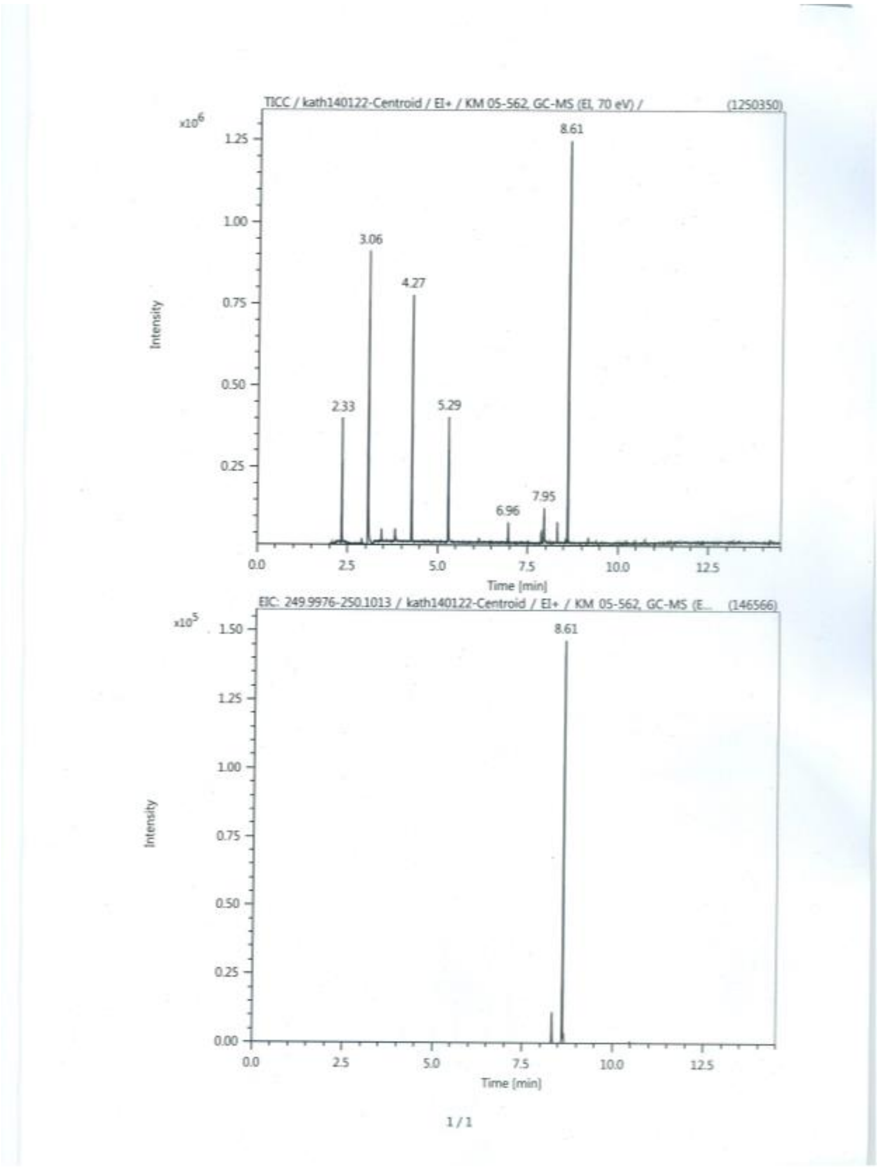

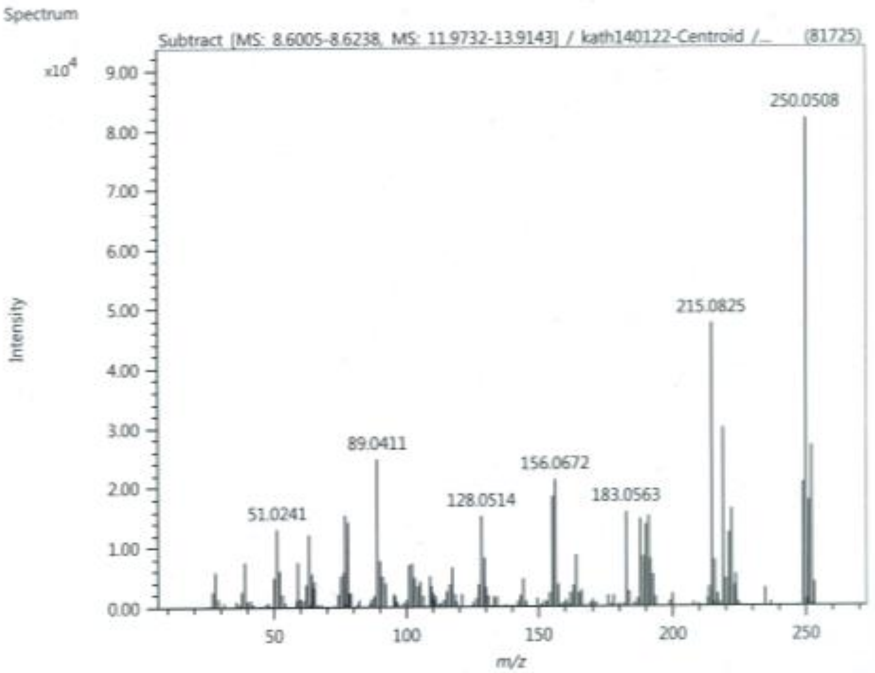

Elemental Composition

Parameters	
Tolerance:	15.00 ppm
Electron:	Odd/Even
Charge:	+1
DBE:	-1.5 - 50.0

Elements:

Symbol	C	H	N	O	S	Cl	B	Bx
Min	1	1	0	0	0	0	0	0
Max	100	100	3	3	0	3	0	0

Symbol	F
Min	0
Max	0

Results

Mass	Intensity	Formula	Calculated Mass	Mass Difference [mDa]	Mass Difference [ppm]	DBE
249.04319	20812.63	C9 H13 N3 O Cl2	249.04302	0.17	0.70	4.0
		C12 H10 N2 O2 Cl	249.04253	0.66	2.66	8.5
		C15 H7 N O3	249.04204	1.15	4.61	13.0
		C11 H15 O2 Cl2	249.04436	-1.17	-4.69	3.5
		C18 H5 N2	249.04472	-1.53	-6.15	17.5
		C8 H18 N O Cl3	249.04485	-1.66	-6.65	-1.0
		C17 H10 Cl	249.04655	-3.36	-13.50	12.5
250.05078	81724.75	C9 H14 N3 O Cl2	250.05084	-0.06	-0.25	3.5
		C12 H11 N2 O2 Cl	250.05036	0.42	1.70	8.0
		C15 H8 N O3	250.04987	0.91	3.65	12.5
		C11 H16 O2 Cl2	250.05219	-1.41	-5.62	3.0

1 / 2

Fig. S12: GC-MS and HRMS spectra of 4-chloro pyrazole trapped product without NH_4Br and $NH_2OH•HCl$

Experiment-2c: Traping of radical cation

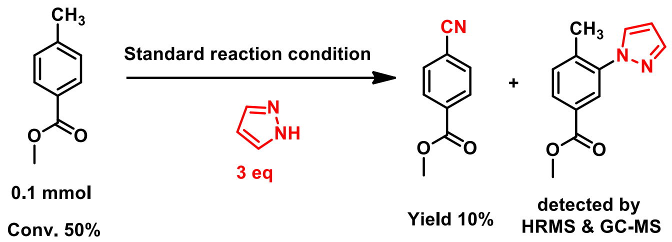

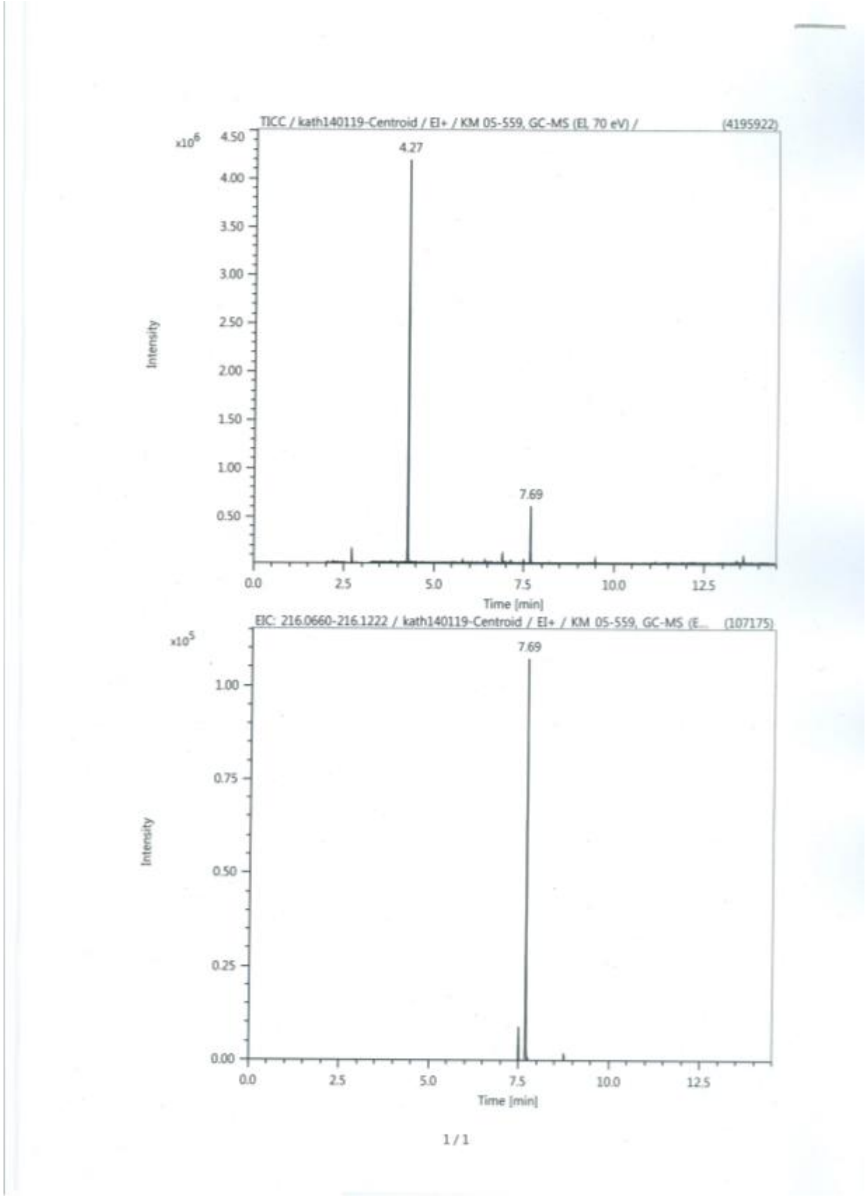

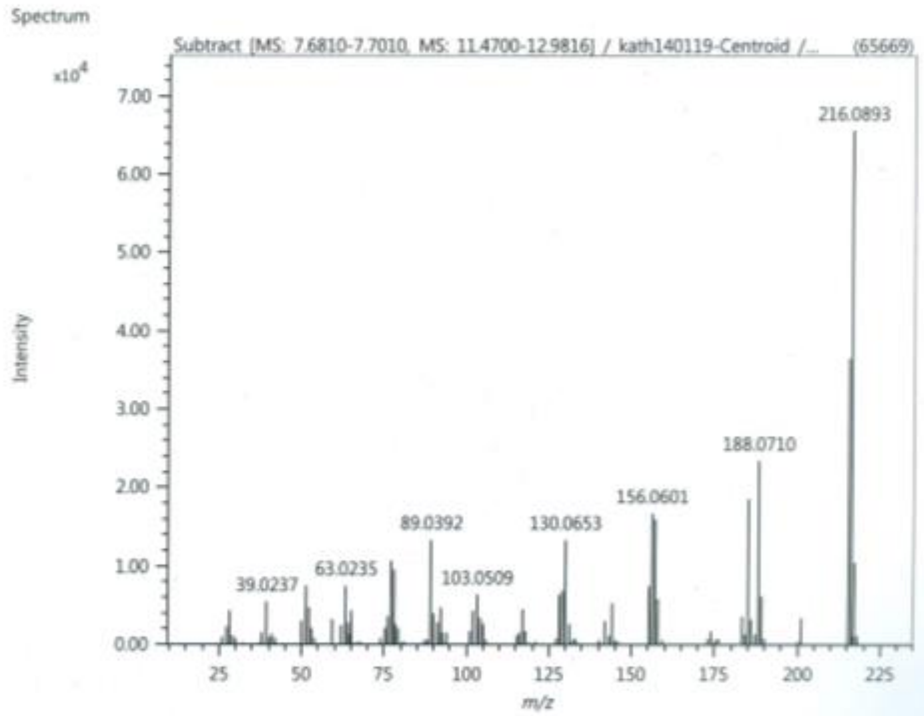

Elemental Composition

Parameters

Tolerance:	15.00 ppm
Electron:	Odd/Even
Charge:	+1
DBE:	-1.5 - 50.0

Elements:

Symbol	C	H	N	O	S	Cl	B	Bx
Min	1	1	0	0	0	0	0	0
Max	100	100	5	5	0	0	0	0

Symbol	F
Min	0
Max	0

Results

Mass	Intensity	Formula	Calculated Mass	Mass Difference [mDa]	Mass Difference [ppm]	DBE
215.08165	36668.71	C12 H11 N2 O2	215.08150	0.14	0.67	8.5
		C10 H9 N5 O	215.08016	1.49	6.91	9.0
		C9 H13 N O5	215.07882	2.82	13.13	4.0
216.08930	65669.14	C12 H12 N2 O2	216.08933	-0.03	-0.15	8.0
		C10 H10 N5 O	216.08799	1.31	6.06	8.5
		C9 H14 N O5	216.08665	2.65	12.25	3.5

1 / 1

Fig. S13: GC-MS and HRMS spectra of pyrazole trapped product under standard reaction condition

Experiment-2d: Traping of radical cation

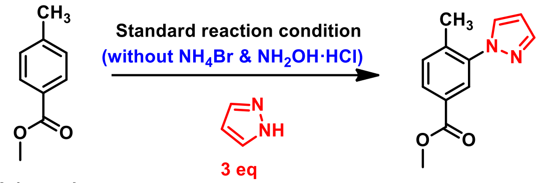

0.1 mmol

Conv. 50%

detected by
HRMS & GC-MS

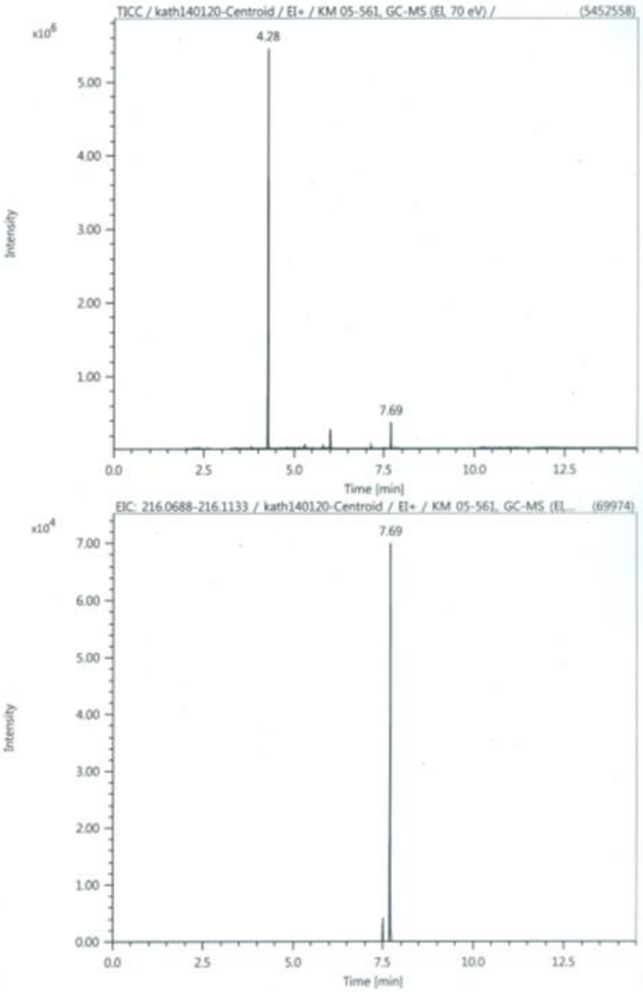

1 / 2

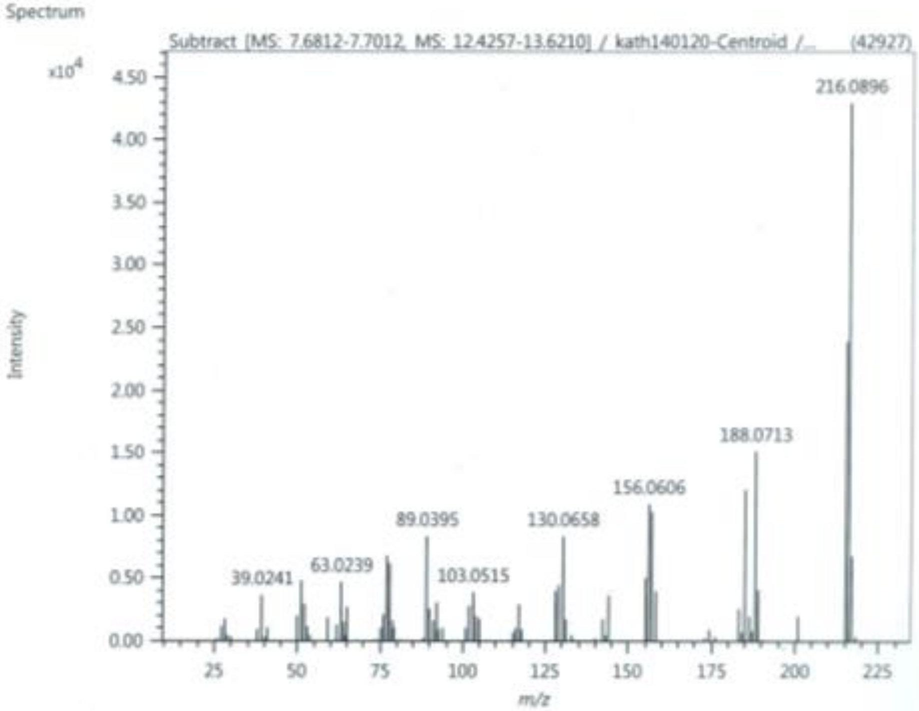

Elemental Composition

Parameters

Tolerance:	15.00 ppm
Electron:	Odd/Even
Charge:	+1
DBE:	-1.5 - 50.0

Elements:

Symbol	C	H	N	O	S	Cl	B	Bx
Min	1	1	0	0	0	0	0	0
Max	100	100	5	5	0	0	0	0

Symbol	F
Min	0
Max	0

Results

Mass	Intensity	Formula	Calculated Mass	Mass Difference [mDa]	Mass Difference [ppm]	DBE
215.08202	23973.43	C12 H11 N2 O2	215.08150	0.52	2.42	8.5
		C10 H9 N5 O	215.08016	1.86	8.66	9.0
		C9 H13 N O5	215.07882	3.20	14.88	4.0
216.08962	42926.57	C12 H12 N2 O2	216.08933	0.29	1.33	8.0
		C10 H10 N5 O	216.08799	1.63	7.55	8.5
		C9 H14 N O5	216.08665	2.97	13.74	3.5

1 / 1

Fig. S14: GC-MS and HRMS spectra of 4-chloro pyrazole trapped product without NH_4Br and $NH_2OH{\bullet}HCl$

4.5 NMR studies

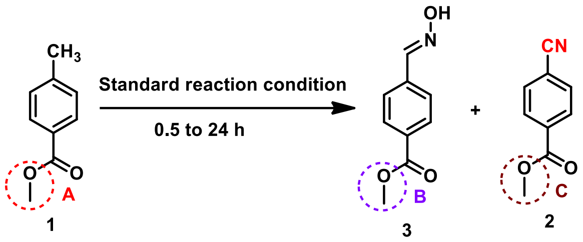

In order to follow the mechanistic pathway in more detail, NMR studies were performed analysing the reaction mixture after defined time intervals (Fig. S16). The signal corresponding to the methyl ester (-COOCH$_3$) of the starting material (1) shows a singlet resonance at δ 3.84 ppm in the ^{1}H-NMR spectrum, while the oxime (3) gives a singlet at δ 3.87, and nitrile (2) a singlet at δ 3.91, allowing for a facile distinction between the species of interest. At the beginning, the depletion of the starting material is accompanied by the increase of the oxime intermediate as well as nitrile product, with all three compounds being clearly observable at the same time after 4 h of irradiation. Further increasing the reaction time leads to the (almost) full conversion of the starting material and the oxime with the dominant signal being the product peak. A similar tendency was observed in the ^{13}C-NMR for the corresponding methyl ester carbon (Fig. S18), all together further supporting the formation of the oxime as crucial intermediate to render the nitrile.

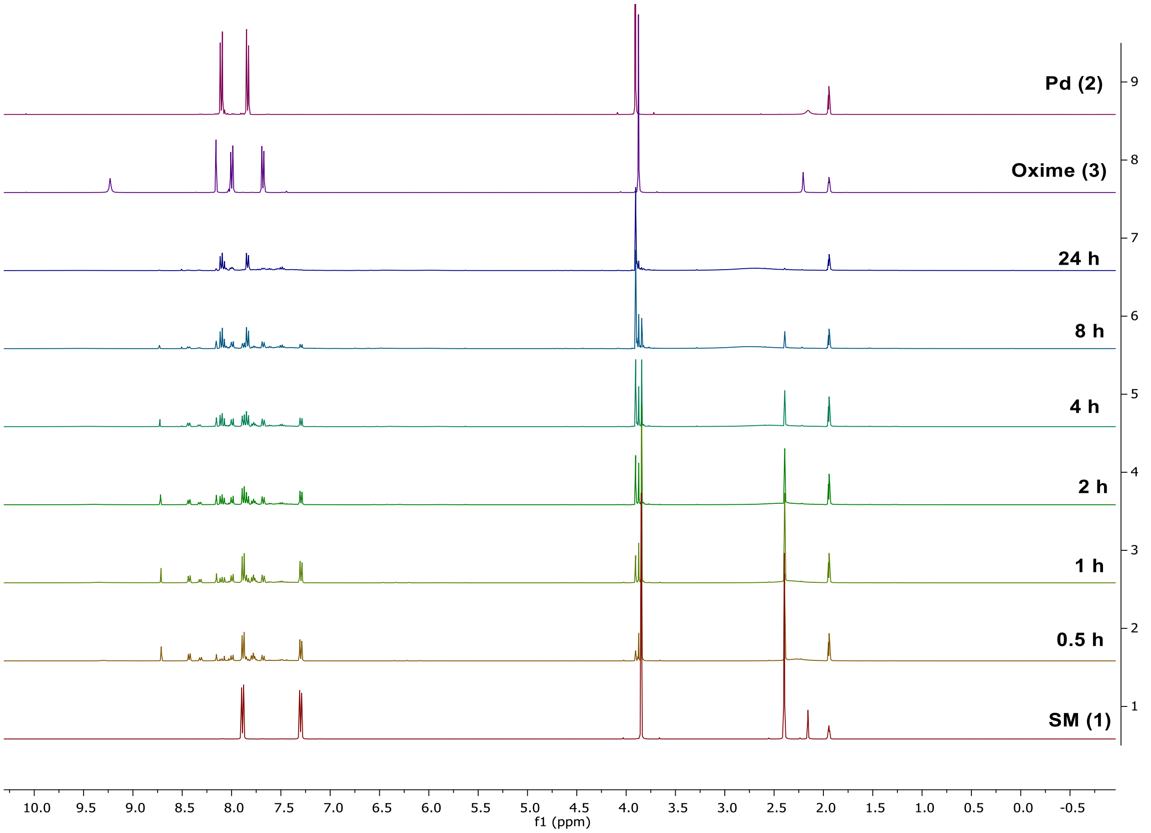

Fig. S15: ^{1}H NMR spectra for different time interval using CD_3CN as a solvent.

Reaction conditions: [a]0.1 mmol substrate, 20 mol% PC, 3 eq. $NH_2OH•HCl$, 2.5 eq. NH_4Br, 25 mg 4 Å MS, 1 bar O_2, 1 mL CD_3CN (0.1 M), 455 nm, 40 °C, 0.5-24 h. After each time interval, the reaction mixture was filtered through filter pad and the NMR of the solution was measured as such.

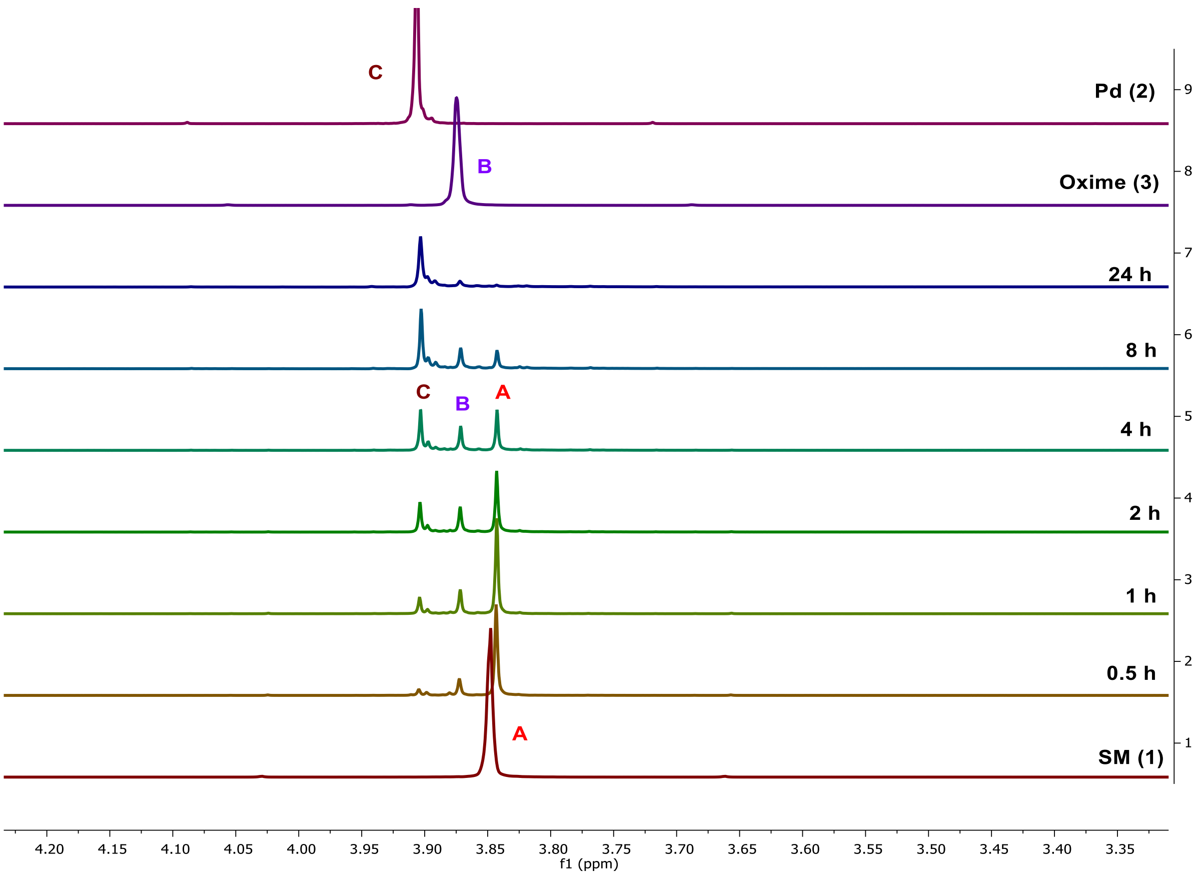

Fig. S16: ^{1}H NMR spectra for different time interval using CD_3CN as a solvent.

Reaction conditions: [a]0.1 mmol substrate, 20 mol% PC, 3 eq. NH_2OH•HCl, 2.5 eq. NH_4Br, 25 mg 4 Å MS, 1 bar O_2, 1 mL CD_3CN (0.1 M), 455 nm, 40 °C, 0.5-24 h. After each interval reaction mixture was filtered through filtered pad and measured as such a for NMR analysis.

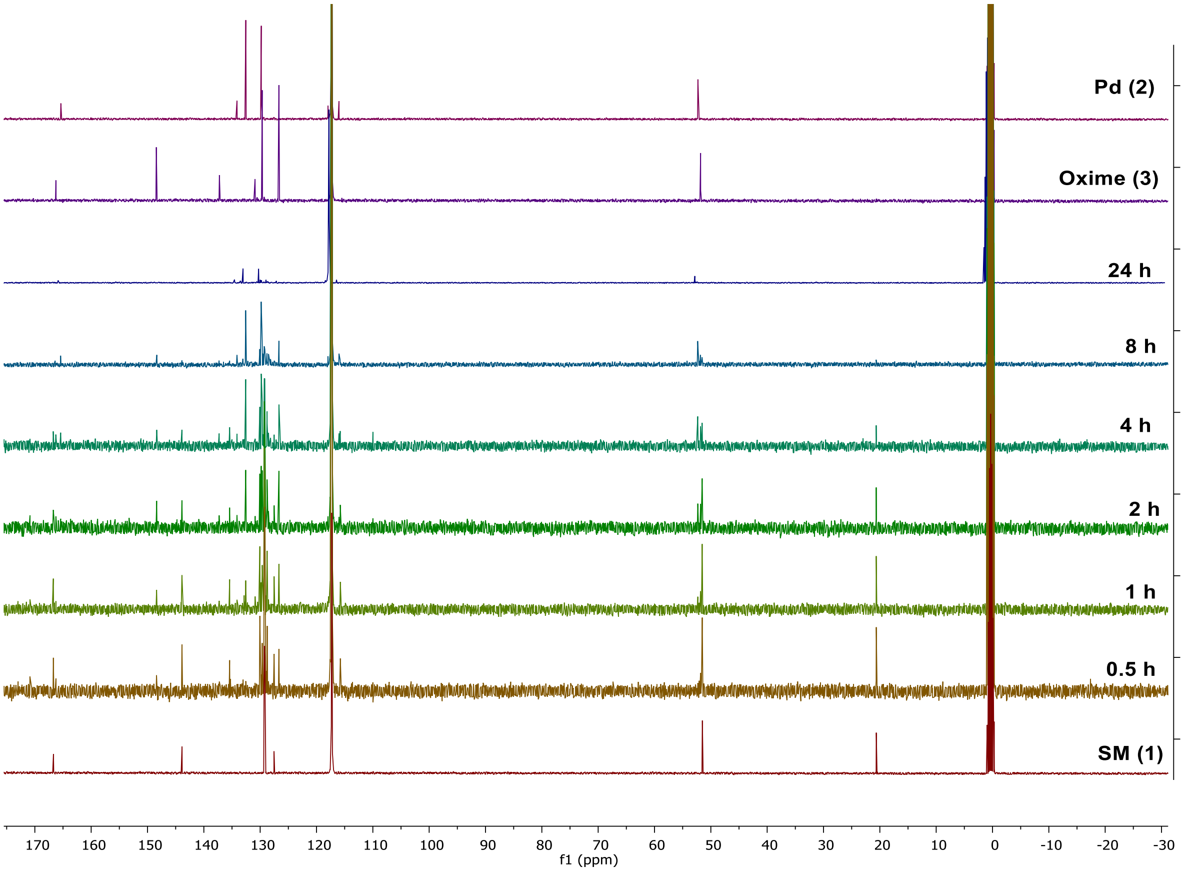

Fig. S17: ^{13}C NMR spectra for different time interval using CD_3CN as a solvent.

Reaction conditions: [a]0.1 mmol substrate, 20 mol% PC, 3 eq. NH_2OH•HCl, 2.5 eq. NH_4Br, 25 mg 4 Å MS, 1 bar O_2, 1 mL CD_3CN (0.1 M), 455 nm, 40 °C, 0.5-24 h. After each interval reaction mixture was filtered through filtered pad and measured as such a for NMR analysis.

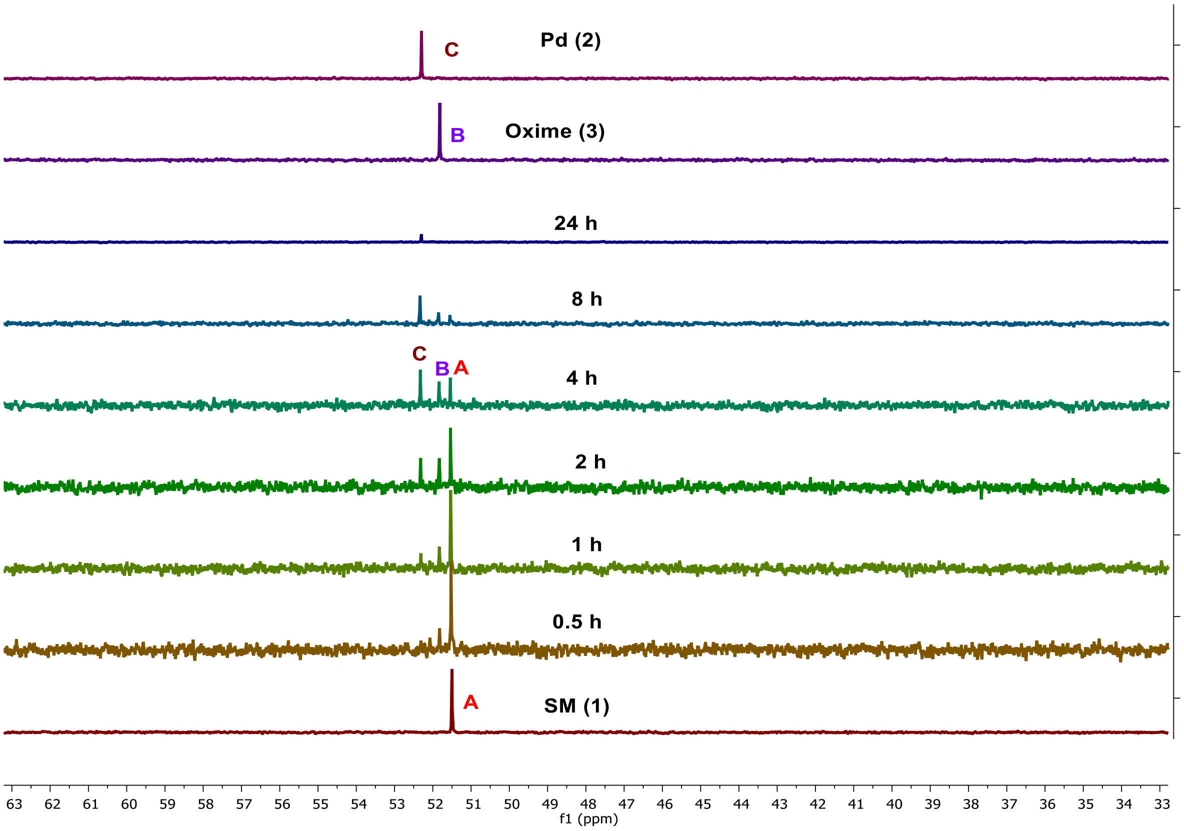

Fig. S18: ^{13}C NMR spectra for different time interval using CD_3CN as a solvent.

Reaction conditions: [a]0.1 mmol substrate, 20 mol% PC, 3 eq. NH_2OH•HCl, 2.5 eq. NH_4Br, 25 mg 4 Å MS, 1 bar O_2, 1 mL CD_3CN (0.1 M), 455 nm, 40 °C, 0.5-24 h. After each interval reaction mixture was filtered through filtered pad and measured as such a for NMR analysis.

4.6 The product yield on irradiation density

The irradiation power of blue LED was adjusted in Photoreactor TAK 120 by turning the voltage or current knob. PowerMax USB-PS19Q Power Sensor was also used to verify the emitted light power.

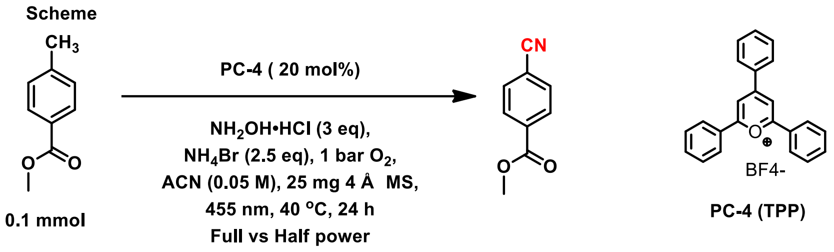

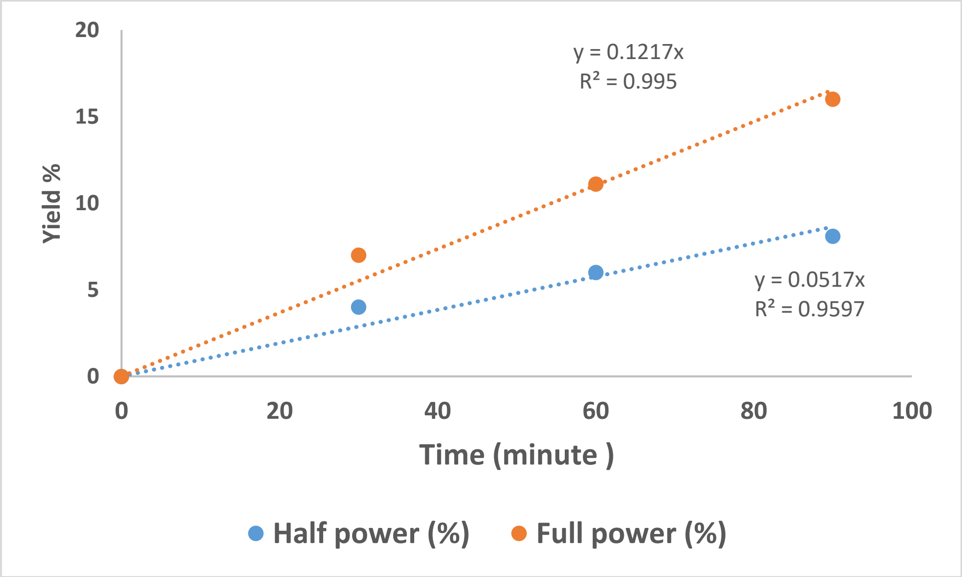

Fig. S19: Two, according to the standard reaction conditions (Section 2.3) identically prepared samples, containing methyl 4-methylbenzoate (1 equiv.), TPP (20 mol%), NH_4Br (2.5 eq.), NH_2OH•HCl (3 eq), 4 Å molecular sieves (25 mg) and 2 mL ACN, were irradiated with the same blue LED using different intensities. To exclude other influencing factors, such as, adverse effects of prolonged reaction time, the reactions were irradiated only for 90 minutes. After 30, 60 and 90 minutes, the irradiation was stopped for both reactions. Yields to the given time were determined by GC analysis using n-decane as internal standard. The slope showed a 2.3:1 (full power: half power) relation, which supports a mechanism involving more than one photon.

4.7 Catalyst deactivation

Scheme-S3

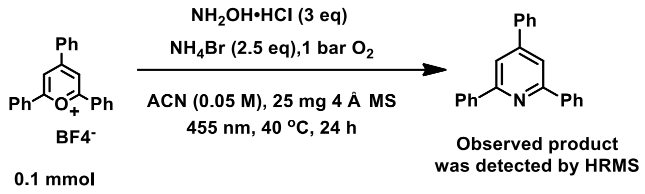

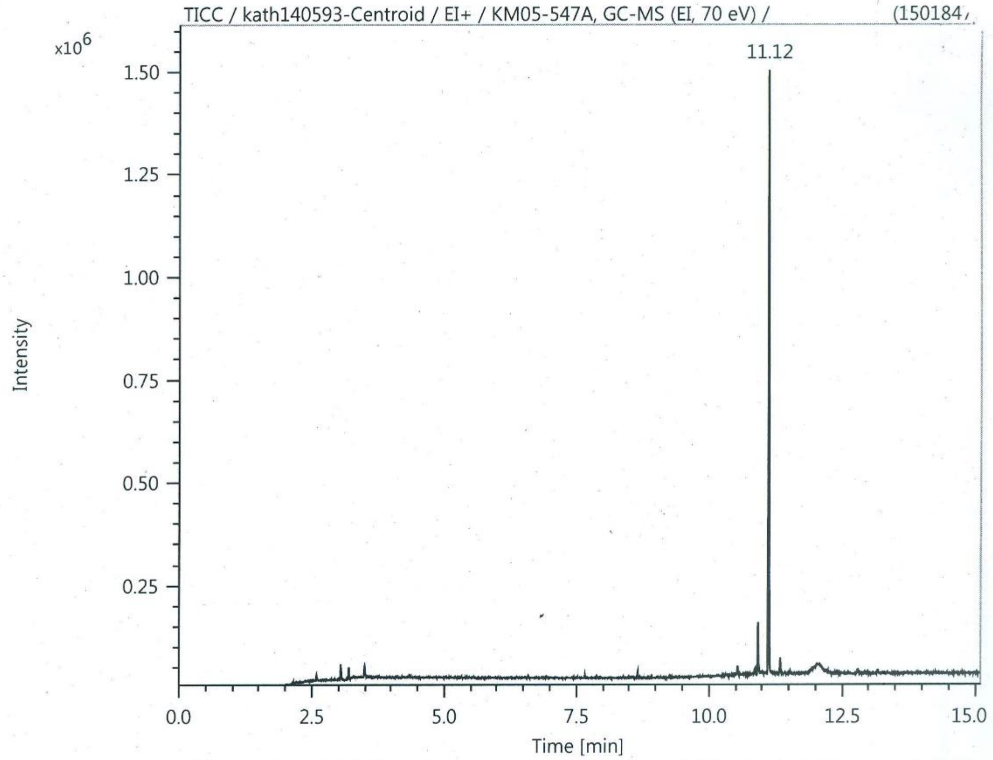
TICC / kath140593-Centroid / EI+ / KM05-547A, GC-MS (EI, 70 eV) / (150184
x10^6
Intensity
11.12
Time [min]

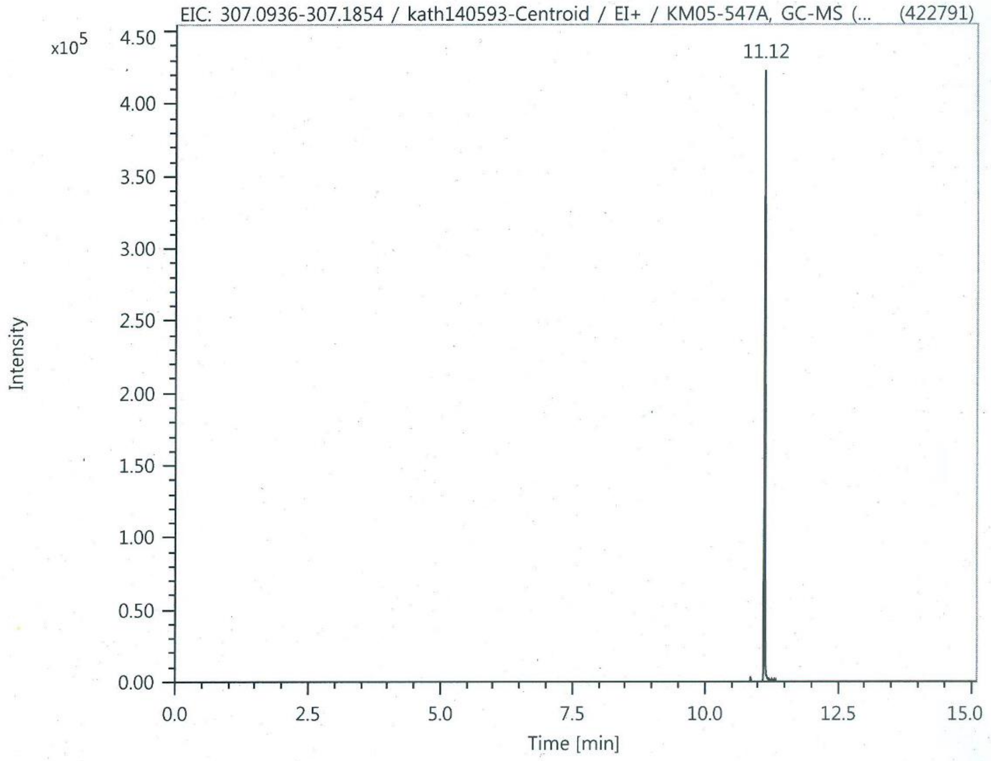
EIC: 307.0936-307.1854 / kath140593-Centroid / EI+ / KM05-547A, GC-MS (... (422791)
x10^5
Intensity
11.12
Time [min]

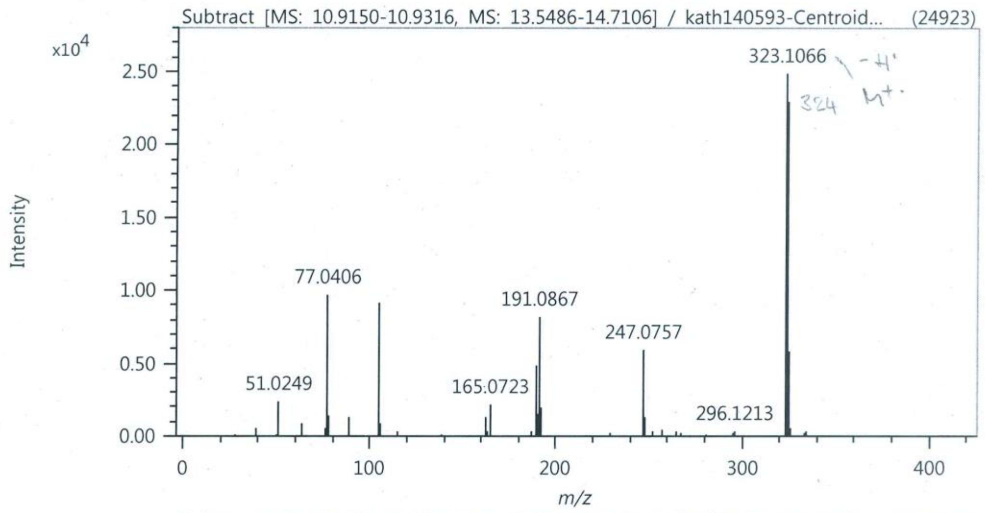
Subtract [MS: 10.9150-10.9316, MS: 13.5486-14.7106] / kath140593-Centroid... (24923)
x10^4
Intensity
m/z
323.1066
77.0406
191.0867
247.0757
51.0249
165.0723
296.1213
-H'
324 M+·

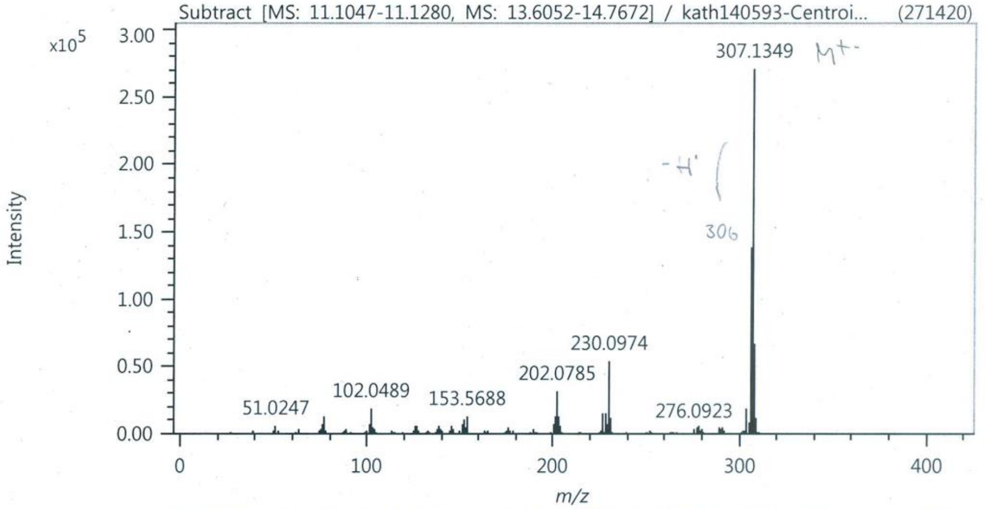
Subtract [MS: 11.1047-11.1280, MS: 13.6052-14.7672] / kath140593-Centroi... (271420)
x10^5
Intensity
m/z
307.1349 M+·
-H'
306
230.0974
202.0785
102.0489
153.5688
51.0247
276.0923

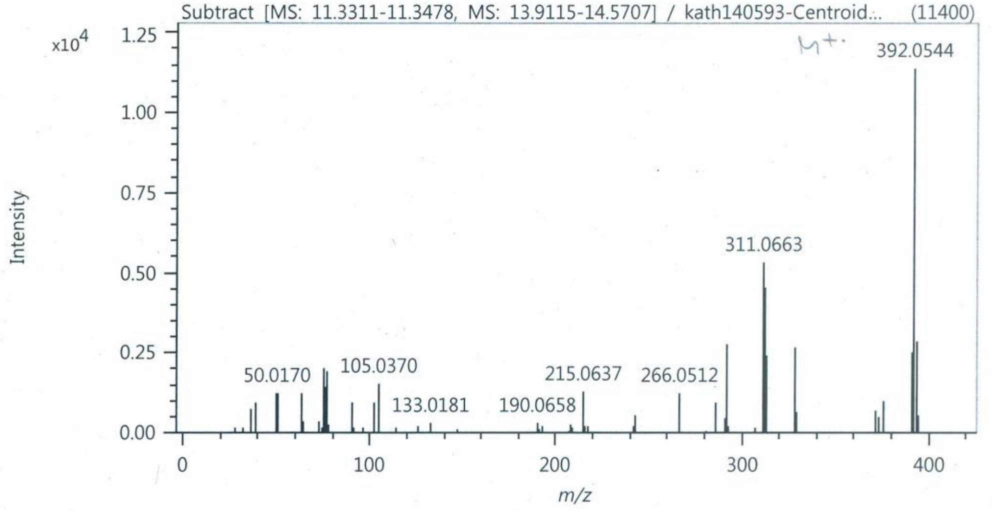
Subtract [MS: 11.3311-11.3478, MS: 13.9115-14.5707] / kath140593-Centroid... (11400)
x10^4
Intensity
m/z
M+·
392.0544
311.0663
50.0170
105.0370
215.0637
266.0512
133.0181
190.0658

Spectrum

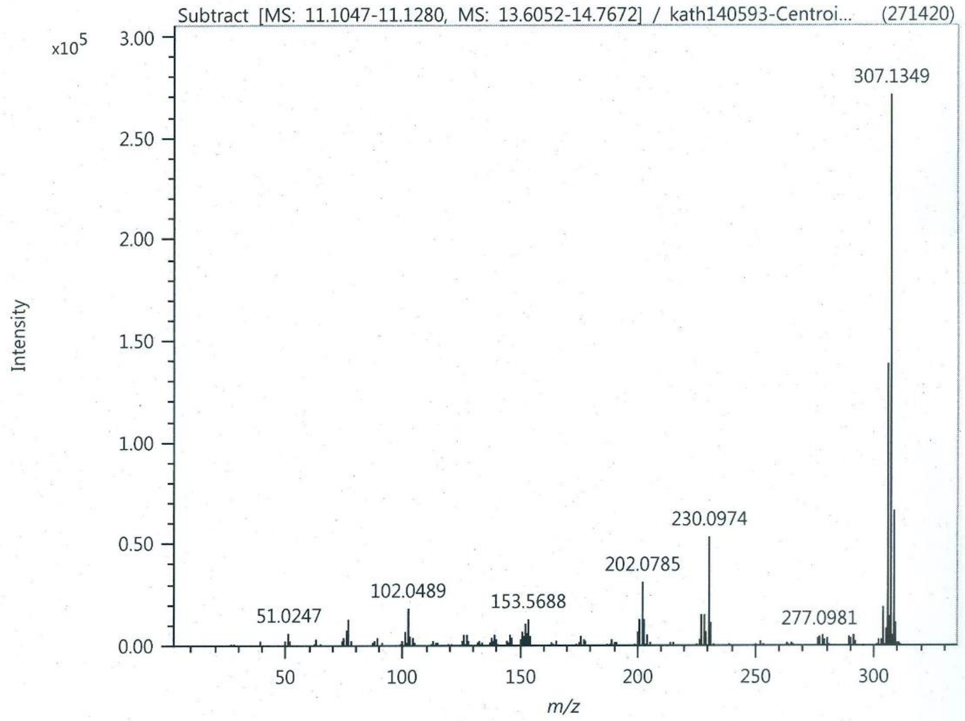

Elemental Composition

Parameters		Elements:								
Tolerance:	10.00 ppm	Symbol	C	H	N	O	Si	Cl	B	Bx
Electron:	Odd/Even	Min	1	1	0	0	0	0	0	0
Charge:	+1	Max	100	100	5	0	0	0	0	0
DBE:	-1.5 - 50.0									

Symbol	I
Min	0
Max	0

Results

Mass	Intensity	Formula	Calculated Mass	Mass Difference [mDa]	Mass Difference [ppm]	DBE
306.12766	138800.25	C23 H16 N	306.12773	-0.06	-0.20	16.5
307.13487	271419.63	C23 H17 N	307.13555	-0.68	-2.22	16.0

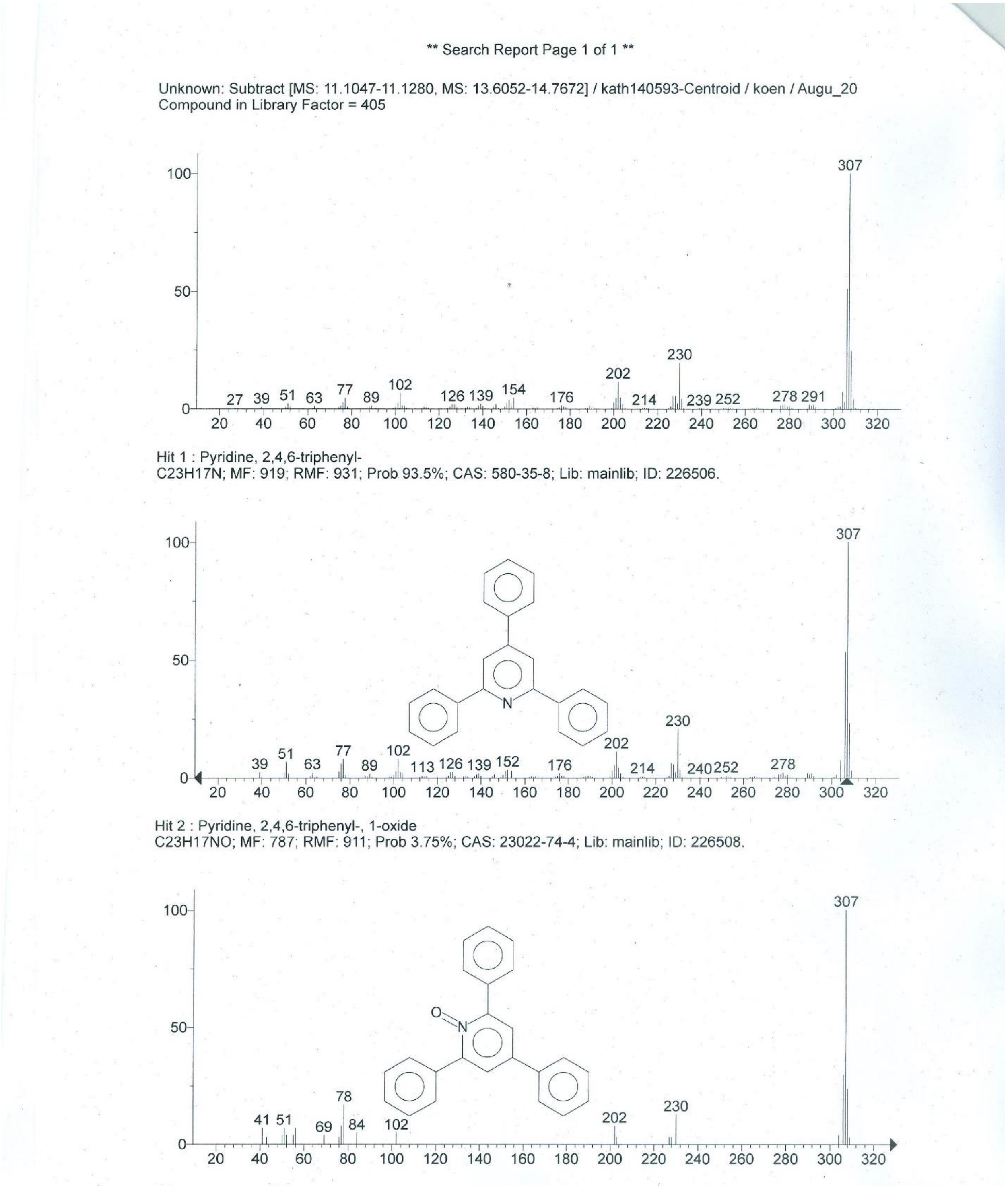

Fig. S20: HRMS and GC-MS spectra for catalyst deactivation

Reaction conditions: [a]0.1 mmol PC, 3 eq. NH_2OH•HCl, 2.5 eq. NH_4Br, 25 mg 4 Å MS, 1 bar O_2, 1 mL acetonitrile (0.05 M), 455 nm, 40 °C, 24 h.

4.8 Cyclic Voltammetry measurement

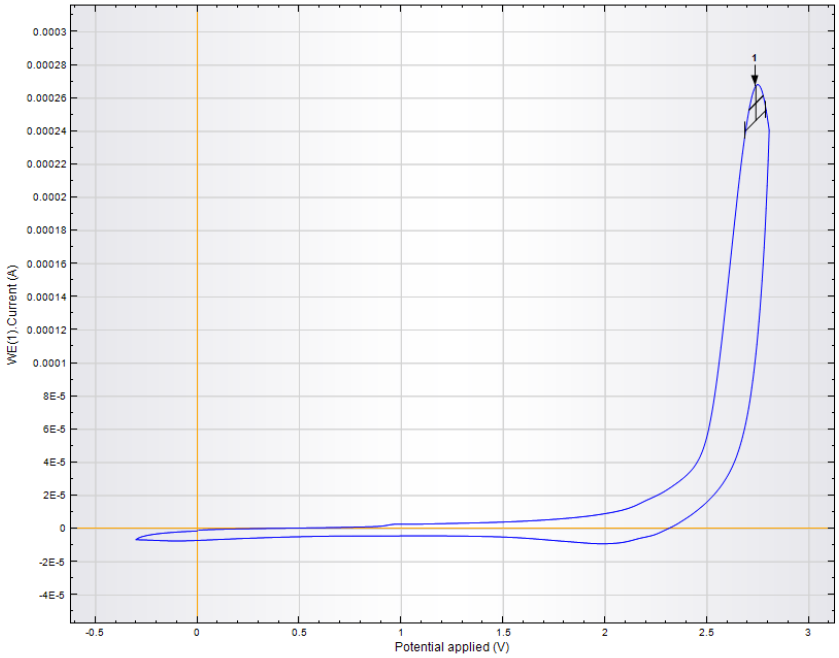

Index	Peak position
1	2.7393

Fig. S21: Cyclic voltammogram of methyl 4-methylbenzoate (**1**) in acetonitrile under argon. The peak at +2.74 V shows the oxidation of **1**. The measurement was performed with a scan rate of 50 mV/s and with TBATFB (0.1M) as supporting electrolyte.

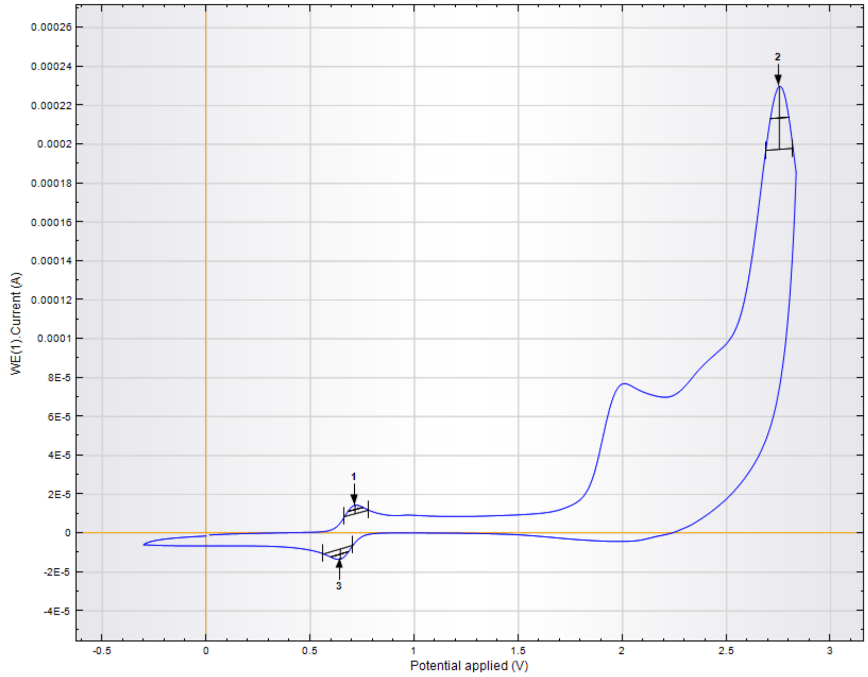

Index Peak position

Index	Peak position
1	0.71503
2	2.7544
3	0.64453

Fig. S22: Cyclic voltammogram of methyl 4-methylbenzoate (**1**) in the presence of ferrocene as internal standard in acetonitrile under argon. The peak at +2.75 V shows the oxidation of **1**, whereas the reversible peak at +0.64 and +0.72 V corresponds to ferrocene. The measurement was performed with a scan rate of 50 mV/s and with TBATFB (0.1M) as supporting electrolyte.

With these values, the oxidation potential of **1** was calculated as follows:

$$E_{1/2}(\mathbf{1}^{\bullet+}/\mathbf{1}) = \left(2.754\,V - \frac{0.715\,V + 0.645\,V}{2}\right)\,vs.\ Fc^{+}/Fc = 2.07\,V\ vs.Fc^{+}/Fc$$

This value was converted from the ferrocene reference to the SCE[6]:

$$E_{1/2}(\mathbf{1}^{\bullet+}/\mathbf{1}) = (2.07\,V + 0.38\,V)\ vs.SCE = 2.45\,V\ vs.SCE$$

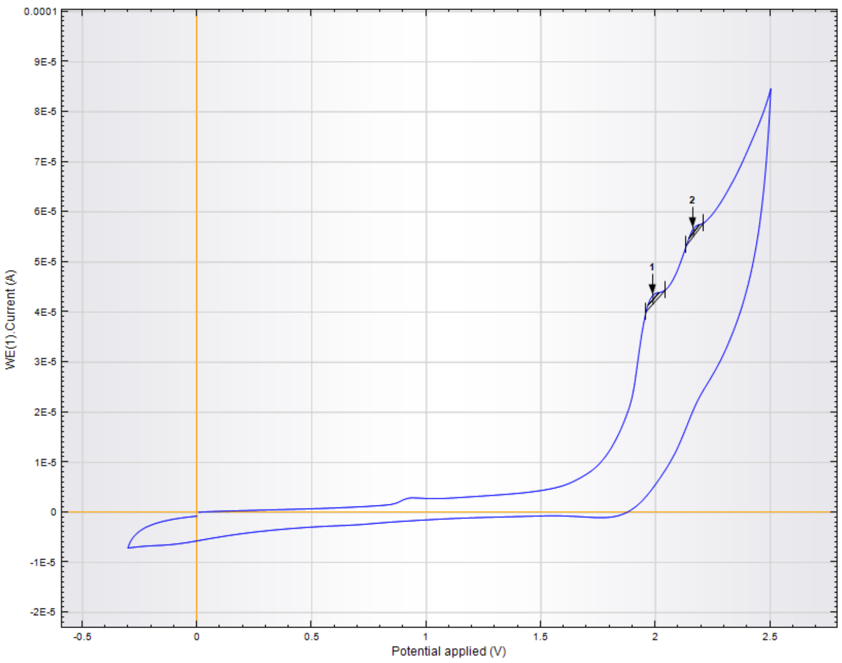

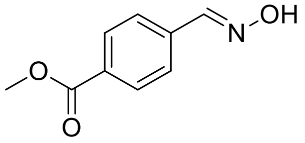

Index	Peak position
1	1.989
2	2.1652

Fig. S23: Cyclic voltammogram of (E)-methyl 4-((hydroxyimino)methyl)benzoate (**3**) in acetonitrile under argon. The peak at +1.99 V shows the oxidation of **3**. The measurement was performed with a scan rate of 50 mV/s and with TBATFB (0.1M) as supporting electrolyte.

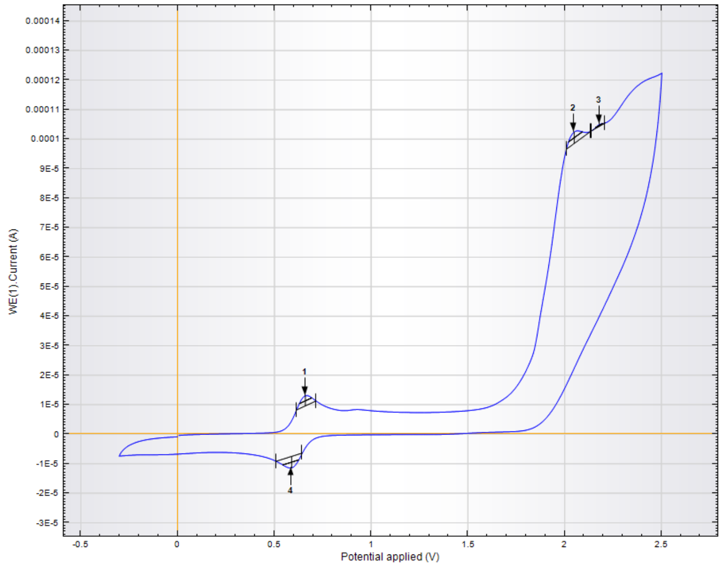

Index	Peak position
1	0.65964
2	2.0494
3	2.1803
4	0.58914

Fig. S24: Cyclic voltammogram of (E)-methyl 4-((hydroxyimino)methyl)benzoate (**3**) in the presence of ferrocene as internal standard in acetonitrile under argon. The peak at +2.05 V shows the oxidation of **3**, whereas the reversible peak at +0.59 and +0.66 V corresponds to ferrocene. The measurement was performed with a scan rate of 50 mV/s and with TBATFB (0.1M) as supporting electrolyte.

With these values, the oxidation potential of **3** was calculated as follows:

$$E_{1/2}(\mathbf{3}^{\bullet+}/\mathbf{3}) = \left(2.049\,V - \frac{0.656\,V + 0.589\,V}{2}\right)\,vs.\;Fc^{+}/Fc = 1.42\,V\,vs.Fc^{+}/Fc$$

This value was converted from the ferrocene reference to the SCE[6]:

$$E_{1/2}(\mathbf{3}^{\bullet+}/\mathbf{3}) = (1.42\,V + 0.38\,V)\,vs.SCE = 1.80\,V\,vs.SCE$$

5. Characterization of prepared compounds

(1H-indazol-1-yl)(p-tolyl)methanone[7]

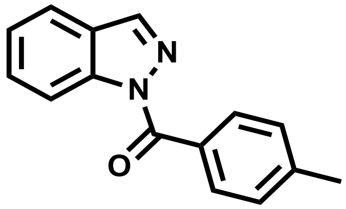

^{1}H NMR (400 MHz, Chloroform-*d*) δ 8.50 (dd, *J* = 8.4, 0.9 Hz, 1H), 8.12 (d, *J* = 0.8 Hz, 1H), 7.92 (d, *J* = 8.2 Hz, 2H), 7.69 (dd, *J* = 7.9, 1.0 Hz, 1H), 7.54 (ddd, *J* = 8.3, 7.1, 1.2 Hz, 1H), 7.32 (ddd, *J* = 8.0, 7.1, 0.9 Hz, 1H), 7.25 (d, *J* = 7.6 Hz, 2H), 2.38 (s, 3H). **^{13}C NMR (101 MHz, Chloroform-*d*)** δ 168.35 , 143.01 , 140.21 , 140.08 , 131.14 , 130.45 , 129.43 , 128.74 , 126.11 , 124.72 , 120.91 , 115.93 , 21.70 . **HRMS (EI)** (m/z): [M^+] ($C_{15}H_{12}N_2O^+$) calc. 236.0944; observed 236.0947. **Off-white solid.**

3-cyclopentylpropyl 4-methylbenzoate

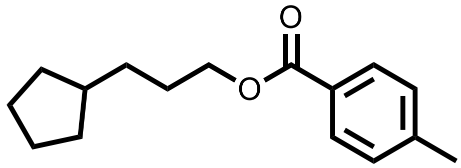

^{1}H NMR (400 MHz, Chloroform-*d*) δ 7.95 (d, *J* = 8.2 Hz, 2H), 7.25 (d, *J* = 8.0 Hz, 2H), 4.31 (t, *J* = 6.7 Hz, 2H), 2.42 (s, 3H), 1.87 – 1.72 (m, 5H), 1.68 – 1.40 (m, 6H), 1.18 – 1.02 (m, 2H). **^{13}C NMR (101 MHz, Chloroform-*d*)** δ 166.77 , 143.39 , 129.56 , 129.02 , 127.83 , 65.16 , 39.84 , 32.69 , 32.43 , 28.02 , 25.18 , 21.65 . **HRMS (APCI)** (m/z): [$M+H^+$] ($[C_{16}H_{22}O_2+H]^+$) calc. 247.1693; observed 247.1698. **Yellow oil.**

(1R,2S,5R)-2-isopropyl-5-methylcyclohexyl 4-methylbenzoate[8]

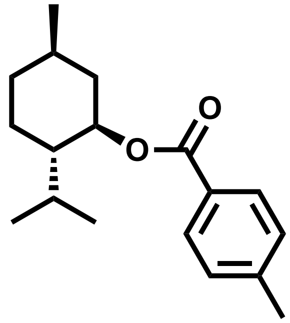

^{1}H NMR (400 MHz, Chloroform-*d*) δ 7.95 (d, *J* = 8.2 Hz, 2H), 7.25 (d, *J* = 7.9 Hz, 2H), 4.93 (td, *J* = 10.9, 4.4 Hz, 1H), 2.42 (s, 3H), 2.20 – 2.09 (m, 1H), 2.04 – 1.88 (m, 1H), 1.83 – 1.70 (m, 2H), 1.63 – 1.46 (m, 2H), 1.24 – 1.02 (m, 2H), 0.98 – 0.90 (m, 7H), 0.81 (d, *J* = 7.0 Hz, 3H). **^{13}C NMR (101 MHz, Chloroform-*d*)** δ 166.16 , 143.28 , 129.58 , 128.99 , 128.15 , 74.59 , 47.31 , 41.02 , 34.37 , 31.46 , 26.52 , 23.69 , 22.06 , 21.63 , 20.77 , 16.56 . **HRMS (APCI)** (m/z): [M+H^{+}] ([$C_{18}H_{26}O_2$+H]$^{+}$) calc. 275.2006; observed 275.2011. **Colorless oil.**

((3aR,5R,5aS,8aS,8bR)-2,2,7,7-tetramethyltetrahydro-3aH-bis([1,3]dioxolo)[4,5-b:4',5'-d]pyran-5-yl)methyl 4-methylbenzoate[9]

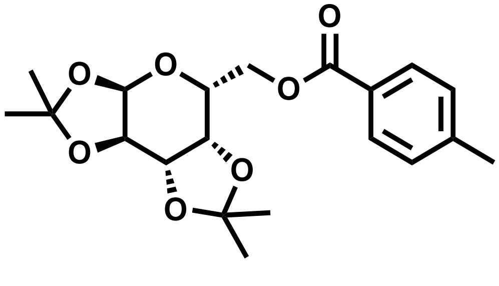

^{1}H NMR (400 MHz, Chloroform-*d*) δ 7.97 (d, *J* = 8.2 Hz, 2H), 7.25 (d, *J* = 8.0 Hz, 2H), 5.59 (d, *J* = 4.9 Hz, 1H), 4.68 (dd, *J* = 7.9, 2.5 Hz, 1H), 4.55 (dd, *J* = 11.5, 4.9 Hz, 1H), 4.47 – 4.33 (m, 3H), 4.25 – 4.16 (m, 1H), 2.43 (s, 3H), 1.54 (s, 3H), 1.50 (s, 3H), 1.38 (s, 3H), 1.36 (s, 3H). **^{13}C NMR (101 MHz, Chloroform-*d*)** δ 166.49 , 143.62 , 129.74 , 129.04 , 127.33 , 109.67 , 108.80 , 96.33 , 71.17 , 70.74 , 70.56 , 66.20 , 63.69 , 26.03 , 25.99 , 25.00 , 24.50 , 21.66 . **HRMS (ESI)** (m/z): [M+H^{+}] ([$C_{20}H_{26}O_7$+H]$^{+}$) calc. 379.1751; observed 379.1753. **Colorless gum.**

1,7,7-trimethylbicyclo[2.2.1]heptan-2-yl 4-methylbenzoate

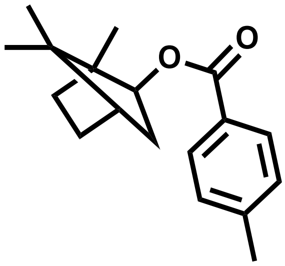

^{1}H NMR (400 MHz, Chloroform-*d*) δ 7.92 (d, *J* = 8.2 Hz, 2H), 7.25 (d, *J* = 7.8 Hz, 2H), 4.92 (dd, *J* = 7.1, 4.5 Hz, 1H), 2.42 (s, 3H), 1.99 – 1.69 (m, 4H), 1.68 – 1.57 (m, 1H), 1.30 – 1.18 (m, 2H), 1.14 (s, 3H), 0.95 (s, 3H), 0.91 (s, 3H). **^{13}C NMR (101 MHz, Chloroform-*d*)** δ 166.13 , 143.30 , 129.49 , 129.05 , 128.18 , 81.33 , 49.01 , 47.03 , 45.14 , 38.95 , 33.78 , 27.10 , 21.64 , 20.16 , 20.09 , 11.60 . **HRMS (APCI)** (m/z): [M+H$^+$] ([$C_{18}H_{24}O_2$+H]$^+$) calc. 273.1849; observed 273.1852. **Colorless oil.**

2,2,3,3,4,4,5,5,6,6,7,7,8,8,9,9,10,10,11,11,12,12,12-tricosafluorododecyl 4-methylbenzoate

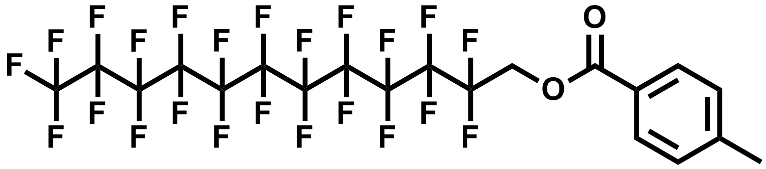

^{1}H NMR (400 MHz, Chloroform-*d*) δ 7.95 (d, *J* = 8.2 Hz, 2H), 7.27 (d, *J* = 8.3 Hz, 2H), 4.92 – 4.68 (m, 2H), 2.43 (s, 3H). **^{13}C NMR (101 MHz, Chloroform-*d*)** δ 164.96 , 144.84 , 130.03 , 129.35 , 125.57 , 59.96 , 21.74 . **^{19}F NMR (282 MHz, Chloroform-*d*)** δ -81.31 (t, *J* = 10.0 Hz, 3F), -119.58 – -120.10 (m, 2F), -121.95 – -122.72 (m, 12F), -123.02 – -123.47 (m, 2F), -123.49 – -124.17 (m, 2F), -126.40 – -127.02 (m, 2F). **HRMS (APCI)** (m/z): [M+NH$_4^+$] ([$C_{20}H_9F_{23}O_2$+NH$_4$]$^+$) calc. 736.0574; observed 736.0589. **White solid.**

(S)-dimethyl 2-((4-methylbenzoyl)oxy)succinate

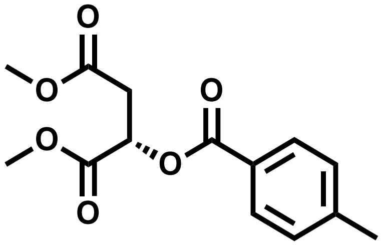

^{1}H NMR (400 MHz, Chloroform-*d*) δ 7.95 (d, *J* = 8.2 Hz, 2H), 7.25 (d, *J* = 8.0 Hz, 2H), 5.72 (dd, *J* = 6.9, 5.4 Hz, 1H), 3.79 (s, 3H), 3.73 (s, 3H), 3.04 (dd, *J* = 6.2, 1.8 Hz, 2H), 2.42 (s, 3H). **^{13}C NMR (101 MHz, Chloroform-*d*)** δ 169.62 , 169.50 , 165.51 , 144.30 , 129.99 , 129.15 , 126.31 , 68.60 , 52.73 , 52.20 , 36.21 , 21.72 . **HRMS (APCI)** (m/z): [M+H^{+}] ([$C_{14}H_{16}O_6$+H]$^{+}$) calc. 281.1020; observed 281.1023. **Yellow oil.**

(S)-methyl 3-methyl-2-(4-methylbenzamido)butanoate[10]

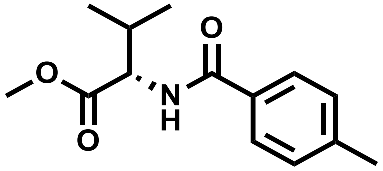

^{1}H NMR (400 MHz, Chloroform-*d*) δ 7.71 (d, *J* = 8.2 Hz, 2H), 7.25 (d, *J* = 8.0 Hz, 2H), 6.59 (d, *J* = 8.6 Hz, 1H), 4.79 (dd, *J* = 8.7, 4.9 Hz, 1H), 3.78 (s, 3H), 2.41 (s, 3H), 2.35 – 2.22 (m, 1H), 1.00 (dd, *J* = 9.2, 6.9 Hz, 6H). **^{13}C NMR (101 MHz, Chloroform-*d*)** δ 172.75 , 167.18 , 142.19 , 131.32 , 129.26 , 127.05 , 57.34 , 52.22 , 31.68 , 21.47 , 19.00 , 17.98 . **HRMS (ESI)** (m/z): [M+H^{+}] ([$C_{14}H_{19}NO_3$+H]$^{+}$) calc. 250.1438; observed 250.1440. **White solid.**

(3S,5S,8R,9S,10S,13R,14S,17R)-10,13-dimethyl-17-((R)-6-methylheptan-2-yl)hexadecahydro-1H-cyclopenta[a]phenanthren-3-yl 4-methylbenzoate[11]

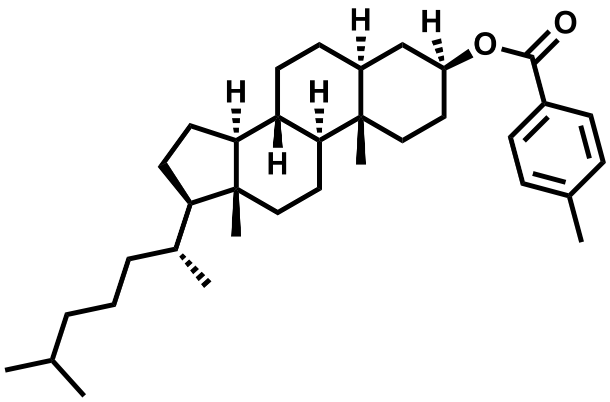

^{1}H NMR (400 MHz, Chloroform-*d*) δ 7.95 (d, *J* = 8.2 Hz, 2H), 7.25 (d, *J* = 8.0 Hz, 2H), 5.29 – 4.47 (m, 1H), 2.43 (s, 3H), 2.07 – 0.84 (m, 43H), 0.78 – 0.61 (m, 3H). **^{13}C NMR (101 MHz, Chloroform-*d*)** δ 166.21 , 143.22 , 129.54 , 128.94 , 128.24 , 74.15 , 56.44 , 56.29 , 54.26 , 44.73 , 42.61 , 40.01 , 39.53 , 36.83 , 36.18 , 35.82 , 35.54 , 35.52 , 34.17 , 32.03 , 28.67 , 28.02 , 27.62 , 26.23, 24.23 , 23.85 , 22.83 , 22.57 , 21.64 , 21.24 , 18.69 , 12.31 , 12.09 . **HRMS (APCI)** (m/z): [M+H^{+}] ([$C_{35}H_{54}O_2$+H]$^{+}$) calc. 507.4197; observed 507.4210. **White solid.**

(2R,3S,4S,5R,6S)-6-(acetoxymethyl)-3-(4-methylbenzamido)tetrahydro-2H-pyran-2,4,5-triyl triacetate[12]

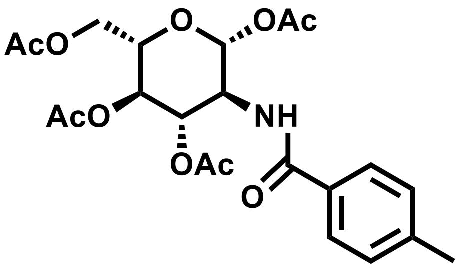

^{1}H NMR (400 MHz, Chloroform-*d*) δ 7.58 (d, *J* = 8.2 Hz, 2H), 7.19 (d, *J* = 8.0 Hz, 2H), 6.33 (d, *J* = 9.5 Hz, 1H), 5.79 (d, *J* = 8.8 Hz, 1H), 5.37 – 5.15 (m, 2H), 4.57 (dt, *J* = 10.4, 9.1 Hz, 1H), 4.29 (dd, *J* = 12.5, 4.7 Hz, 1H), 4.16 (dd, *J* = 12.5, 2.2 Hz, 1H), 3.86 (ddd, *J* = 9.8, 4.7, 2.2 Hz, 1H), 2.37 (s, 3H), 2.10 (s, 3H), 2.06 (s, 6H), 1.98 (s, 3H). **^{13}C NMR (101 MHz, Chloroform-*d*)** δ 171.54 , 170.69 , 169.62 , 169.25 , 167.21 , 142.55 , 130.69 , 129.39 , 126.93 , 92.88 , 73.15 , 72.73 , 67.77 , 61.76 , 53.22 , 21.45 , 20.86 , 20.75 , 20.63 , 20.60 . **HRMS (ESI)** (m/z): [M+H$^+$] ([$C_{22}H_{27}NO_{10}$+H]$^+$) calc. 466.1708; observed 466.1706. **White solid.**

(R)-methyl 4-methyl-2-((S)-3-methyl-2-(4-methylbenzamido)butanamido)pentanoate

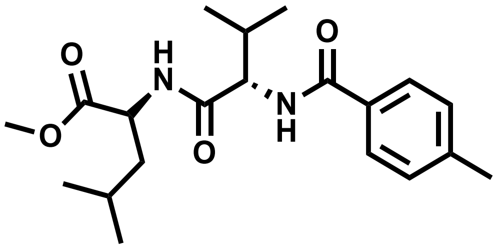

^{1}H NMR (400 MHz, Chloroform-*d*) δ 7.70 (d, *J* = 8.2 Hz, 2H), 7.22 (d, *J* = 7.4 Hz, 2H), 6.88 (d, *J* = 8.7 Hz, 1H), 6.81 (d, *J* = 7.8 Hz, 1H), 4.68 – 4.51 (m, 2H), 3.73 (s, 3H), 2.38 (s, 3H), 2.29 – 2.12 (m, 1H), 1.69 – 1.42 (m, 3H), 1.03 (dd, *J* = 8.2, 6.8 Hz, 6H), 0.84 (dd, *J* = 6.2, 4.9 Hz, 6H). **^{13}C NMR (101 MHz, Chloroform-*d*)** δ 173.11 , 171.34 , 167.31 , 142.20 , 131.23 , 129.23 , 127.12 , 58.49 , 52.24 , 50.92 , 41.07 , 31.75 , 24.80 , 22.65 , 21.81 , 21.47 , 19.15 , 18.37 . **HRMS (ESI)** (m/z): [M+H$^+$] ([$C_{20}H_{30}N_2O_4$+H]$^+$) calc. 363.2278; observed 363.2285. **White solid.**

(E)-methyl 4-((hydroxyimino)methyl)benzoate[4]

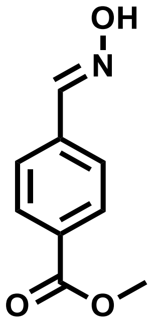

^{1}H NMR (400 MHz, Chloroform-*d*) δ 7.86 (s, 1H), 7.27 (d, *J* = 8.7 Hz, 2H), 6.66 (d, *J* = 8.7 Hz, 2H), 3.58 (s, 3H). **^{13}C NMR (101 MHz, Chloroform-*d*)** δ 161.10 , 149.92 , 128.56 , 124.56 , 114.27 , 113.96 , 55.36 . **White solid.** (Exchangeable OH-proton was not picked-up)

(E)-4-((hydroxyimino)methyl)benzoic acid[13]

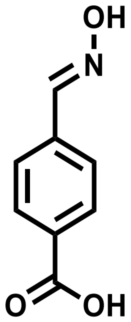

^{1}H NMR (400 MHz, DMSO-*d*$_6$) δ 13.03 (br s, 1H), 11.53 (s, 1H), 8.21 (s, 1H), 7.94 (d, *J* = 8.3 Hz, 2H), 7.69 (d, *J* = 8.3 Hz, 2H). **^{13}C NMR (101 MHz, DMSO-*d*$_6$)** δ 167.38 , 147.99 , 137.65 , 131.56 , 130.15 , 126.88 . **White solid.**

(E)-4-bromobenzaldehyde oxime[14]

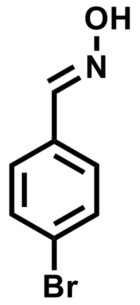

^{1}H NMR (400 MHz, DMSO-*d*$_6$) δ 11.35 (s, 1H), 8.12 (s, 1H), 7.59 (d, *J* = 8.6 Hz, 2H), 7.53 (d, *J* = 8.6 Hz, 2H). **^{13}C NMR (101 MHz, DMSO-*d*$_6$)** δ 147.68 , 132.82 , 132.16 , 128.75 , 122.86 . **White solid.**

(E)-4-((hydroxyimino)methyl)benzonitrile[15]

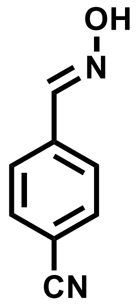

^{1}H NMR (400 MHz, DMSO-*d*$_6$) δ 11.72 (s, 1H), 8.23 (s, 1H), 7.85 (d, *J* = 8.4 Hz, 2H), 7.76 (d, *J* = 8.4 Hz, 2H). **^{13}C NMR (101 MHz, DMSO-*d*$_6$)** δ 147.57 , 138.08 , 133.14 , 127.47 , 119.16 , 111.83 . **White solid.**

methyl 2-cyanobenzoate[16]

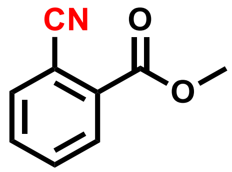

^{1}H NMR (400 MHz, Chloroform-*d*) δ 8.18 – 8.09 (m, 1H), 7.83 – 7.77 (m, 1H), 7.70 – 7.63 (m, 2H), 3.99 (s, 3H). **^{13}C NMR (101 MHz, Chloroform-*d*)** δ 164.51 , 134.82 , 132.72 , 132.50 , 132.42 , 131.19 , 117.53 , 112.95 , 52.87 . **Yellow solid.**

methyl 3-cyanobenzoate[17]

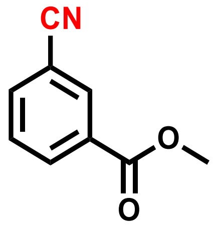

^{1}H NMR (400 MHz, Chloroform-*d*) δ 8.32 (td, *J* = 1.7, 0.6 Hz, 1H), 8.26 (dt, *J* = 8.0, 1.5 Hz, 1H), 7.83 (dt, *J* = 7.8, 1.4 Hz, 1H), 7.58 (td, *J* = 7.9, 0.7 Hz, 1H), 3.95 (s, 3H). **^{13}C NMR (101 MHz, Chloroform-*d*)** δ 165.09 , 135.98 , 133.65 , 133.27 , 131.44 , 129.46 , 117.88 , 112.99 , 52.72 . **White solid.**

methyl 4-cyanobenzoate[18]

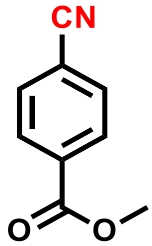

^{1}H NMR (400 MHz, Chloroform-*d*) δ 8.13 (d, *J* = 8.5 Hz, 2H), 7.74 (d, *J* = 8.4 Hz, 2H), 3.95 (s, 3H). **^{13}C NMR (101 MHz, Chloroform-*d*)** δ 165.43 , 133.94 , 132.24 , 130.11 , 117.97 , 116.42 , 52.74 . **White solid.**

4-methoxybenzonitrile[19]

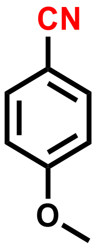

^{1}H NMR (400 MHz, Chloroform-*d*) δ 7.58 (d, *J* = 8.9 Hz, 2H), 6.95 (d, *J* = 8.8 Hz, 2H), 3.86 (s, 3H). **^{13}C NMR (101 MHz, Chloroform-*d*)** δ 162.86 , 134.00 , 119.24 , 114.77 , 104.00 , 55.57 . **White solid.**

3-cyanobenzoic acid[20]

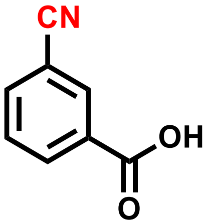

^{1}H NMR (400 MHz, DMSO-*d*$_6$) δ 8.27 (d, *J* = 1.7 Hz, 1H), 8.22 (dt, *J* = 7.9, 1.5 Hz, 1H), 8.08 (dt, *J* = 7.8, 1.5 Hz, 1H), 7.78 – 7.66 (m, 1H). **^{13}C NMR (101 MHz, DMSO-*d*$_6$)** δ 166.15 , 136.72 , 134.22 , 133.22 , 132.51 , 130.55 , 118.53 , 112.38 . **White solid.** (Exchangeable OH-proton was not picked-up)

4-cyanobenzoic acid[21]

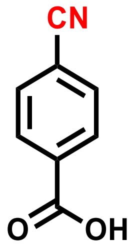

^{1}H NMR (400 MHz, DMSO-*d*$_6$) δ 13.55 (br s, 1H), 8.08 (d, *J* = 8.5 Hz, 2H), 7.98 (d, *J* = 8.4 Hz, 2H). **^{13}C NMR (101 MHz, DMSO-*d*$_6$)** δ 166.48 , 135.32 , 133.12 , 130.37 , 118.64 , 115.51 . **Off-white solid.**

4-cyanobenzamide[22]

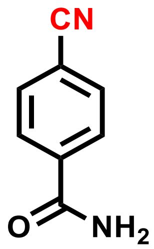

^{1}H NMR (400 MHz, DMSO-*d*$_6$) δ 8.19 (br s, 1H), 8.01 (d, *J* = 8.7 Hz, 2H), 7.93 (d, *J* = 8.4 Hz, 2H), 7.66 (br s, 1H). **^{13}C NMR (101 MHz, DMSO-*d*$_6$)** δ 166.90 , 138.75 , 132.84 , 128.71 , 118.83 , 114.10 . **White solid.**

ethyl 4-cyanobenzoate[23]

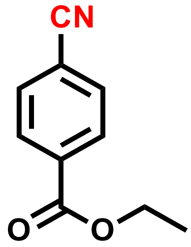

^{1}H NMR (400 MHz, Chloroform-*d*) δ 8.14 (d, *J* = 8.7 Hz, 2H), 7.74 (d, *J* = 8.7 Hz, 2H), 4.42 (q, *J* = 7.1 Hz, 2H), 1.41 (t, *J* = 7.1 Hz, 3H). **^{13}C NMR (101 MHz, Chloroform-*d*)** δ 164.96 , 134.32 , 132.19 , 130.08 , 118.03 , 116.30 , 61.84 , 14.26 . **White solid.**

4-(tert-butyl)benzonitrile[23]

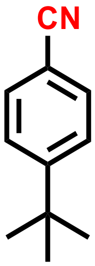

^{1}H NMR (400 MHz, Chloroform-*d*) δ 7.58 (d, *J* = 8.7 Hz, 2H), 7.48 (d, *J* = 8.5 Hz, 2H), 1.33 (s, 9H). **^{13}C NMR (101 MHz, Chloroform-*d*)** δ 156.66 , 131.98 , 126.19 , 119.17 , 109.33 , 35.29 , 30.97 . **Yellow oil.**

4-(4,4,5,5-tetramethyl-1,3,2-dioxaborolan-2-yl)benzonitrile[24]

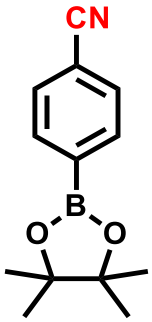

^{1}H NMR (400 MHz, Chloroform-*d*) δ 7.88 (d, *J* = 8.2 Hz, 2H), 7.64 (d, *J* = 8.3 Hz, 2H), 1.35 (s, 12H). **^{13}C NMR (101 MHz, Chloroform-*d*)** δ 135.11 , 135.10 , 131.15 , 118.88 , 114.56 , 84.52 , 24.89 . **White solid.**

1-naphthonitrile[25]

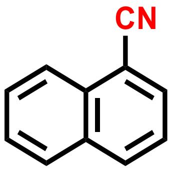

^{1}H NMR (400 MHz, Chloroform-*d*) δ 8.28 – 8.19 (m, 1H), 8.08 (dd, *J* = 8.4, 1.1 Hz, 1H), 7.91 (ddd, *J* = 7.1, 6.3, 1.1 Hz, 2H), 7.69 (ddd, *J* = 8.3, 6.9, 1.4 Hz, 1H), 7.62 (ddd, *J* = 8.2, 6.9, 1.3 Hz, 1H), 7.52 (dd, *J* = 8.3, 7.2 Hz, 1H). **^{13}C NMR (101 MHz, Chloroform-*d*)** δ 133.31 , 132.95 , 132.65 , 132.38 , 128.69 , 128.62 , 127.58 , 125.17 , 124.95 , 117.85 , 110.22 . **Off-white solid.**

4-chlorobenzonitrile[25]

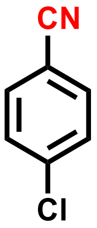

^{1}H NMR (400 MHz, Chloroform-*d*) δ 7.60 (d, *J* = 8.6 Hz, 2H), 7.46 (d, *J* = 8.6 Hz, 2H). **^{13}C NMR (101 MHz, Chloroform-*d*)** δ 139.57 , 133.40 , 129.72 , 117.98 , 110.82 . **White solid.**

4-iodobenzonitrile[26]

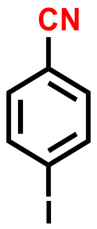

^{1}H NMR (400 MHz, Chloroform-*d*) δ 7.85 (d, *J* = 8.4 Hz, 2H), 7.37 (d, *J* = 8.4 Hz, 2H). **^{13}C NMR (101 MHz, Chloroform-*d*)** δ 138.54 , 133.18 , 118.23 , 111.78 , 100.32. **Pale brown solid.**

4-(trifluoromethoxy)benzonitrile[23]

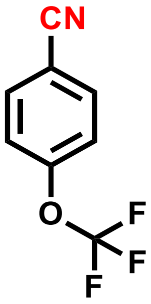

^{1}H NMR (400 MHz, Chloroform-*d*) δ 7.72 (d, *J* = 8.9 Hz, 2H), 7.32 (d, *J* = 8.0 Hz, 2H). **^{13}C NMR (101 MHz, Chloroform-*d*)** δ 152.21 (d, *J* = 1.9 Hz), 134.19 ,120.18 (q, *J* = 259.7 Hz), 121.23 , 117.65 , 110.85 . **^{19}F NMR (376 MHz, Chloroform-*d*)** δ -58.30 . **Yellow oil.**

methyl 4-cyano-2-methoxybenzoate[27]

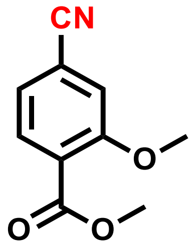

^{1}H NMR (400 MHz, Chloroform-*d*) δ 7.68 – 7.58 (m, 3H), 3.98 (s, 3H), 3.93 (s, 3H). **^{13}C NMR (101 MHz, Chloroform-*d*)** δ 165.50 , 161.12 , 135.49 , 133.74 , 121.65 , 115.61 , 112.03 , 105.84 , 56.36 , 52.78 . **HRMS (EI)** (m/z): [M$^+$] ($C_{10}H_9NO_3^+$) calc. 191.0576; observed 191.0578. **Yellow solid**.

[1,1'-biphenyl]-4-carbonitrile[26]

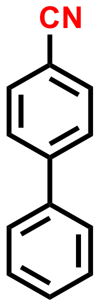

^{1}H NMR (400 MHz, Chloroform-*d*) δ 7.76 – 7.66 (m, 4H), 7.62 – 7.56 (m, 2H), 7.53 – 7.39 (m, 3H). **^{13}C NMR (101 MHz, Chloroform-*d*)** δ 145.70 , 139.20 , 132.62 , 129.14 , 128.68 , 127.76 , 127.25 , 118.96 , 110.95 . **White solid.**

[1,1'-biphenyl]-3-carbonitrile[26]

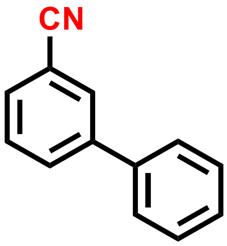

^{1}H NMR (400 MHz, Chloroform-*d*) δ 7.90 – 7.84 (m, 1H), 7.82 (dt, *J* = 7.8, 1.6 Hz, 1H), 7.64 (dt, *J* = 7.7, 1.4 Hz, 1H), 7.59 – 7.52 (m, 3H), 7.52 – 7.38 (m, 3H). **^{13}C NMR (101 MHz, Chloroform-*d*)** δ 142.48 , 138.91 , 131.51 , 130.74 , 130.71 , 129.62 , 129.15 , 128.41 , 127.11 , 118.87 , 112.99 . **Yellow gum.**

[1,1'-biphenyl]-2,4'-dicarbonitrile[28]

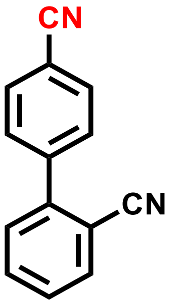

^{1}H NMR (400 MHz, Chloroform-*d*) δ 7.59 – 7.52 (m, 3H), 7.50 – 7.40 (m, 3H), 7.34 – 7.24 (m, 2H). **^{13}C NMR (101 MHz, Chloroform-*d*)** δ 143.33 , 142.53 , 133.99 , 133.20 , 132.54 , 129.93 , 129.59 , 128.80 , 118.40 , 118.03 , 112.66 , 111.25 . **HRMS (EI)** (m/z): [M$^+$] ($C_{14}H_8N_2^+$) calc. 204.0682; observed 204.0682. **Off-white solid.**

methyl 4'-cyano-[1,1'-biphenyl]-2-carboxylate[29]

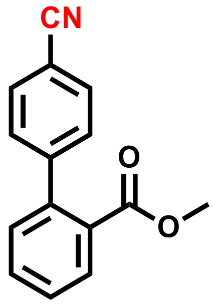

^{1}H NMR (400 MHz, Chloroform-*d*) δ 7.78 (dd, *J* = 7.7, 1.4 Hz, 1H), 7.54 (d, *J* = 8.3 Hz, 2H), 7.47 – 7.38 (m, 1H), 7.38 – 7.29 (m, 1H), 7.25 (d, *J* = 8.3 Hz, 2H), 7.16 (dd, *J* = 7.7, 1.3 Hz, 1H), 3.52 (s, 3H). **^{13}C NMR (101 MHz, Chloroform-*d*)** δ 167.93 , 146.39 , 141.09 , 131.79 , 131.77 , 130.52 , 130.45 , 130.07 , 129.19 , 128.29 , 118.89 , 111.10 , 52.11 . **HRMS (EI)** (m/z): [M$^+$] ($C_{15}H_{11}NO_2^+$) calc. 237.0784; observed 237.0786. **Colorless gum.**

[1,1'-biphenyl]-4,4'-dicarbonitrile[23]

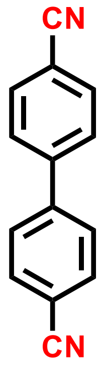

^{1}H NMR (400 MHz, Chloroform-*d*) δ 7.78 (d, *J* = 8.6 Hz, 4H), 7.69 (d, *J* = 8.6 Hz, 4H). **^{13}C NMR (101 MHz, Chloroform-*d*)** δ 146.76 , 132.91 , 127.95 , 118.41 , 112.48 . **HRMS (EI)** (m/z): [M$^+$] ($C_{14}H_8N_2^+$) calc. 204.0682; observed 204.0684. **White solid.**

4-(1H-indazole-1-carbonyl)benzonitrile[30]

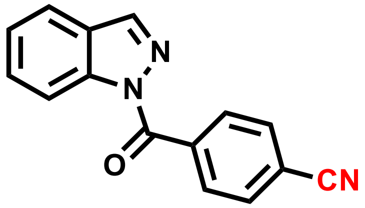

^{1}H NMR (400 MHz, Chloroform-*d*) δ 8.57 (dt, *J* = 8.4, 0.9 Hz, 1H), 8.22 (s, 1H), 8.16 (d, *J* = 8.4 Hz, 2H), 7.87 – 7.76 (m, 3H), 7.66 (ddd, *J* = 8.4, 7.2, 1.1 Hz, 1H), 7.46 (ddd, *J* = 8.0, 7.1, 0.9 Hz, 1H). **^{13}C NMR (101 MHz, Chloroform-*d*)** δ 166.57 , 141.22 , 139.96 , 137.36 , 131.74 , 131.38 , 130.03 , 126.28 , 125.45 , 121.21 , 118.06 , 115.93 , 115.53 . **HRMS (EI)** (m/z): [M^+] ($C_{15}H_9N_3O^+$) calc. 247.0740; observed 247.0749. **White solid.**

benzo[b]thiophene-3-carbonitrile[23]

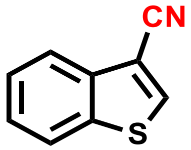

^{1}H NMR (400 MHz, Chloroform-*d*) δ 7.89 (s, 1H), 7.81 – 7.74 (m, 1H), 7.72 – 7.65 (m, 1H), 7.38 – 7.20 (m, 2H). **^{13}C NMR (101 MHz, Chloroform-*d*)** δ 138.52 , 137.54 , 137.30 , 126.21 , 126.01 , 122.86 , 122.58 , 114.34 , 107.17 . **HRMS (EI)** (m/z): [M^+] ($C_9H_5NS^+$) calc. 159.0137; observed 159.0133. **Brown solid.**

3-cyclopentylpropyl 4-cyanobenzoate

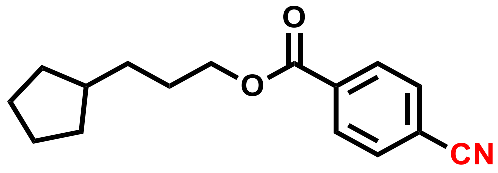

^{1}H NMR (400 MHz, Chloroform-*d*) δ 8.13 (d, *J* = 8.4 Hz, 2H), 7.74 (d, *J* = 8.4 Hz, 2H), 4.34 (t, *J* = 6.7 Hz, 2H), 1.86 – 1.70 (m, 5H), 1.66 – 1.36 (m, 6H), 1.17 – 1.03 (m, 2H). **^{13}C NMR (101 MHz, Chloroform-*d*)** δ 164.99 , 134.34 , 132.19 , 130.05 , 118.02 , 116.27 , 66.18 , 39.78 , 32.67 , 32.34 , 27.89 , 25.16 . **HRMS (APCI)** (m/z): [$M+NH_4^+$] ($C_{16}H_{19}NO_2+NH_4^+$) calc. 275.1754; observed 275.1760. **Colorless oil.**

(1R,2S,5R)-2-isopropyl-5-methylcyclohexyl 4-cyanobenzoate[31]

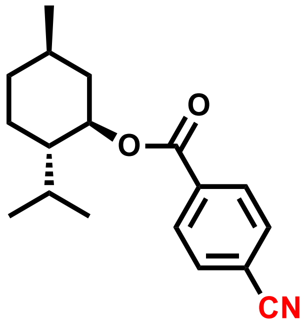

^{1}H NMR (400 MHz, Chloroform-*d*) δ 8.13 (d, *J* = 8.4 Hz, 2H), 7.73 (d, *J* = 8.4 Hz, 2H), 4.95 (td, *J* = 10.9, 4.4 Hz, 1H), 2.18 – 2.03 (m, 1H), 1.96 – 1.84 (m, 1H), 1.78 – 1.67 (m, 2H), 1.63 – 1.49 (m, 2H), 1.19 – 1.02 (m, 2H), 0.98 – 0.87 (m, 7H), 0.78 (d, *J* = 7.0 Hz, 3H). **^{13}C NMR (101 MHz, Chloroform-*d*)** δ 164.42 , 134.65 , 132.16 , 130.06 , 118.05 , 116.16 , 75.94 , 47.20 , 40.85 , 34.20 , 31.45 , 26.57 , 23.59 , 22.00 , 20.73 , 16.49 . **HRMS (APCI)** (m/z): [$M+NH_4^+$] ($[C_{18}H_{23}NO_2+NH_4]^+$) calc. 303.2067; observed 303.2068. **Yellow gum.**

((3aR,5R,5aS,8aS,8bR)-2,2,7,7-tetramethyltetrahydro-3aH-bis([1,3]dioxolo)[4,5-b:4',5'-d]pyran-5-yl)methyl 4-cyanobenzoate[32]

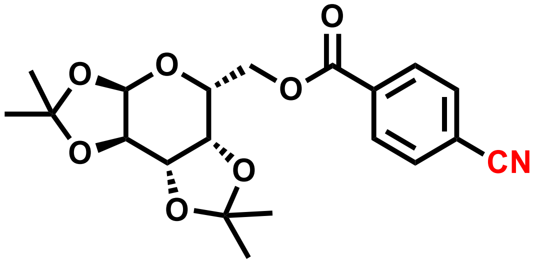

^{1}H NMR (400 MHz, Chloroform-*d*) δ 8.13 (d, *J* = 8.5 Hz, 2H), 7.72 (d, *J* = 8.5 Hz, 2H), 5.55 (d, *J* = 4.9 Hz, 1H), 4.64 (dd, *J* = 7.9, 2.5 Hz, 1H), 4.53 (dd, *J* = 11.6, 4.5 Hz, 1H), 4.45 (dd, *J* = 11.6, 7.8 Hz, 1H), 4.34 (dd, *J* = 5.0, 2.5 Hz, 1H), 4.30 (dd, *J* = 7.9, 1.9 Hz, 1H), 4.22 – 4.11 (m, 1H), 1.47 (d, *J* = 13.4 Hz, 6H), 1.33 (d, *J* = 7.6 Hz, 6H). **^{13}C NMR (101 MHz, Chloroform-*d*)** δ 164.84 , 133.89 , 132.23 , 130.20 , 117.98 , 116.46 , 109.82 , 108.85 , 96.32 , 71.10 , 70.76 , 70.46 , 66.06 , 64.74 , 26.02 , 25.98 , 24.95 , 24.50 . **HRMS (ESI)** (m/z): [M+H$^+$] ([$C_{20}H_{23}NO_7$+H]$^+$) calc. 390.1547; observed 390.1559. **Yellow gum.**

1,7,7-trimethylbicyclo[2.2.1]heptan-2-yl 4-cyanobenzoate

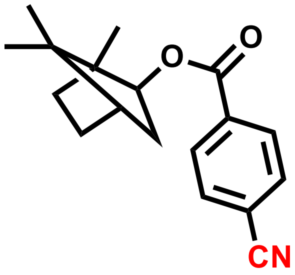

^{1}H NMR (400 MHz, Chloroform-*d*) δ 8.09 (d, *J* = 8.4 Hz, 2H), 7.73 (d, *J* = 8.5 Hz, 2H), 4.93 (dd, *J* = 7.4, 4.2 Hz, 1H), 1.97 – 1.85 (m, 2H), 1.85 – 1.70 (m, 2H), 1.67 – 1.55 (m, 1H), 1.28 – 1.12 (m, 2H), 1.10 (s, 3H), 0.91 (s, 3H), 0.89 (s, 3H). **^{13}C NMR (101 MHz, Chloroform-*d*)** δ 164.37 , 134.68 , 132.24 , 129.94 , 118.03 , 116.18 , 82.60 , 49.13 , 47.06 , 45.07 , 38.82 , 33.69 , 27.01 , 20.08 , 20.06 , 11.59 . **HRMS (APCI)** (m/z): [M+NH$_4^+$] ([$C_{18}H_{21}NO_2$+NH_4]$^+$) calc. 301.1911; observed 301.1918. **White solid.**

2,2,3,3,4,4,5,5,6,6,7,7,8,8,9,9,10,10,11,11,12,12,12-tricosafluorododecyl 4-cyanobenzoate

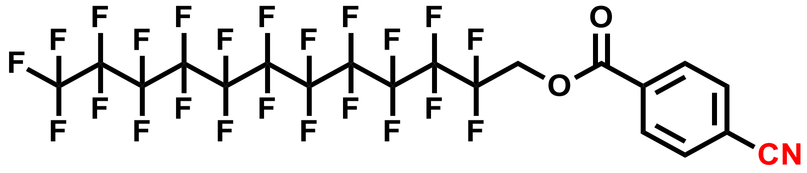

^{1}H NMR (400 MHz, Chloroform-*d*) δ 8.17 (d, *J* = 8.5 Hz, 2H), 7.79 (d, *J* = 8.4 Hz, 2H), 4.85 (t, *J* = 13.1 Hz, 2H).**^{13}C NMR (101 MHz, Chloroform-*d*)** δ 163.35 , 132.45 , 132.03 , 130.44 , 117.61 , 117.45 , 60.56 (t, *J* = 27.8 Hz). **^{19}F NMR (282 MHz, Chloroform-*d*)** δ -81.35 (t, *J* = 10.0 Hz, 3F), -119.41 – -120.36 (m, 2F), -121.97 – -122.76 (m, 12F), -123.19 – -123.37 (m, 2F), -123.58 – -123.80 (m, 2F), -126.26 – -126.97 (m, 2F). **HRMS (APCI)** (m/z): [M+NH_4^+] ([$C_{20}H_6F_{23}NO_2$+NH_4]$^+$) calc. 747.0370; observed 747.0391. **White solid.**

(S)-dimethyl 2-((4-cyanobenzoyl)oxy)succinate

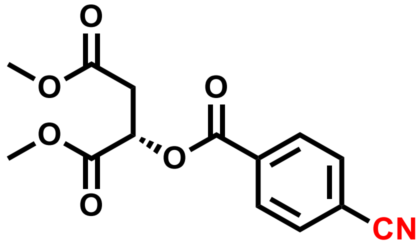

^{1}H NMR (400 MHz, Chloroform-*d*) δ 8.13 (d, *J* = 8.6 Hz, 2H), 7.74 (d, *J* = 8.7 Hz, 2H), 5.72 (dd, *J* = 6.8, 5.3 Hz, 1H), 3.78 (s, 3H), 3.71 (s, 3H), 3.07 – 3.00 (m, 2H). **^{13}C NMR (101 MHz, Chloroform-*d*)** δ 169.39 , 168.82 , 163.90 , 132.89 , 132.29 , 130.42 , 117.83 , 116.89 , 69.27 , 52.93 , 52.31 , 35.92 . **HRMS (APCI)** (m/z): [M+NH_4^+] ([$C_{14}H_{13}NO_6$+NH_4]$^+$) calc. 309.1081; observed 309.1089. **Yellow gum.**

(S)-methyl 2-(4-cyanobenzamido)-3-methylbutanoate

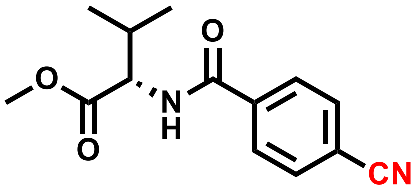

^{1}H NMR (400 MHz, Chloroform-*d*) δ 7.89 (d, *J* = 8.4 Hz, 2H), 7.73 (d, *J* = 8.6 Hz, 2H), 6.74 (d, *J* = 8.6 Hz, 1H), 4.75 (dd, *J* = 8.6, 4.9 Hz, 1H), 3.78 (s, 3H), 2.44 – 2.08 (m, 1H), 0.99 (dd, *J* = 7.9, 6.9 Hz, 6H). **^{13}C NMR (101 MHz, Chloroform-*d*)** δ 172.43 , 165.60 , 138.02 , 132.49 , 127.83 , 117.96 , 115.33 , 57.71 , 52.46 , 31.59 , 19.00 , 18.00 . **HRMS (ESI)** (m/z): [M+H^{+}] ([$C_{14}H_{16}N_2O_3$+H]$^{+}$) calc. 261.1234; observed 261.1238. **Off-white solid.**

4-cyano-N-(perfluorophenyl)benzamide[33]

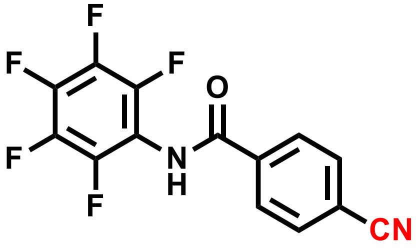

^{1}H NMR (400 MHz, DMSO-*d*$_6$) δ 10.85 (s, 1H), 8.15 (d, *J* = 8.4 Hz, 2H), 8.07 (d, *J* = 8.4 Hz, 2H). **^{13}C NMR (101 MHz, DMSO-*d*$_6$)** δ 164.71 , 153.11 – 151.07 (m), 145.22 – 143.67 (m), 139.64 – 138.22 (m), 136.69 , 134.29 – 132.51 (m), 130.10 – 128.57 (m), 118.57 , 115.30 , 113.35 – 112.62 (m). ^{19}F NMR (376 MHz, DMSO-*d*$_6$) δ -144.66 (d, *J* = 21.0 Hz, 2F), -156.39 (t, *J* = 22.9 Hz, 1F), -159.09 – -168.13 (m, 2F). **HRMS (EI)** (m/z): [M^{+}] ($C_{14}H_5N_2F_5O^+$) calc. 312.0316; observed 312.0308. **Colorless gum.**

(3S,5S,8R,9S,10S,13R,14S,17R)-10,13-dimethyl-17-((R)-6-methylheptan-2-yl)hexadecahydro-1H-cyclopenta[a]phenanthren-3-yl 4-cyanobenzoate

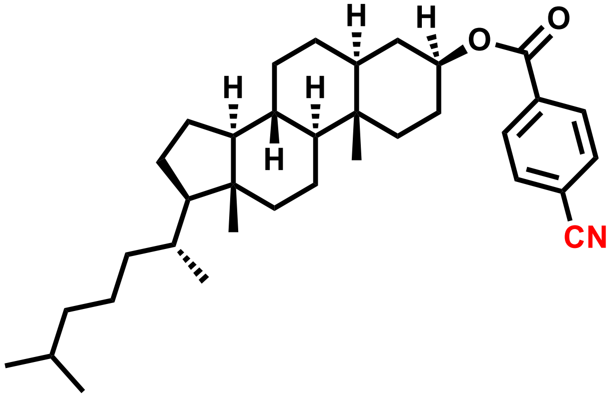

^{1}H NMR (400 MHz, Chloroform-*d*) δ 8.12 (d, *J* = 8.4 Hz, 2H), 7.72 (d, *J* = 8.4 Hz, 2H), 5.04 – 4.86 (m, 1H), 2.03 – 0.81 (m, 42H), 0.75 – 0.55 (m, 4H). **^{13}C NMR (101 MHz, Chloroform-*d*)** δ 164.43 , 134.78 , 132.12 , 130.06 , 118.08 , 116.14 , 75.47 , 56.43 , 56.30 , 54.24 , 44.71 , 42.62 , 40.00 , 39.54 , 36.77 , 36.20 , 35.83 , 35.53 , 35.51 , 34.05 , 32.00 , 28.65 , 28.27 , 28.04 , 27.53 , 24.24 , 23.87 , 22.85 , 22.59 , 21.26 , 18.71 , 12.31 , 12.11 . **HRMS (APCI)** (m/z): [$M+NH_4^+$] ([$C_{35}H_{51}NO_2+NH_4$]$^+$) calc. 535.4258; observed 535.4255. **White solid.**

4-(5-(4-cyanophenyl)-3-(trifluoromethyl)-1H-pyrazol-1-yl)benzenesulfonamide[34]

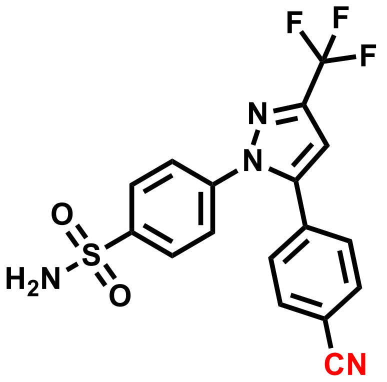

^{1}H NMR (300 MHz, Chloroform-*d*) δ 7.96 (d, *J* = 8.8 Hz, 2H), 7.69 (d, *J* = 8.7 Hz, 2H), 7.46 (d, *J* = 8.7 Hz, 2H), 7.37 (d, *J* = 8.6 Hz, 2H), 6.87 (s, 1H), 4.95 (s, 2H). **^{13}C NMR (101 MHz, Chloroform-*d*)** δ 144.56 (q, *J* = 39.0 Hz), 143.00 , 142.20 , 141.85 , 132.94 , 132.87 , 129.41 , 127.90 , 125.64 , 120.74 (q, *J* = 269.6 Hz), 117.82 , 113.47 , 107.51 . **^{19}F NMR (376 MHz, Chloroform-*d*)** δ -63.03 . **HRMS (ESI)** (m/z): [M+H$^+$] ([$C_{17}H_{11}F_3N_4O_2S$+H]$^+$) calc. 393.0628; observed 393.0636. **Off-white solid.**

(2R,3S,4S,5R,6S)-6-(acetoxymethyl)-3-(4-cyanobenzamido)tetrahydro-2H-pyran-2,4,5-triyl triacetate

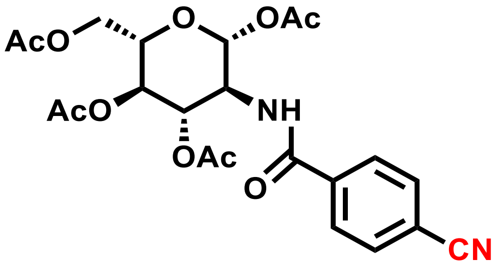

^{1}H NMR (400 MHz, Chloroform-*d*) δ 7.79 (d, *J* = 8.6 Hz, 2H), 7.71 (d, *J* = 8.6 Hz, 2H), 6.54 (d, *J* = 9.4 Hz, 1H), 5.81 (d, *J* = 8.7 Hz, 1H), 5.36 – 5.15 (m, 2H), 4.53 (dt, *J* = 10.4, 9.1 Hz, 1H), 4.29 (dd, *J* = 12.5, 4.7 Hz, 1H), 4.20 – 4.06 (m, 1H), 3.91 – 3.82 (m, 1H), 2.10 (s, 3H), 2.07 (s, 3H), 2.06 (s, 3H), 2.00 (s, 3H). **^{13}C NMR (101 MHz, Chloroform-*d*)** δ 171.63 , 170.69 , 169.58 , 169.25 , 165.57 , 137.34 , 132.62 , 127.66 , 117.78 , 115.64 , 92.72 , 73.14 , 72.67 , 67.63 , 61.69

, 53.79 , 20.86 , 20.74 , 20.65 , 20.60. **HRMS (ESI)** (m/z): [M+H$^+$] ([$C_{22}H_{24}N_2O_{10}$+H]$^+$) calc. 477.1504; observed 477.1505. **Brown solid.**

(R)-methyl 2-((S)-2-(4-cyanobenzamido)-3-methylbutanamido)-4-methylpentanoate

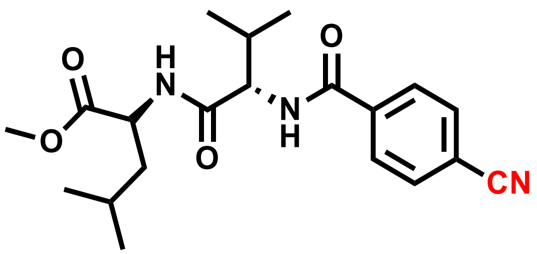

^{1}H NMR (400 MHz, Chloroform-*d*) δ 7.82 (d, *J* = 8.4 Hz, 2H), 7.60 (d, *J* = 8.4 Hz, 2H), 7.27 (d, *J* = 8.7 Hz, 1H), 6.86 (d, *J* = 7.8 Hz, 1H), 4.57 – 4.41 (m, 2H), 3.63 (s, 3H), 2.16 – 2.03 (m, 1H), 1.58 – 1.35 (m, 3H), 0.91 (dd, *J* = 9.7, 6.7 Hz, 6H), 0.74 (d, *J* = 6.1 Hz, 6H). **^{13}C NMR (101 MHz, Chloroform-*d*)** δ 173.00 , 171.20 , 165.65 , 137.85 , 132.38 , 127.96 , 117.94 , 115.29 , 58.94 , 52.32 , 51.02 , 41.00 , 31.74 , 24.81 , 22.64 , 21.81 , 19.08 , 18.48 . **HRMS (ESI)** (m/z): [M+H$^+$] ([$C_{20}H_{27}N_3O_4$+H]$^+$) calc. 374.2074; observed 374.2078. **Brown solid**

4-(pentafluorothio)benzonitrile[35]

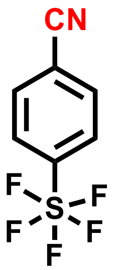

^{1}H NMR (400 MHz, Chloroform-*d*) δ 7.89 (d, *J* = 8.8 Hz, 2H), 7.79 (d, *J* = 9.6 Hz, 2H). **^{13}C NMR (101 MHz, Chloroform-*d*)** δ 157.62 – 155.08 (m), 132.72 , 128.44 – 125.04 (m), 116.90 , 115.87 . **White solid**

terephthalonitrile[25]

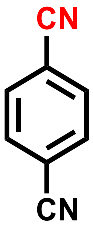

^{1}H NMR (400 MHz, Chloroform-*d*) δ 7.80 (s, 4H). **^{13}C NMR (101 MHz, Chloroform-*d*)** δ 132.80 , 117.01 , 116.73 . **White solid**

isophthalonitrile[25]

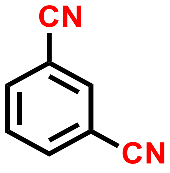

^{1}H NMR (400 MHz, Chloroform-*d*) δ 7.99 – 7.93 (m, 1H), 7.91 (d, *J* = 1.6 Hz, 1H), 7.89 (d, *J* = 1.6 Hz, 1H), 7.70 – 7.61 (m, 1H). **^{13}C NMR (101 MHz, Chloroform-*d*)** δ 136.00 , 135.43 , 130.35 , 116.60 , 114.19 . **White solid.**

3,5-dibromobenzonitrile[36]

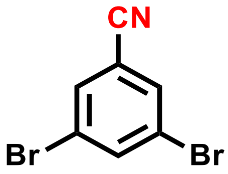

^{1}H NMR (400 MHz, Chloroform-*d*) δ 7.91 (t, *J* = 1.8 Hz, 1H), 7.74 (d, *J* = 1.7 Hz, 2H). **^{13}C NMR (101 MHz, Chloroform-*d*)** δ 138.84 , 133.45 , 123.60 , 115.92 , 115.47 . **White solid**.

2,6-dichlorobenzonitrile[37]

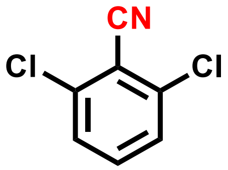

^{1}H NMR (400 MHz, Chloroform-*d*) δ 7.96 – 6.74 (m, 3H). **^{13}C NMR (101 MHz, Chloroform-*d*)** δ 138.51 , 133.86 , 128.17 , 114.44 , 113.35 . **White solid**.

4-nitrobenzonitrile[25]

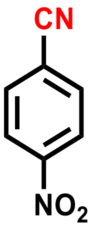

^{1}H NMR (400 MHz, Chloroform-*d*) δ 8.36 (d, *J* = 8.8 Hz, 2H), 7.89 (d, *J* = 8.8 Hz, 2H). ^{13}C NMR (101 MHz, Chloroform-*d*) δ 150.04 , 133.48 , 124.30 , 118.35 , 116.79 . **Yellow solid.**

6. NMR Spectra

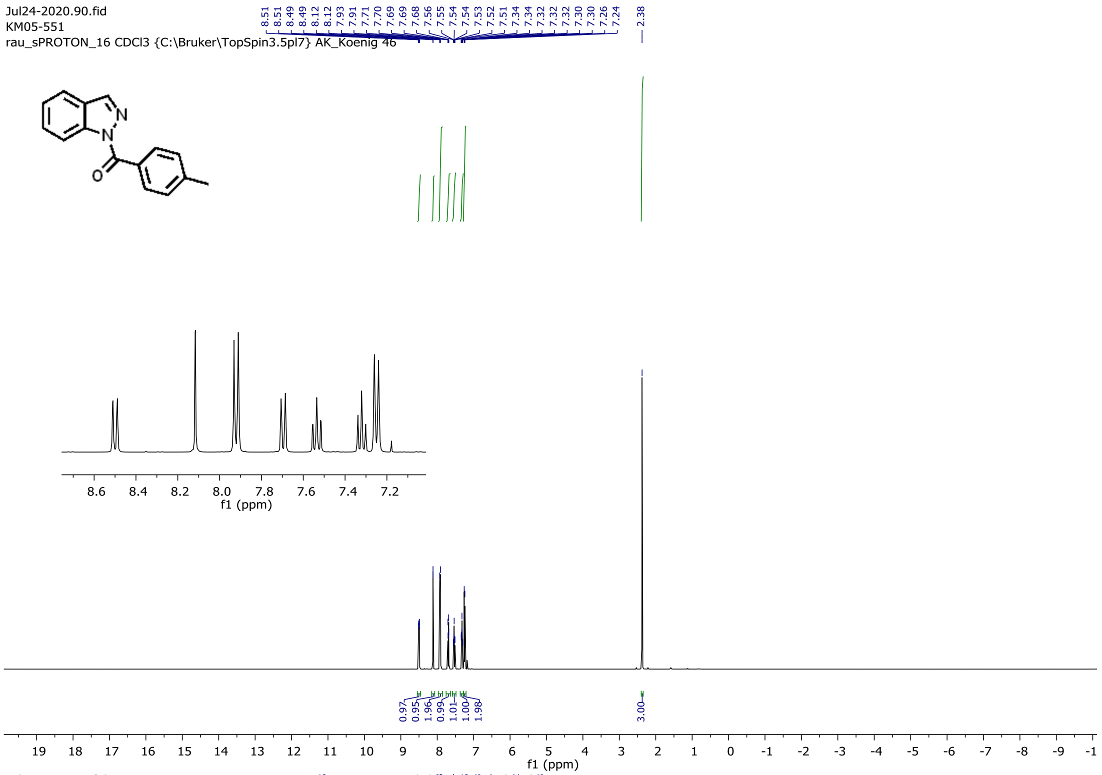

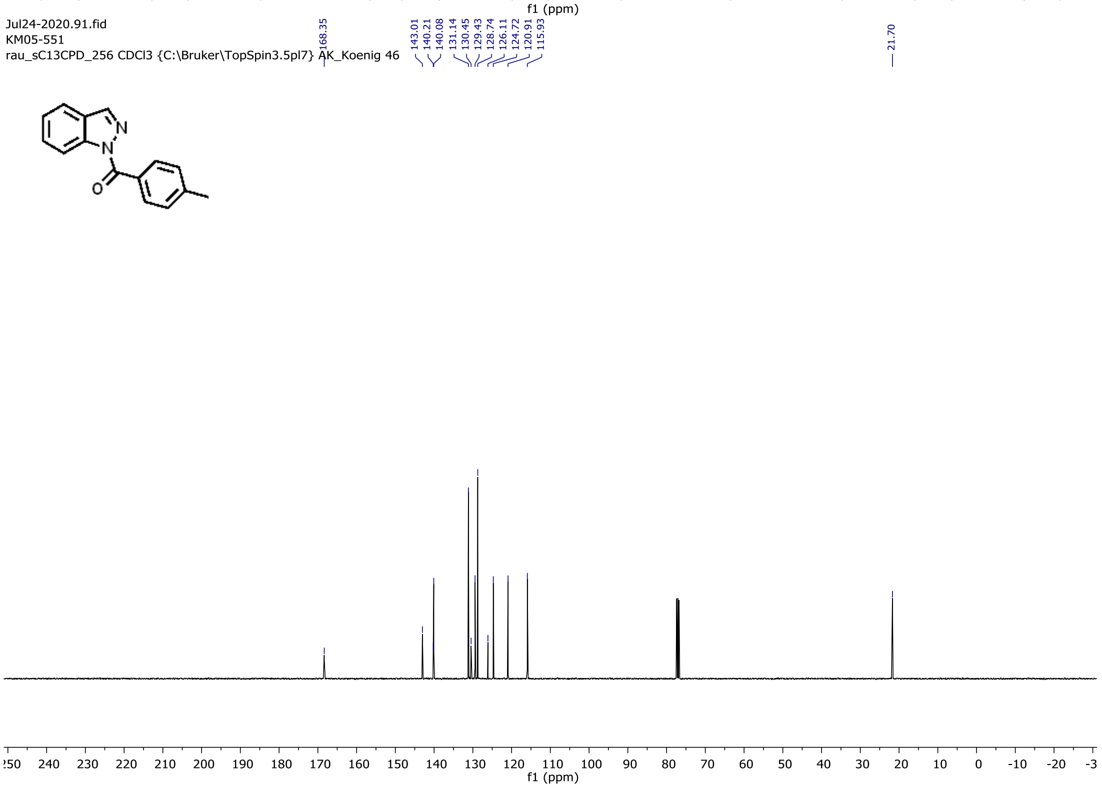

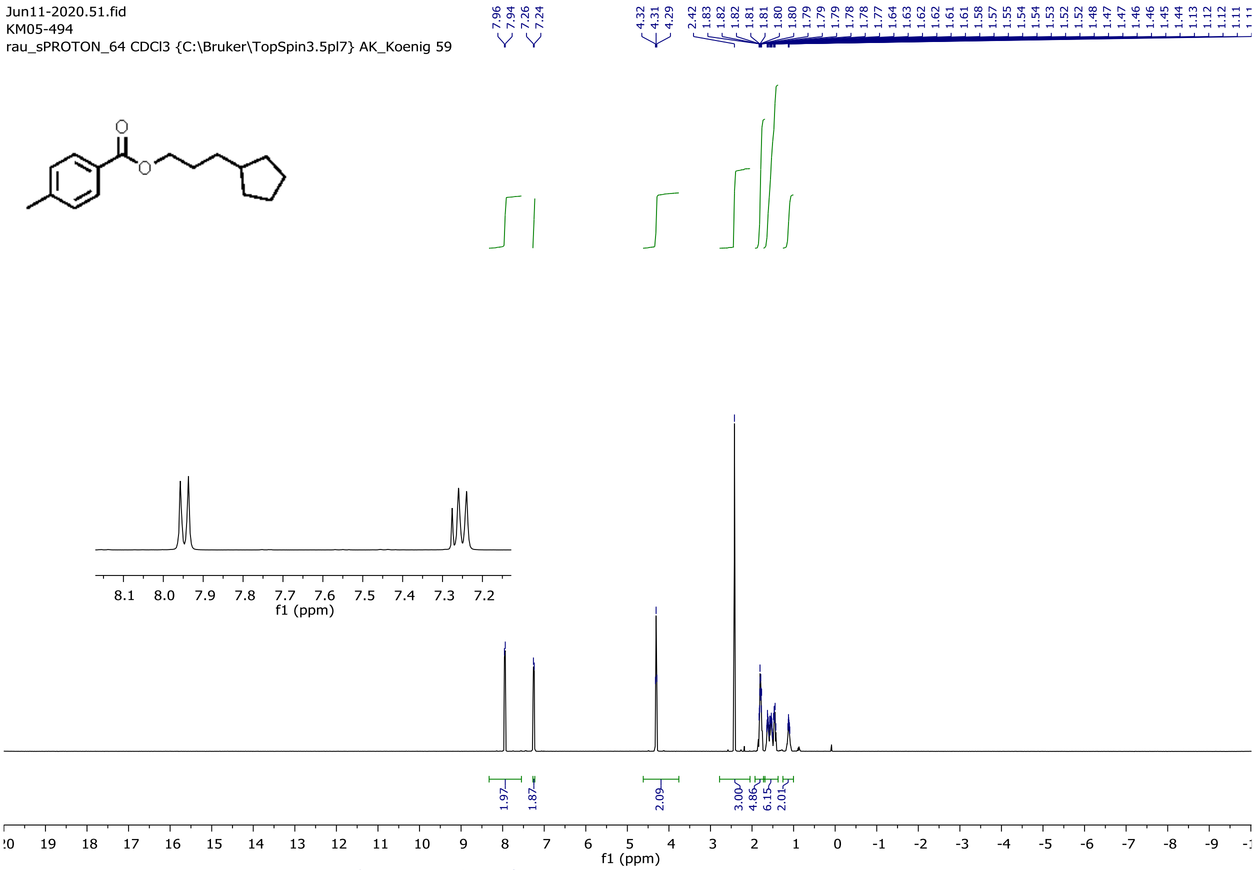
Jun11-2020.51.fid
KM05-494
rau_sPROTON_64 CDCl3 {C:\Bruker\TopSpin3.5pl7} AK_Koenig 59
7.96 7.94 7.26 7.24
4.32 4.31 4.29
2.42 1.83 1.82 1.82 1.81 1.81 1.80 1.80 1.79 1.79 1.78 1.77 1.64 1.63 1.62 1.62 1.61 1.61 1.58 1.57 1.55 1.54 1.54 1.53 1.52 1.52 1.48 1.47 1.46 1.46 1.45 1.44 1.13 1.12 1.12 1.11
8.1 8.0 7.9 7.8 7.7 7.6 7.5 7.4 7.3 7.2
f1 (ppm)
1.97 1.87 2.09 3.00 4.86 6.15 2.01
20 19 18 17 16 15 14 13 12 11 10 9 8 7 6 5 4 3 2 1 0 -1 -2 -3 -4 -5 -6 -7 -8 -9
f1 (ppm)

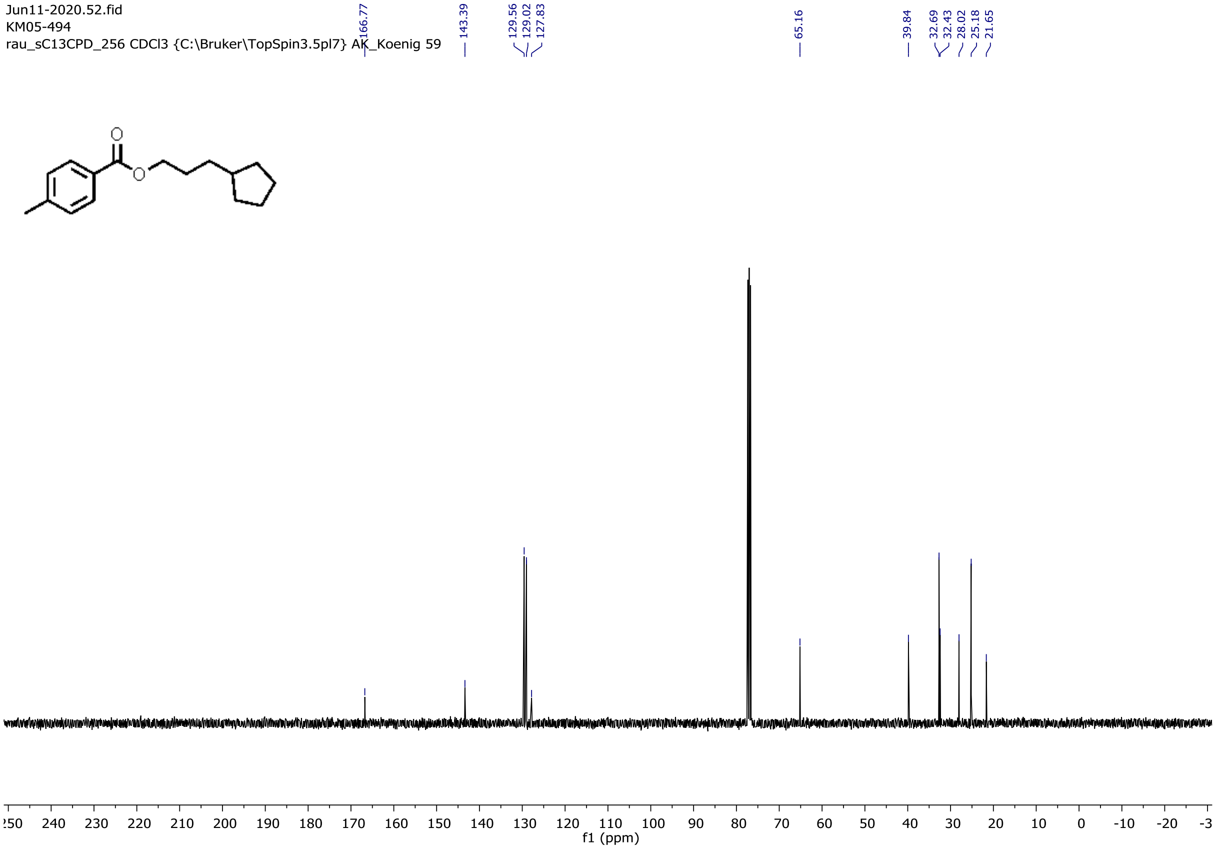
Jun11-2020.52.fid
KM05-494
rau_sC13CPD_256 CDCl3 {C:\Bruker\TopSpin3.5pl7} AK_Koenig 59
166.77
143.39
129.56 129.02 127.83
65.16
39.84
32.69 32.43 28.02 25.18 21.65
250 240 230 220 210 200 190 180 170 160 150 140 130 120 110 100 90 80 70 60 50 40 30 20 10 0 -10 -20
f1 (ppm)

May27-2020.70.fid
KM05-431-F1
rau_sPROTON_64 CDCl3 {C:\Bruker\TopSpin3.5pl7} AK_Koenig 54

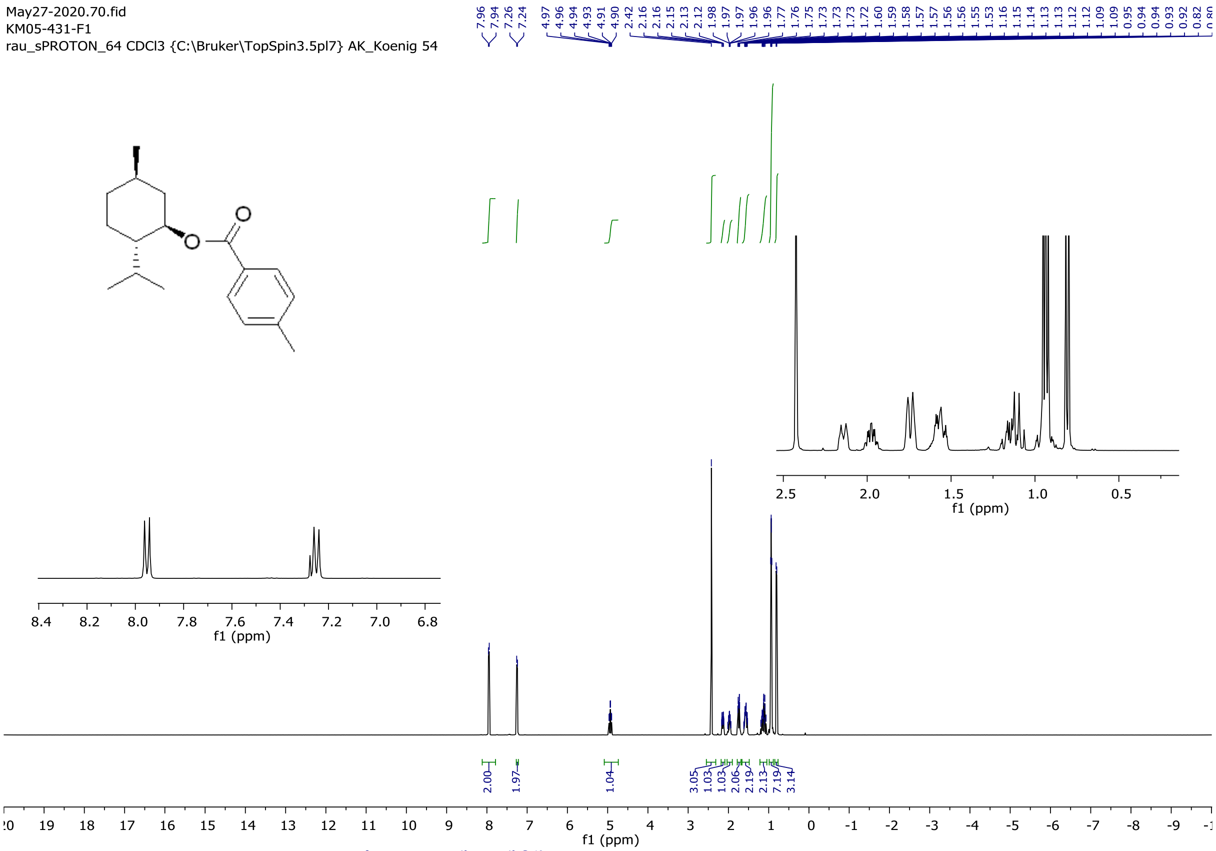

May27-2020.71.fid
KM05-431-F1
rau_sC13CPD_256 CDCl3 {C:\Bruker\TopSpin3.5pl7} AK_Koenig 54

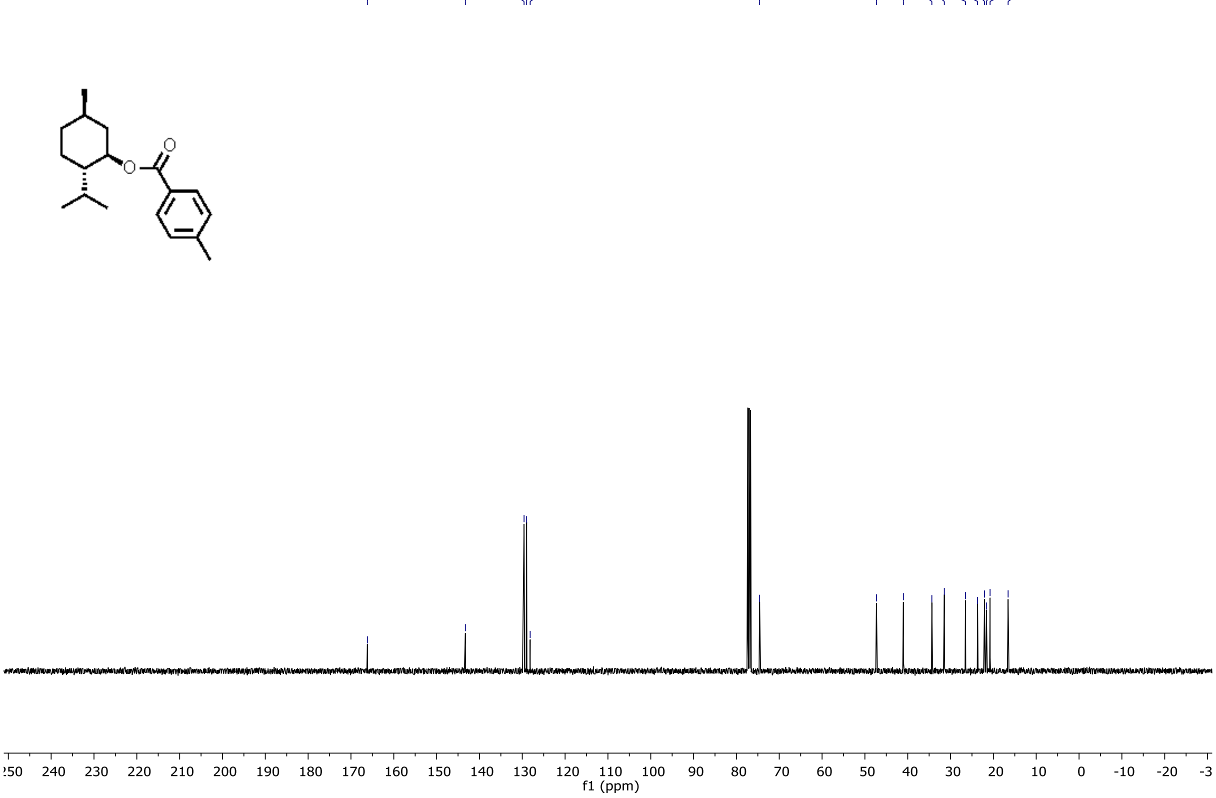

Jun11-2020.11.fid
KM05-490
rau_sPROTON_64 CDCl3 {C:\Bruker\TopSpin3.5pl7} AK_Koenig 55

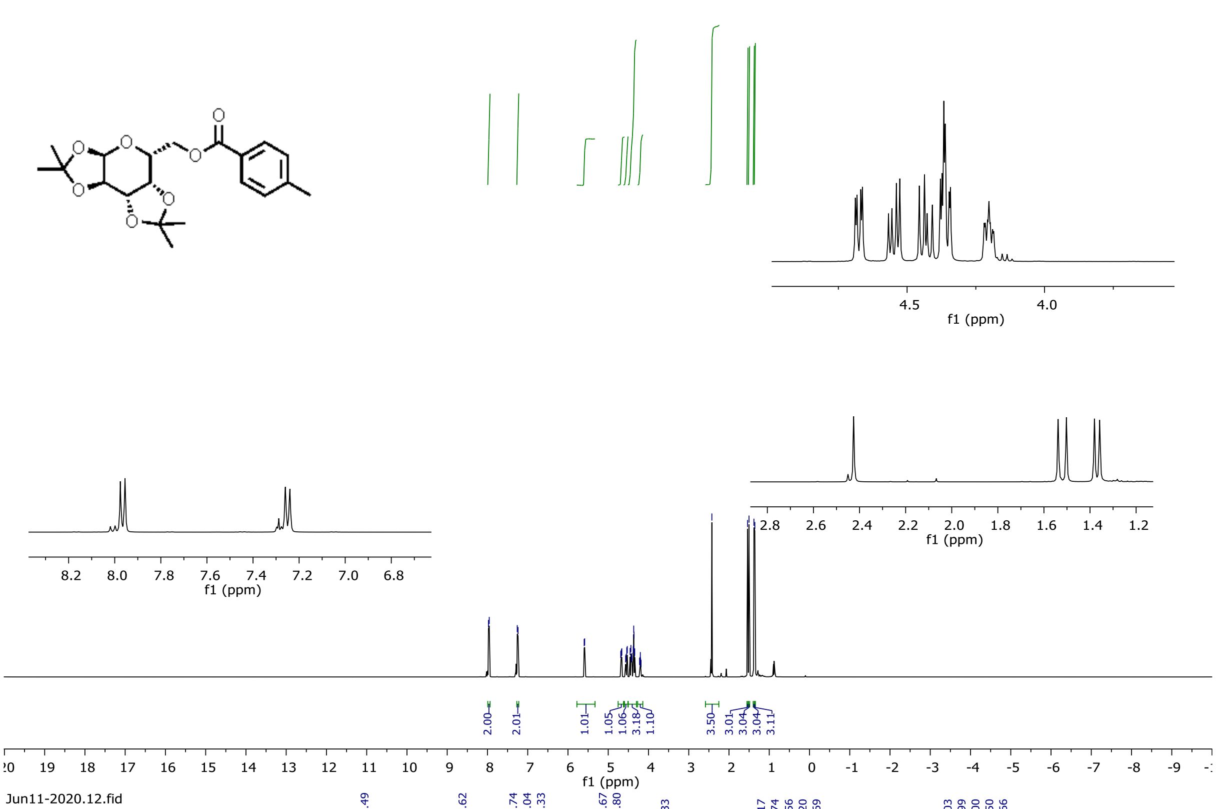

Jun11-2020.12.fid
KM05-490
rau_sC13CPD_256 CDCl3 {C:\Bruker\TopSpin3.5pl7} AK_Koenig 55

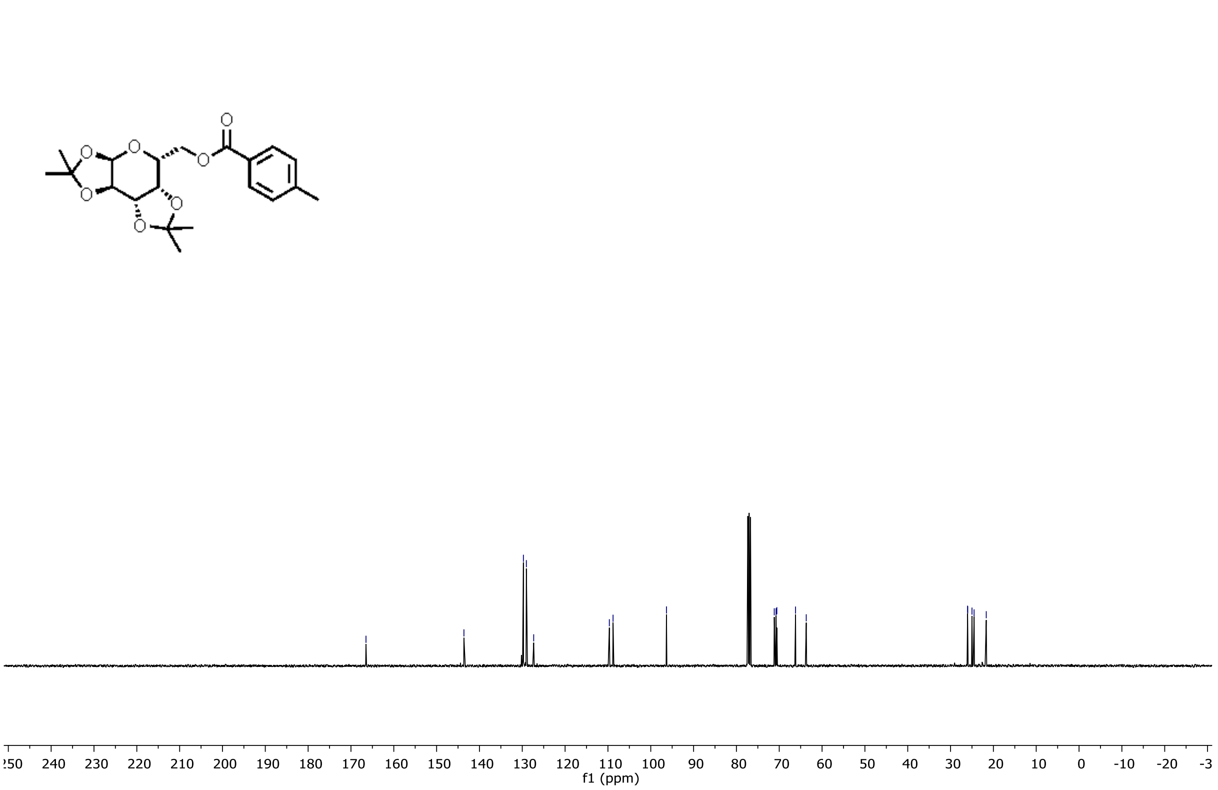

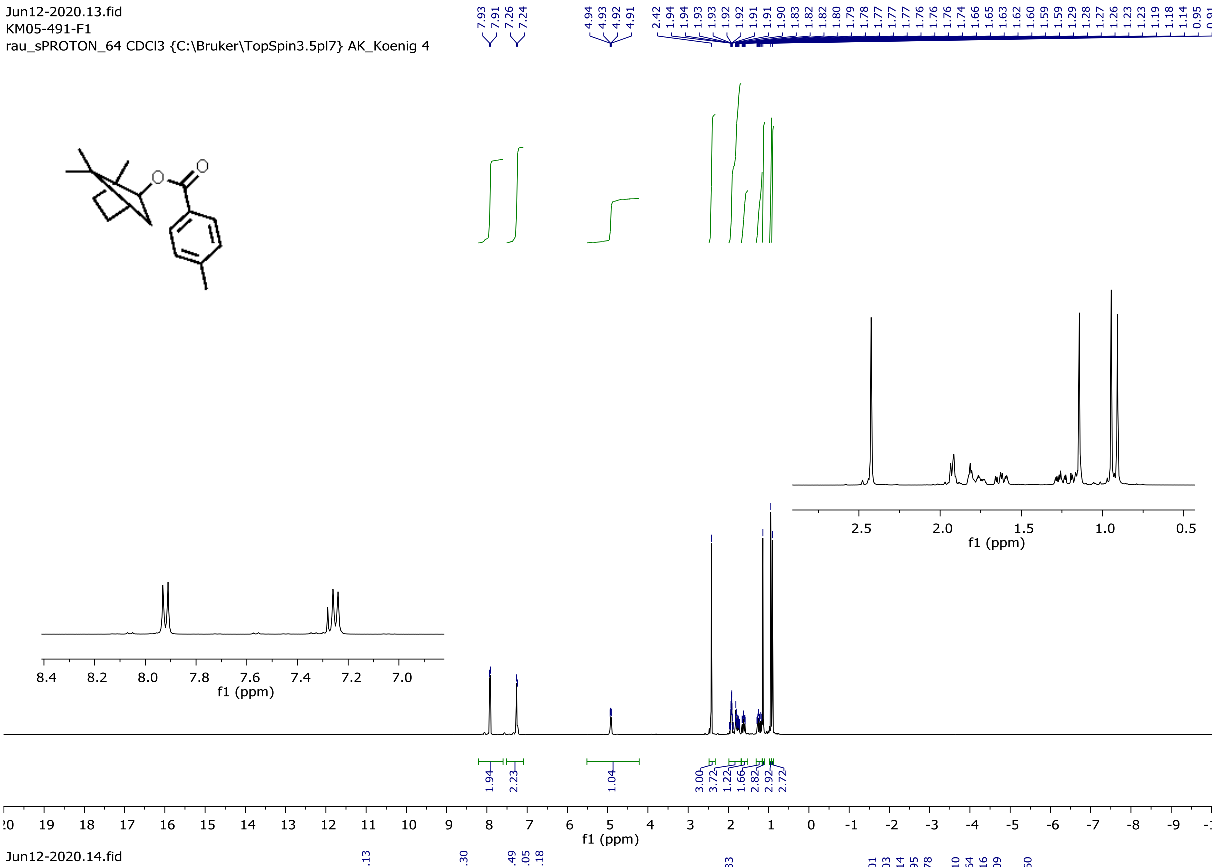
Jun12-2020.13.fid
KM05-491-F1
rau_sPROTON_64 CDCl3 {C:\Bruker\TopSpin3.5pl7} AK_Koenig 4
7.93
7.91
7.26
7.24
4.94
4.93
4.92
4.91
2.42
1.94
1.94
1.93
1.93
1.92
1.92
1.91
1.91
1.90
1.83
1.82
1.82
1.80
1.79
1.78
1.77
1.77
1.77
1.76
1.76
1.76
1.74
1.66
1.65
1.63
1.62
1.60
1.59
1.59
1.29
1.28
1.27
1.26
1.23
1.23
1.19
1.18
1.14
0.95
1.94
2.23
1.04
3.00
3.72
1.22
1.66
2.82
2.92
2.72
f1 (ppm)
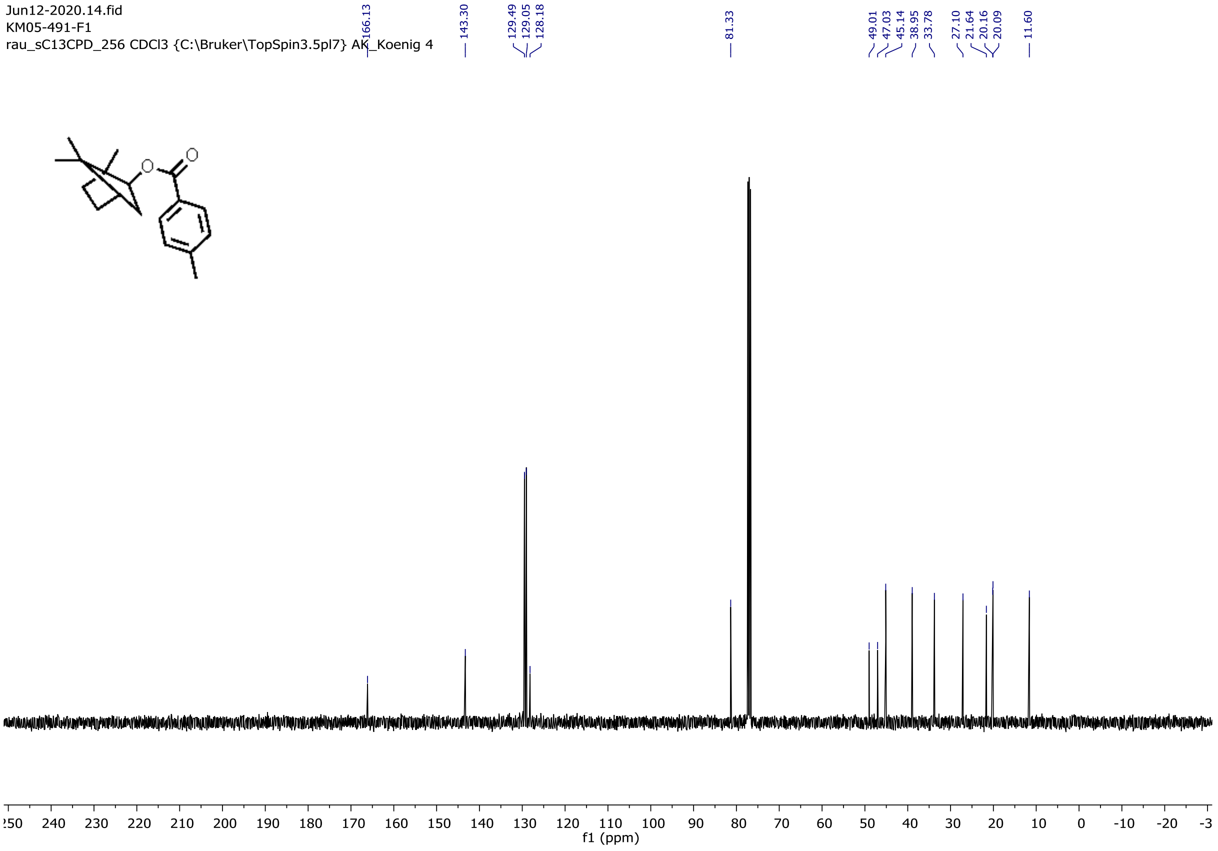
Jun12-2020.14.fid
KM05-491-F1
rau_sC13CPD_256 CDCl3 {C:\Bruker\TopSpin3.5pl7} AK_Koenig 4
166.13
143.30
129.49
129.05
128.18
81.33
49.01
47.03
45.14
38.95
33.78
27.10
21.64
20.16
20.09
11.60
f1 (ppm)

Jun12-2020.73.fid
KM05-495
rau_sPROTON_64 CDCl3 {C:\Bruker\TopSpin3.5pl7} AK_Koenig 13

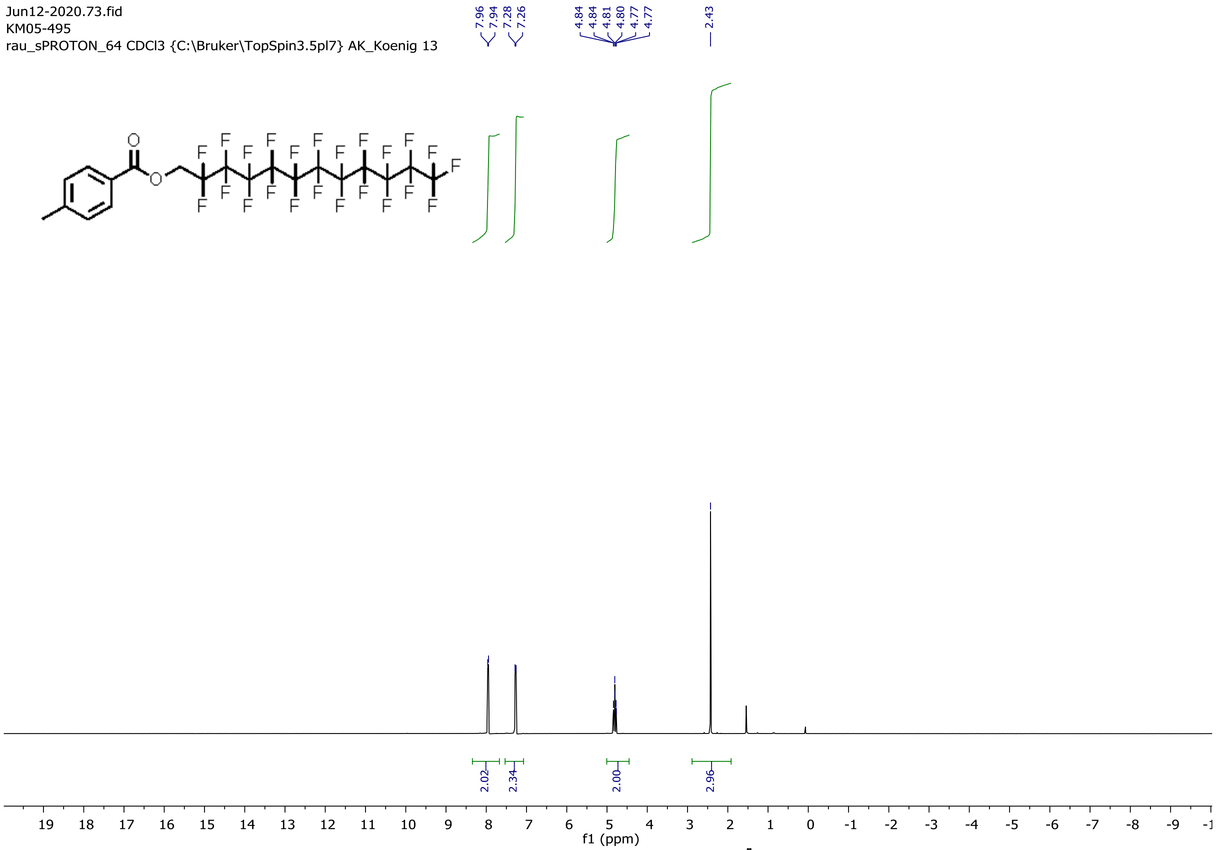

Jun12-2020.74.fid
KM05-495
rau_sC13CPD_256 CDCl3 {C:\Bruker\TopSpin3.5pl7} AK_Koenig 13

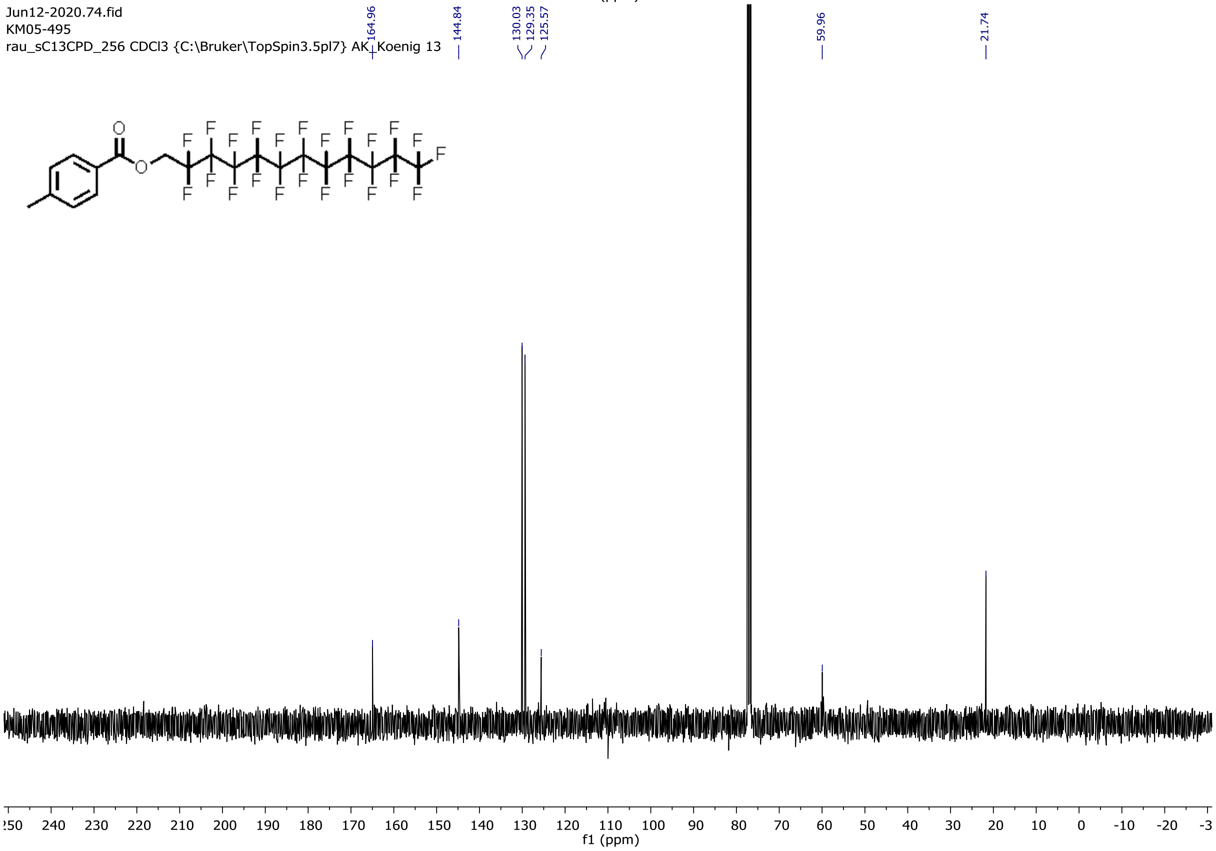

Jun30-2020.82.fid
KM05-495
rau_F19CPD CDCl3 {C:\Bruker\TOPSPIN2.1PL3} AK_Koenig 54

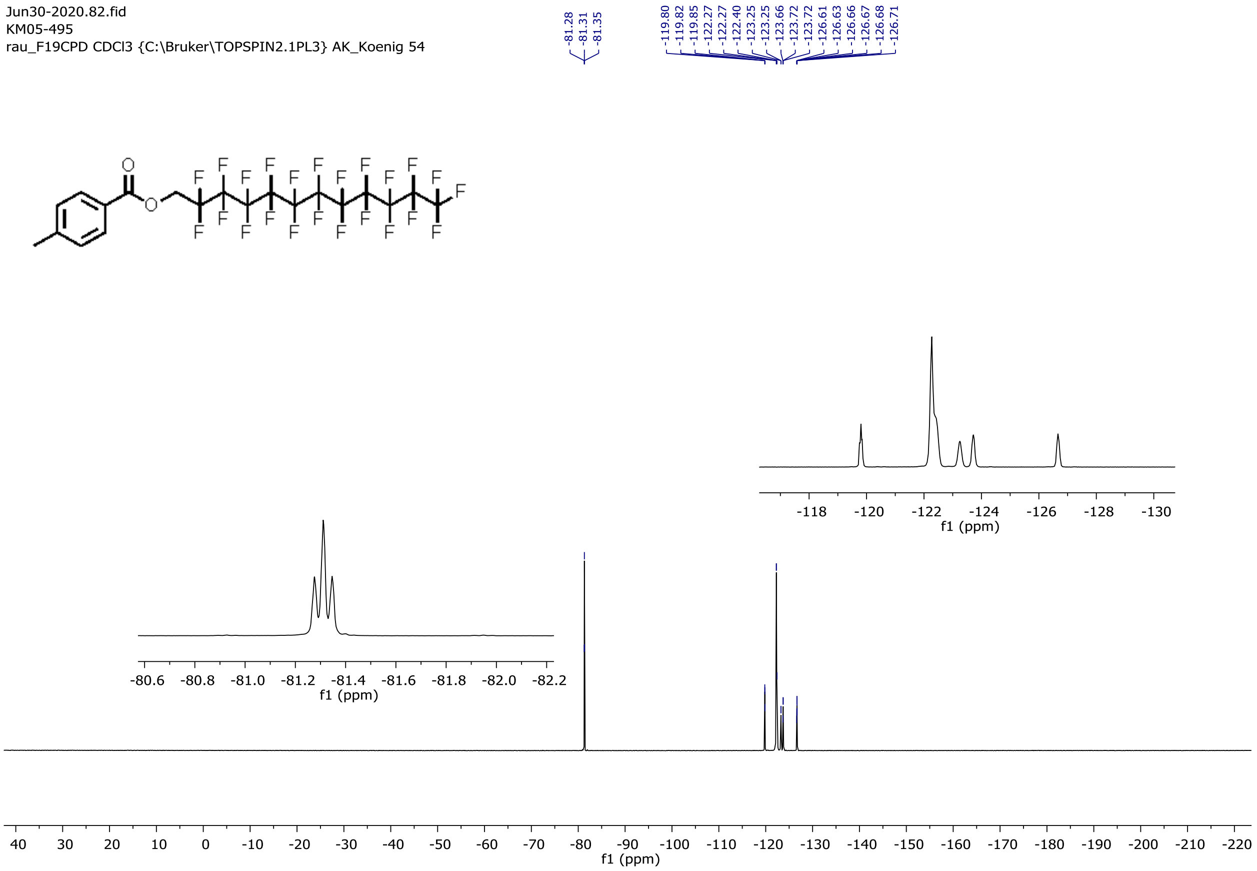

Jun12-2020.81.fid
KM05-496
rau_sPROTON_64 CDCl3 {C:\Bruker\TopSpin3.5pl7} AK_Koenig 14

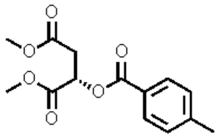

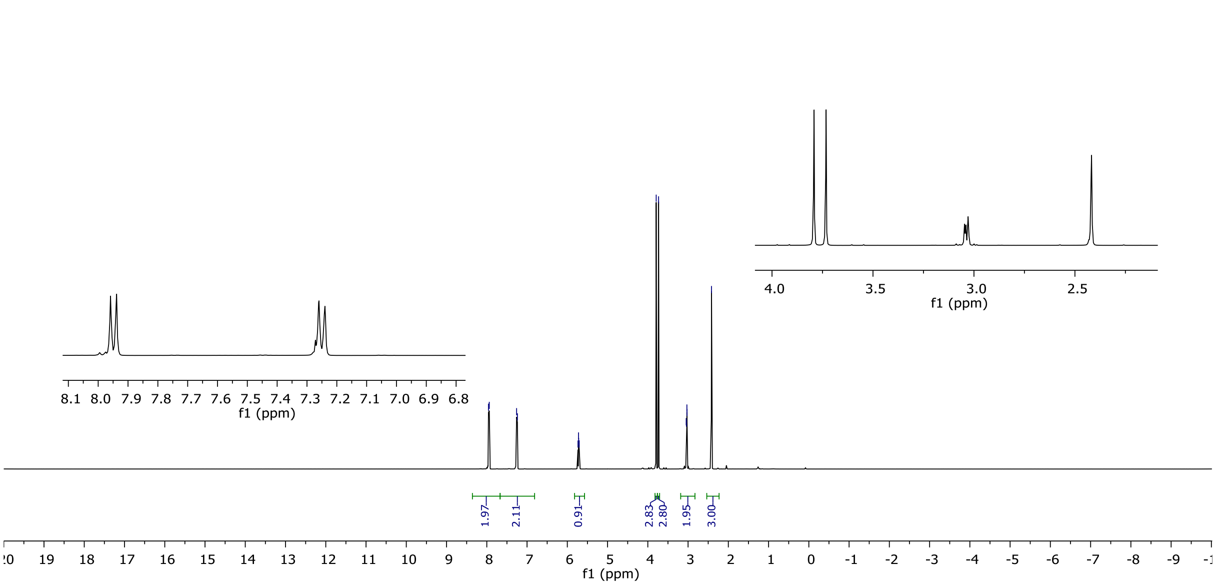

Jun12-2020.82.fid
KM05-496
rau_sC13CPD_256 CDCl3 {C:\Bruker\TopSpin3.5pl7} AK_Koenig 14

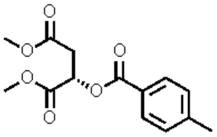

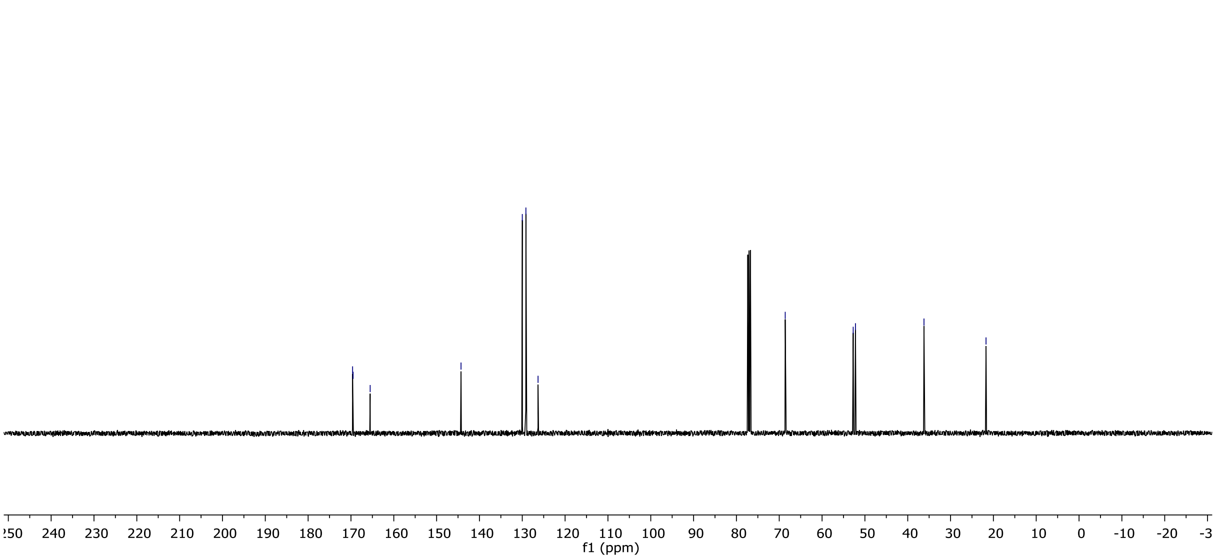

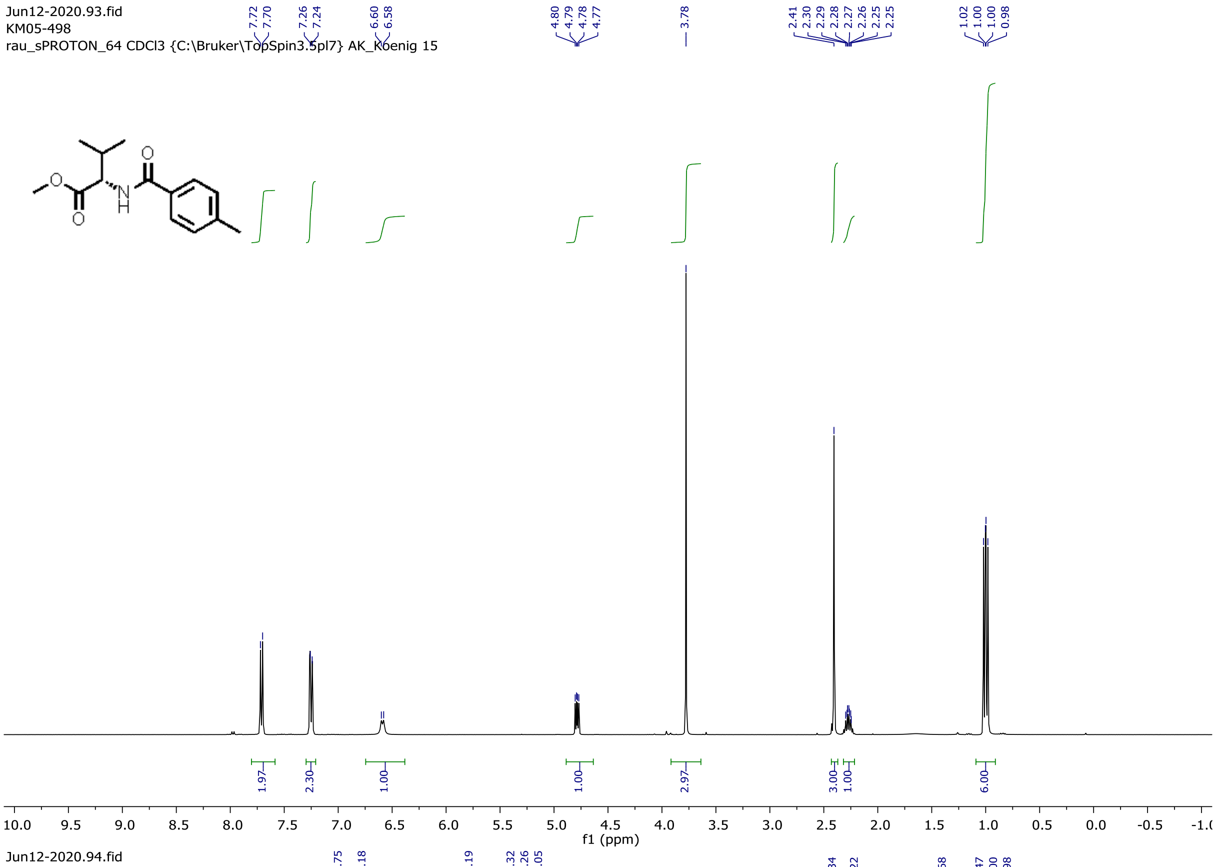
Jun12-2020.93.fid
KM05-498
rau_sPROTON_64 CDCl3 {C:\Bruker\TopSpin3.5pl7} AK_Koenig 15
7.72
7.70
7.26
7.24
6.60
6.58
4.80
4.79
4.78
4.77
3.78
2.41
2.30
2.29
2.28
2.27
2.26
2.25
2.25
1.02
1.00
1.00
0.98
1.97
2.30
1.00
1.00
2.97
3.00
1.00
6.00
f1 (ppm)

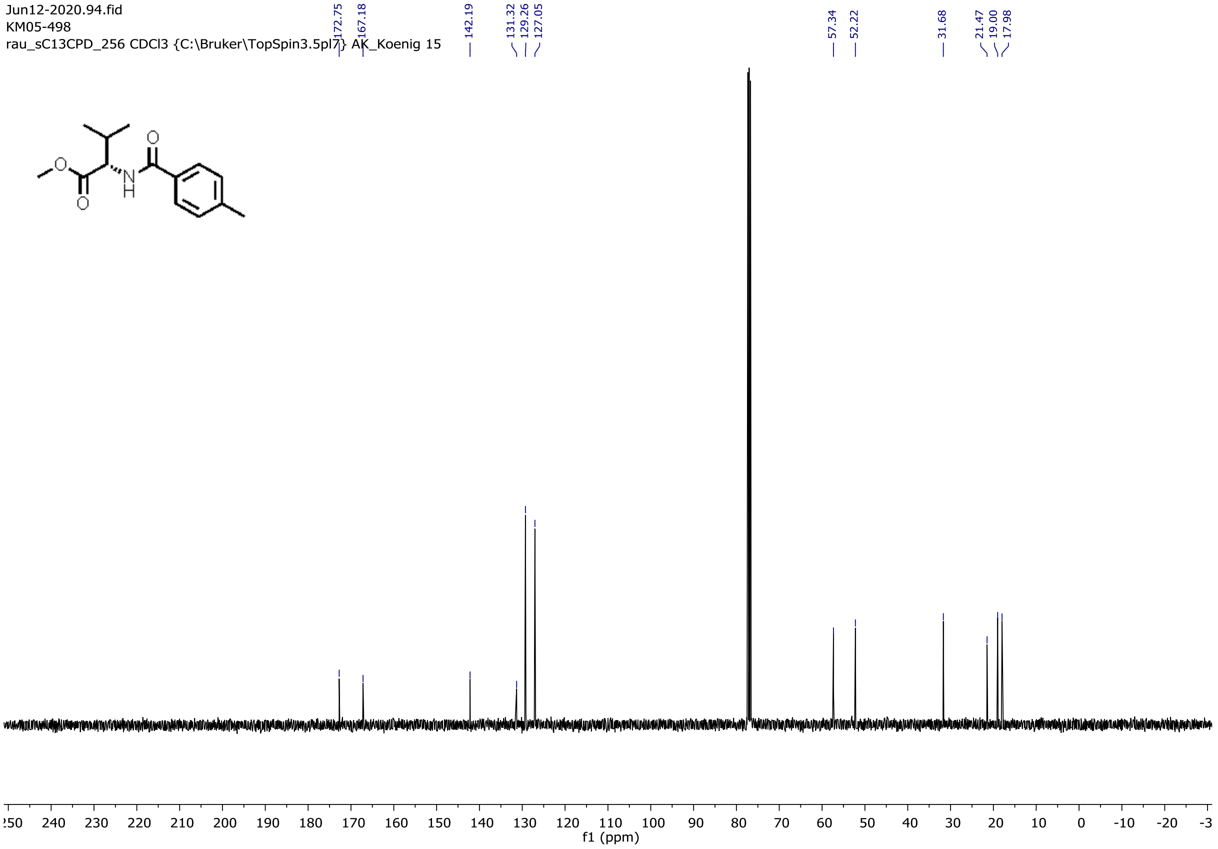
Jun12-2020.94.fid
KM05-498
rau_sC13CPD_256 CDCl3 {C:\Bruker\TopSpin3.5pl7} AK_Koenig 15
172.75
167.18
142.19
131.32
129.26
127.05
57.34
52.22
31.68
21.47
19.00
17.98
f1 (ppm)

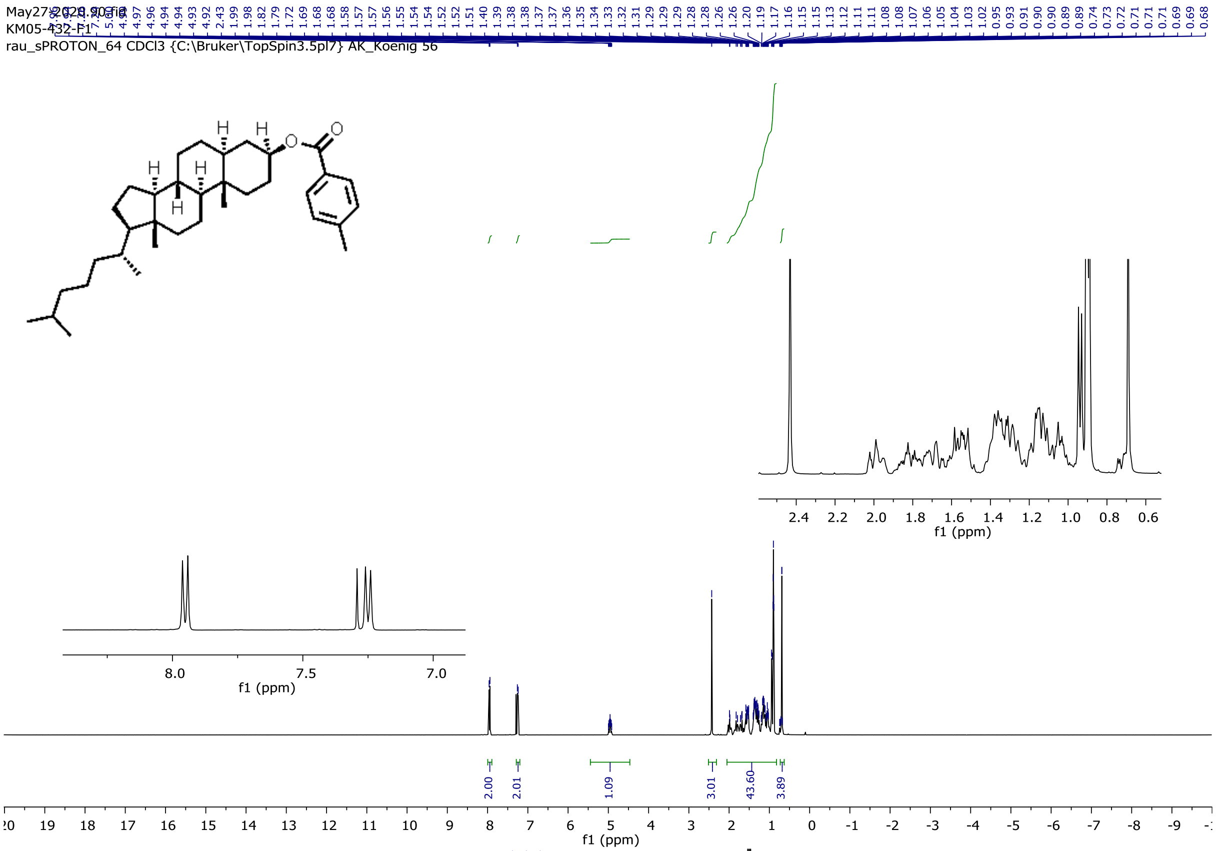
May27-2020.90.fid
KM05-432-F1
rau_sPROTON_64 CDCl3 {C:\Bruker\TopSpin3.5pl7} AK_Koenig 56
2.00
2.01
1.09
3.01
43.60
3.89
f1 (ppm)

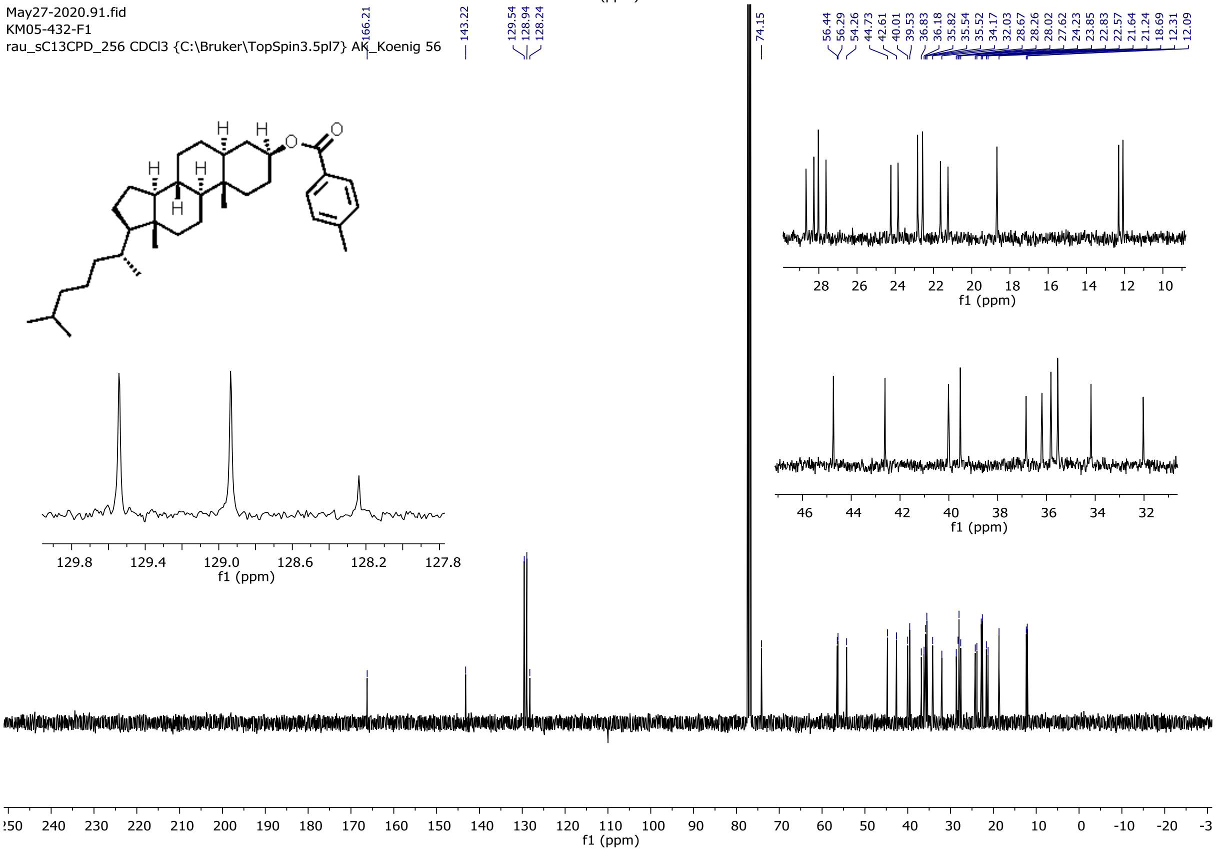
May27-2020.91.fid
KM05-432-F1
rau_sC13CPD_256 CDCl3 {C:\Bruker\TopSpin3.5pl7} AK_Koenig 56
166.21
143.22
129.54
128.94
128.24
74.15
56.44
56.29
54.26
44.73
42.61
40.01
39.53
36.83
36.18
35.82
35.54
35.52
34.17
32.03
28.67
28.26
28.02
27.62
24.23
23.85
22.83
22.57
21.64
21.24
18.69
12.31
12.09
f1 (ppm)

Jul03-2020.90.fid
KM05-549
rau_sPROTON_64 CDCl3 {C:\Bruker\TopSpin3.5pl7} AK_Koenig 53

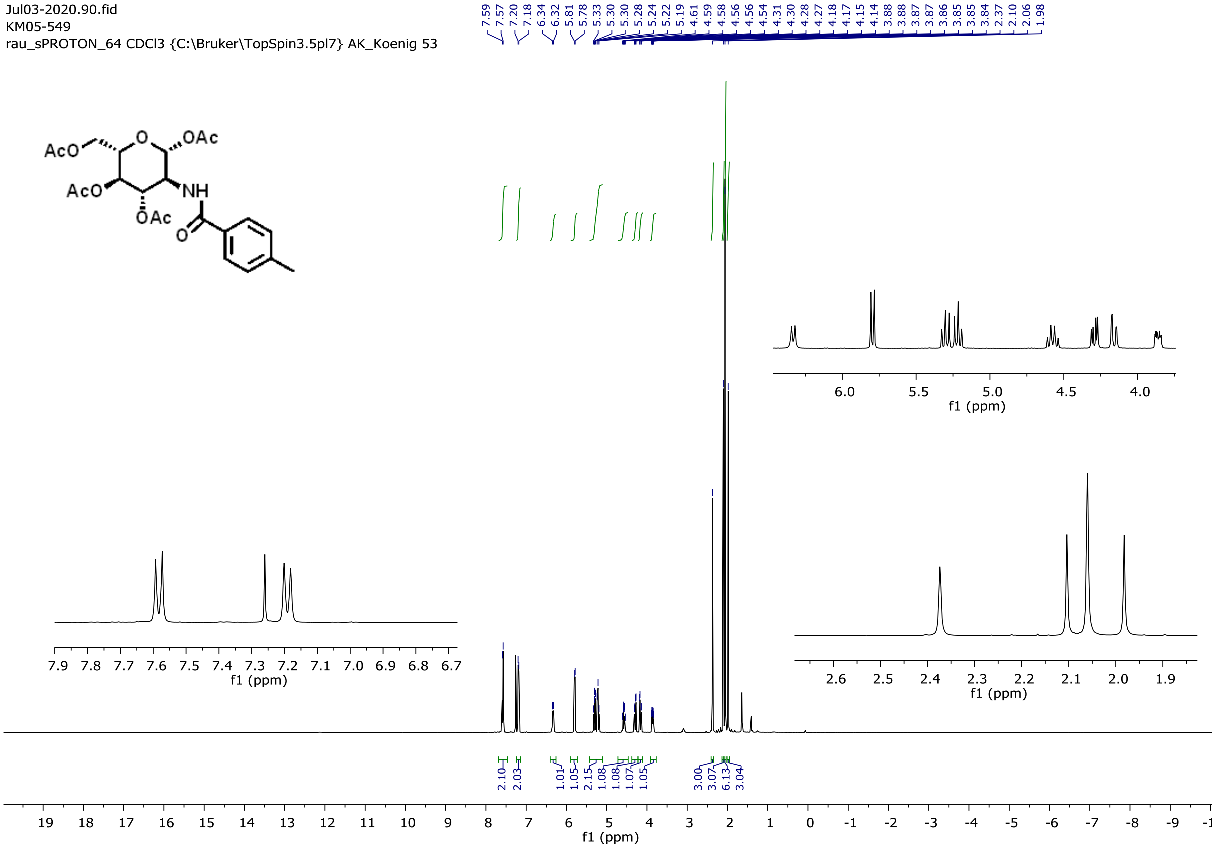

Jul03-2020.91.fid
KM05-549
rau_sC13CPD_256 CDCl3 {C:\Bruker\TopSpin3.5pl7} AK_Koenig 53

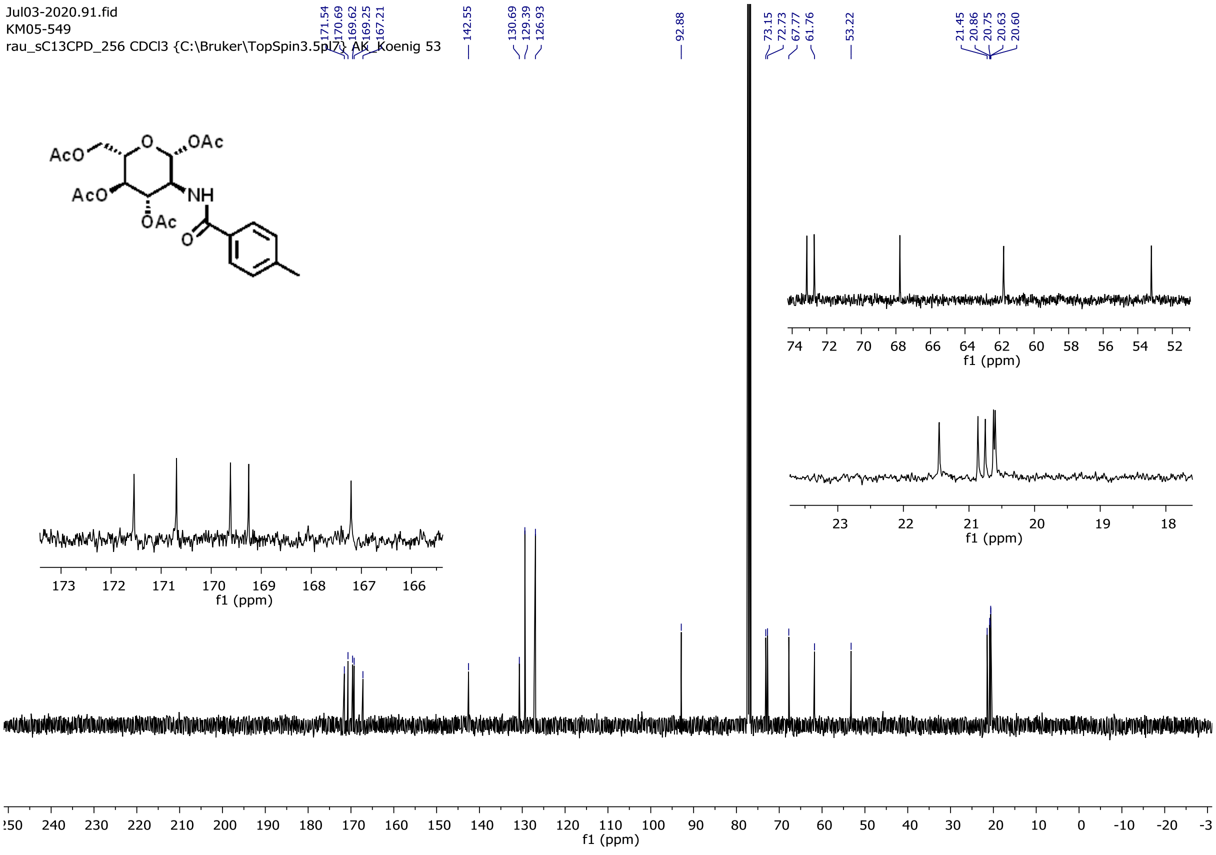

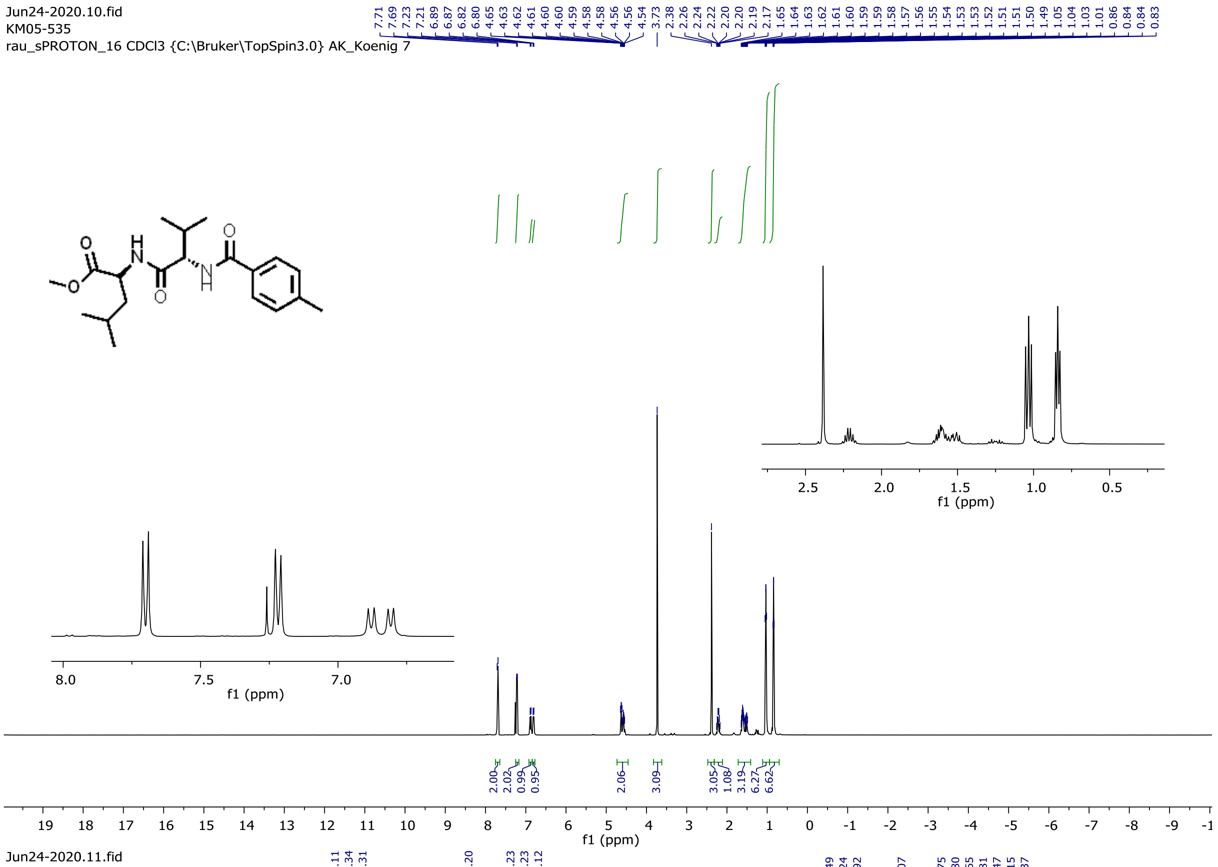
Jun24-2020.10.fid
KM05-535
rau_sPROTON_16 CDCl3 {C:\Bruker\TopSpin3.0} AK_Koenig 7
7.71 7.69 7.23 7.21 6.89 6.87 6.82 6.80 4.65 4.63 4.62 4.61 4.60 4.60 4.59 4.58 4.58 4.56 4.56 4.54 3.73 2.38 2.26 2.24 2.22 2.20 2.20 2.19 2.17 1.65 1.64 1.63 1.62 1.61 1.60 1.59 1.59 1.58 1.57 1.56 1.55 1.54 1.53 1.53 1.52 1.51 1.51 1.50 1.49 1.05 1.04 1.03 1.01 0.86 0.84 0.84 0.83
2.00 2.02 0.99 0.95 2.06 3.09 3.05 1.08 3.19 6.27 6.62
f1 (ppm)

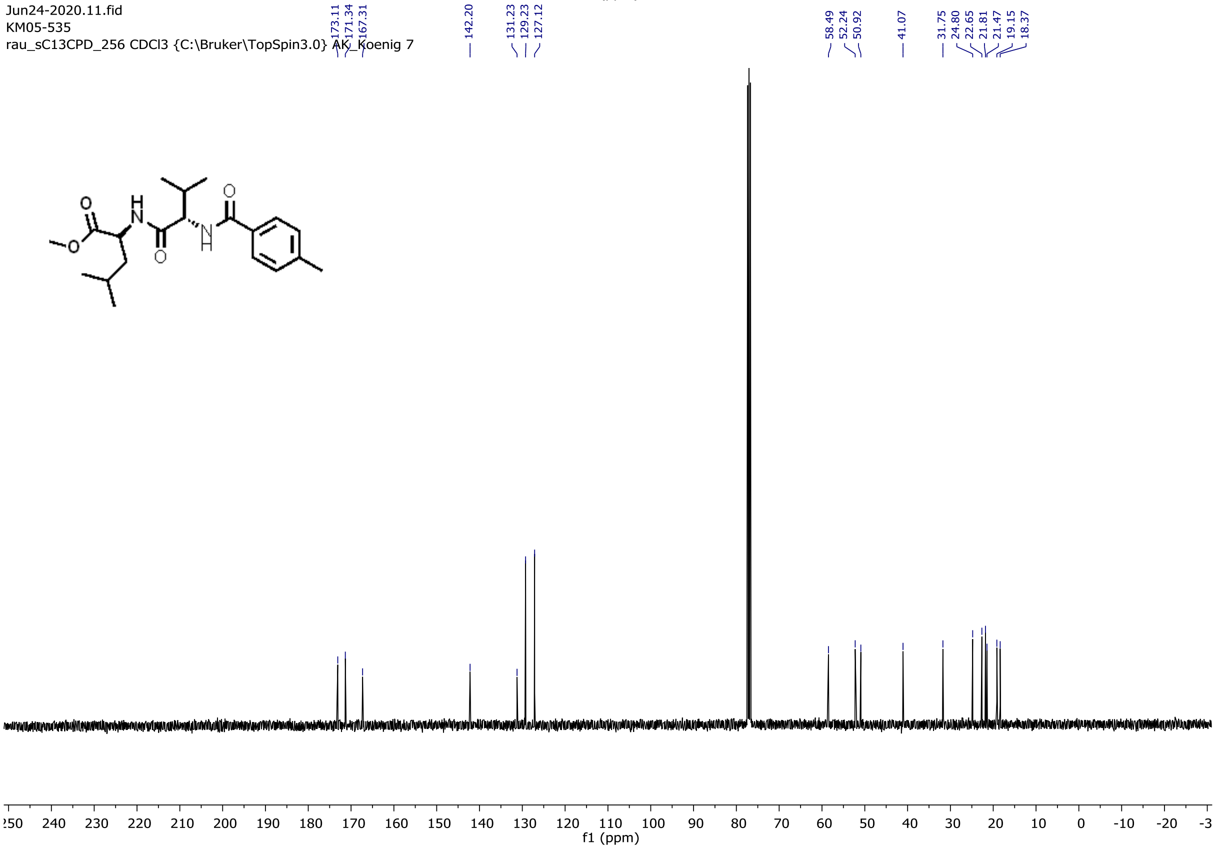
Jun24-2020.11.fid
KM05-535
rau_sC13CPD_256 CDCl3 {C:\Bruker\TopSpin3.0} AK_Koenig 7
173.11 171.34 167.31 142.20 131.23 129.23 127.12 58.49 52.24 50.92 41.07 31.75 24.80 22.65 21.81 21.47 19.15 18.37
f1 (ppm)

May27-2020.60.fid
KM05-4-ome-oxime
rau_sPROTON_64 CDCl3 {C:\Bruker\TopSpin3.5pl7} AK_Koenig 53

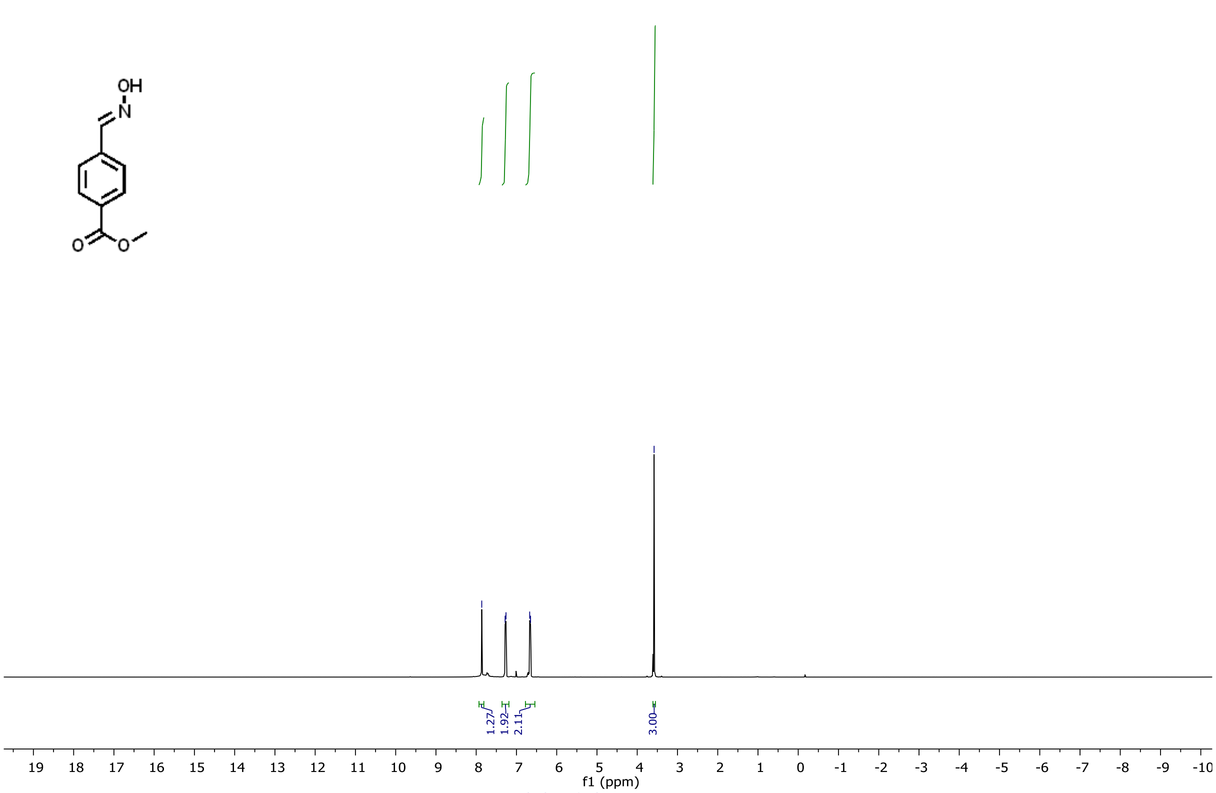

May27-2020.61.fid
KM05-4-ome-oxime
rau_sC13CPD_256 CDCl3 {C:\Bruker\TopSpin3.5pl7} AK_Koenig 53

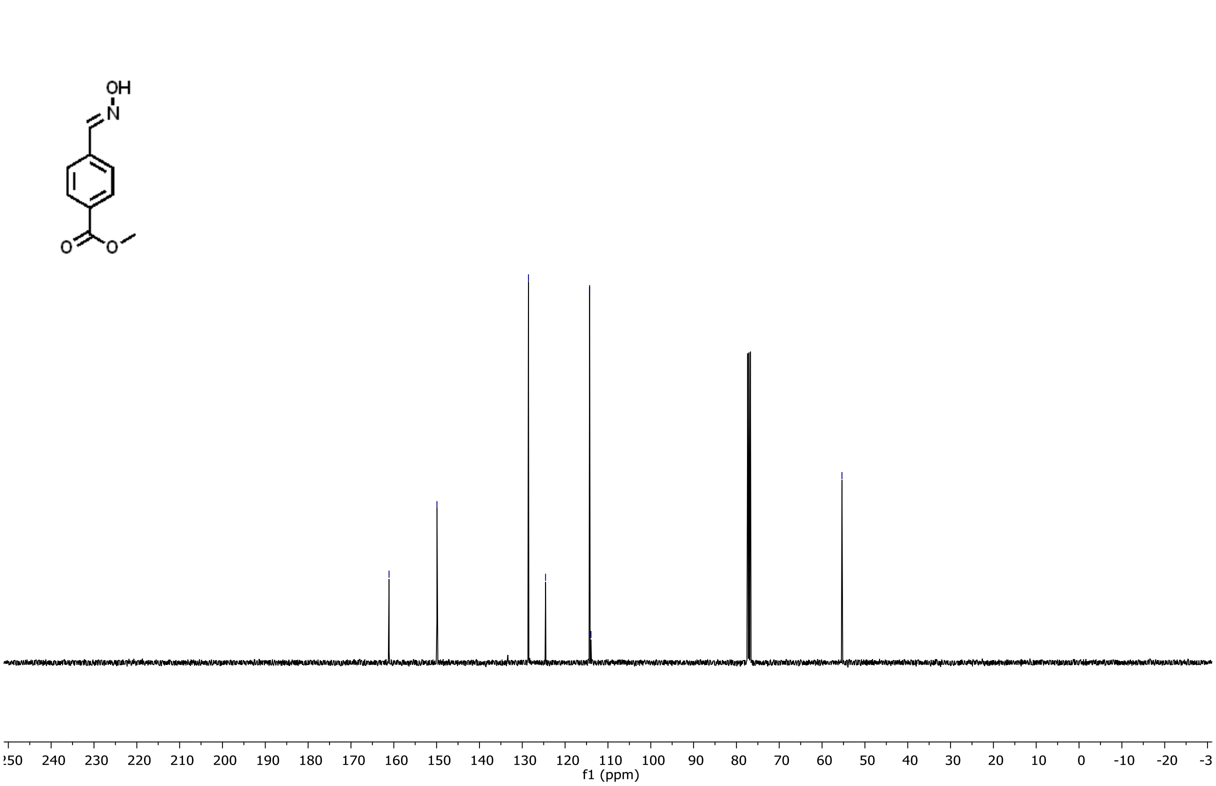

Jul23-2020.20.fid
KM05-617
rau_sPROTON_16 DMSO {C:\Bruker\TopSpin3.5pl7} AK_Koenig 16

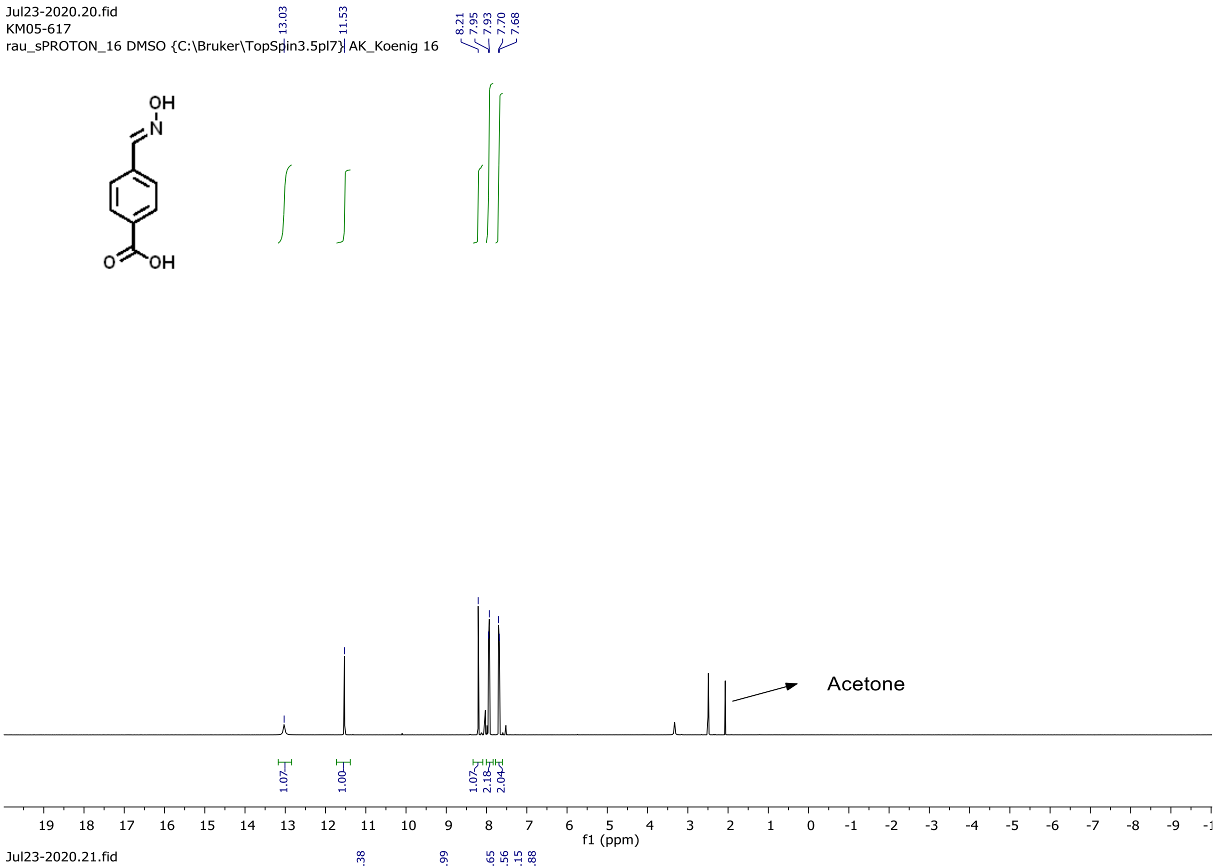

Jul23-2020.21.fid
KM05-617
rau_sC13CPD_256 DMSO {C:\Bruker\TopSpin3.5pl7} AK_Koenig 16

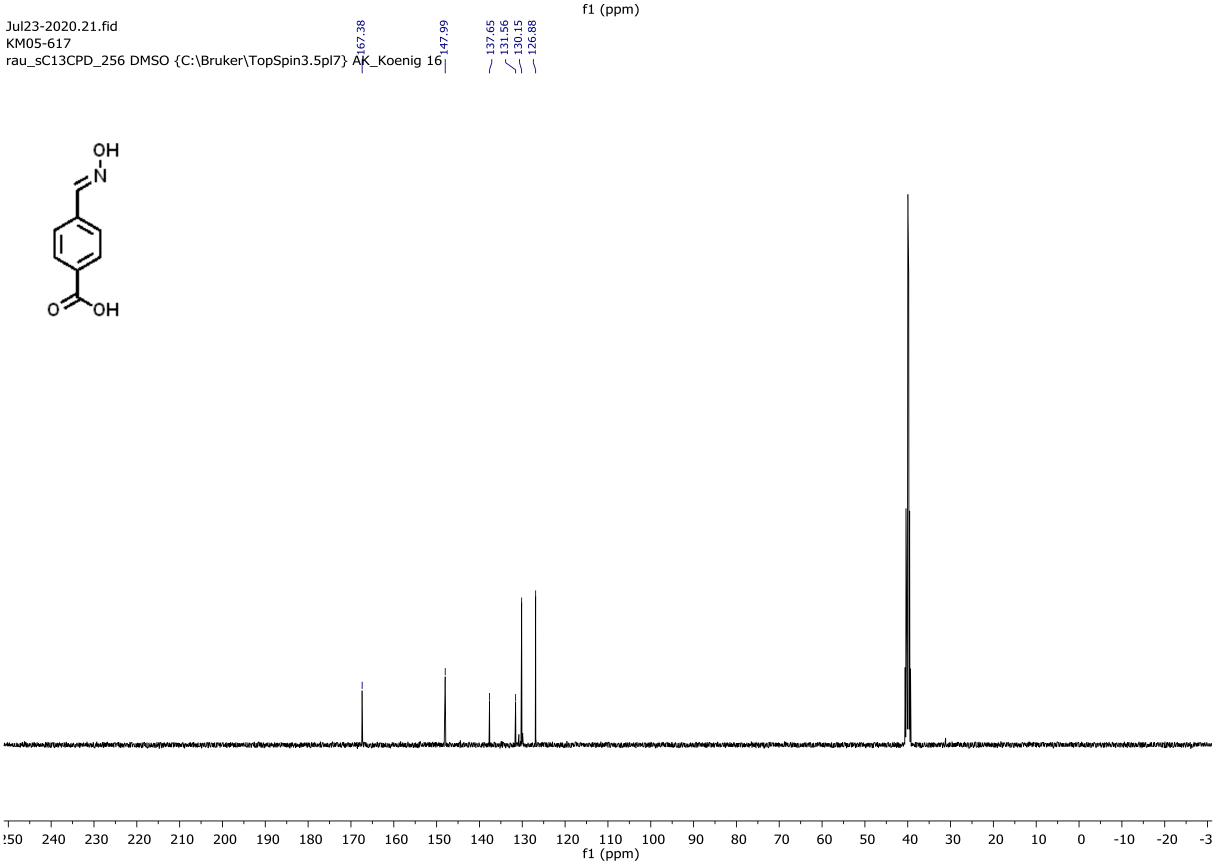

Jul23-2020.40.fid
KM05-619
rau_sPROTON_16 DMSO {C:\Bruker\TopSpin3.5pl7} AK_Koenig 18

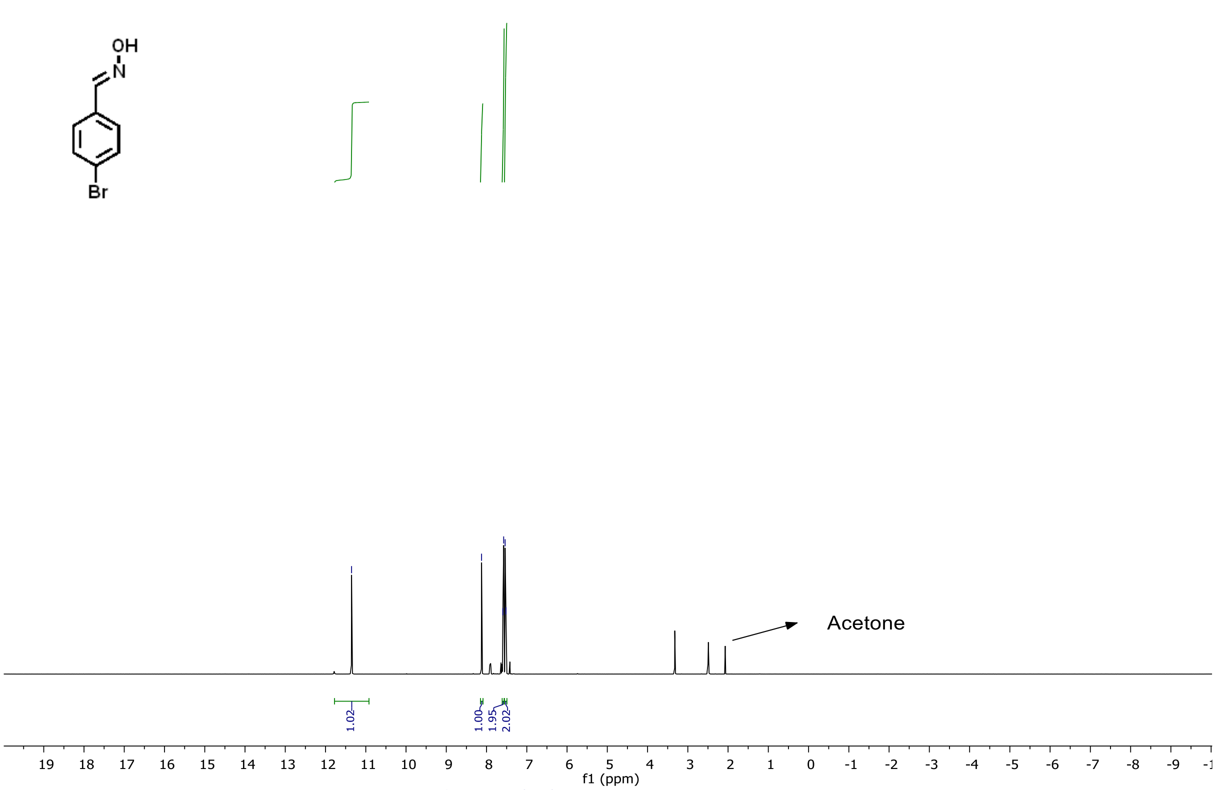

Jul23-2020.41.fid
KM05-619
rau_sC13CPD_256 DMSO {C:\Bruker\TopSpin3.5pl7} AK_Koenig 18

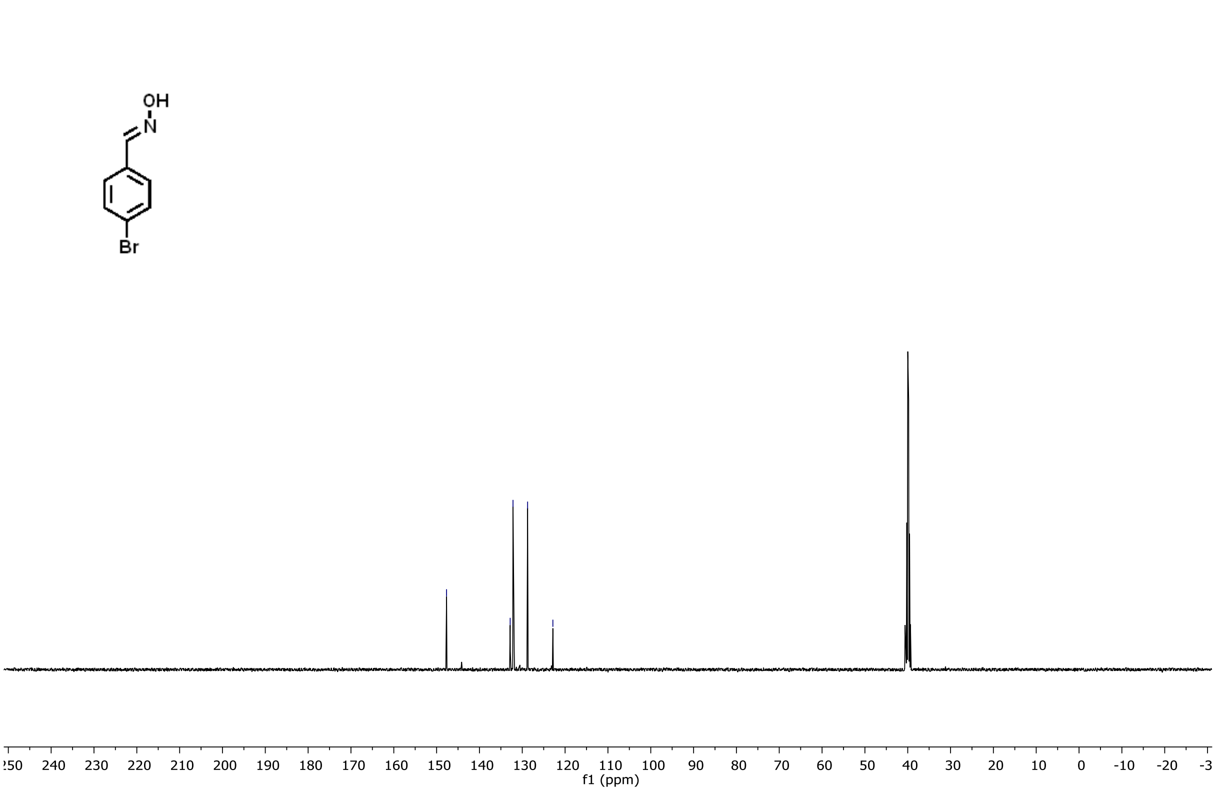

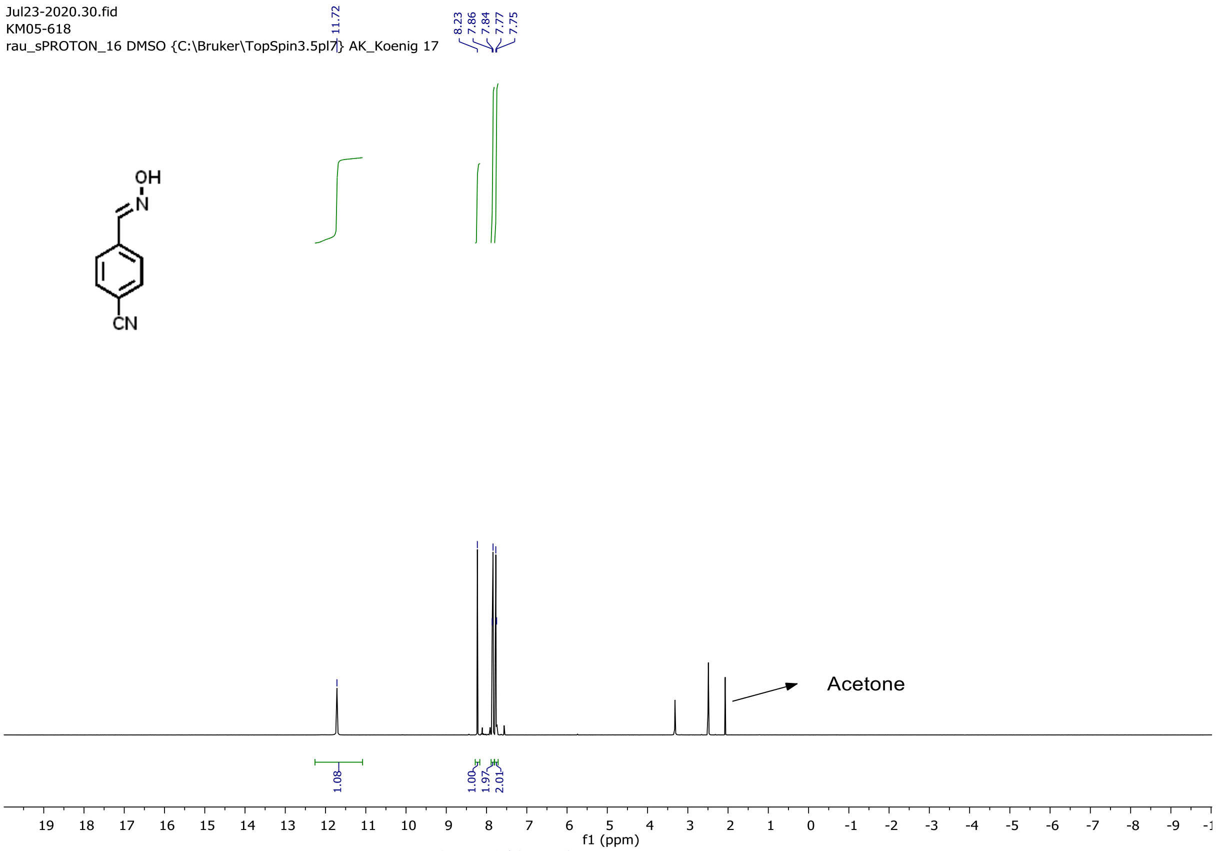
Jul23-2020.30.fid
KM05-618
rau_sPROTON_16 DMSO {C:\Bruker\TopSpin3.5pl7} AK_Koenig 17
11.72
8.23
7.86
7.84
7.77
7.75
OH
N
CN
Acetone
1.08
1.00
1.97
2.01
f1 (ppm)

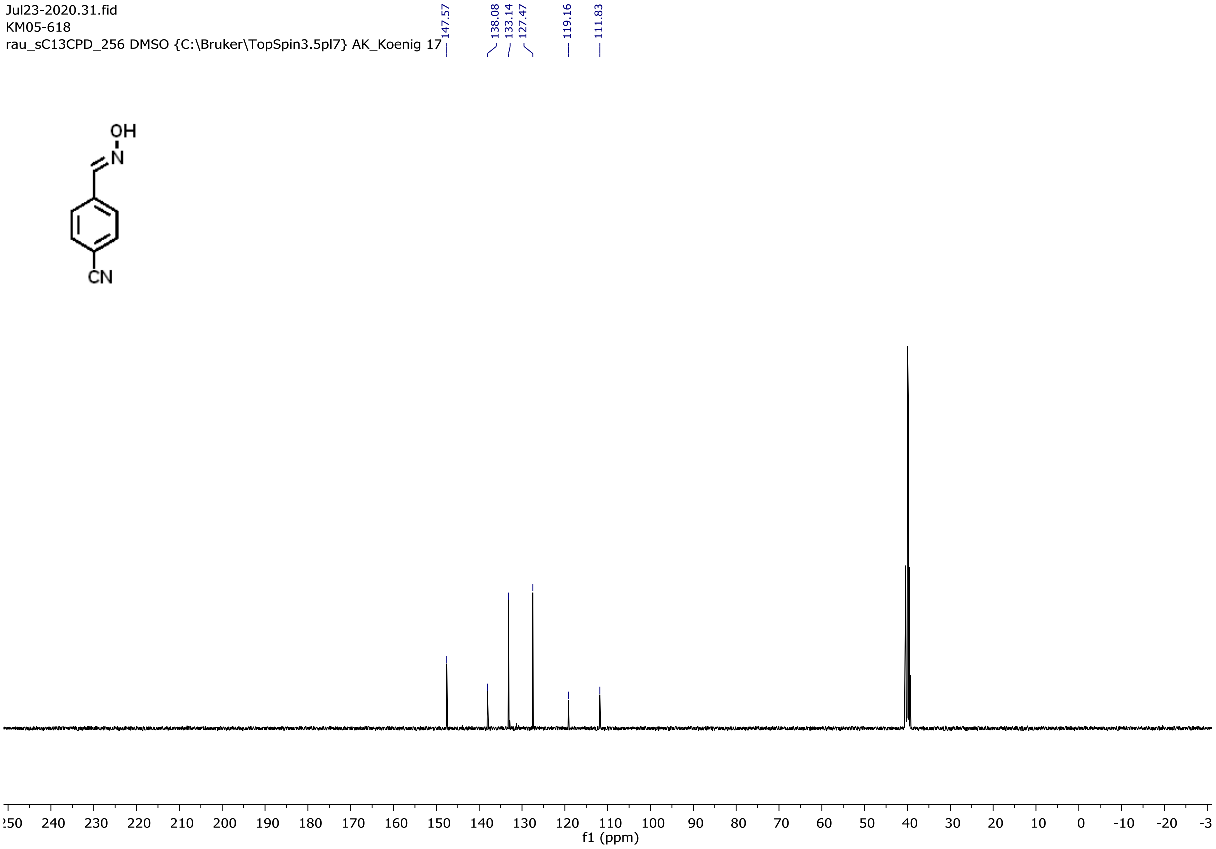
Jul23-2020.31.fid
KM05-618
rau_sC13CPD_256 DMSO {C:\Bruker\TopSpin3.5pl7} AK_Koenig 17
147.57
138.08
133.14
127.47
119.16
111.83
OH
N
CN
f1 (ppm)

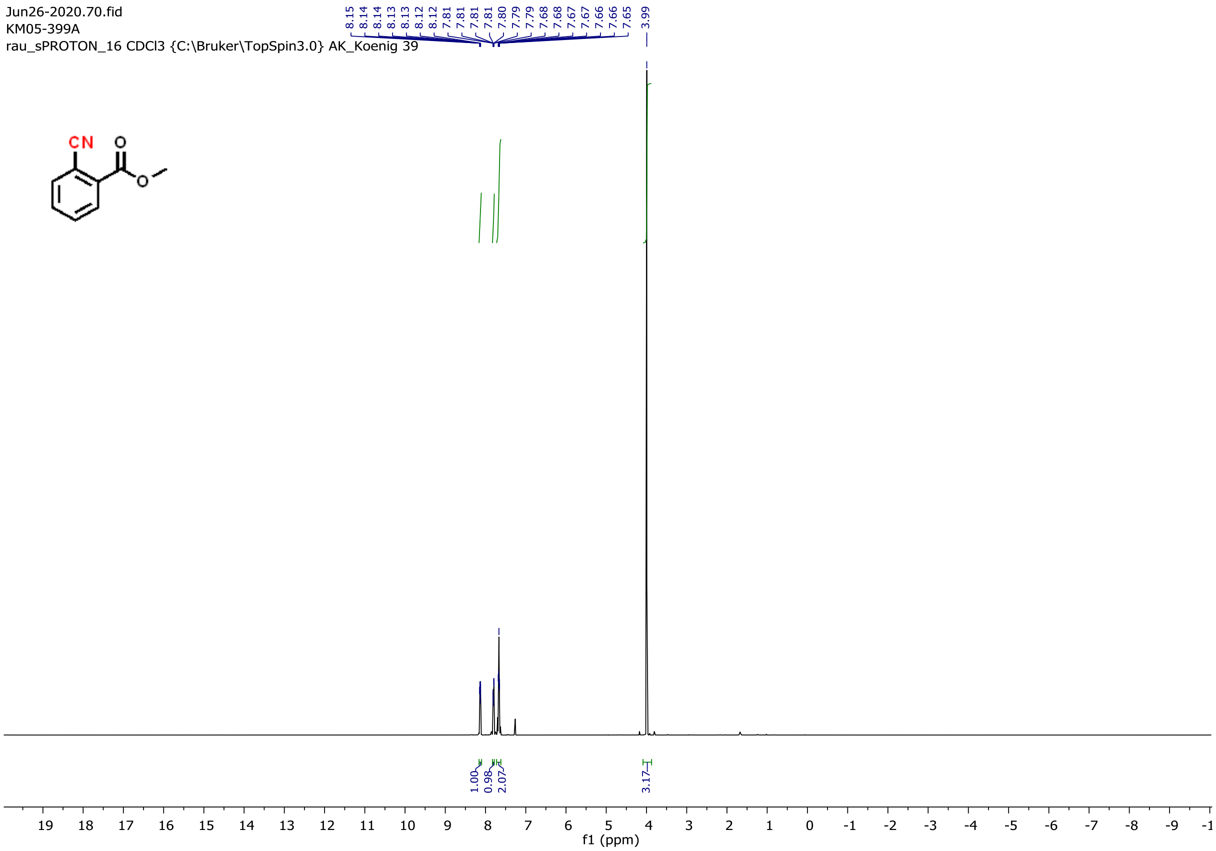
Jun26-2020.70.fid
KM05-399A
rau_sPROTON_16 CDCl3 {C:\Bruker\TopSpin3.0} AK_Koenig 39
8.15
8.14
8.14
8.13
8.13
8.12
8.12
7.81
7.81
7.81
7.80
7.79
7.79
7.68
7.68
7.67
7.66
7.66
7.65
3.99
CN
O
O
1.00
0.98
2.07
3.17
19 18 17 16 15 14 13 12 11 10 9 8 7 6 5 4 3 2 1 0 -1 -2 -3 -4 -5 -6 -7 -8 -9 -1
f1 (ppm)

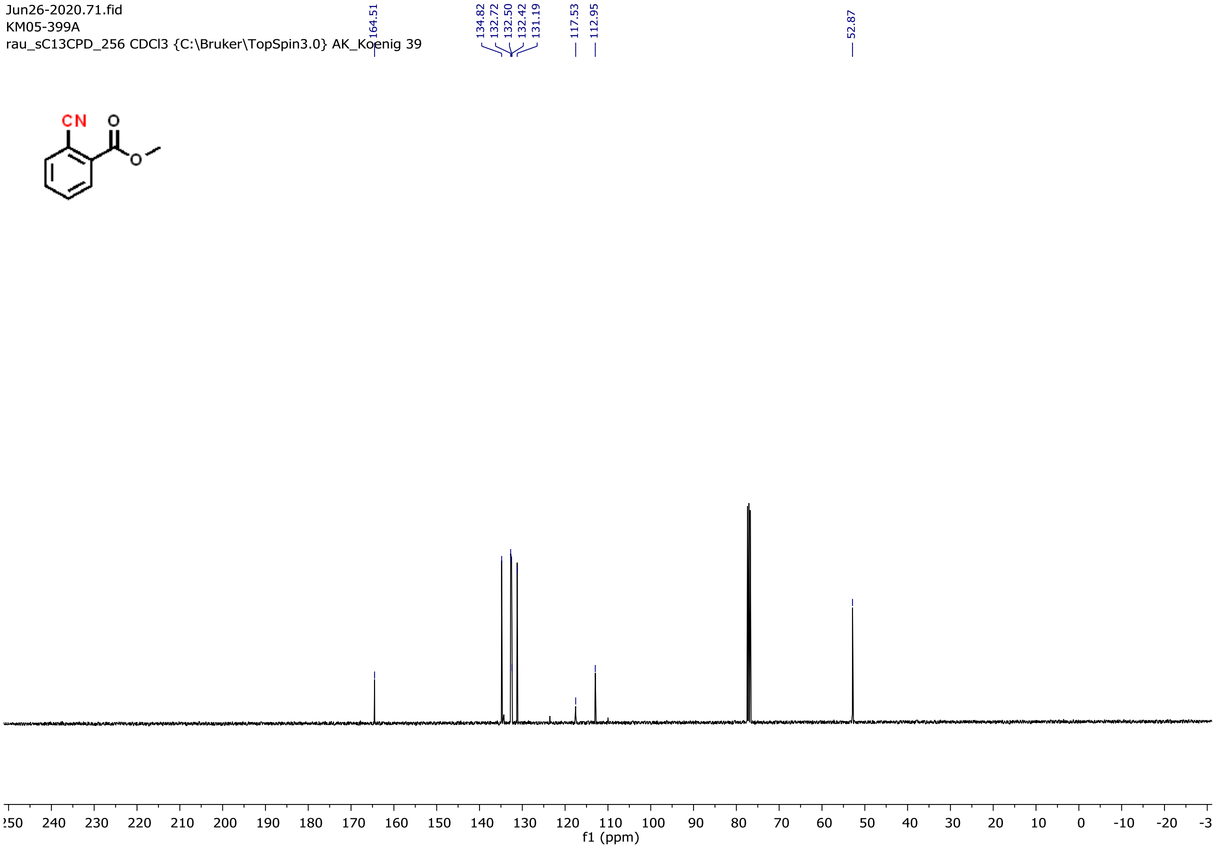
Jun26-2020.71.fid
KM05-399A
rau_sC13CPD_256 CDCl3 {C:\Bruker\TopSpin3.0} AK_Koenig 39
164.51
134.82
132.72
132.50
132.42
131.19
117.53
112.95
52.87
CN
O
O
250 240 230 220 210 200 190 180 170 160 150 140 130 120 110 100 90 80 70 60 50 40 30 20 10 0 -10 -20 -3
f1 (ppm)

Jun26-2020.80.fid
KM05-400
rau_sPROTON_16 CDCl3 {C:\Bruker\TopSpin3.0} AK_Koenig 40

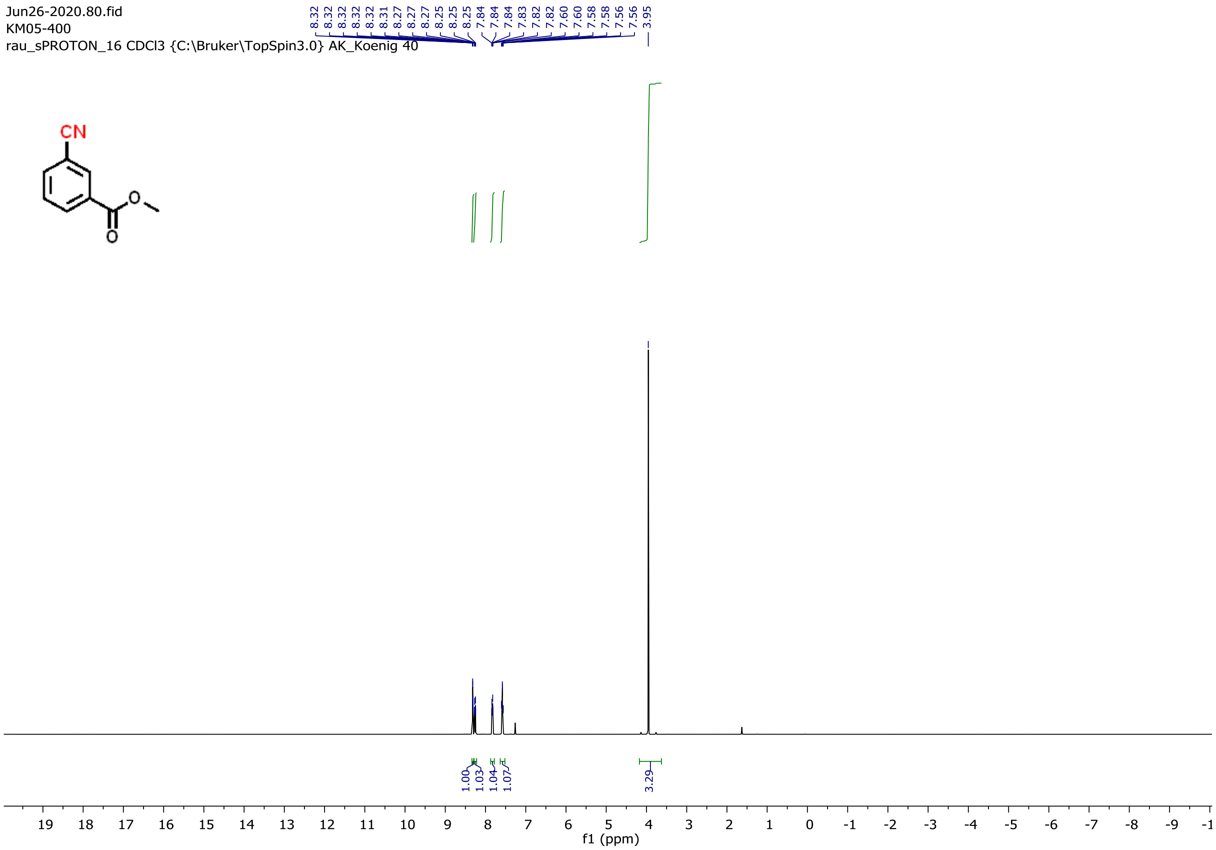

Jun26-2020.81.fid
KM05-400
rau_sC13CPD_256 CDCl3 {C:\Bruker\TopSpin3.0} AK_Koenig 40

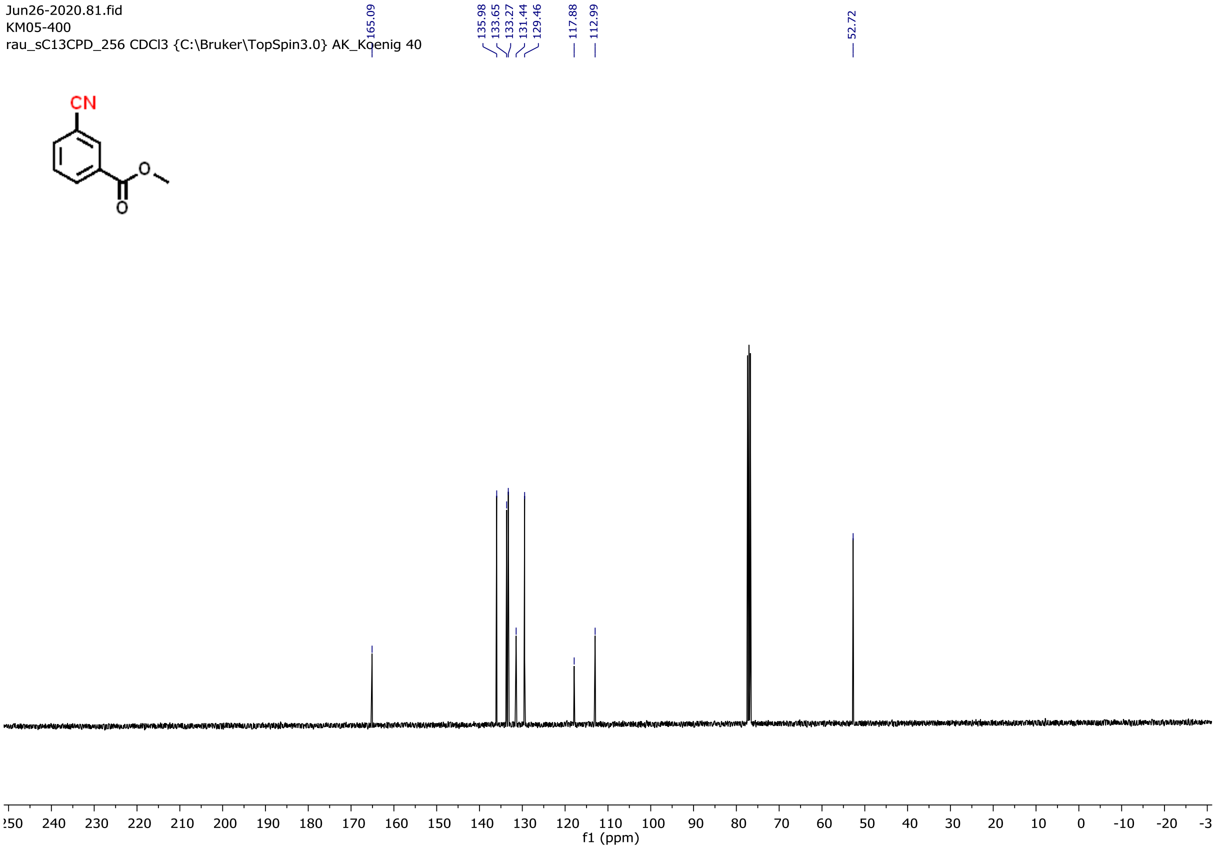

Jun26-2020.40.fid
KM05-284
rau_sPROTON_16 CDCl3 {C:\Bruker\TopSpin3.0} AK_Koenig 36

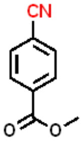

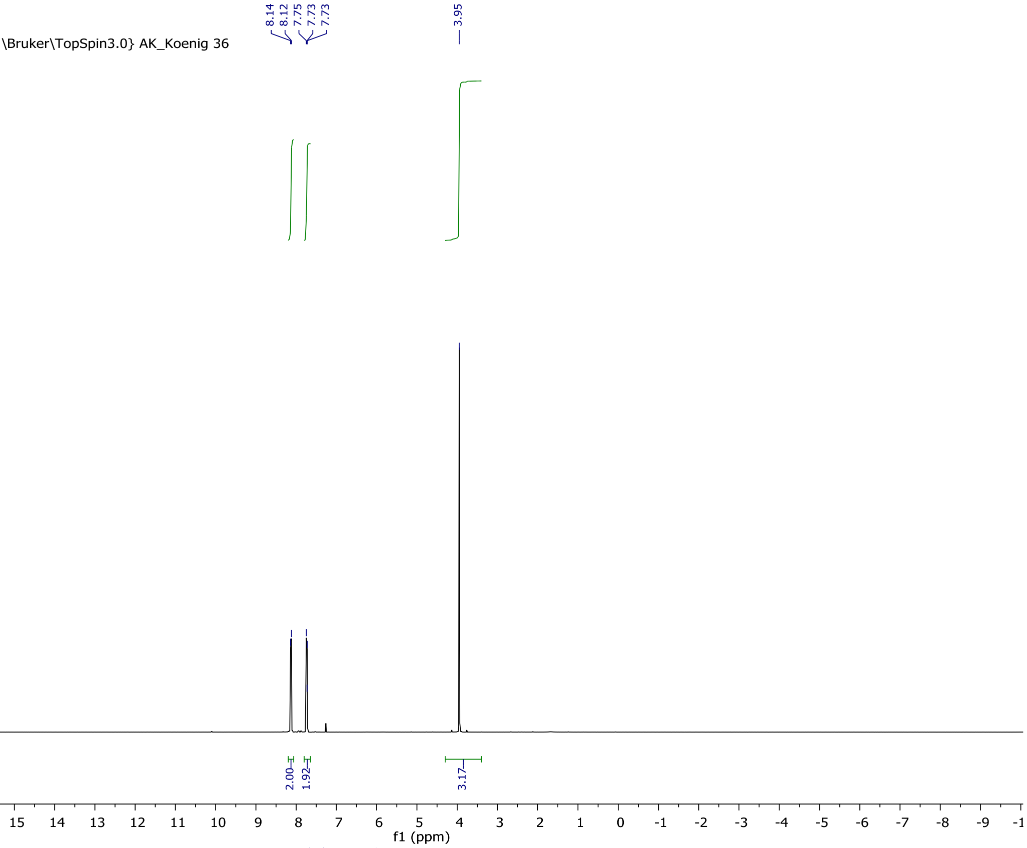

Jun26-2020.41.fid
KM05-284
rau_sC13CPD_256 CDCl3 {C:\Bruker\TopSpin3.0} AK_Koenig 36

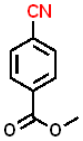

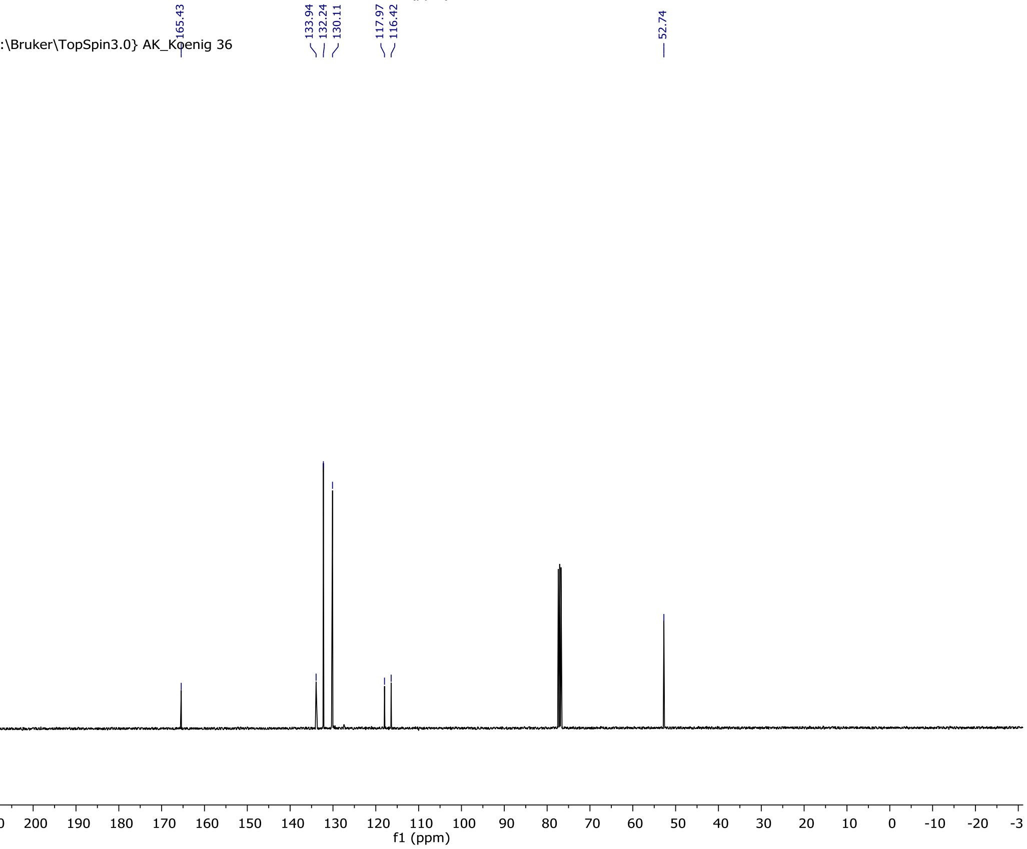

Jun26-2020.60.fid
KM05-392
rau_sPROTON_16 CDCl3 {C:\Bruker\TopSpin3.0} AK_Koenig 38

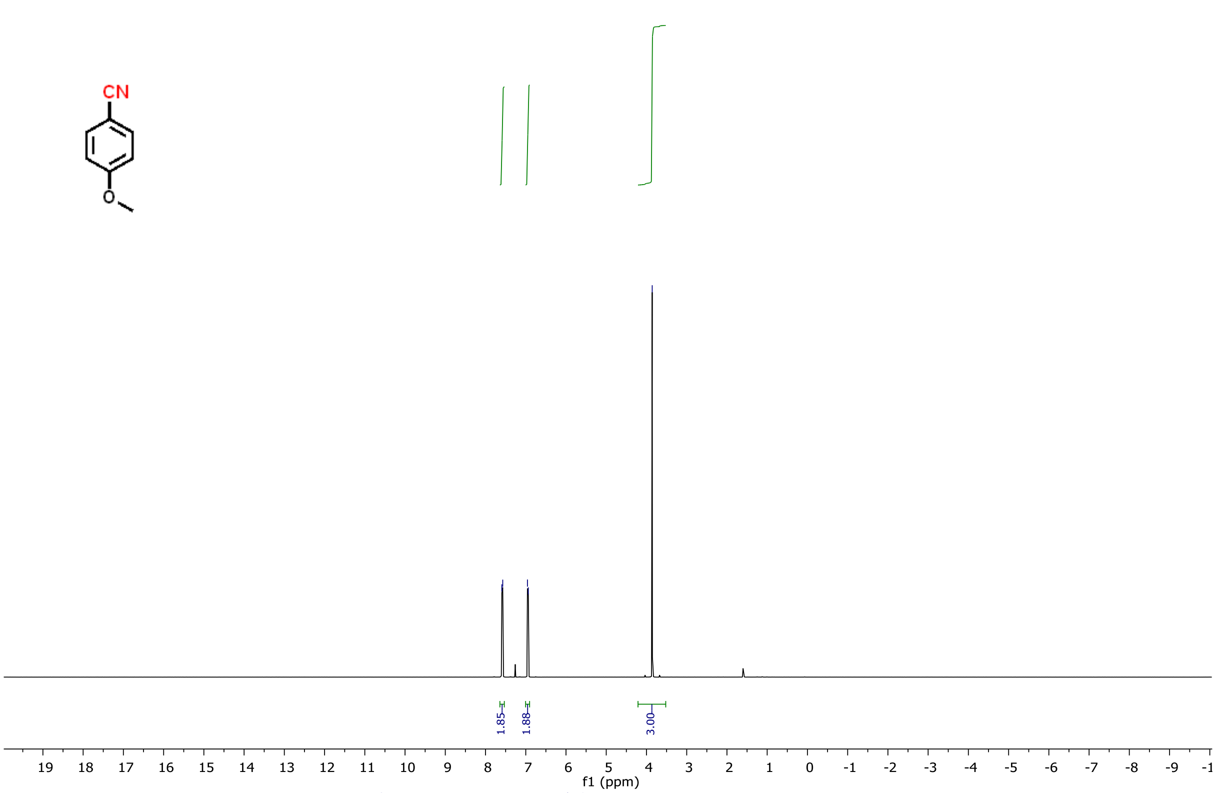

Jun26-2020.61.fid
KM05-392
rau_sC13CPD_256 CDCl3 {C:\Bruker\TopSpin3.0} AK_Koenig 38

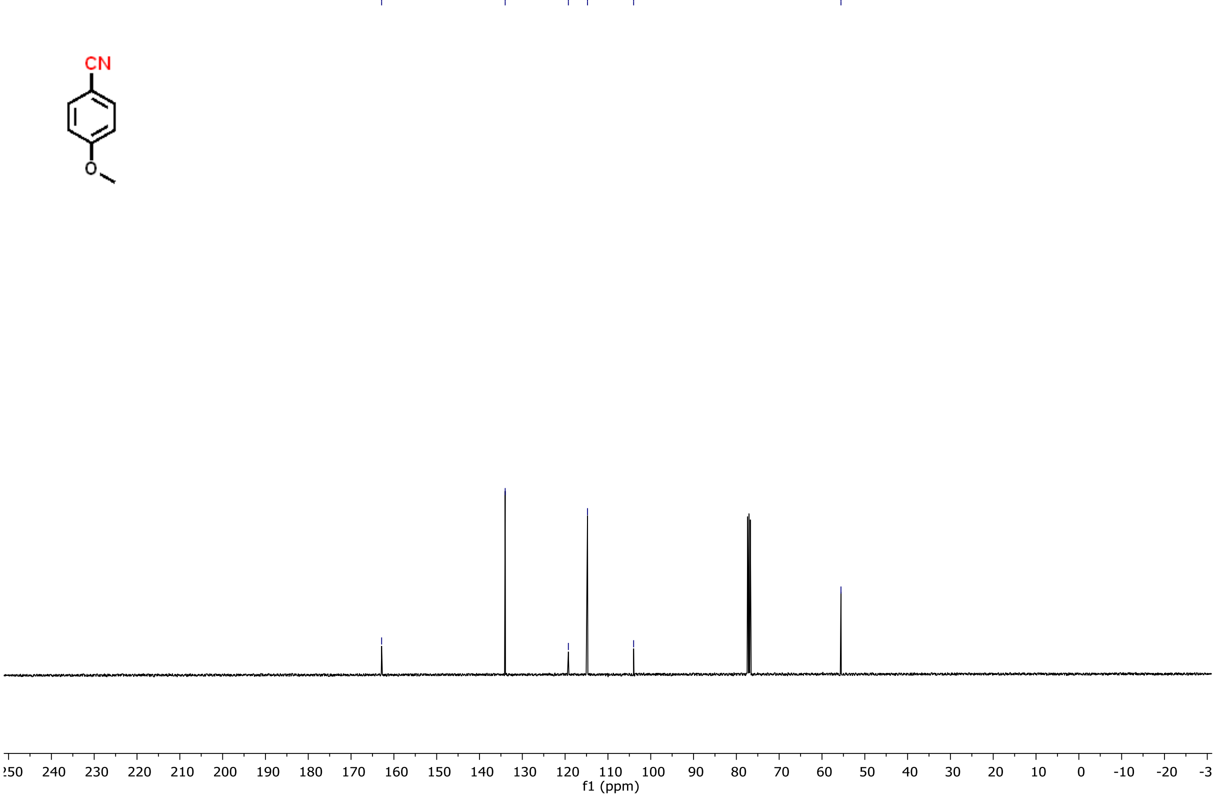

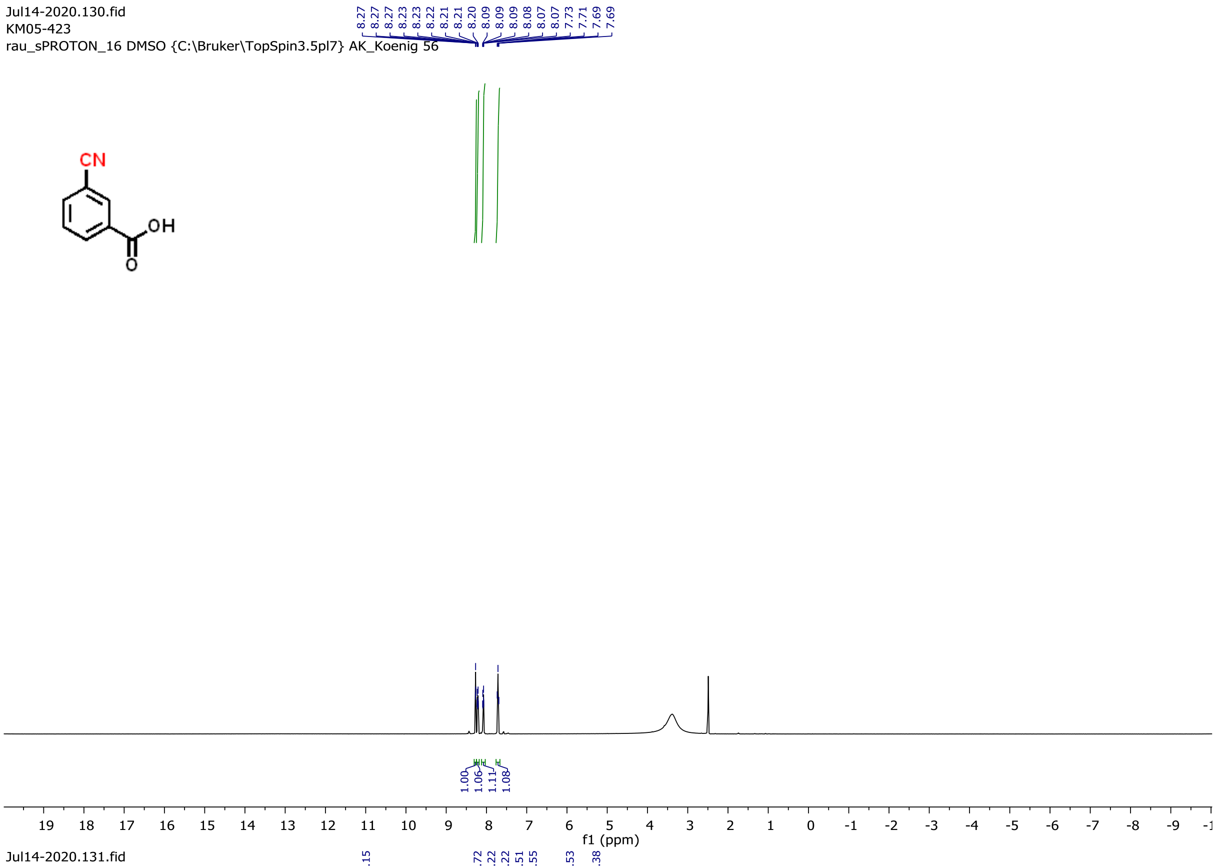
Jul14-2020.130.fid
KM05-423
rau_sPROTON_16 DMSO {C:\Bruker\TopSpin3.5pl7} AK_Koenig 56
8.27
8.27
8.27
8.23
8.23
8.22
8.21
8.21
8.20
8.09
8.09
8.09
8.08
8.07
8.07
7.73
7.71
7.69
7.69
CN
OH
O
1.00
1.06
1.11
1.08
19 18 17 16 15 14 13 12 11 10 9 8 7 6 5 4 3 2 1 0 -1 -2 -3 -4 -5 -6 -7 -8 -9
f1 (ppm)

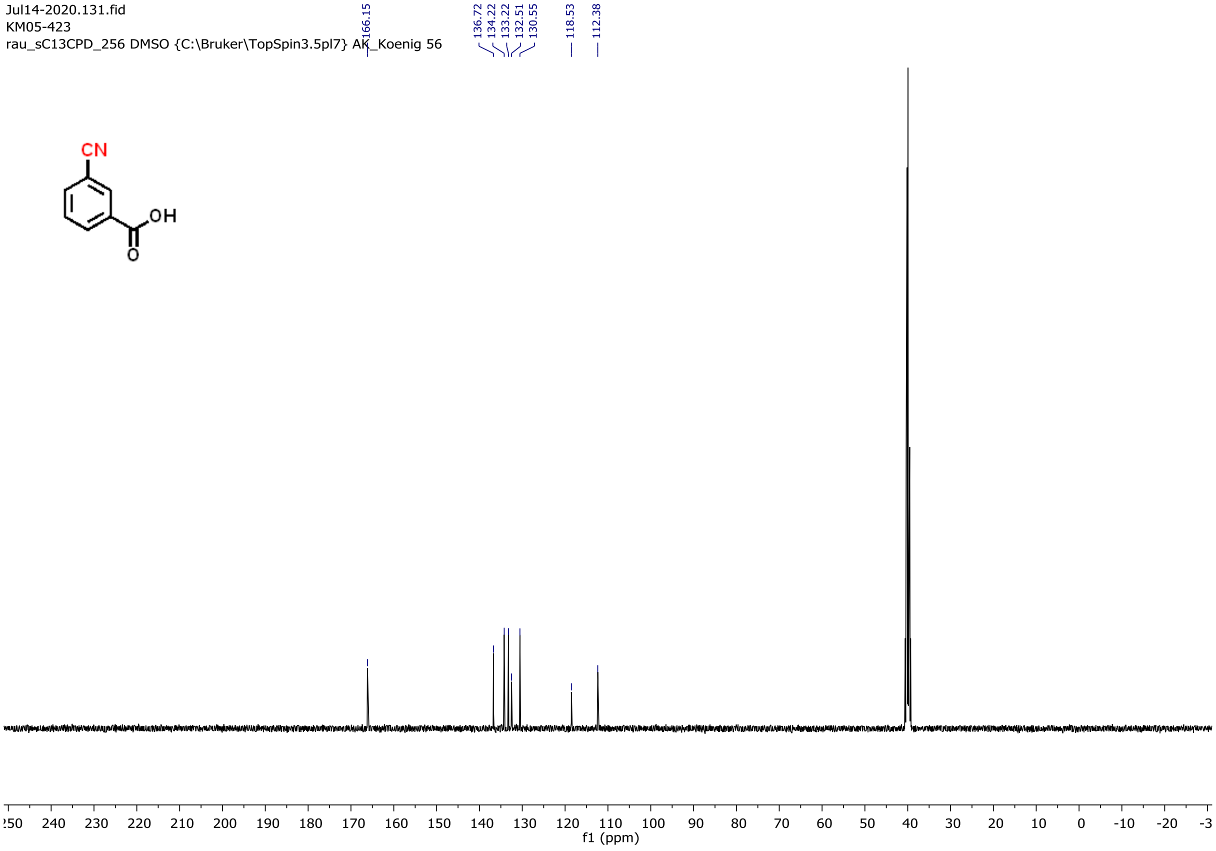
Jul14-2020.131.fid
KM05-423
rau_sC13CPD_256 DMSO {C:\Bruker\TopSpin3.5pl7} AK_Koenig 56
166.15
136.72
134.22
133.22
132.51
130.55
118.53
112.38
CN
OH
O
250 240 230 220 210 200 190 180 170 160 150 140 130 120 110 100 90 80 70 60 50 40 30 20 10 0 -10 -20 -30
f1 (ppm)

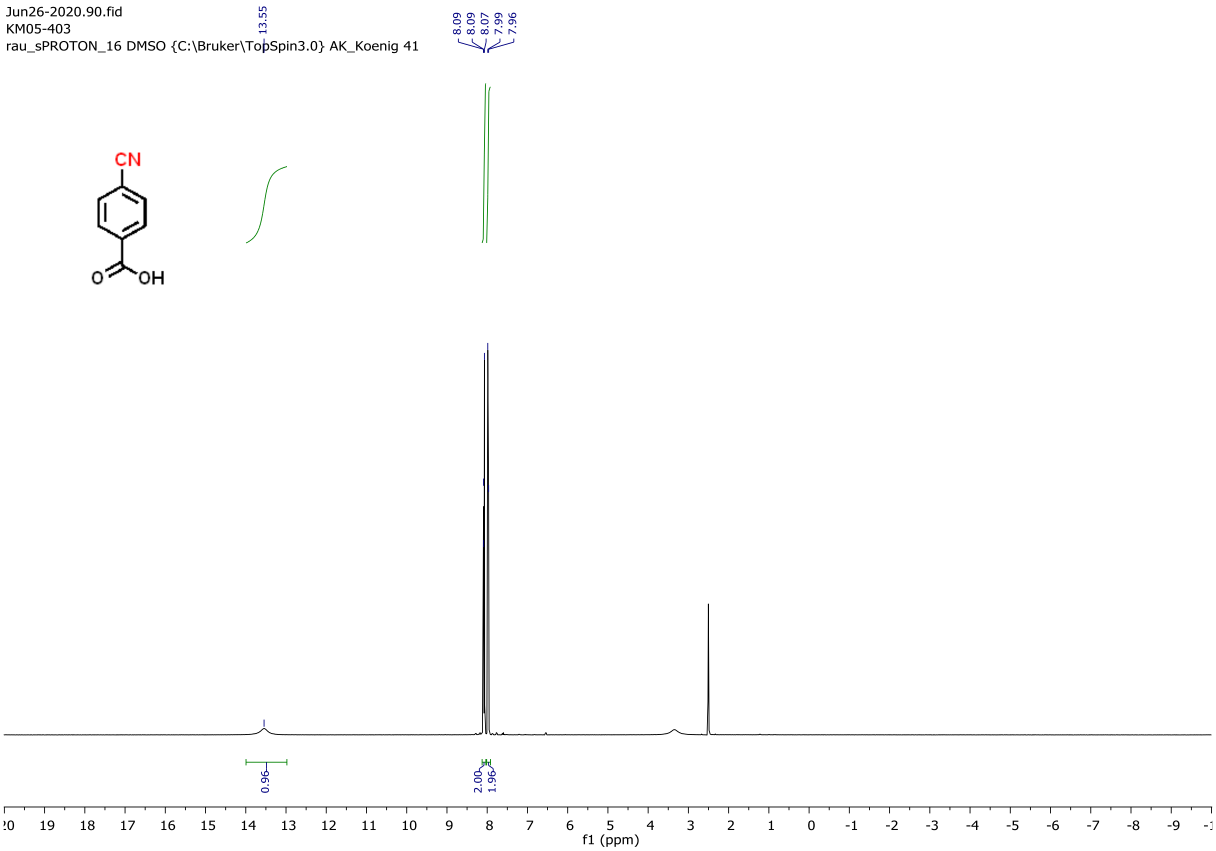

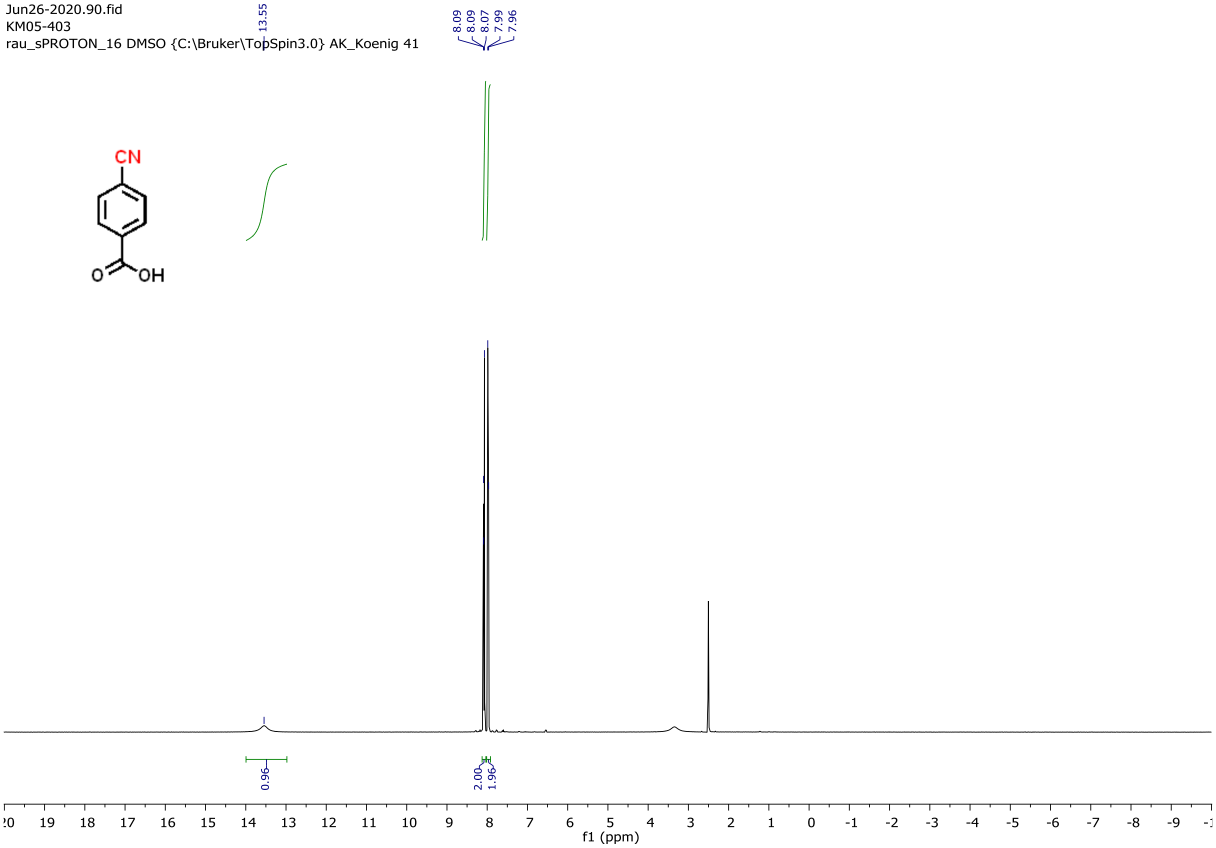
Jun26-2020.90.fid
KM05-403
rau_sPROTON_16 DMSO {C:\Bruker\TopSpin3.0} AK_Koenig 41
13.55
8.09
8.09
8.07
7.99
7.96
CN
O
OH
0.96
2.00
1.96
f1 (ppm)

Jul27-2020.60.fid
KM05-434A
rau_sPROTON_16 DMSO {C:\Bruker\TopSpin3.5pl7} AK_Koenig 17

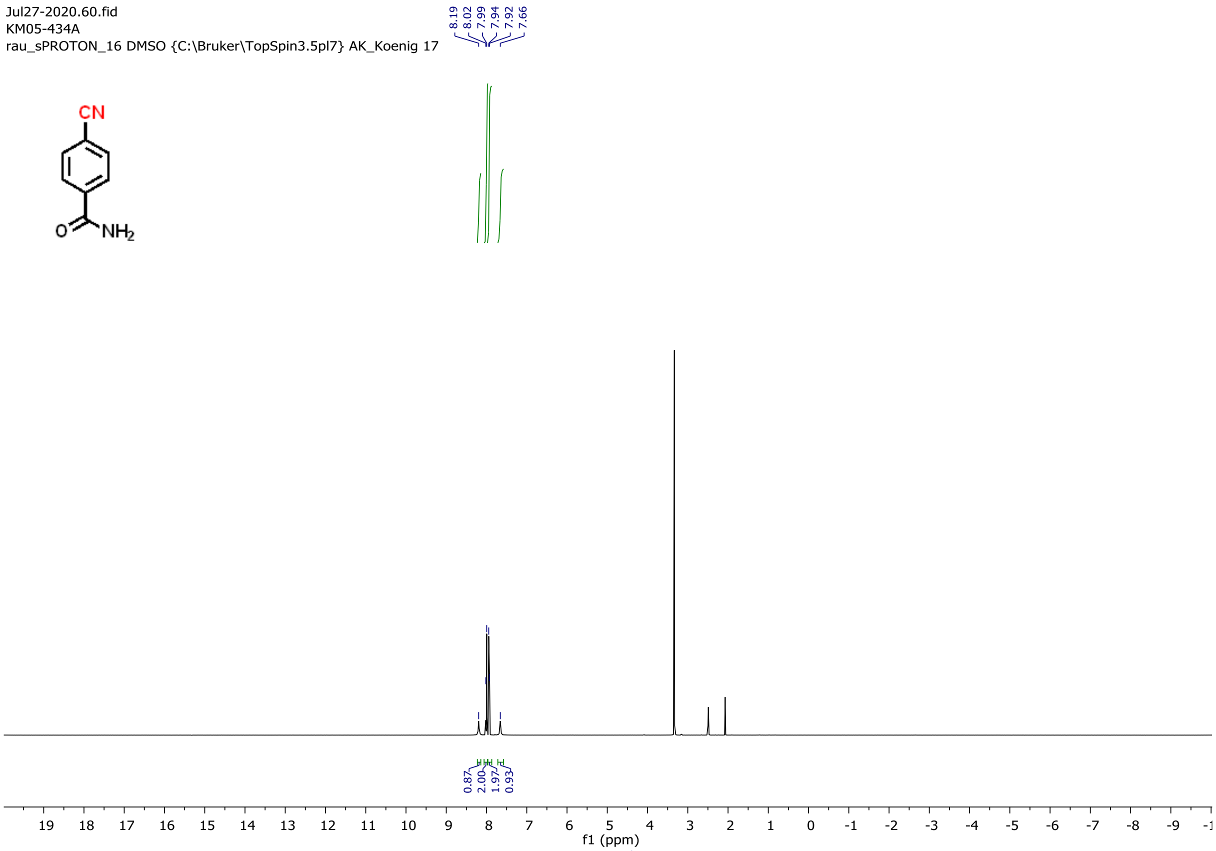

Jul27-2020.61.fid
KM05-434A
rau_sC13CPD_256 DMSO {C:\Bruker\TopSpin3.5pl7} AK_Koenig 17

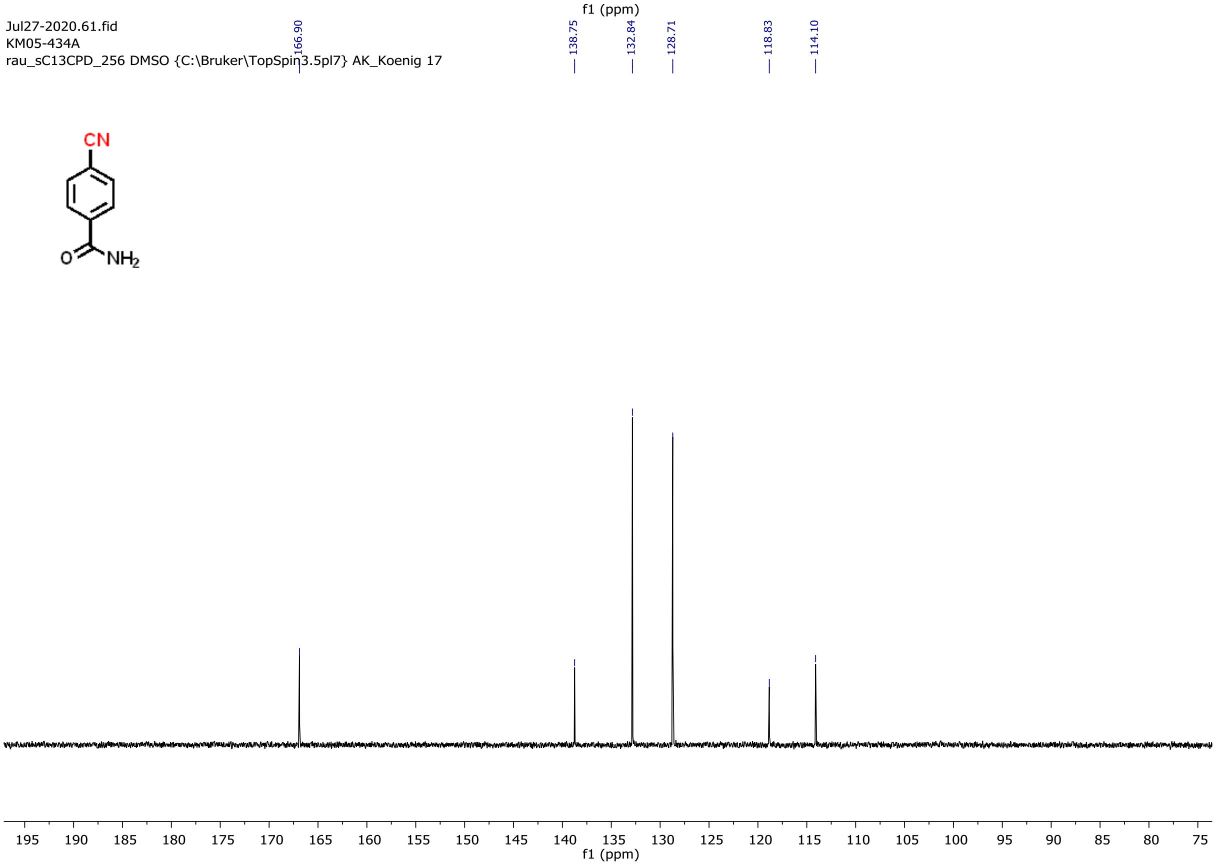

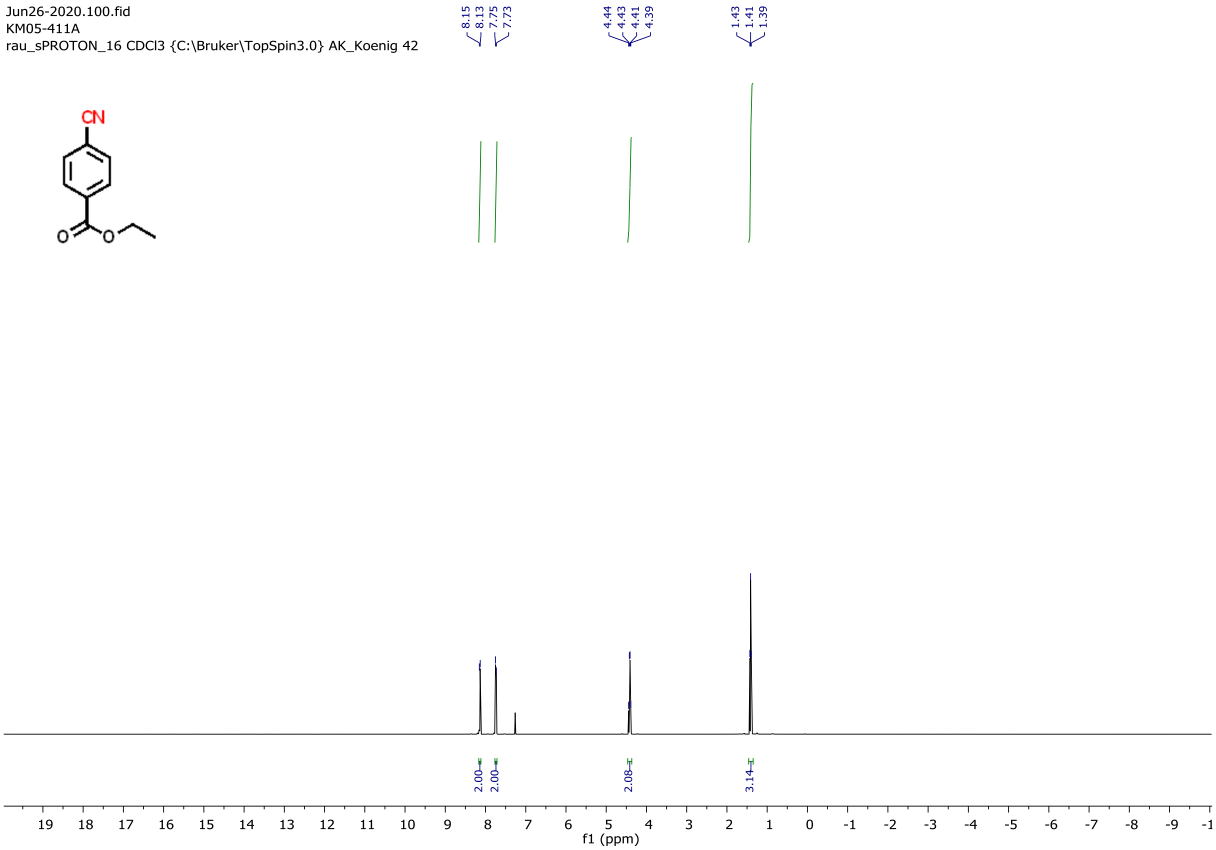
Jun26-2020.100.fid
KM05-411A
rau_sPROTON_16 CDCl3 {C:\Bruker\TopSpin3.0} AK_Koenig 42
8.15
8.13
7.75
7.73
4.44
4.43
4.41
4.39
1.43
1.41
1.39
CN
O
O
2.00
2.00
2.08
3.14
19 18 17 16 15 14 13 12 11 10 9 8 7 6 5 4 3 2 1 0 -1 -2 -3 -4 -5 -6 -7 -8 -9
f1 (ppm)

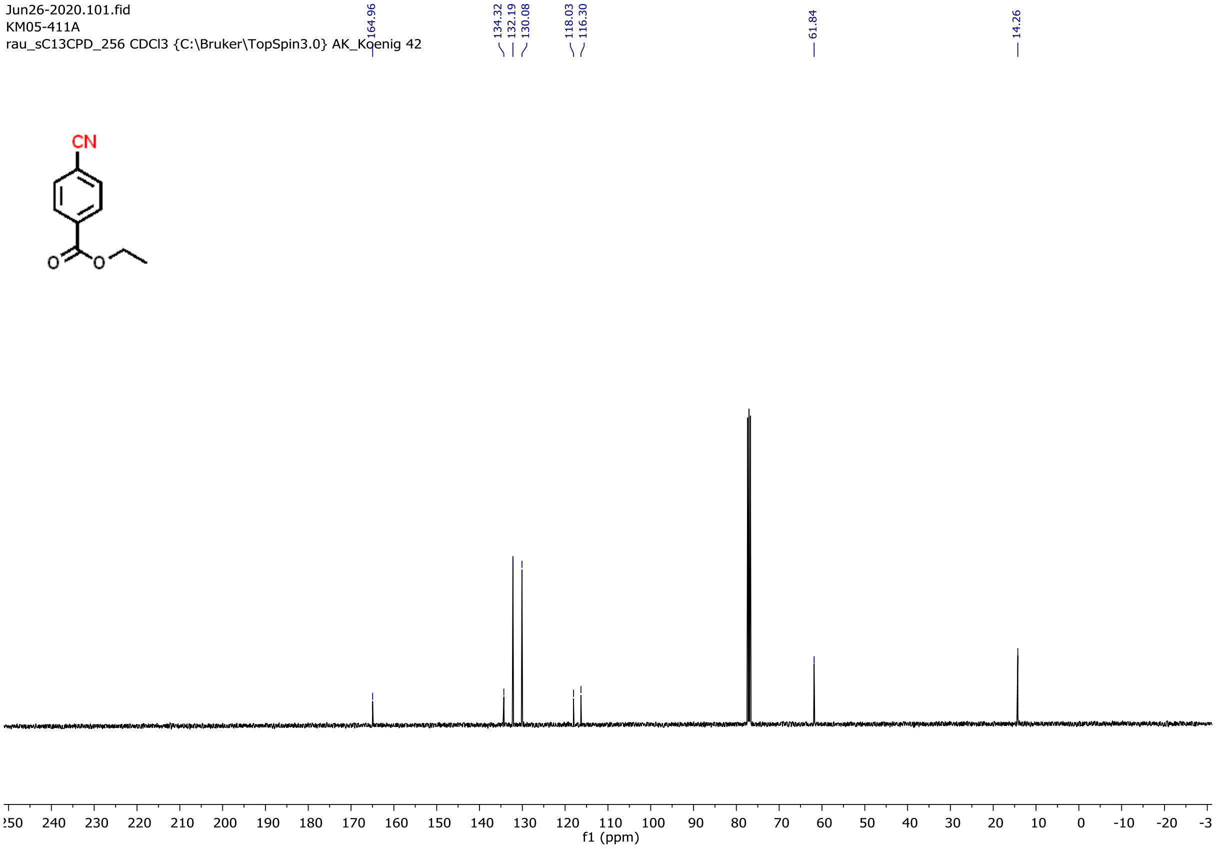
Jun26-2020.101.fid
KM05-411A
rau_sC13CPD_256 CDCl3 {C:\Bruker\TopSpin3.0} AK_Koenig 42
164.96
134.32
132.19
130.08
118.03
116.30
61.84
14.26
CN
O
O
250 240 230 220 210 200 190 180 170 160 150 140 130 120 110 100 90 80 70 60 50 40 30 20 10 0 -10 -20
f1 (ppm)

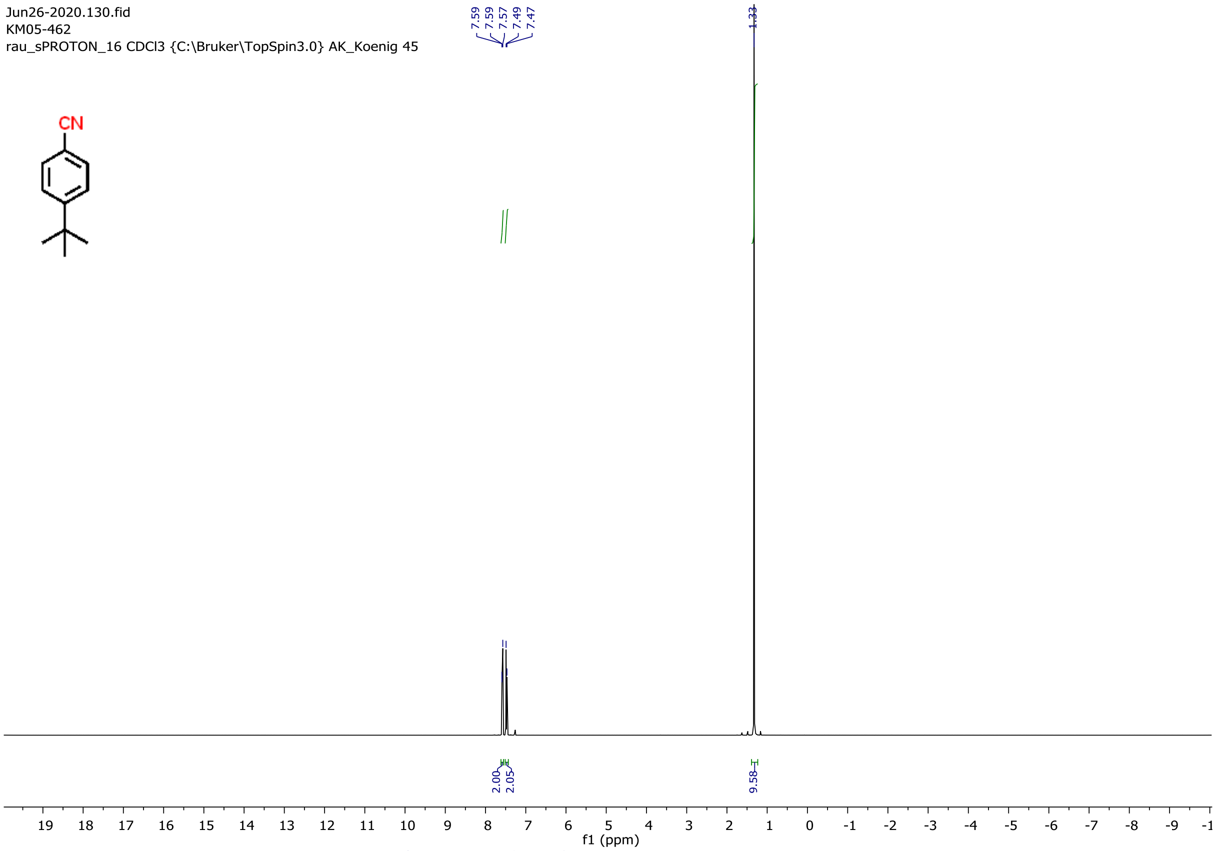
Jun26-2020.130.fid
KM05-462
rau_sPROTON_16 CDCl3 {C:\Bruker\TopSpin3.0} AK_Koenig 45
CN
7.59
7.59
7.57
7.49
7.47
1.33
2.00
2.05
9.58
f1 (ppm)

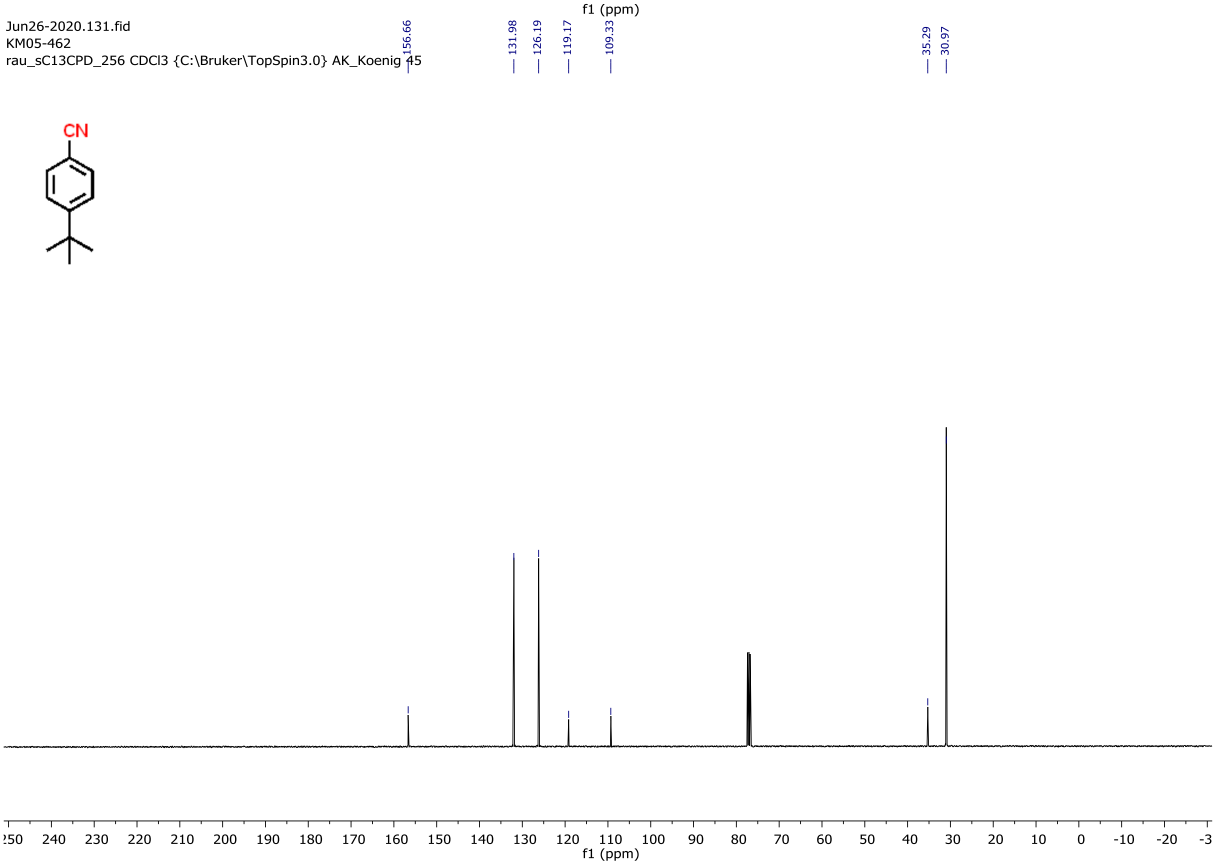
Jun26-2020.131.fid
KM05-462
rau_sC13CPD_256 CDCl3 {C:\Bruker\TopSpin3.0} AK_Koenig 45
CN
156.66
131.98
126.19
119.17
109.33
35.29
30.97
f1 (ppm)

Jun26-2020.200.fid
KM05-524
rau_sPROTON_16 CDCl3 {C:\Bruker\TopSpin3.0} AK_Koenig 52

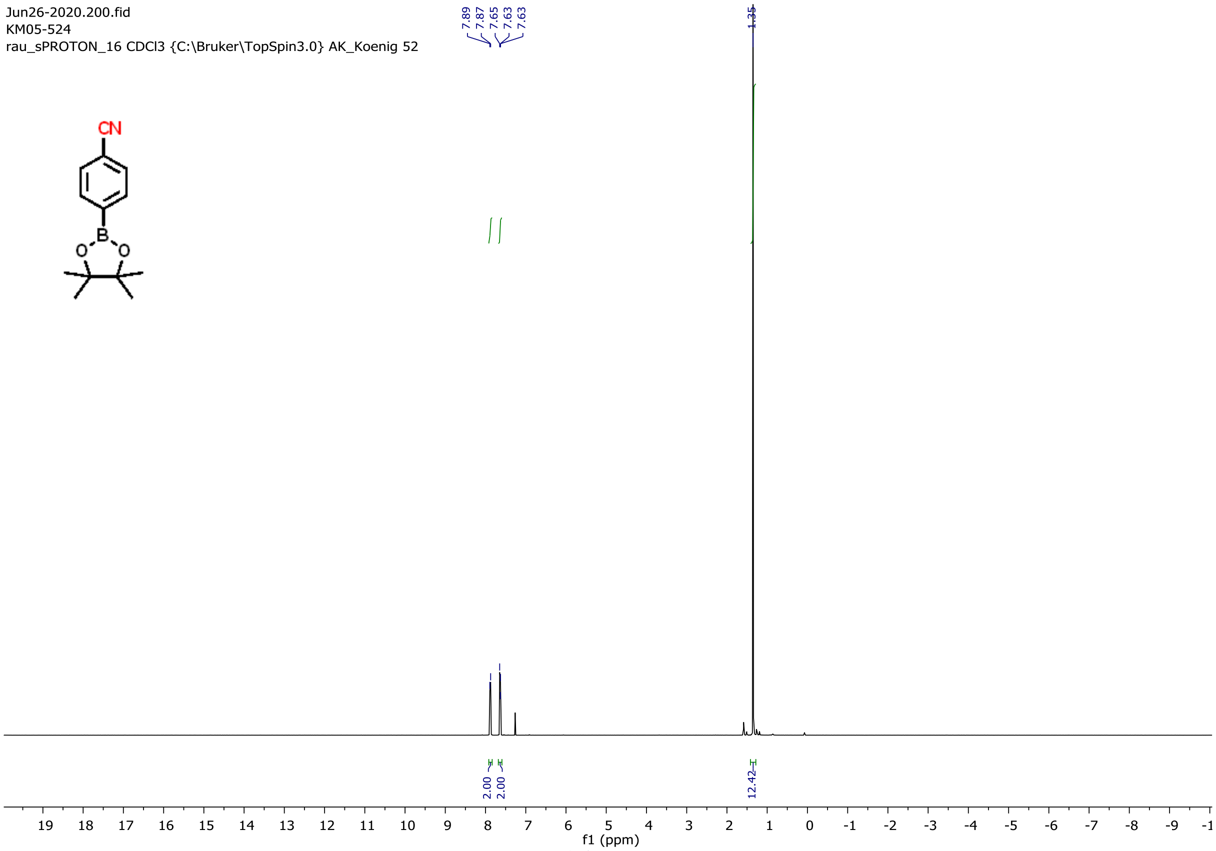

Jun26-2020.201.fid
KM05-524
rau_sC13CPD_256 CDCl3 {C:\Bruker\TopSpin3.0} AK_Koenig 52

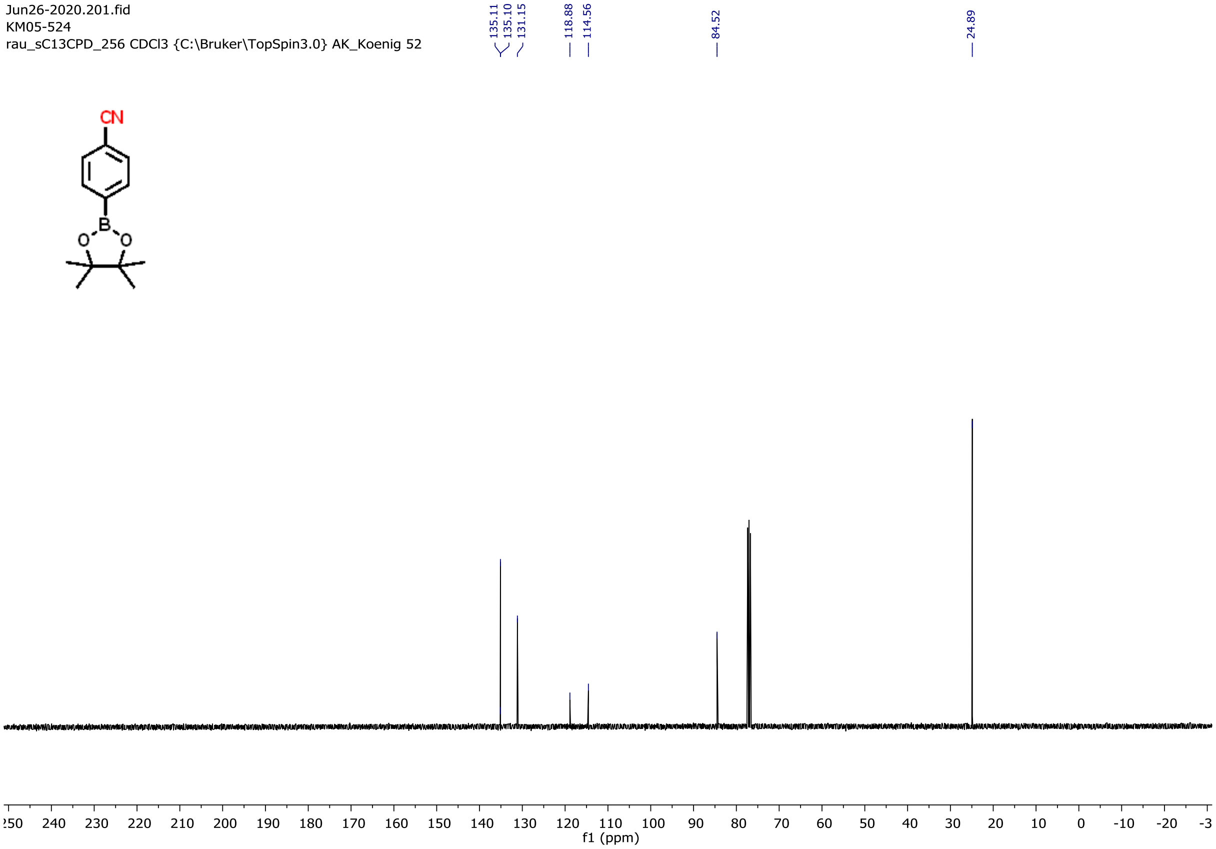

Jun26-2020.150.fid
KM05-482
rau_sPROTON_16 CDCl3 {C:\Bruker\TopSpin3.0} AK_Koenig 47

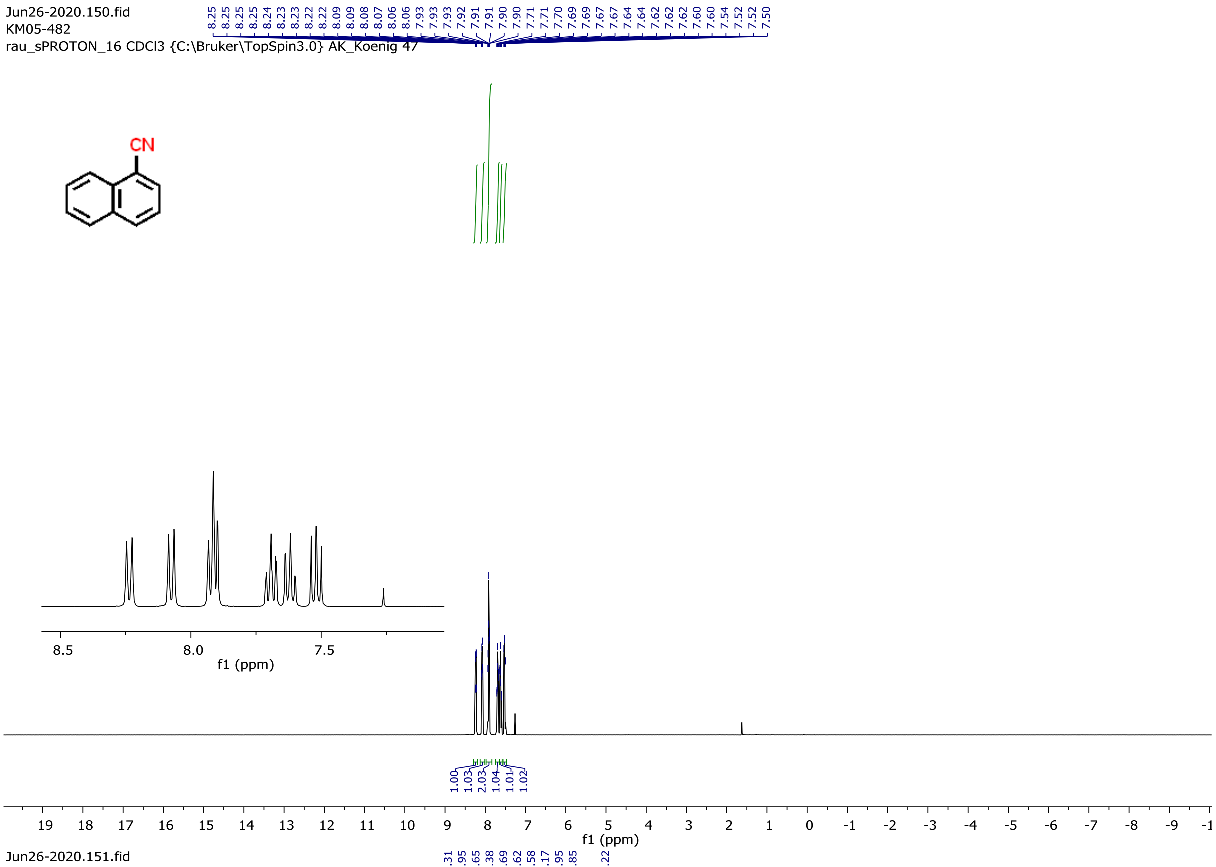

Jun26-2020.151.fid
KM05-482
rau_sC13CPD_256 CDCl3 {C:\Bruker\TopSpin3.0} AK_Koenig 47

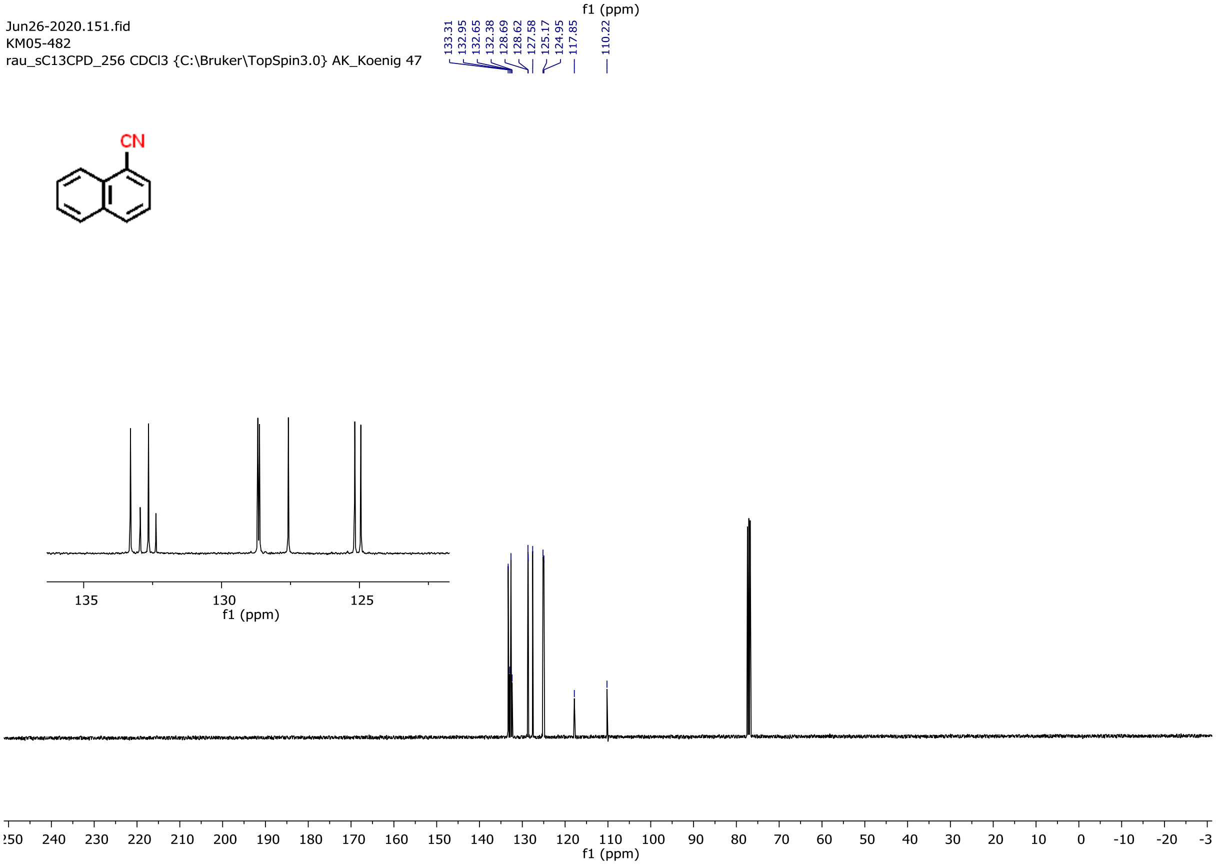

Jun26-2020.50.fid
KM05-306
rau_sPROTON_16 CDCl3 {C:\Bruker\TopSpin3.0} AK_Koenig 37

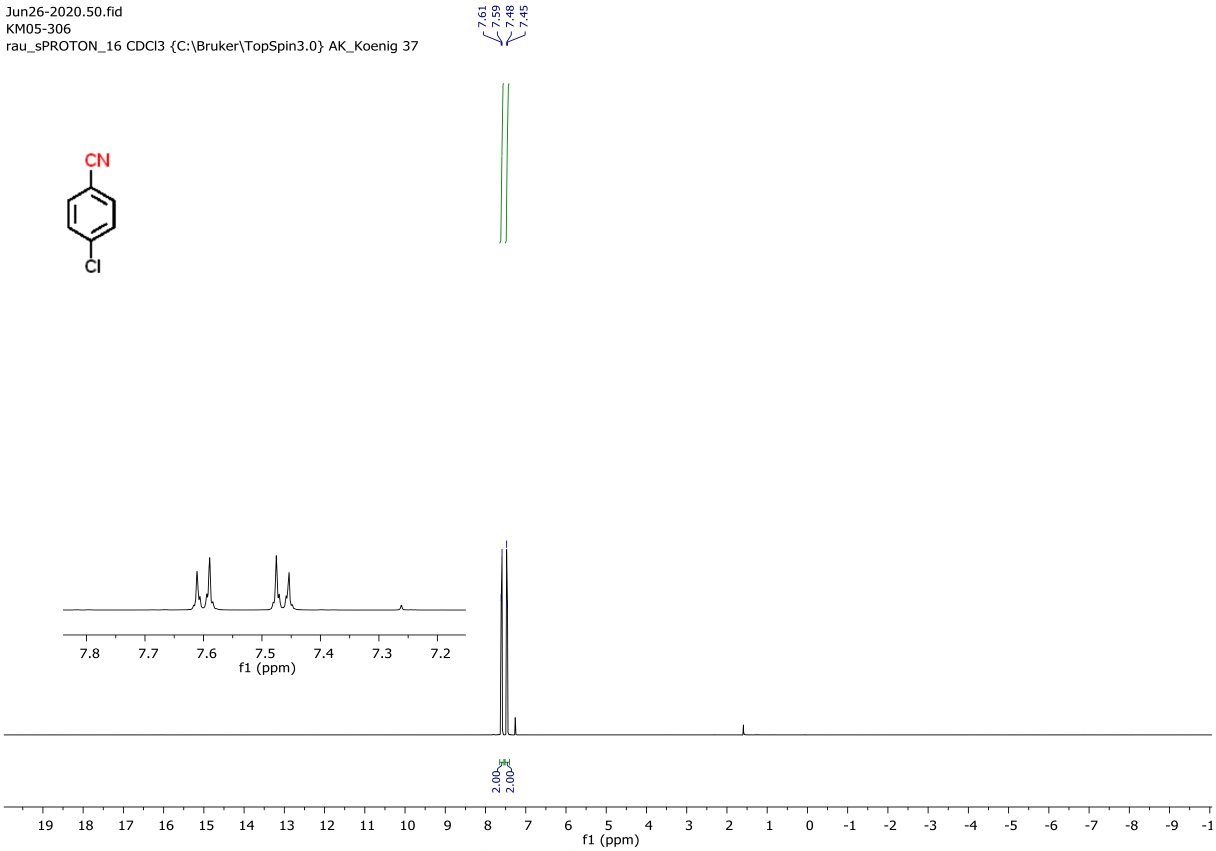

Jun26-2020.51.fid
KM05-306
rau_sC13CPD_256 CDCl3 {C:\Bruker\TopSpin3.0} AK_Koenig 37

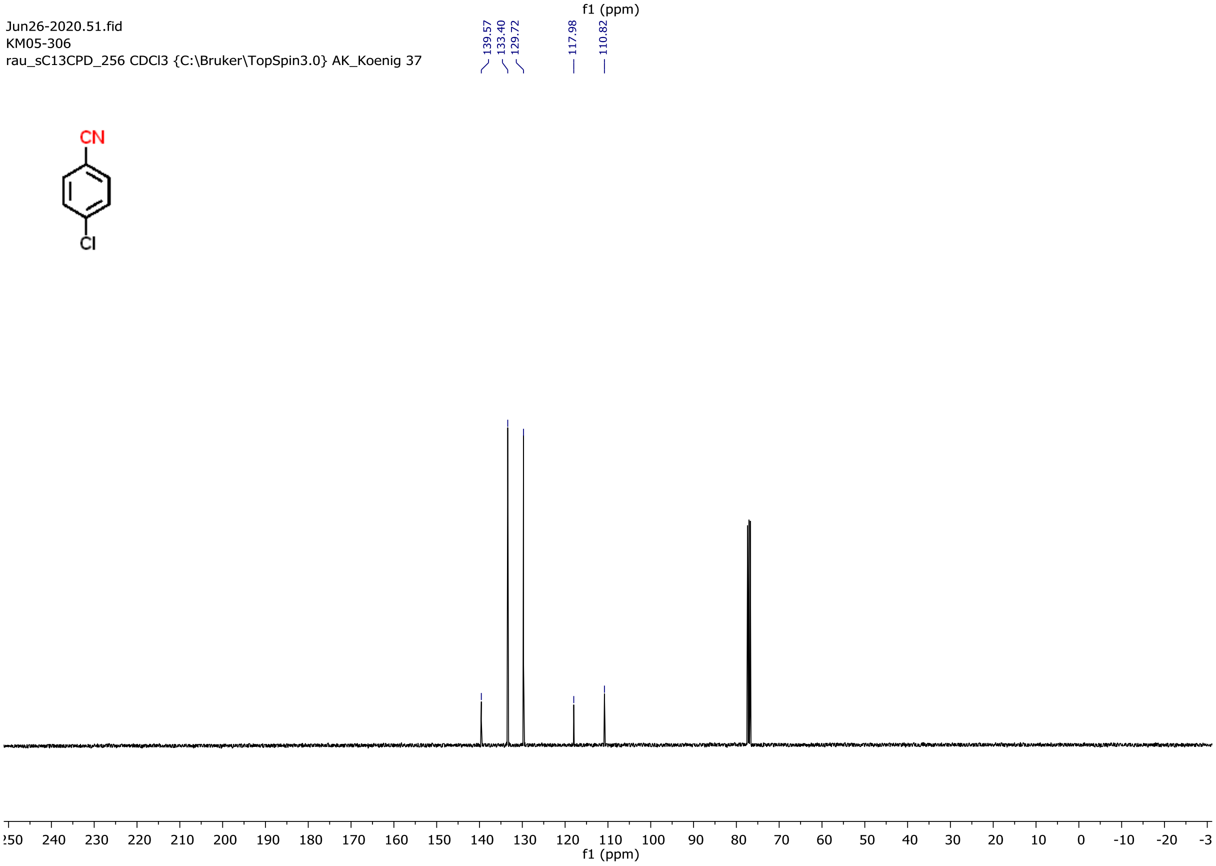

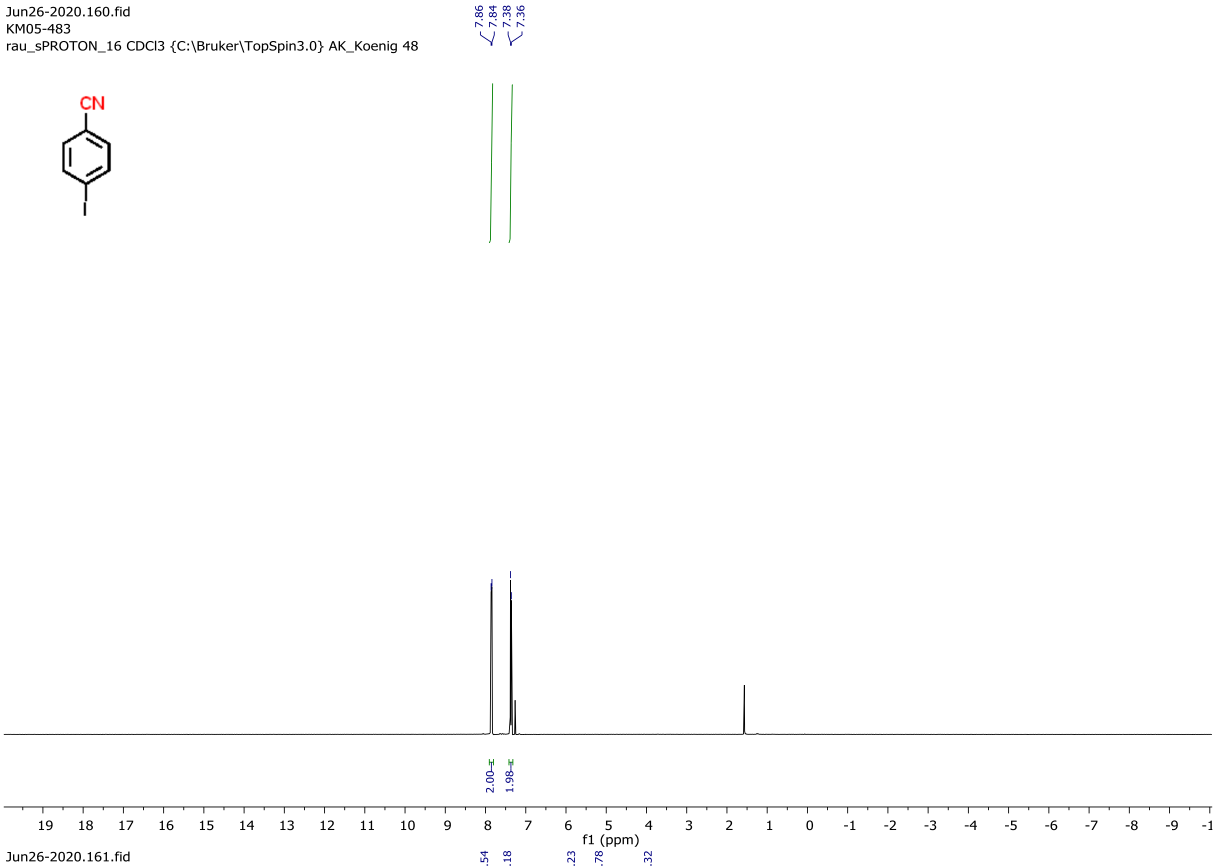
Jun26-2020.160.fid
KM05-483
rau_sPROTON_16 CDCl3 {C:\Bruker\TopSpin3.0} AK_Koenig 48
CN
I
7.86
7.84
7.38
7.36
2.00
1.98
f1 (ppm)

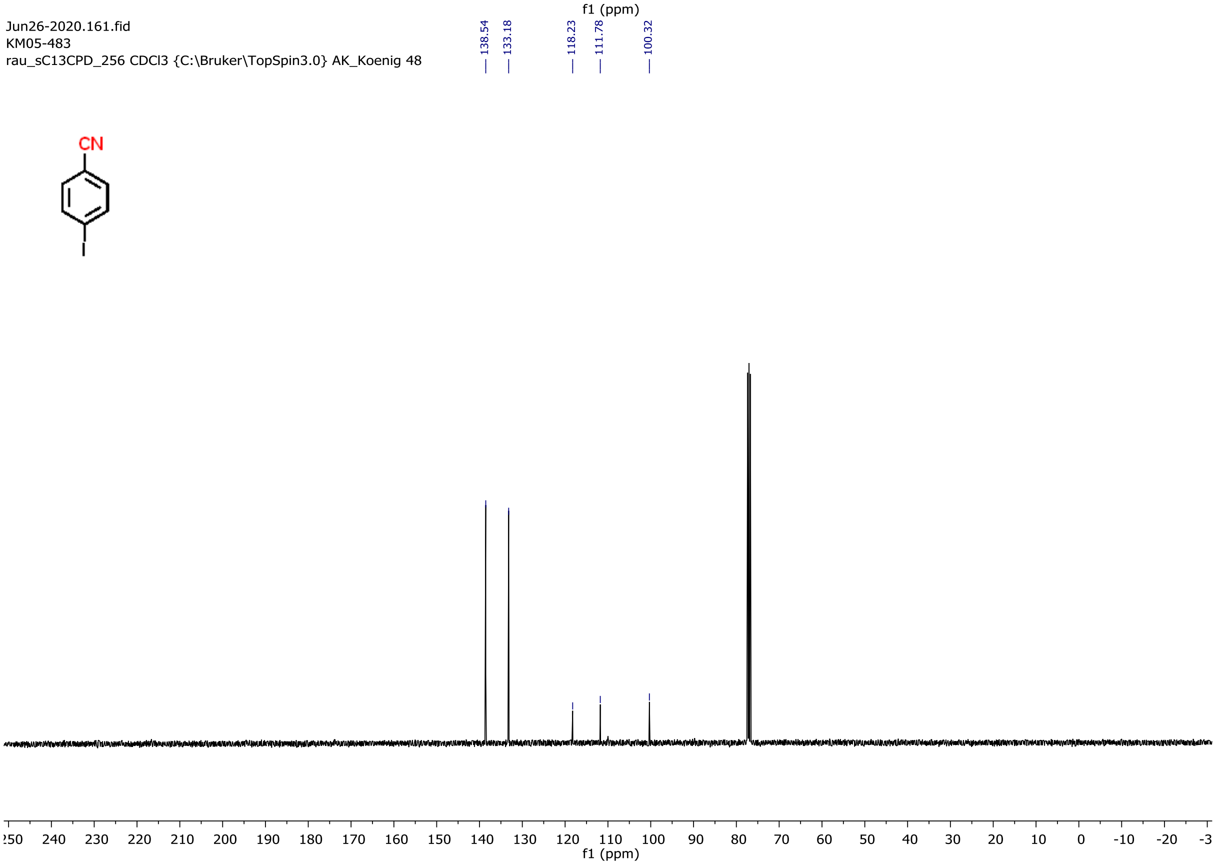
Jun26-2020.161.fid
KM05-483
rau_sC13CPD_256 CDCl3 {C:\Bruker\TopSpin3.0} AK_Koenig 48
138.54
133.18
118.23
111.78
100.32
CN
I
f1 (ppm)

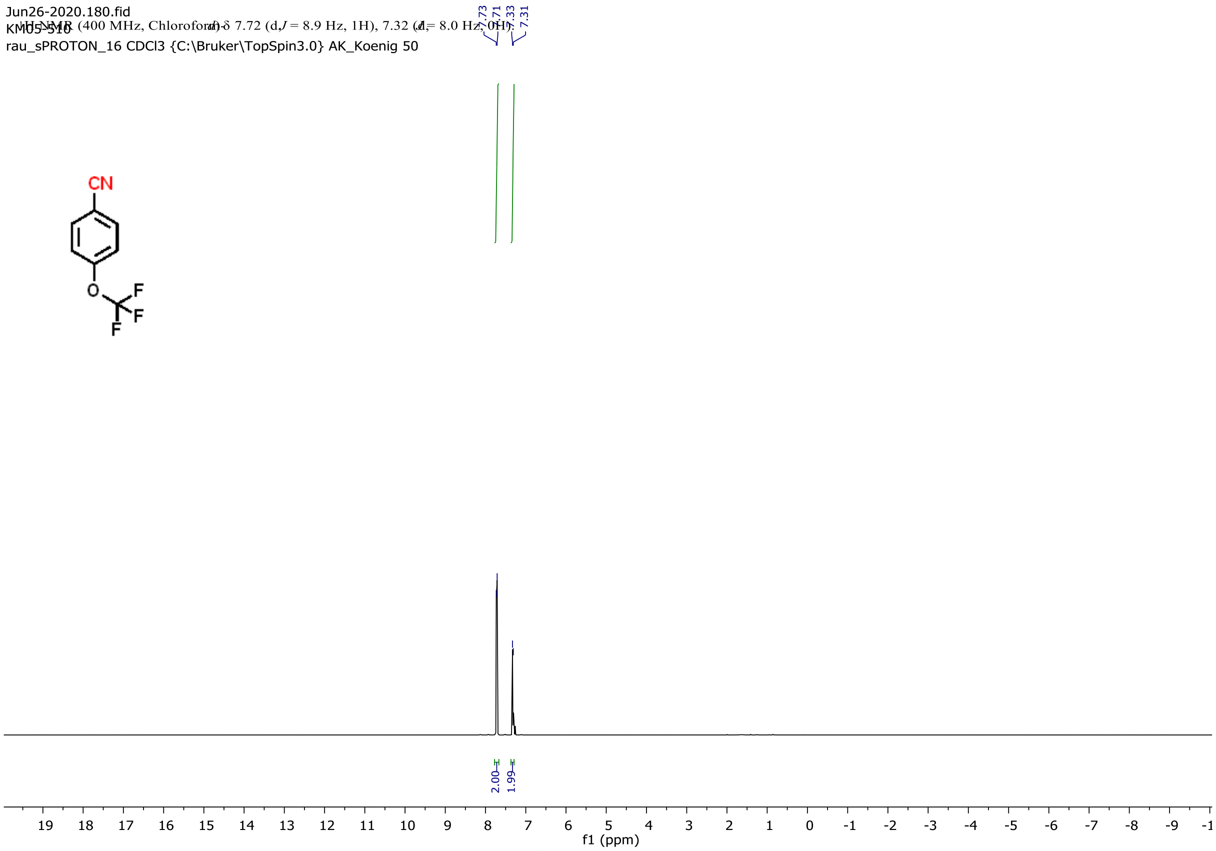
Jun26-2020.180.fid
KM05-510
1H NMR (400 MHz, Chloroform-d) δ 7.72 (d, J = 8.9 Hz, 1H), 7.32 (d, J = 8.0 Hz, 2H).
rau_sPROTON_16 CDCl3 {C:\Bruker\TopSpin3.0} AK_Koenig 50
7.73
7.71
7.33
7.31
CN
O
F
F
F
2.00
1.99
f1 (ppm)

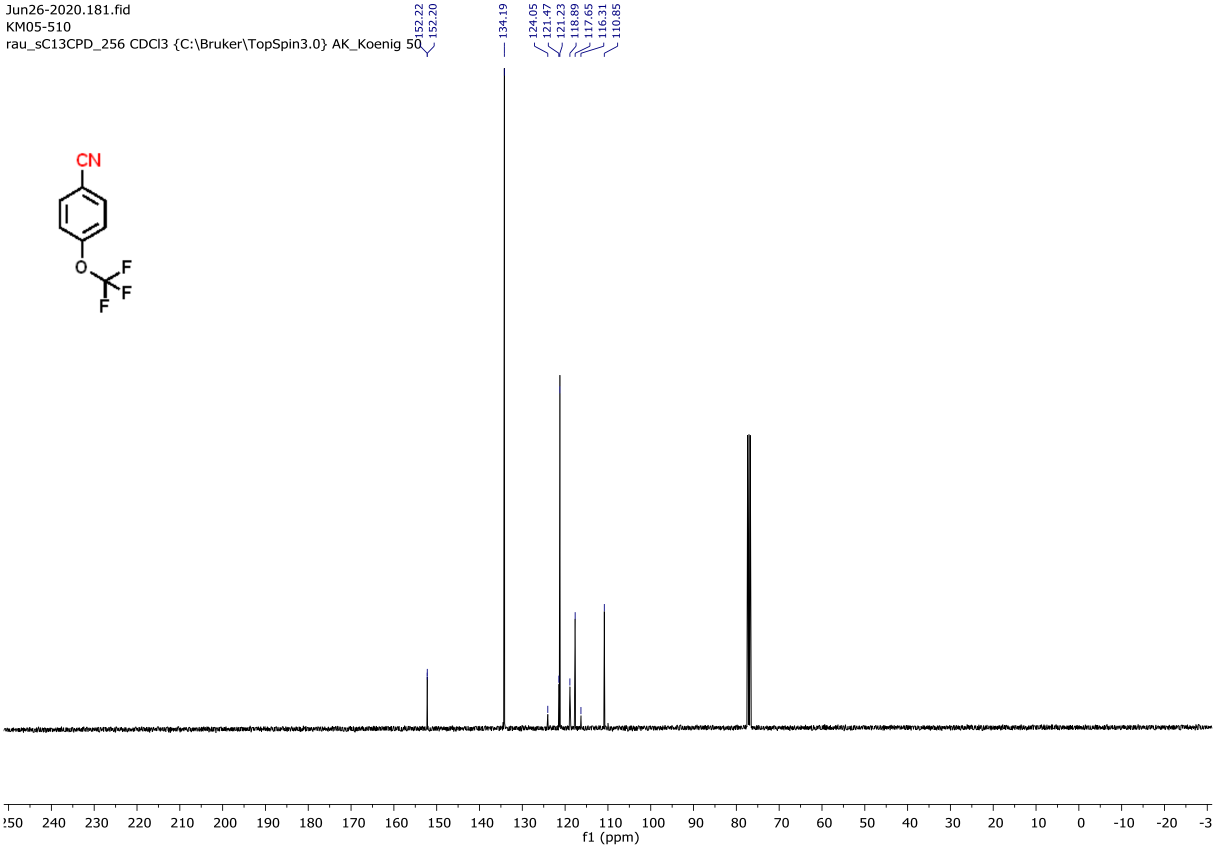
Jun26-2020.181.fid
KM05-510
rau_sC13CPD_256 CDCl3 {C:\Bruker\TopSpin3.0} AK_Koenig 50
152.22
152.20
134.19
124.05
121.47
121.23
118.89
117.65
116.31
110.85
CN
O
F
F
F
f1 (ppm)

Jun26-2020.182.fid
KM05-510
rau_sF19CPD CDCl3 {C:\Bruker\TopSpin3.0} AK_Koenig 50

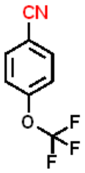

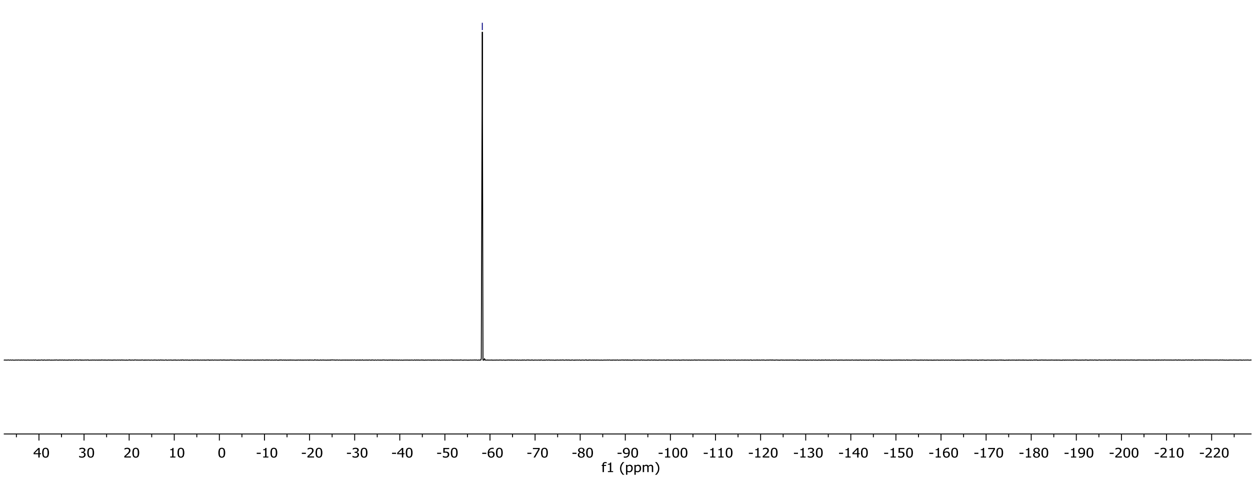

Jul03-2020.40.fid
KM05-419
rau_sPROTON_64 CDCl3 {C:\Bruker\TopSpin3.5pl7} AK_Koenig 48

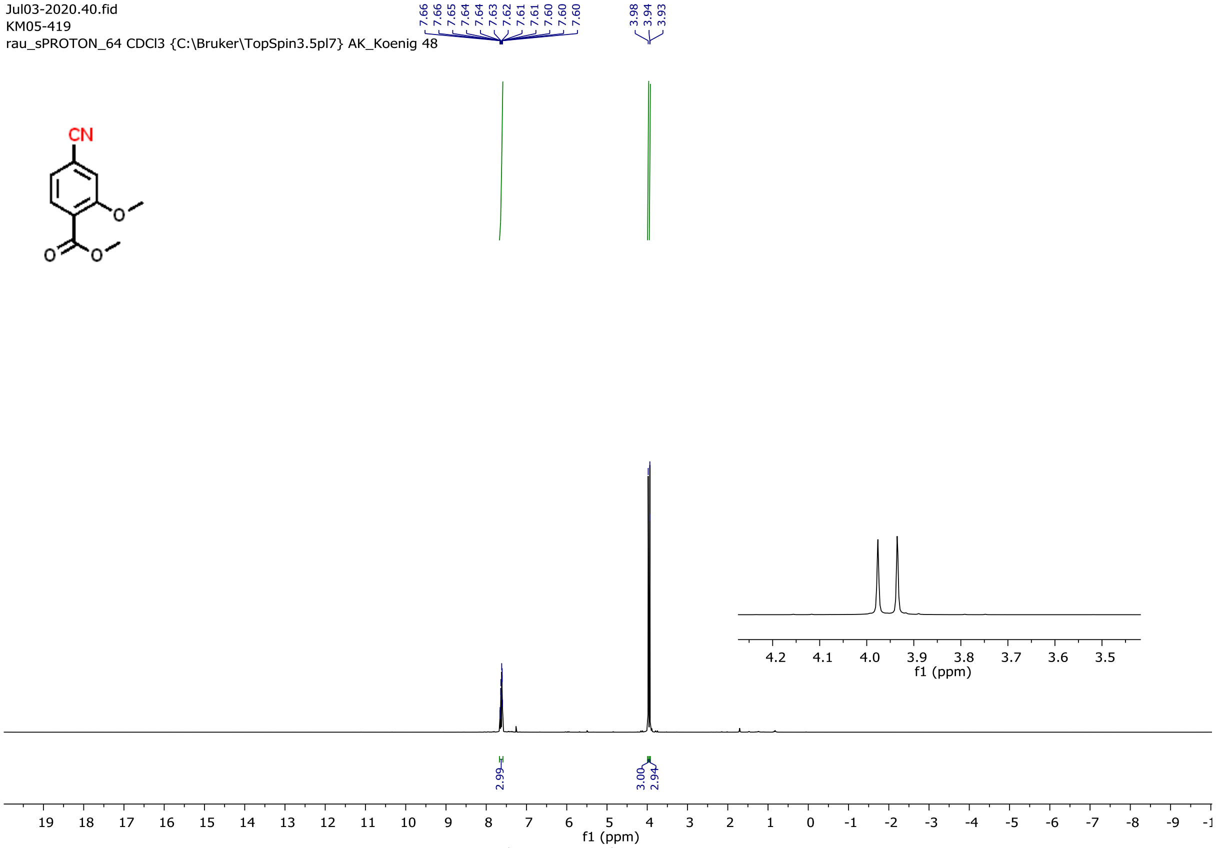

Jul03-2020.41.fid
KM05-419
rau_sC13CPD_256 CDCl3 {C:\Bruker\TopSpin3.5pl7} AK_Koenig 48

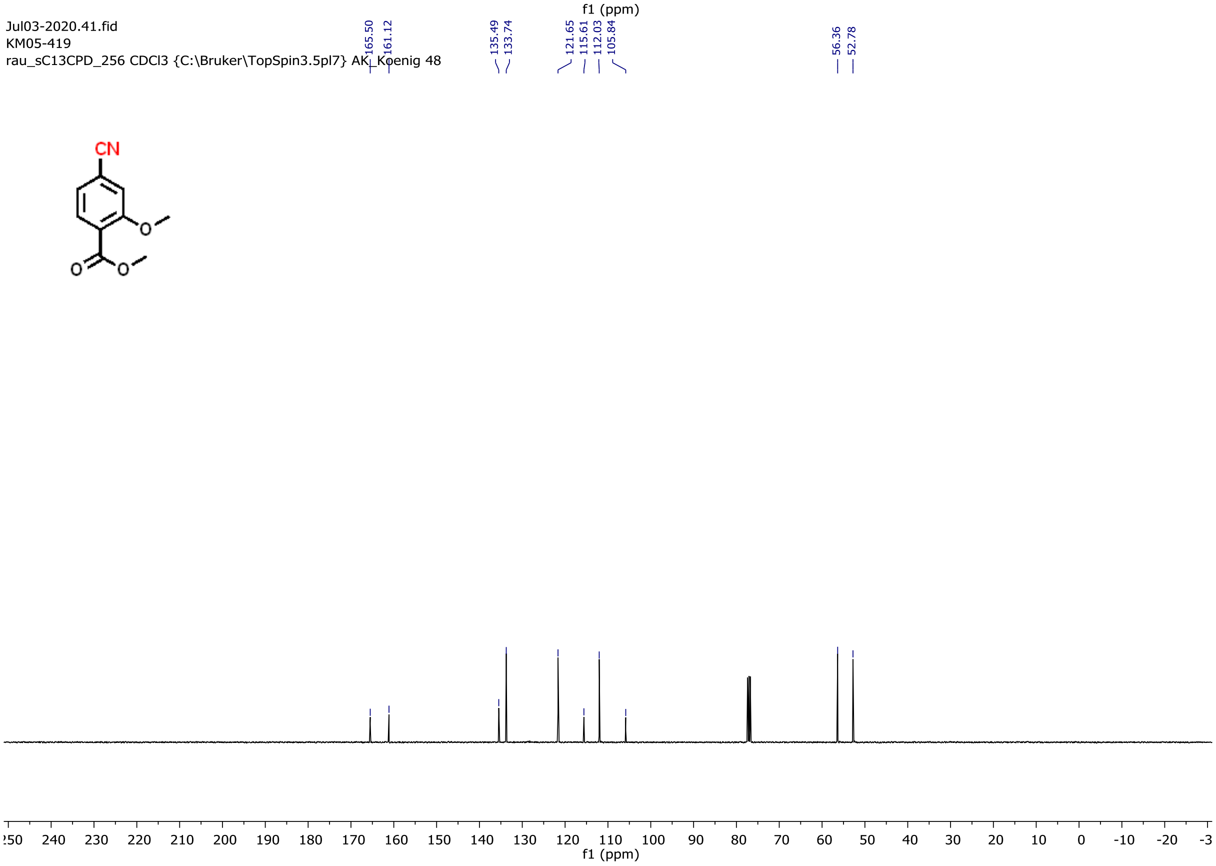

Jun26-2020.170.fid
KM05-488
rau_sPROTON_16 CDCl3 {C:\Bruker\TopSpin3.0} AK_Koenig 49

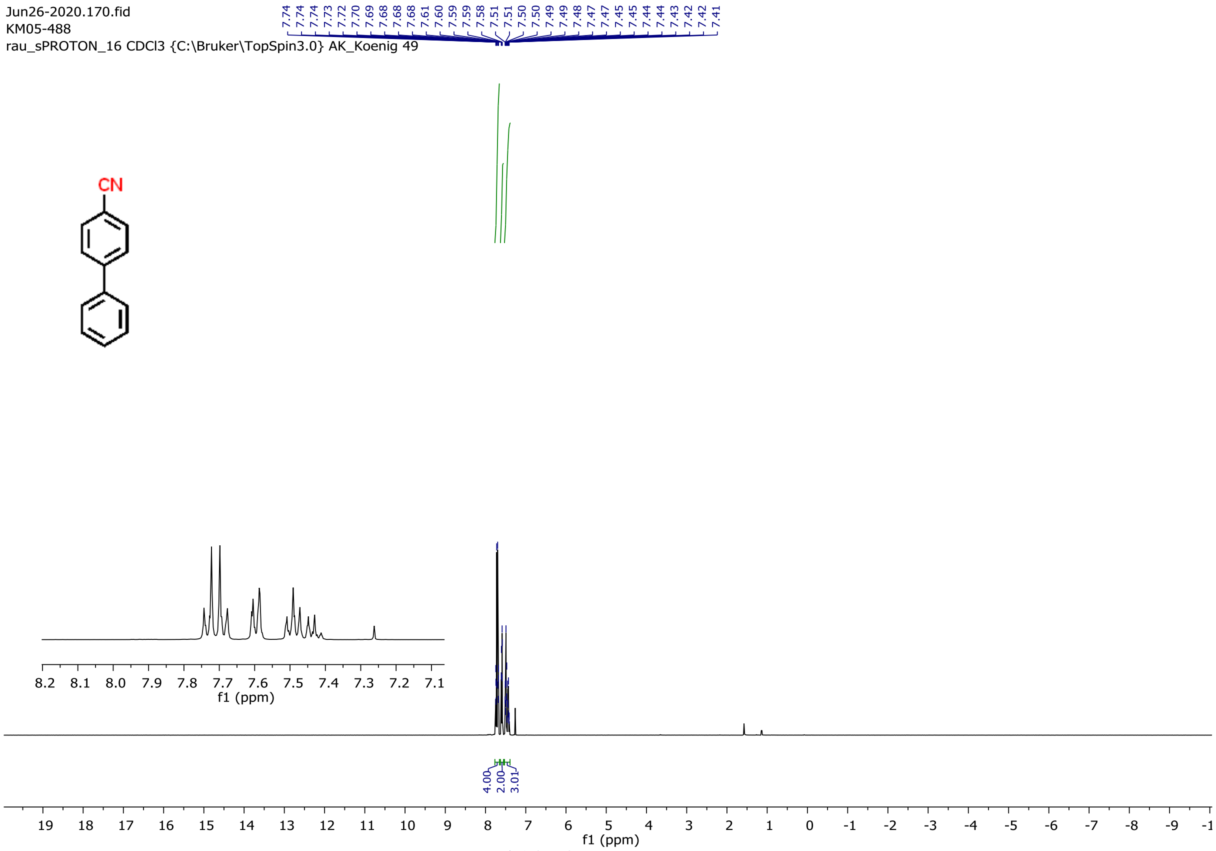

Jun26-2020.171.fid
KM05-488
rau_sC13CPD_256 CDCl3 {C:\Bruker\TopSpin3.0} AK_Koenig 49

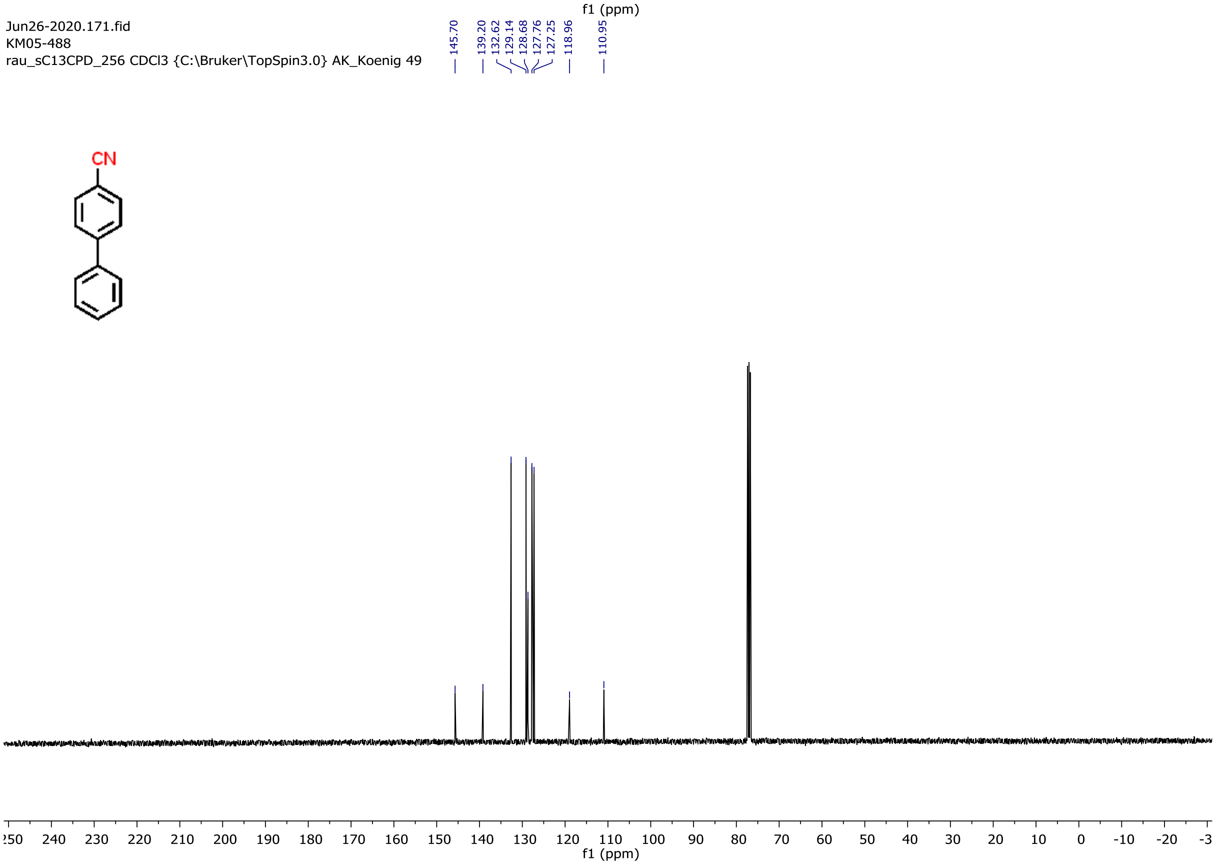

Jun26-2020.190.fid
KM05-523
rau_sPROTON_16 CDCl3 {C:\Bruker\TopSpin3.0} AK_Koenig 51

7.88 7.87 7.87 7.87 7.83 7.82 7.81 7.81 7.80 7.65 7.65 7.64 7.63 7.63 7.62 7.58 7.58 7.57 7.56 7.56 7.55 7.55 7.53 7.51 7.50 7.50 7.49 7.49 7.48 7.47 7.47 7.46 7.44 7.44 7.43 7.43 7.42 7.41 7.41 7.40 7.40

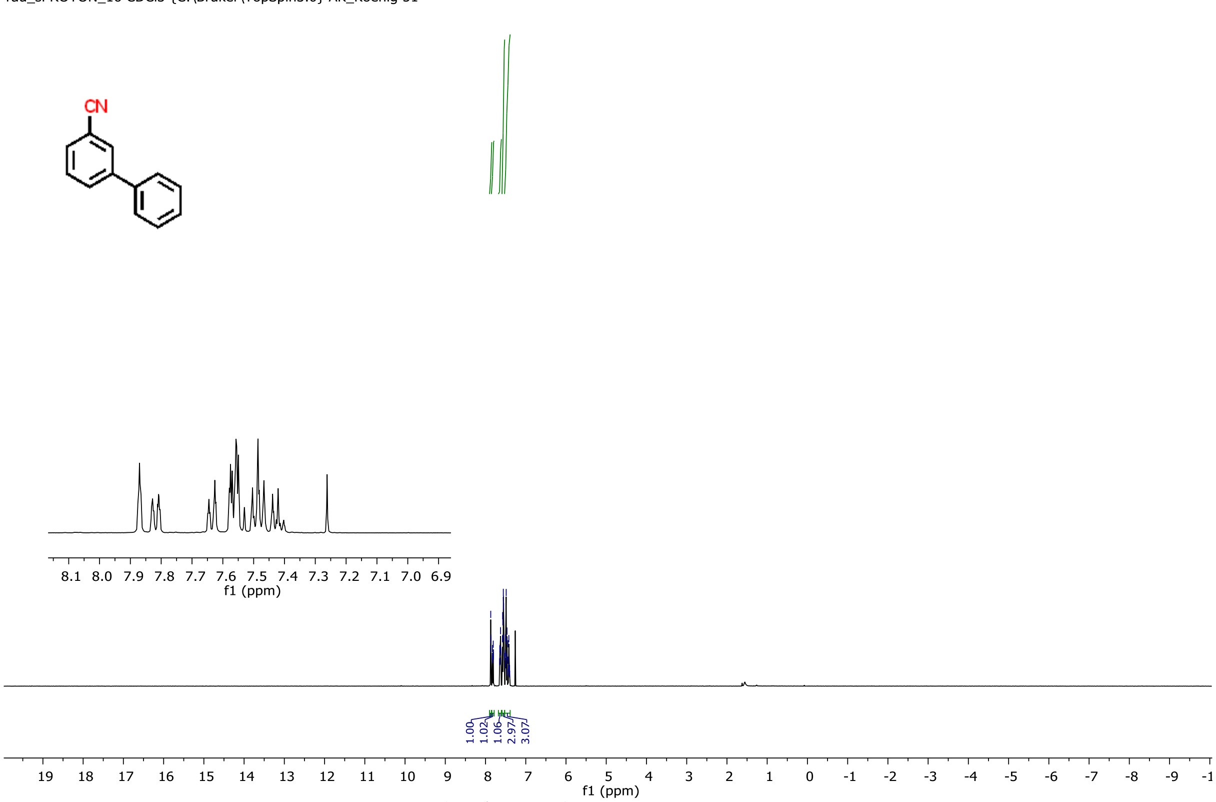

Jun26-2020.191.fid
KM05-523
rau_sC13CPD_256 CDCl3 {C:\Bruker\TopSpin3.0} AK_Koenig 51

142.48 138.91 131.51 130.74 130.71 129.62 129.15 128.41 127.11 118.87 112.99

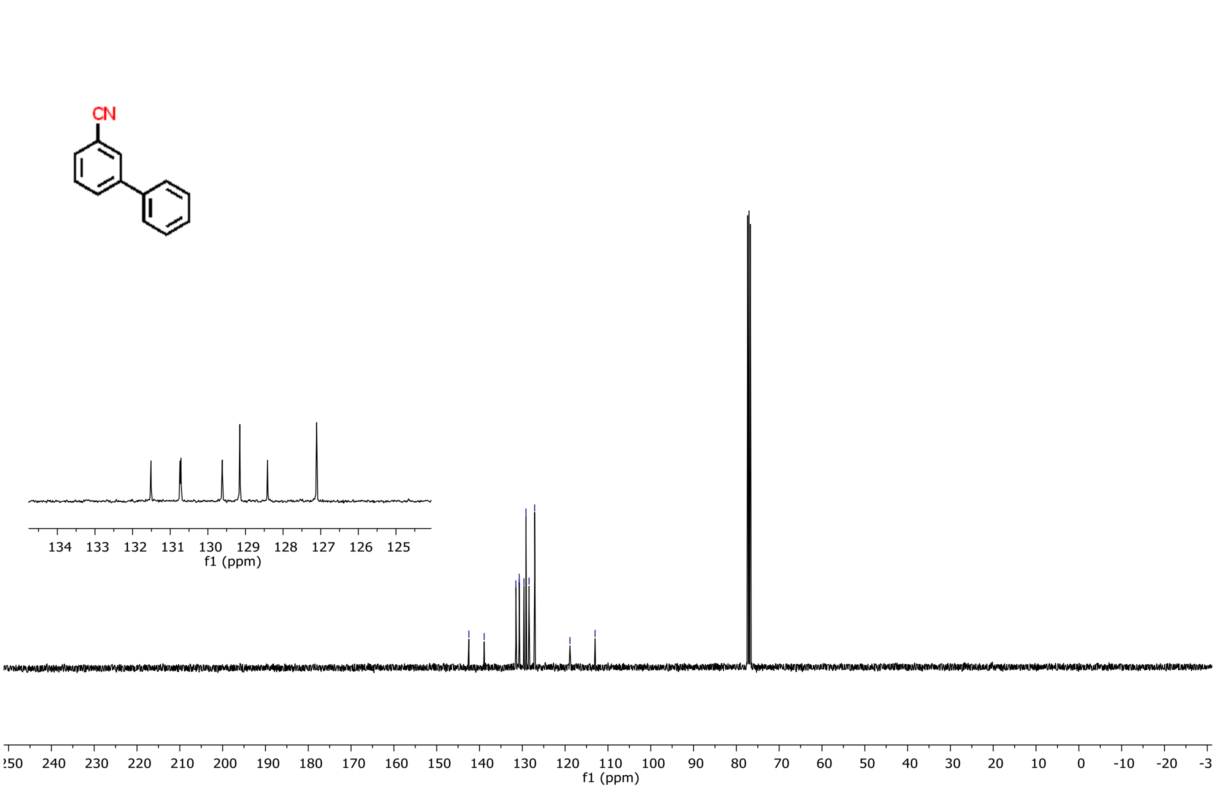

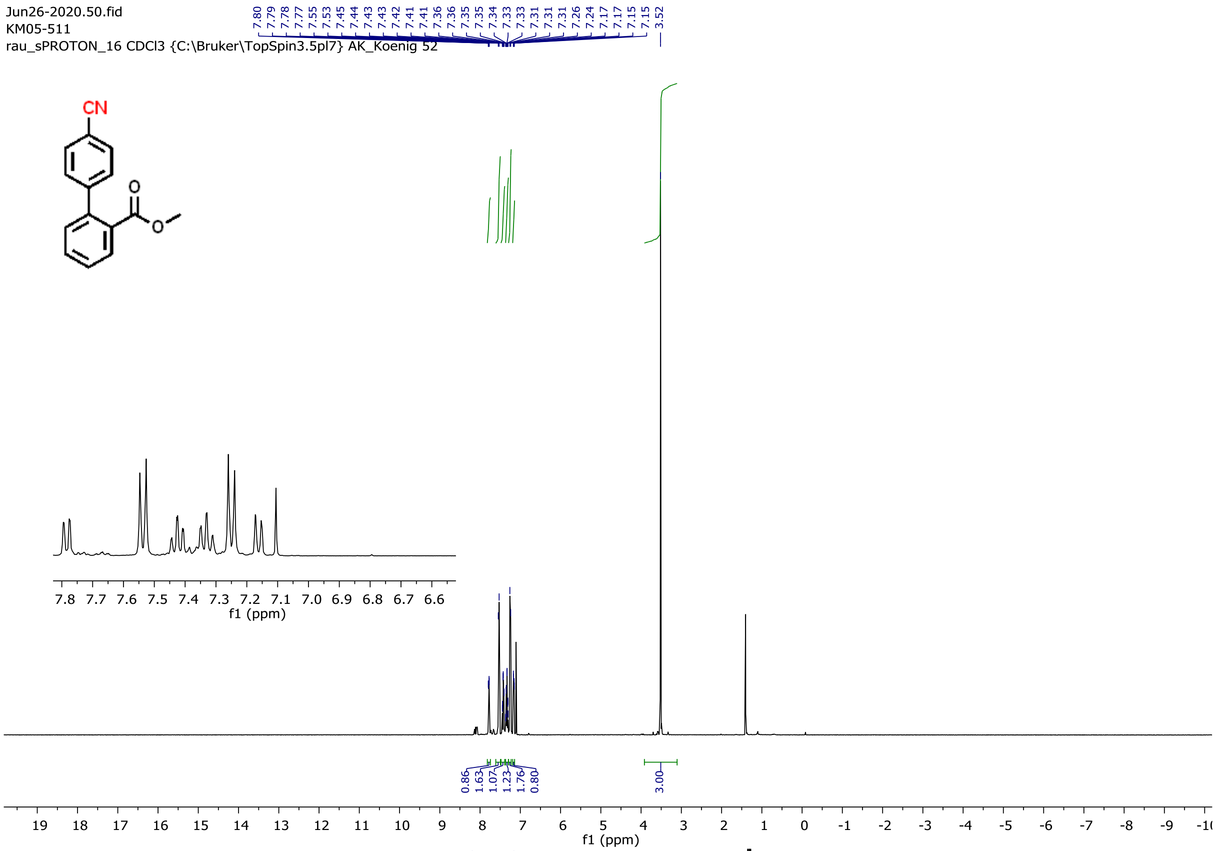
Jun26-2020.50.fid
KM05-511
rau_sPROTON_16 CDCl3 {C:\Bruker\TopSpin3.5pl7} AK_Koenig 52
7.80 7.79 7.78 7.77 7.55 7.53 7.45 7.44 7.43 7.43 7.42 7.41 7.41 7.36 7.36 7.35 7.35 7.34 7.33 7.33 7.31 7.31 7.26 7.24 7.17 7.17 7.15 3.52
CN
O
O
0.86 1.63 1.07 1.23 1.76 0.80
3.00
f1 (ppm)

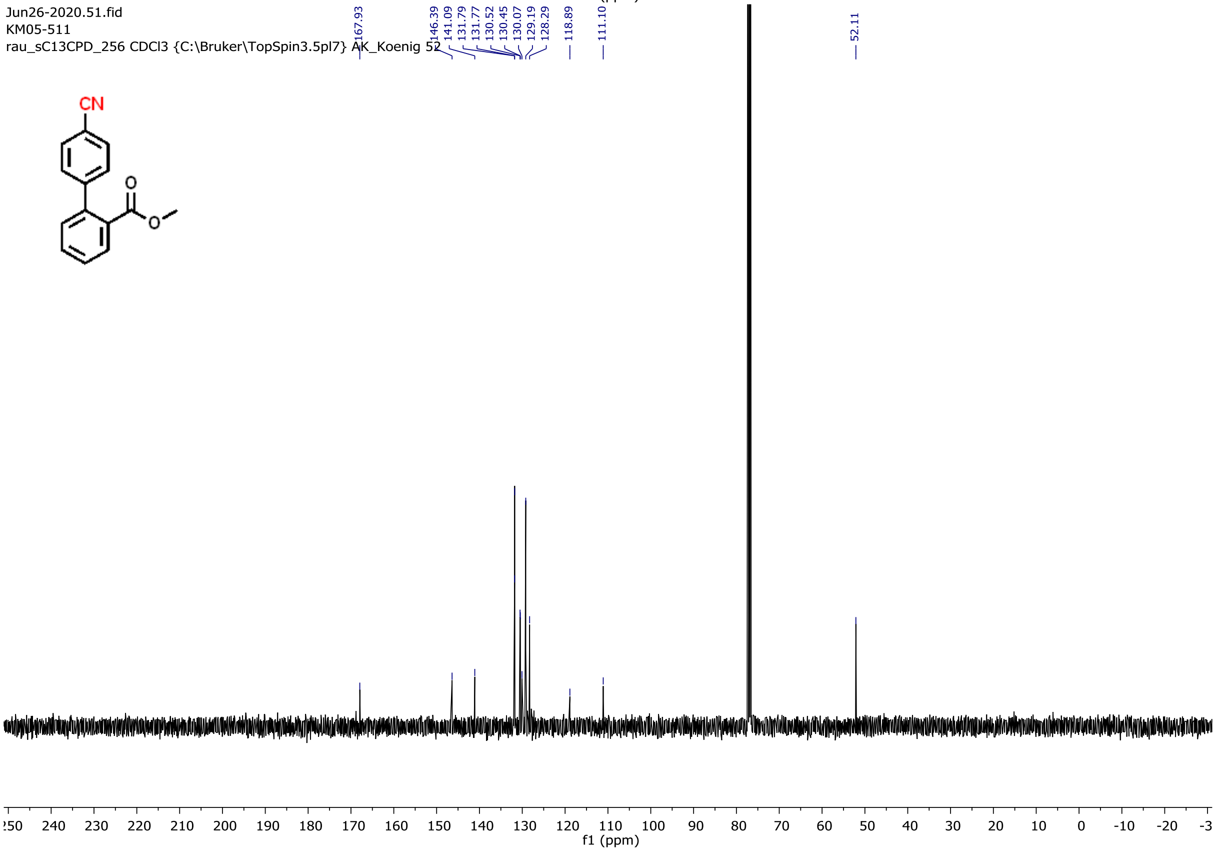
Jun26-2020.51.fid
KM05-511
rau_sC13CPD_256 CDCl3 {C:\Bruker\TopSpin3.5pl7} AK_Koenig 52
167.93
146.39 141.09 131.79 131.77 130.52 130.45 130.07 129.19 128.29
118.89
111.10
52.11
CN
O
O
f1 (ppm)

Jun12-2020.12.fid
KM05-499
rau_sPROTON_64 CDCl3 {C:\Bruker\TopSpin3.5pl7} AK_Koenig 18

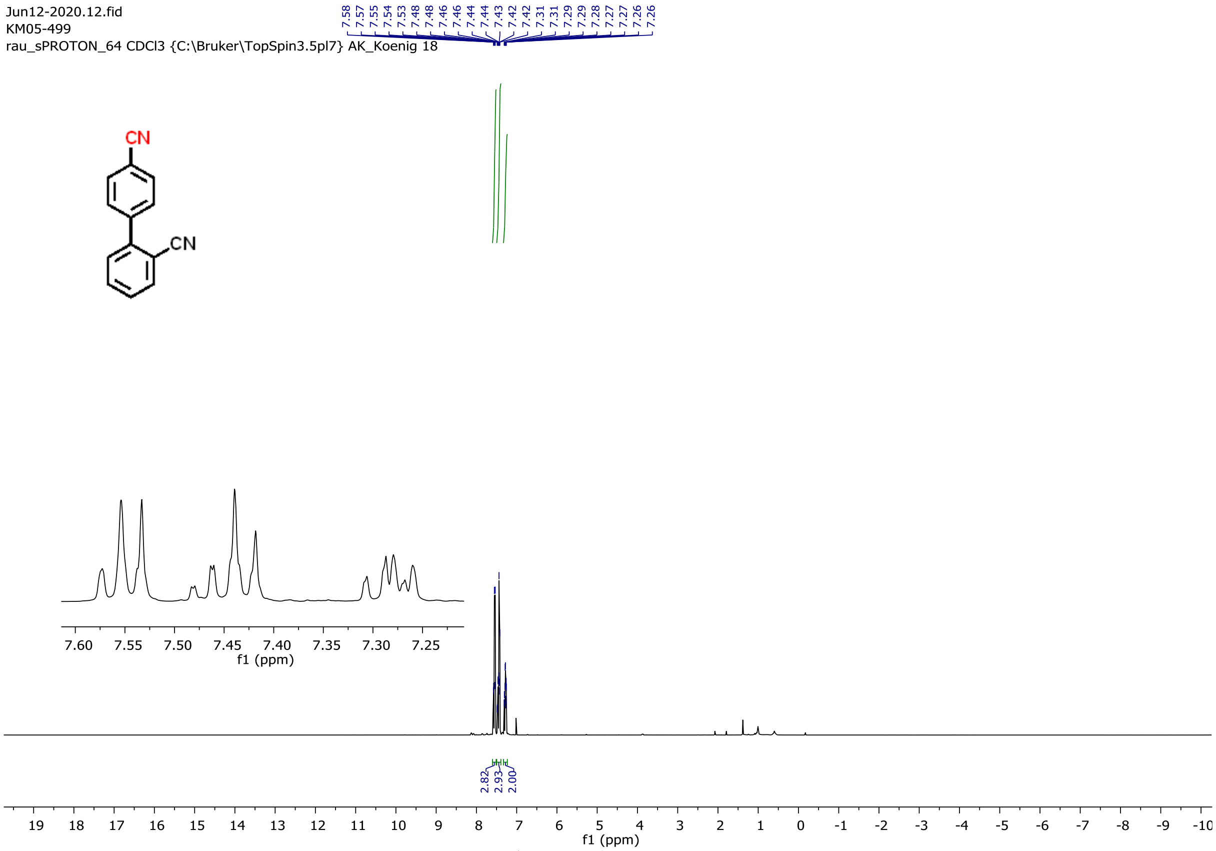

Jun12-2020.13.fid
KM05-499
rau_sC13CPD_256 CDCl3 {C:\Bruker\TopSpin3.5pl7} AK_Koenig 18

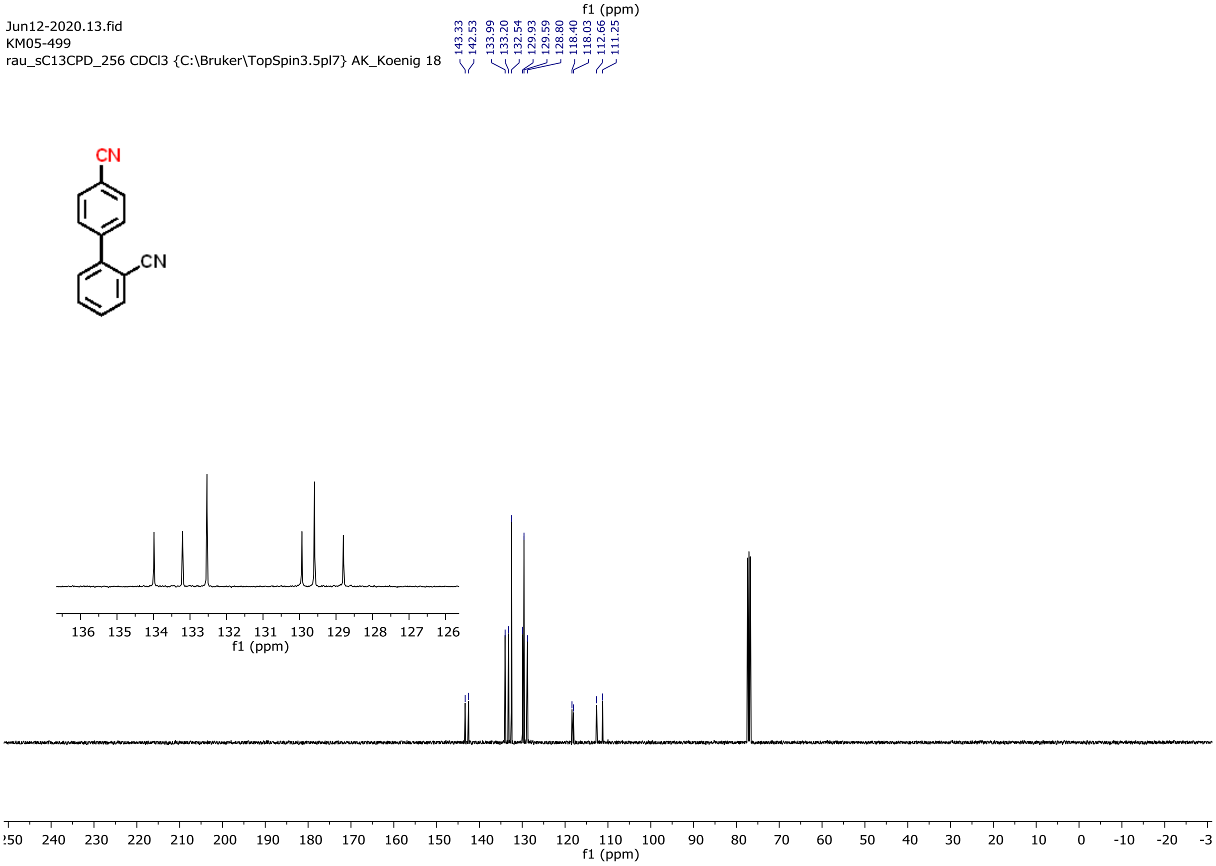

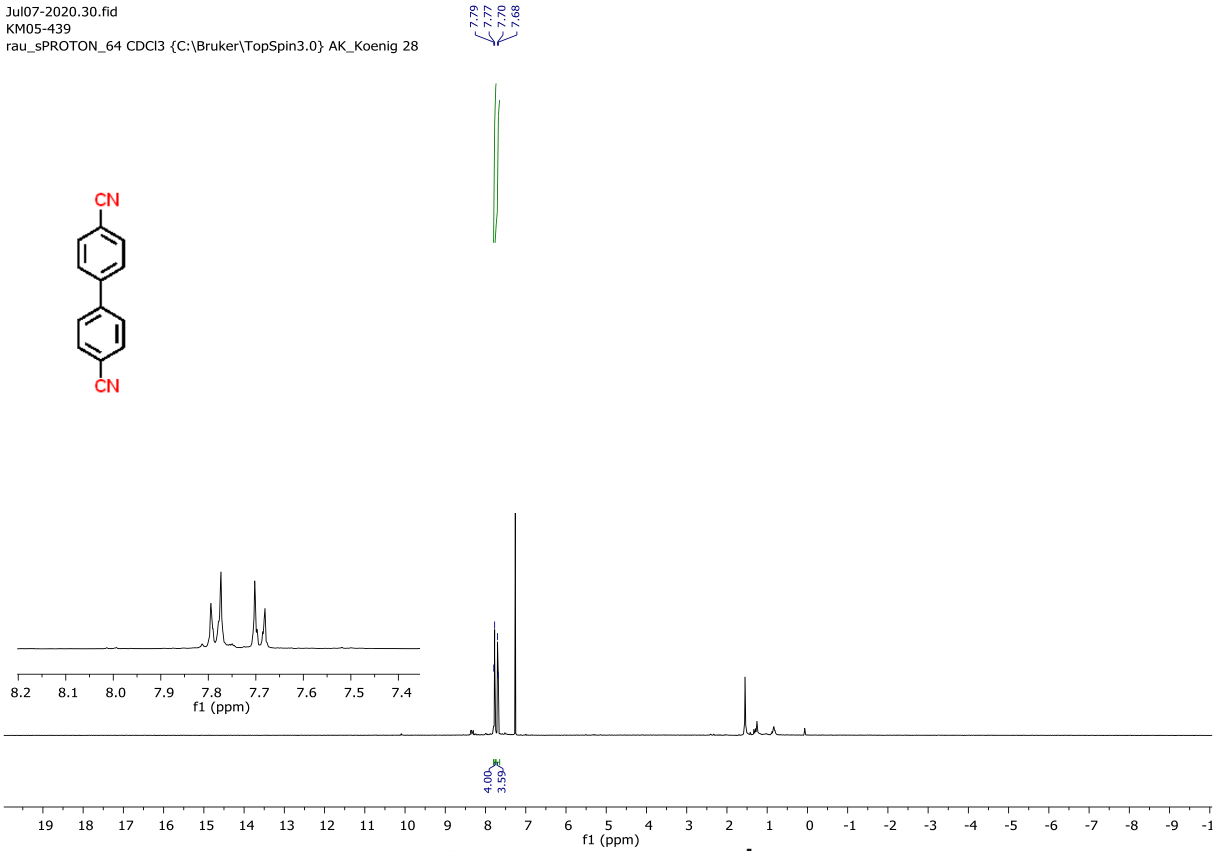
Jul07-2020.30.fid
KM05-439
rau_sPROTON_64 CDCl3 {C:\Bruker\TopSpin3.0} AK_Koenig 28
7.79
7.77
7.70
7.68
CN
CN
4.00
3.59
f1 (ppm)
f1 (ppm)

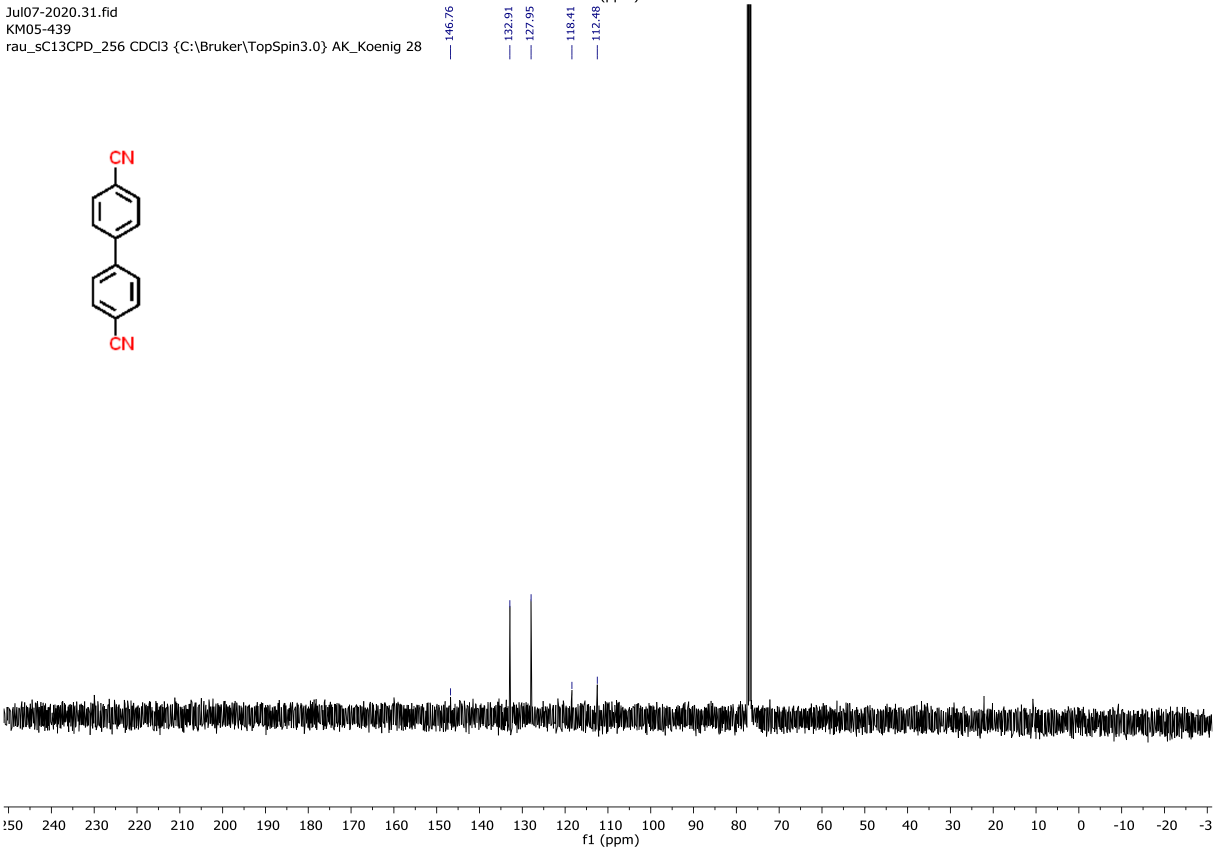
Jul07-2020.31.fid
KM05-439
rau_sC13CPD_256 CDCl3 {C:\Bruker\TopSpin3.0} AK_Koenig 28
146.76
132.91
127.95
118.41
112.48
CN
CN
f1 (ppm)

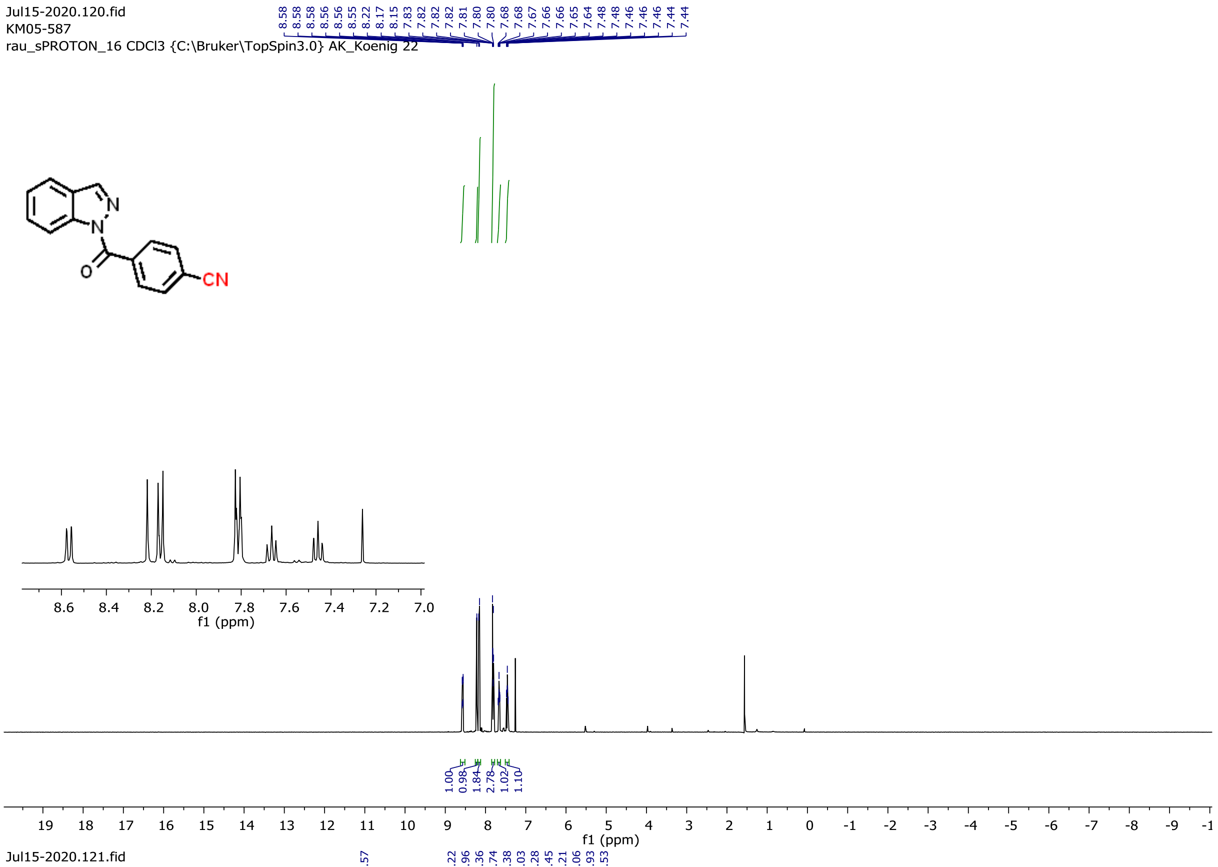
Jul15-2020.120.fid
KM05-587
rau_sPROTON_16 CDCl3 {C:\Bruker\TopSpin3.0} AK_Koenig 22
8.58 8.58 8.58 8.56 8.56 8.55 8.22 8.17 8.15 7.83 7.82 7.82 7.82 7.81 7.80 7.80 7.68 7.68 7.67 7.66 7.66 7.65 7.64 7.48 7.46 7.46 7.44 7.44
N
N
O
CN
1.00 0.98 1.84 2.78 1.02 1.10
f1 (ppm)

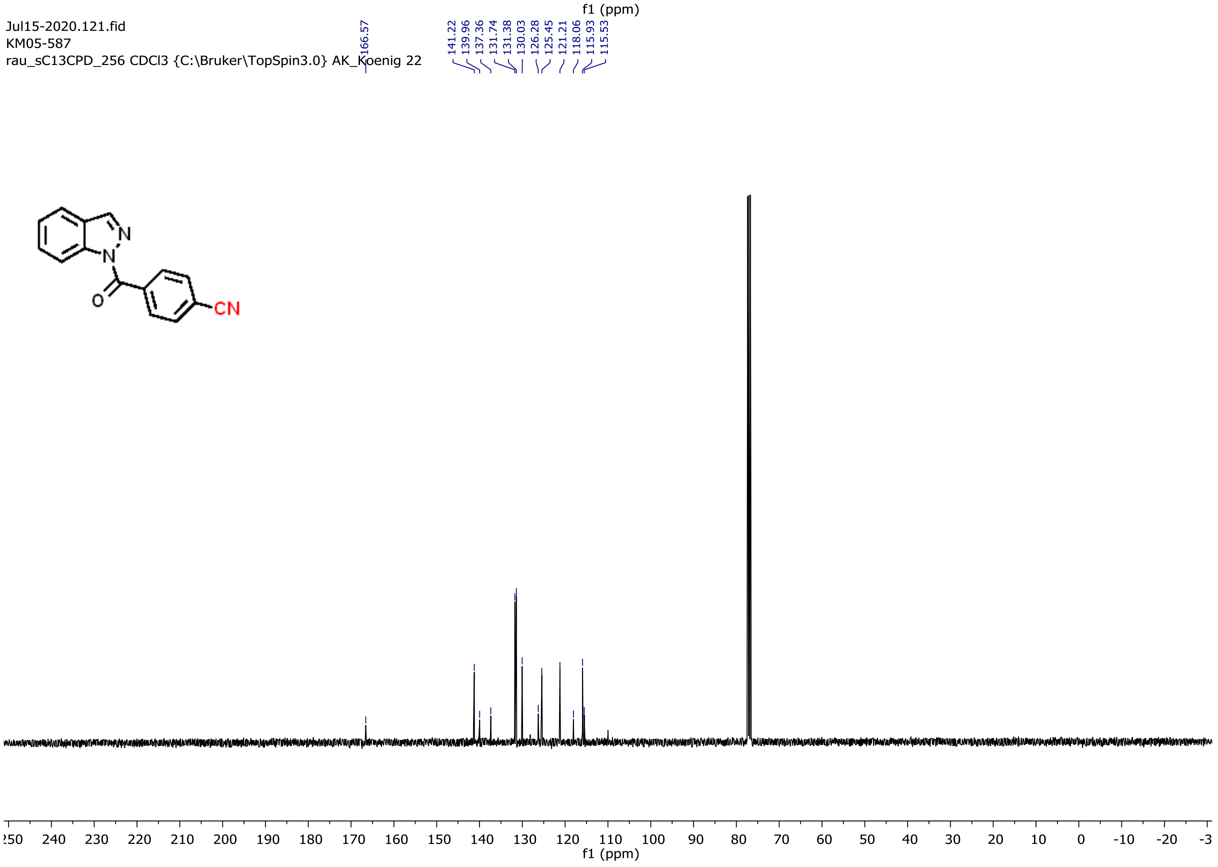
Jul15-2020.121.fid
KM05-587
rau_sC13CPD_256 CDCl3 {C:\Bruker\TopSpin3.0} AK_Koenig 22
166.57
141.22 139.96 137.36 131.74 131.38 130.03 126.28 125.45 121.21 118.06 115.93 115.53
N
N
O
CN
f1 (ppm)

Jun30-2020.80.fid
KM05-529
rau_sPROTON_64 CDCl3 {C:\Bruker\TopSpin3.5pl7} AK_Koenig 40

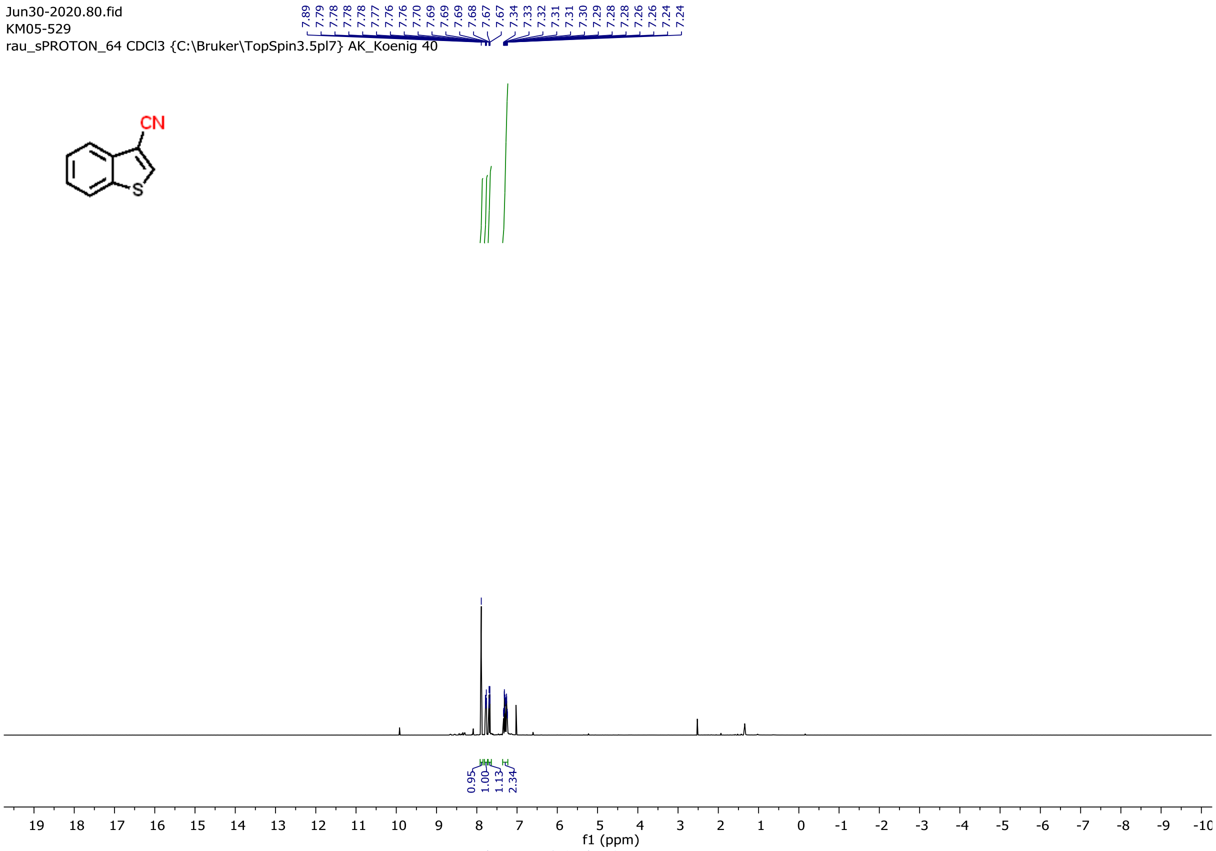

Jun30-2020.82.fid
KM05-529
rau_sC13CPD_256 CDCl3 {C:\Bruker\TopSpin3.5pl7} AK_Koenig 40

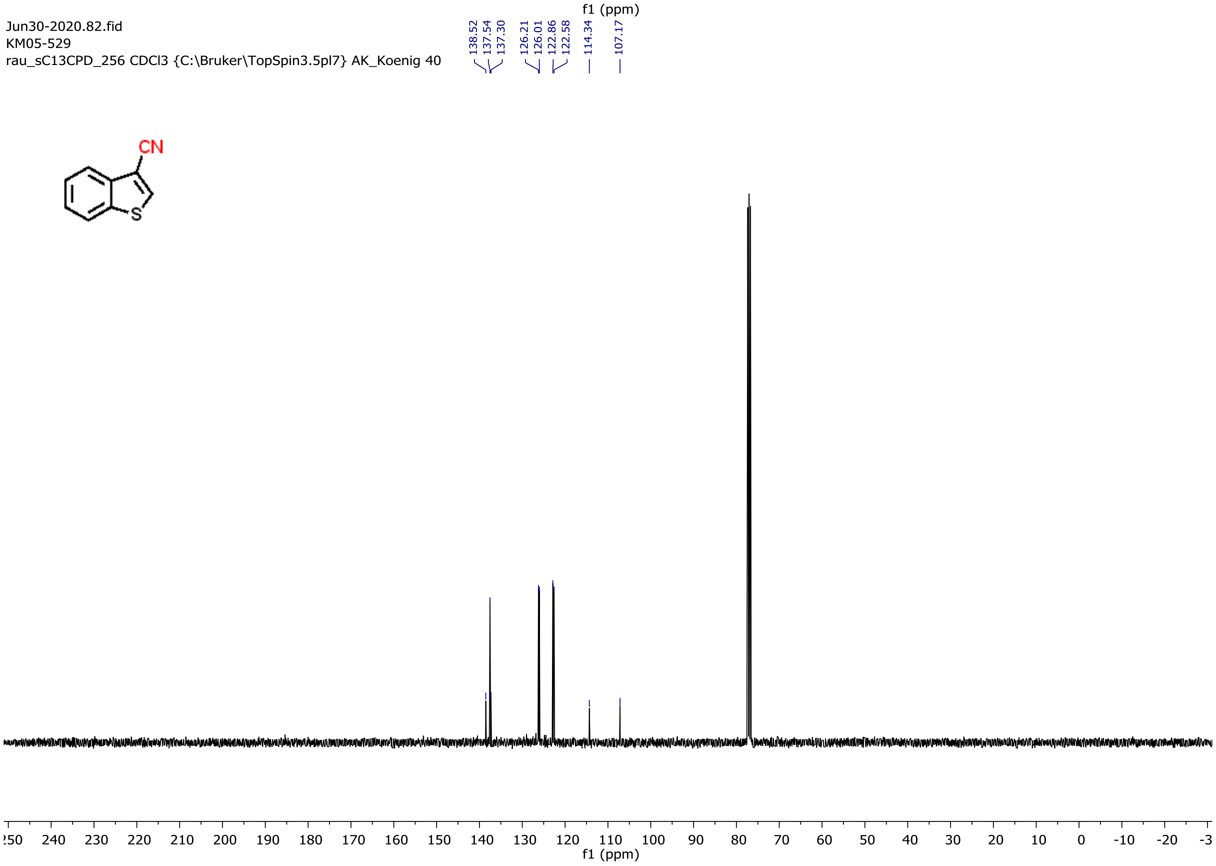

Jun26-2020.10.fid
KM05-505
rau_sPROTON_16 CDCl3 {C:\Bruker\TopSpin3.5pl7} AK_Koenig 48

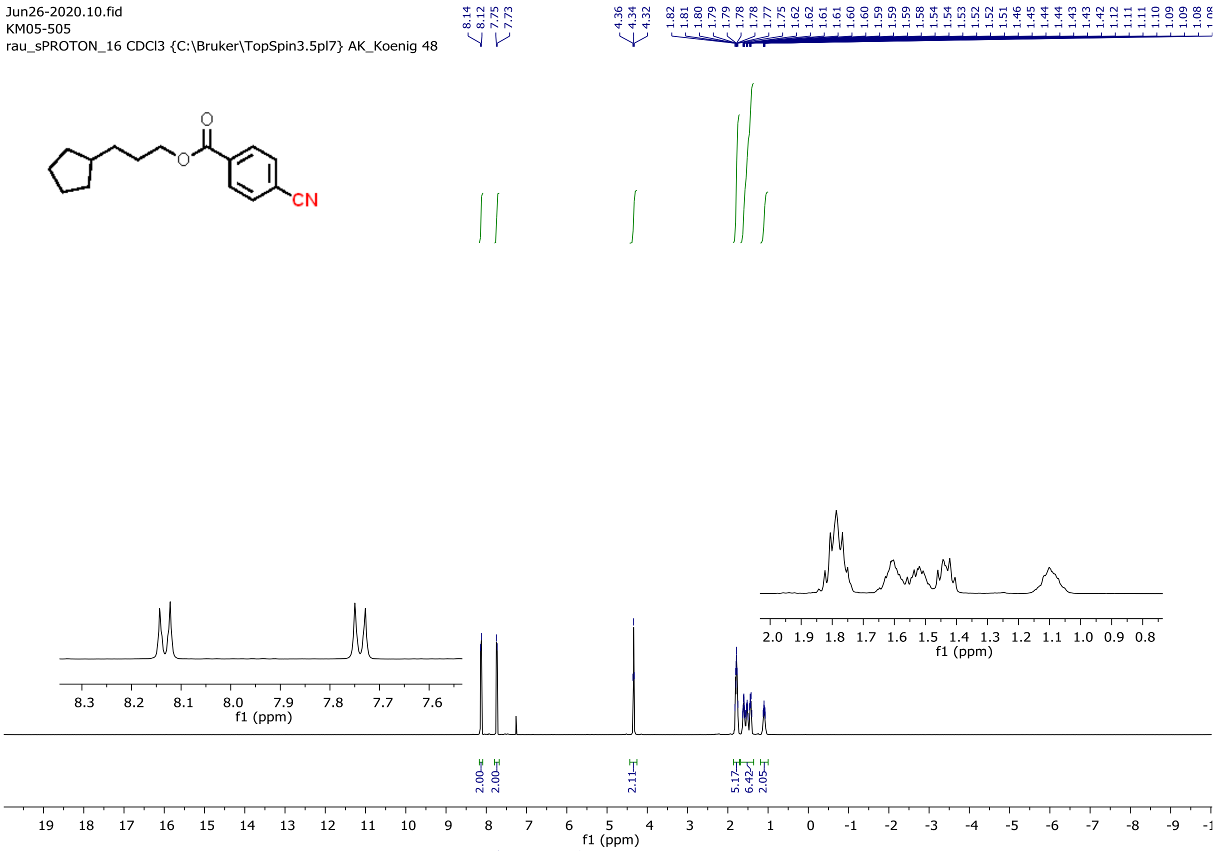

Jun26-2020.11.fid
KM05-505
rau_sC13CPD_256 CDCl3 {C:\Bruker\TopSpin3.5pl7} AK_Koenig 48

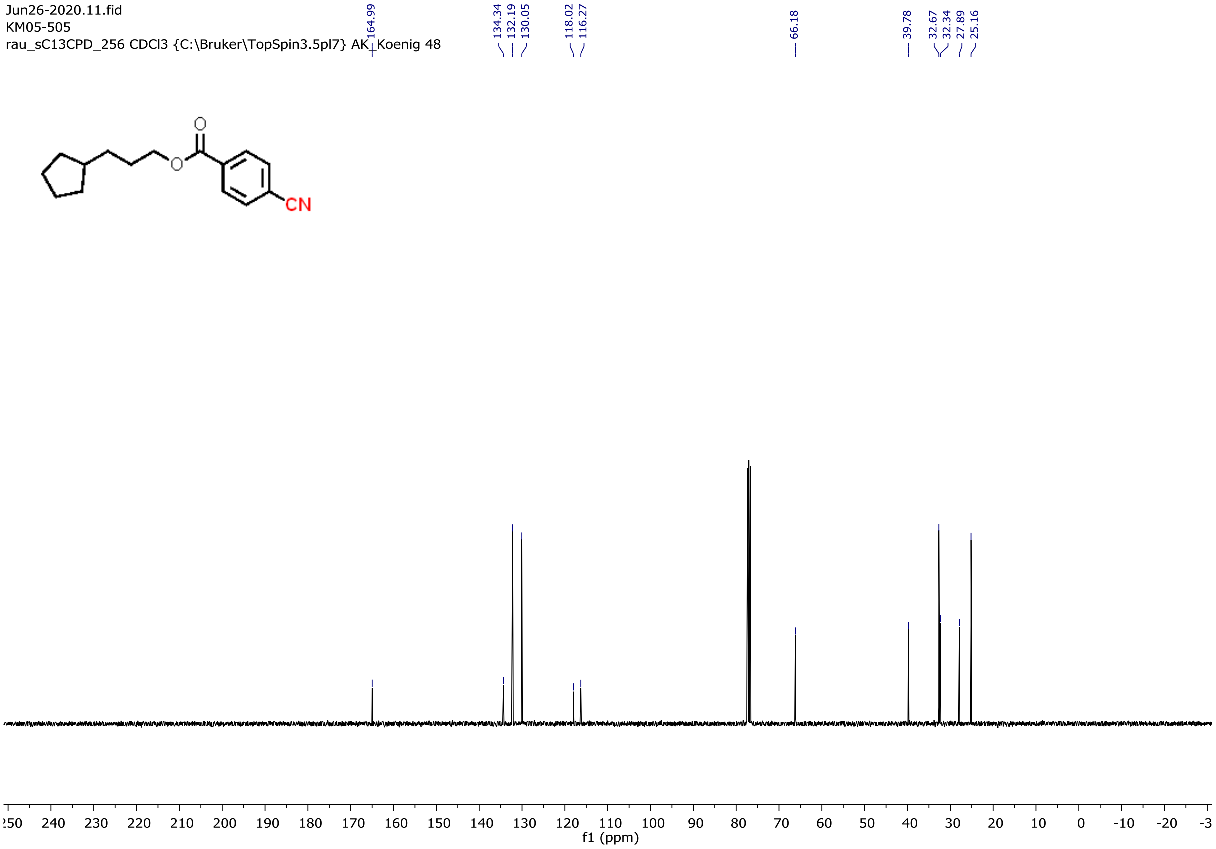

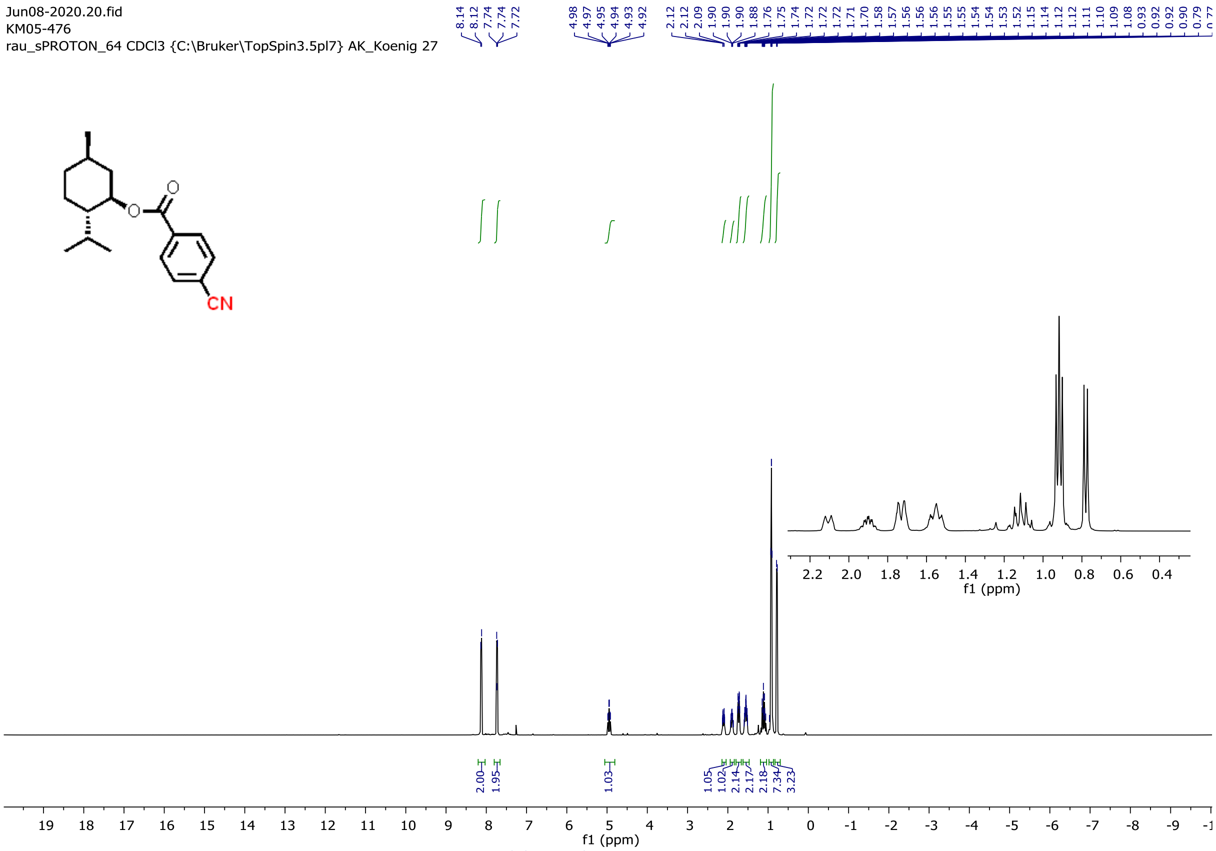
Jun08-2020.20.fid
KM05-476
rau_sPROTON_64 CDCl3 {C:\Bruker\TopSpin3.5pl7} AK_Koenig 27
8.14
8.12
7.74
7.74
7.72
4.98
4.97
4.95
4.94
4.93
4.92
2.12
2.12
2.09
1.90
1.90
1.90
1.88
1.76
1.75
1.74
1.72
1.72
1.71
1.70
1.58
1.57
1.56
1.56
1.55
1.55
1.54
1.54
1.53
1.52
1.15
1.14
1.12
1.12
1.11
1.10
1.09
1.08
0.93
0.92
0.92
0.90
0.79
CN
2.00
1.95
1.03
1.05
1.02
2.14
2.17
2.18
7.34
3.23
f1 (ppm)
f1 (ppm)

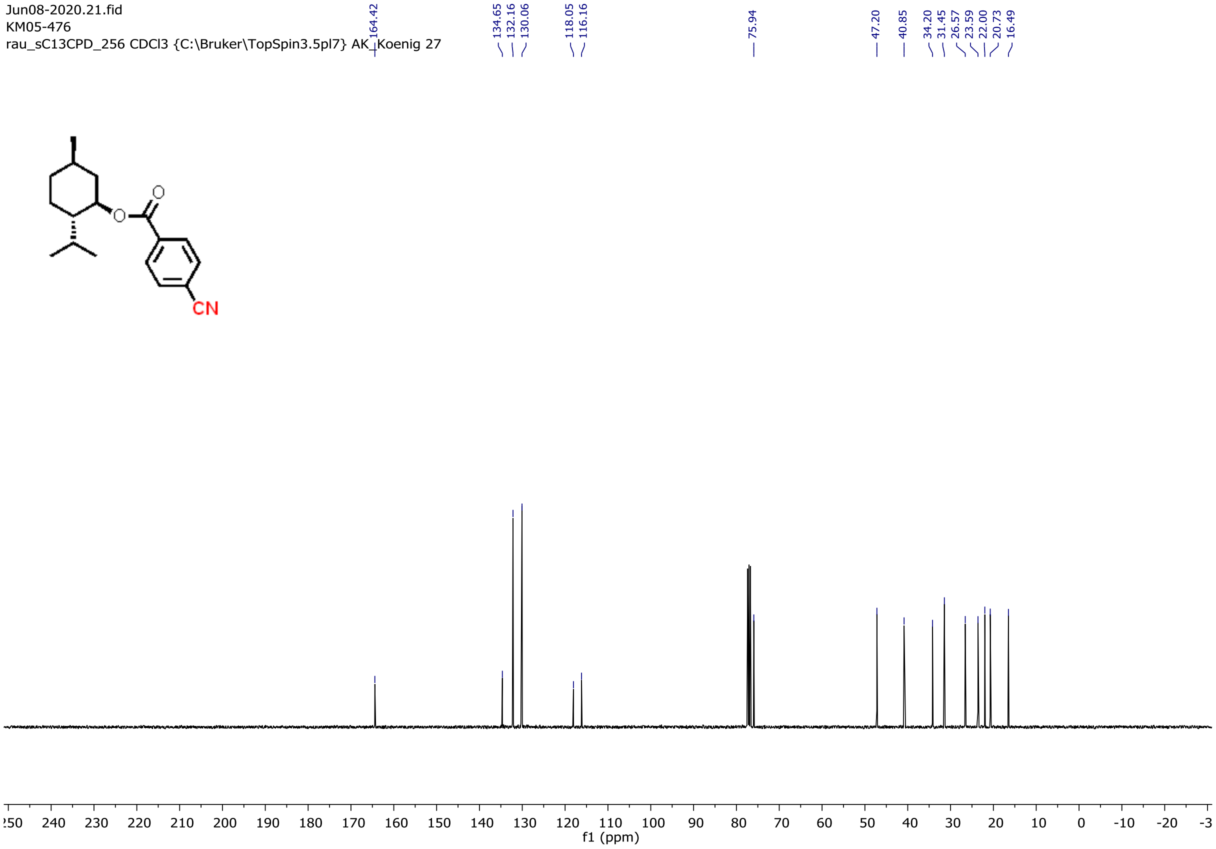
Jun08-2020.21.fid
KM05-476
rau_sC13CPD_256 CDCl3 {C:\Bruker\TopSpin3.5pl7} AK_Koenig 27
164.42
134.65
132.16
130.06
118.05
116.16
75.94
47.20
40.85
34.20
31.45
26.57
23.59
22.00
20.73
16.49
CN
f1 (ppm)

Jun23-2020.40.fid
KM05-502
rau_sPROTON_64 CDCl3 {C:\Bruker\TopSpin3.0} AK_Koenig 22

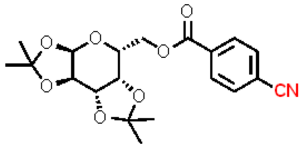

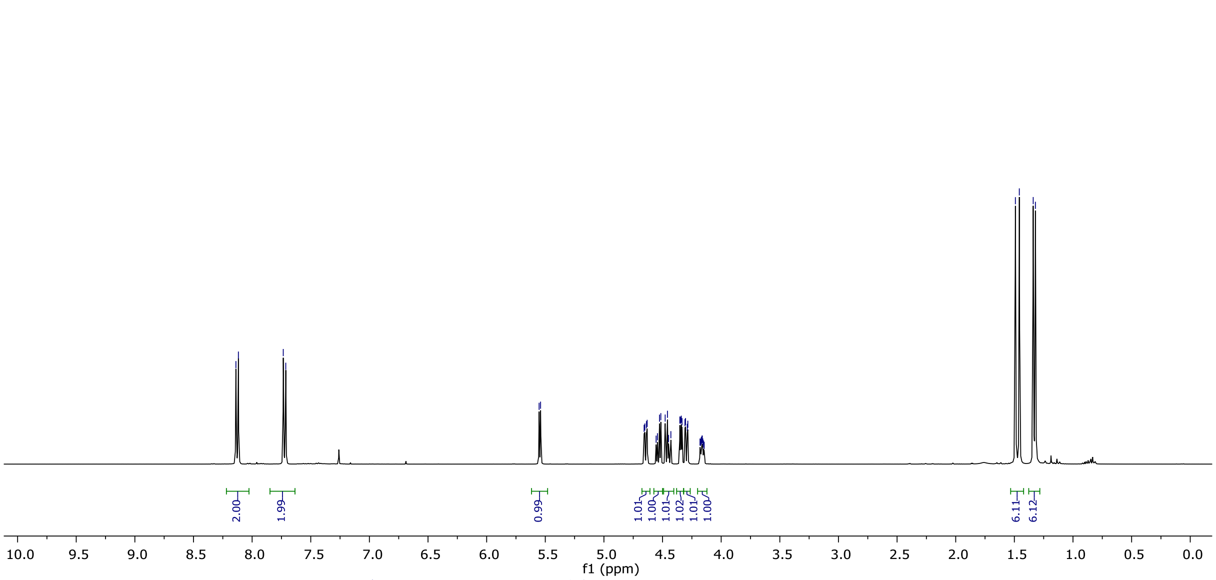

Jun23-2020.41.fid
KM05-502
rau_sC13CPD_256 CDCl3 {C:\Bruker\TopSpin3.0} AK_Koenig 22

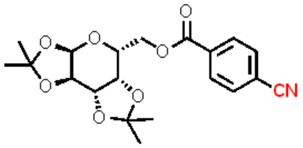

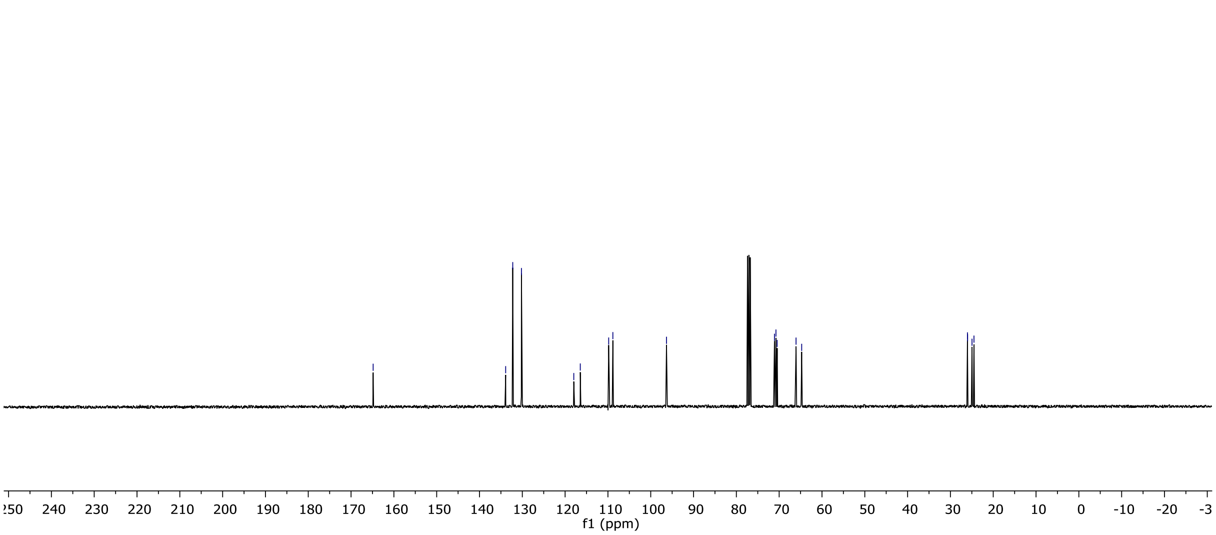

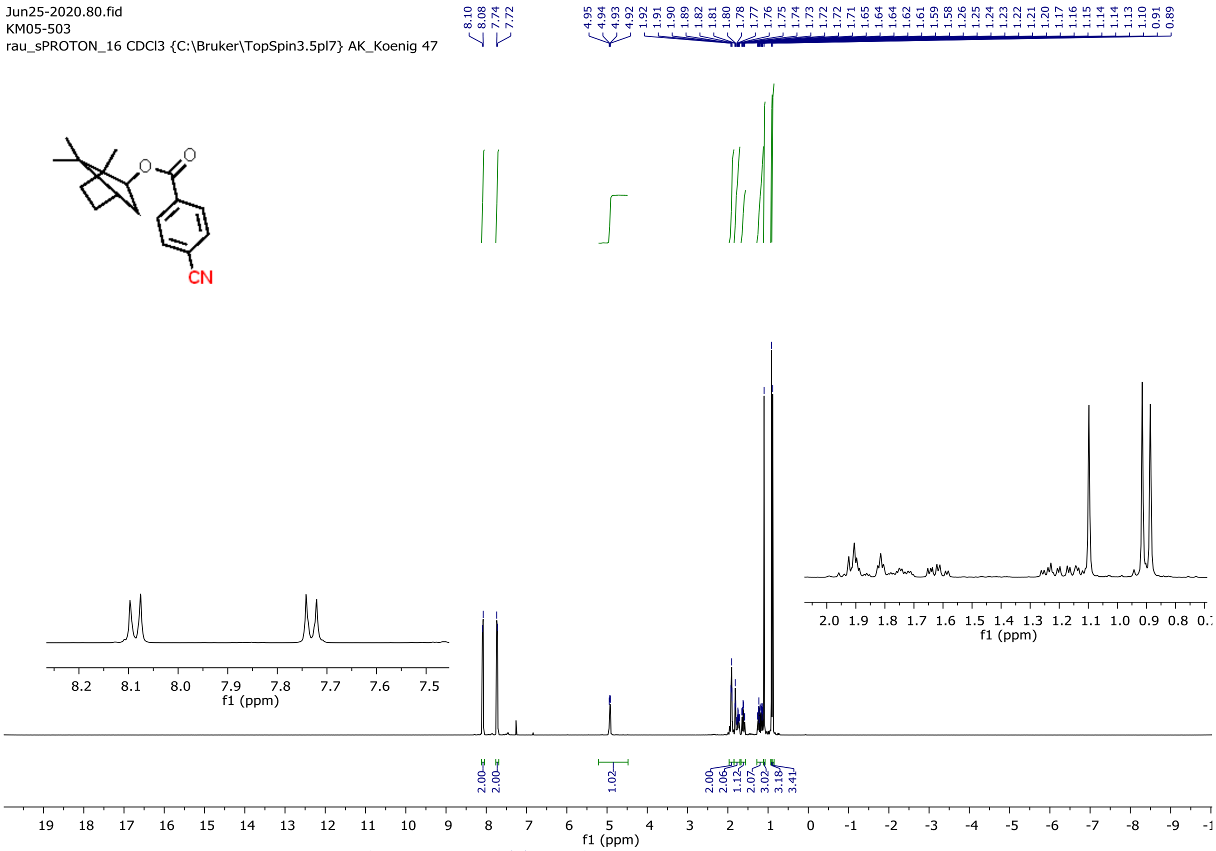
Jun25-2020.80.fid
KM05-503
rau_sPROTON_16 CDCl3 {C:\Bruker\TopSpin3.5pl7} AK_Koenig 47
8.10 8.08 7.74 7.72
4.95 4.94 4.93 4.92 1.92 1.91 1.90 1.89 1.82 1.81 1.80 1.78 1.77 1.76 1.75 1.74 1.73 1.72 1.72 1.71 1.65 1.64 1.64 1.62 1.61 1.59 1.58 1.26 1.25 1.24 1.23 1.22 1.21 1.20 1.17 1.16 1.15 1.14 1.14 1.13 1.10 0.91 0.89
CN
2.00 2.00 1.02 2.00 2.06 1.12 2.07 3.02 3.18 3.41
f1 (ppm)

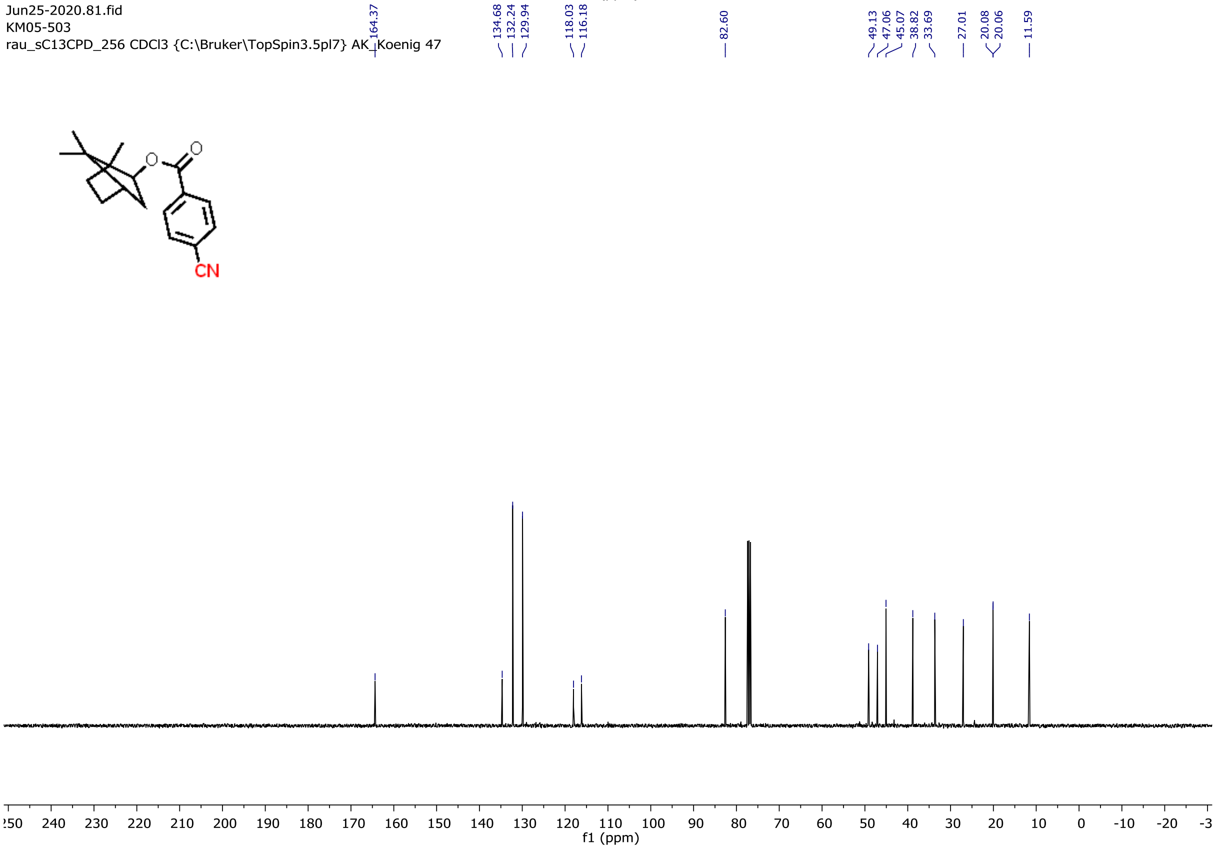
Jun25-2020.81.fid
KM05-503
rau_sC13CPD_256 CDCl3 {C:\Bruker\TopSpin3.5pl7} AK_Koenig 47
164.37
134.68 132.24 129.94
118.03 116.18
82.60
49.13 47.06 45.07 38.82 33.69 27.01 20.08 20.06 11.59
CN
f1 (ppm)

Jun26-2020.20.fid
KM05-506
rau_sPROTON_16 CDCl3 {C:\Bruker\TopSpin3.5pl7} AK_Koenig 49

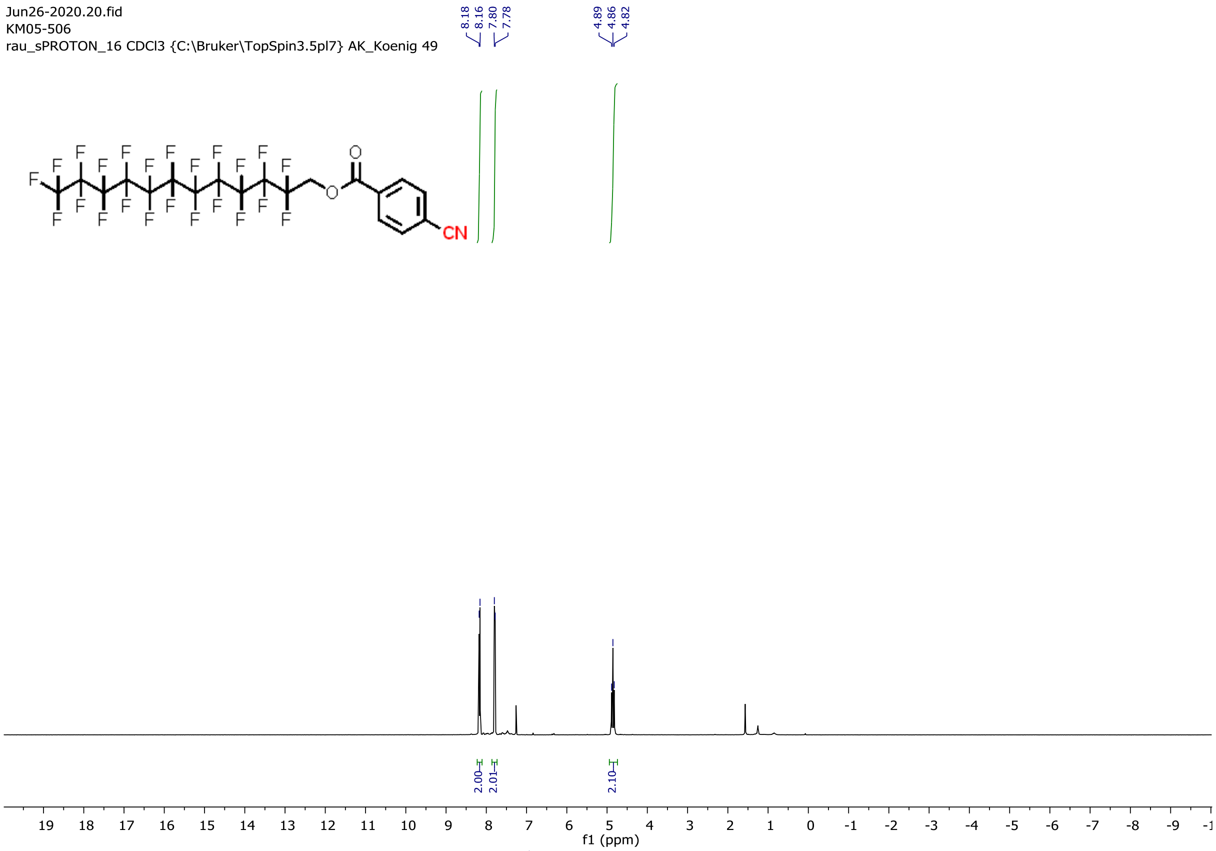

Jun26-2020.21.fid
KM05-506
rau_sC13CPD_256 CDCl3 {C:\Bruker\TopSpin3.5pl7} AK_Koenig 49

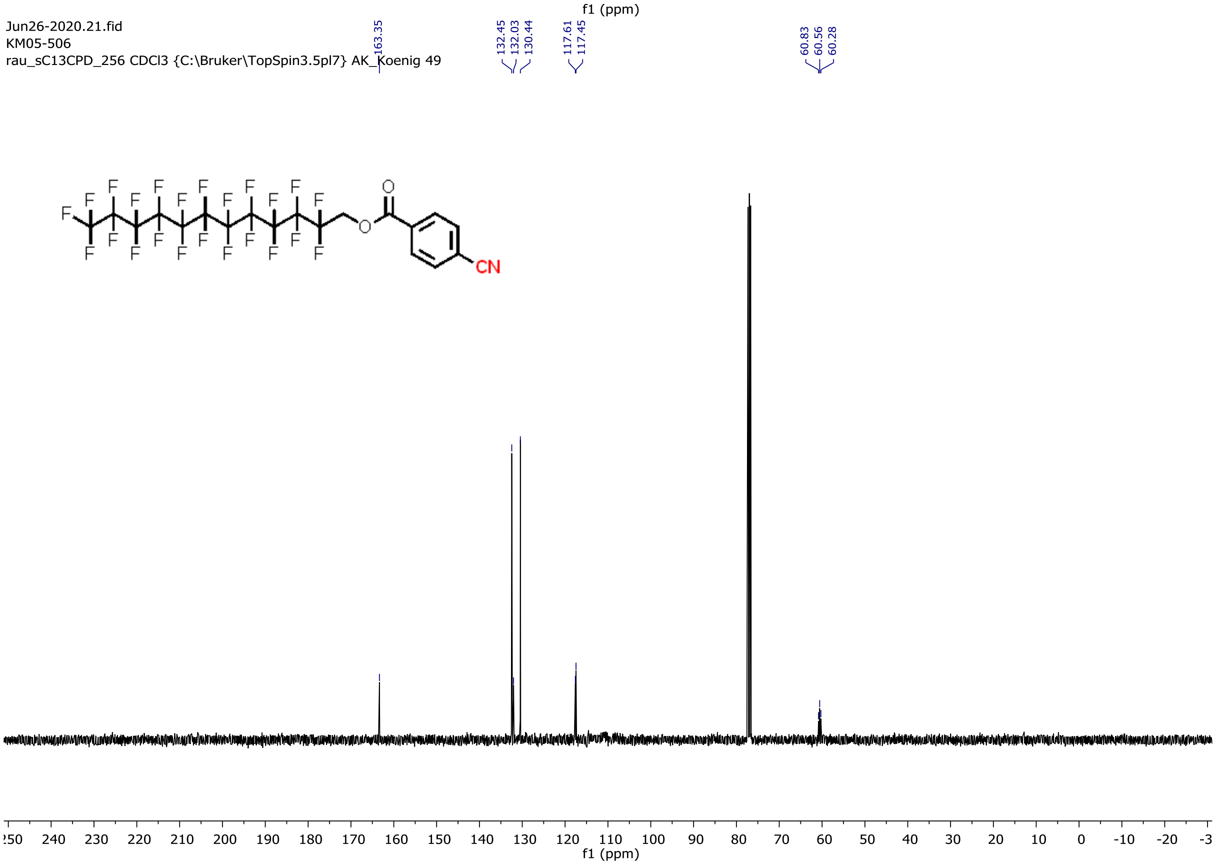

Jun30-2020.92.fid
KM05-506
rau_F19CPD CDCl3 {C:\Bruker\TOPSPIN2.1PL3} AK_Koenig 55

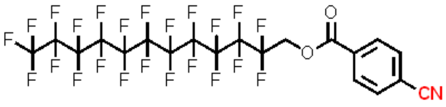

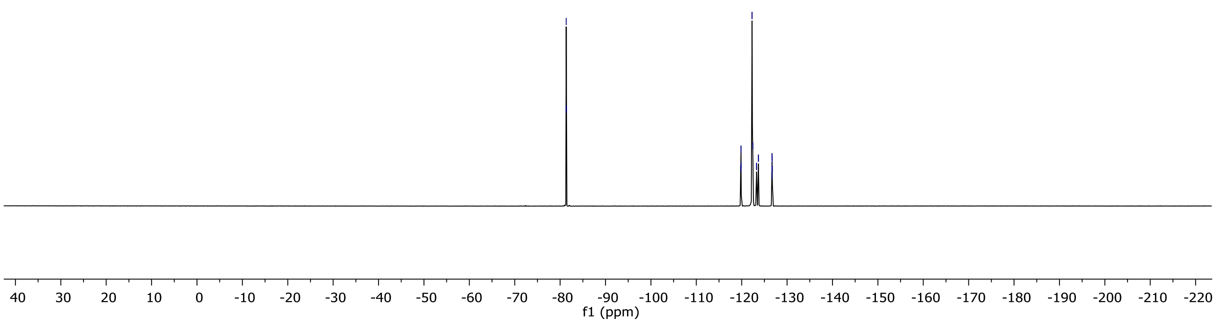

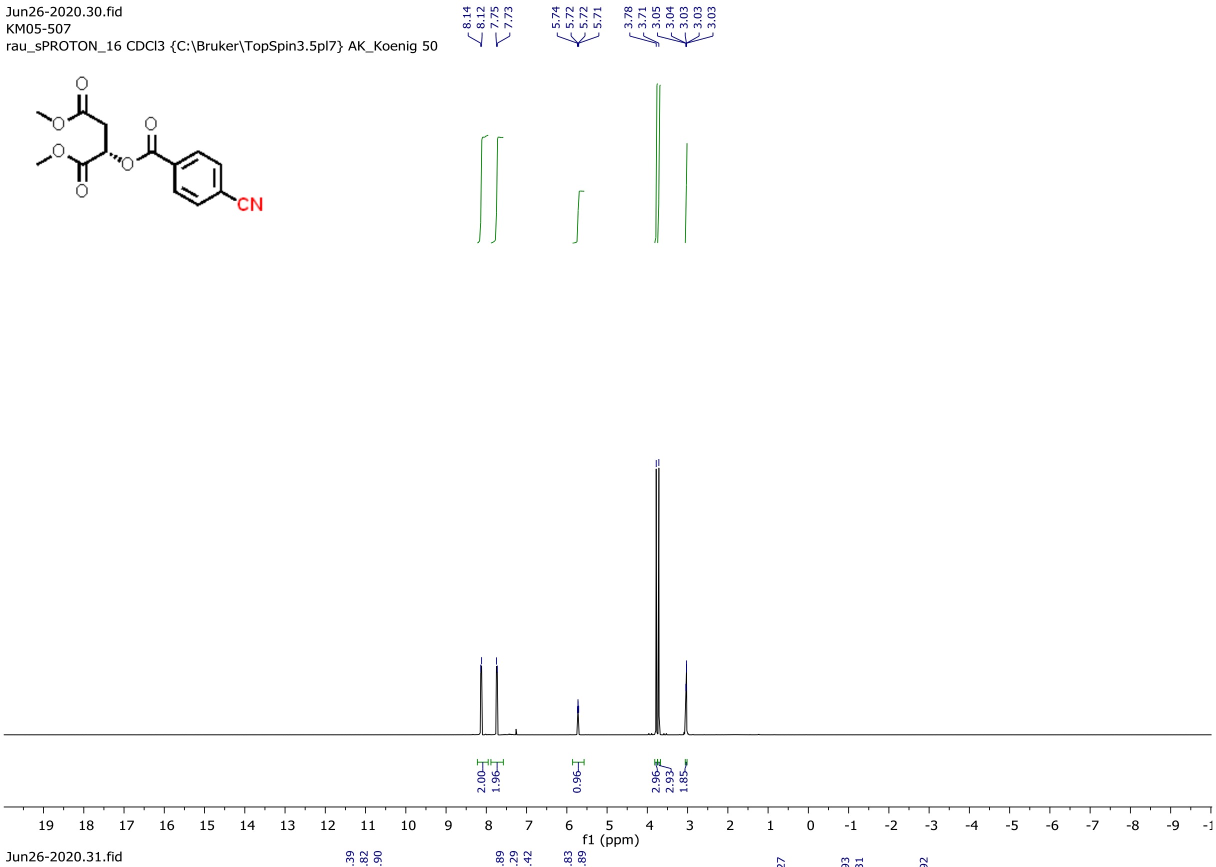
Jun26-2020.30.fid
KM05-507
rau_sPROTON_16 CDCl3 {C:\Bruker\TopSpin3.5pl7} AK_Koenig 50
CN
8.14
8.12
7.75
7.73
5.74
5.72
5.72
5.71
3.78
3.71
3.05
3.04
3.03
3.03
3.03
2.00
1.96
0.96
2.96
2.93
1.85
f1 (ppm)

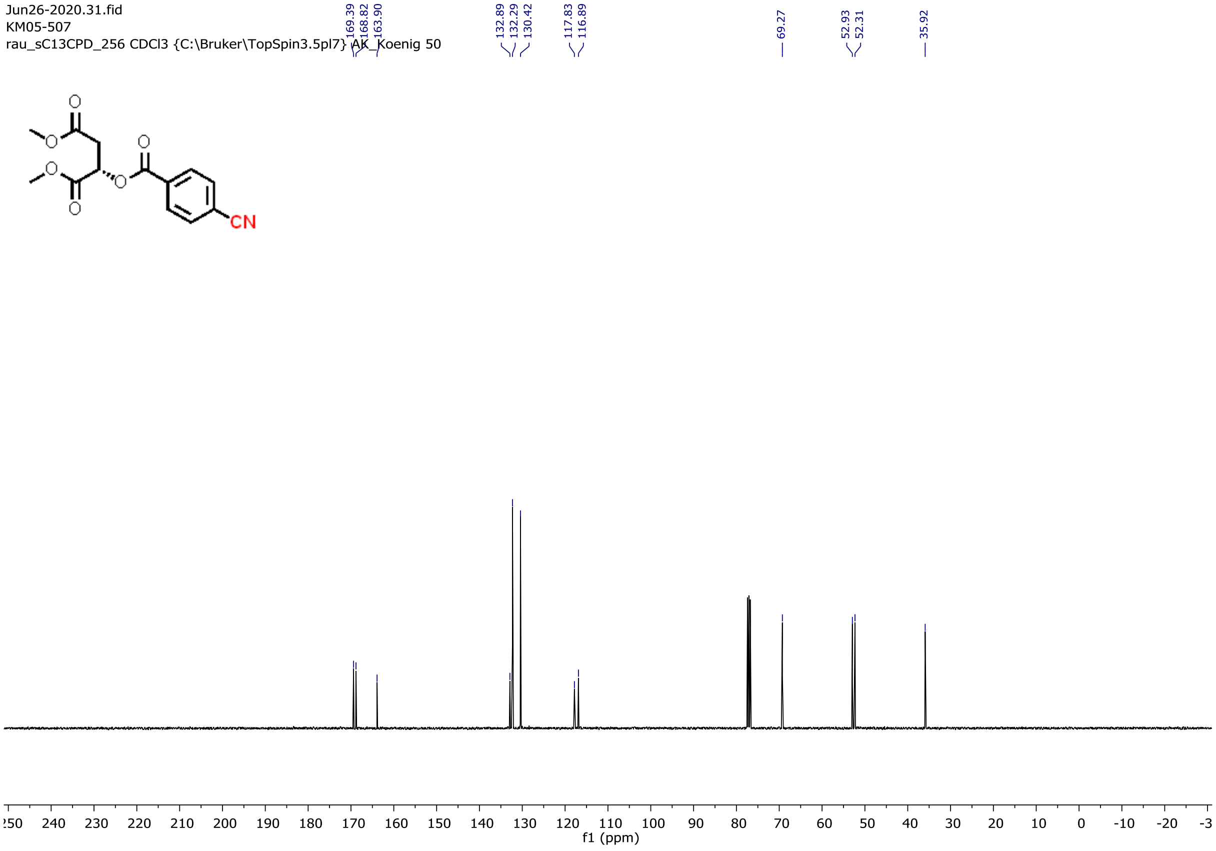
Jun26-2020.31.fid
KM05-507
rau_sC13CPD_256 CDCl3 {C:\Bruker\TopSpin3.5pl7} AK_Koenig 50
CN
169.39
168.82
163.90
132.89
132.29
130.42
117.83
116.89
69.27
52.93
52.31
35.92
f1 (ppm)

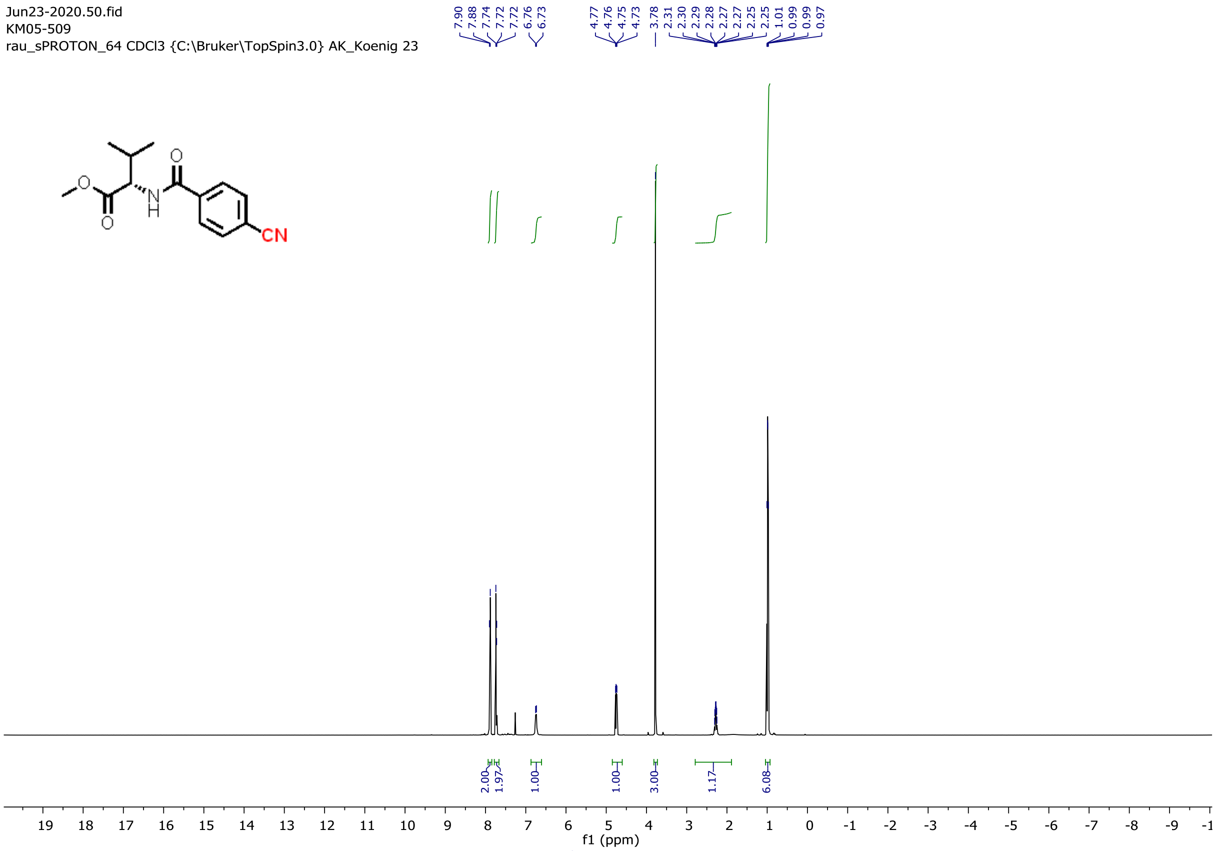

Jun23-2020.50.fid
KM05-509
rau_sPROTON_64 CDCl3 {C:\Bruker\TopSpin3.0} AK_Koenig 23
7.90 7.88 7.74 7.72 7.72 6.76 6.73 4.77 4.76 4.75 4.73 3.78 2.31 2.30 2.29 2.28 2.27 2.27 2.25 1.01 0.99 0.99 0.97
O
N
H
O
O
CN
2.00 1.97 1.00 1.00 3.00 1.17 6.08
f1 (ppm)

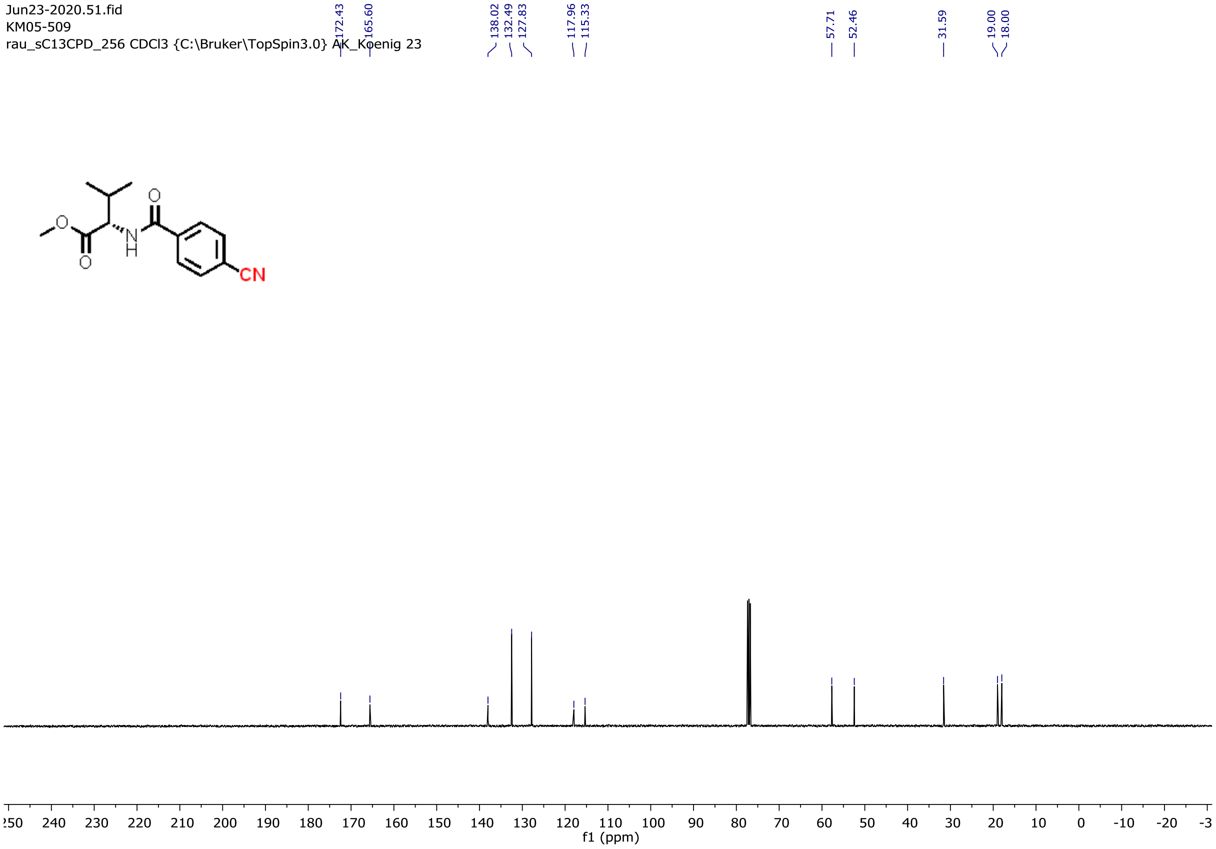

Jun23-2020.51.fid
KM05-509
rau_sC13CPD_256 CDCl3 {C:\Bruker\TopSpin3.0} AK_Koenig 23
172.43 165.60 138.02 132.49 127.83 117.96 115.33 57.71 52.46 31.59 19.00 18.00
O
N
H
O
O
CN
f1 (ppm)

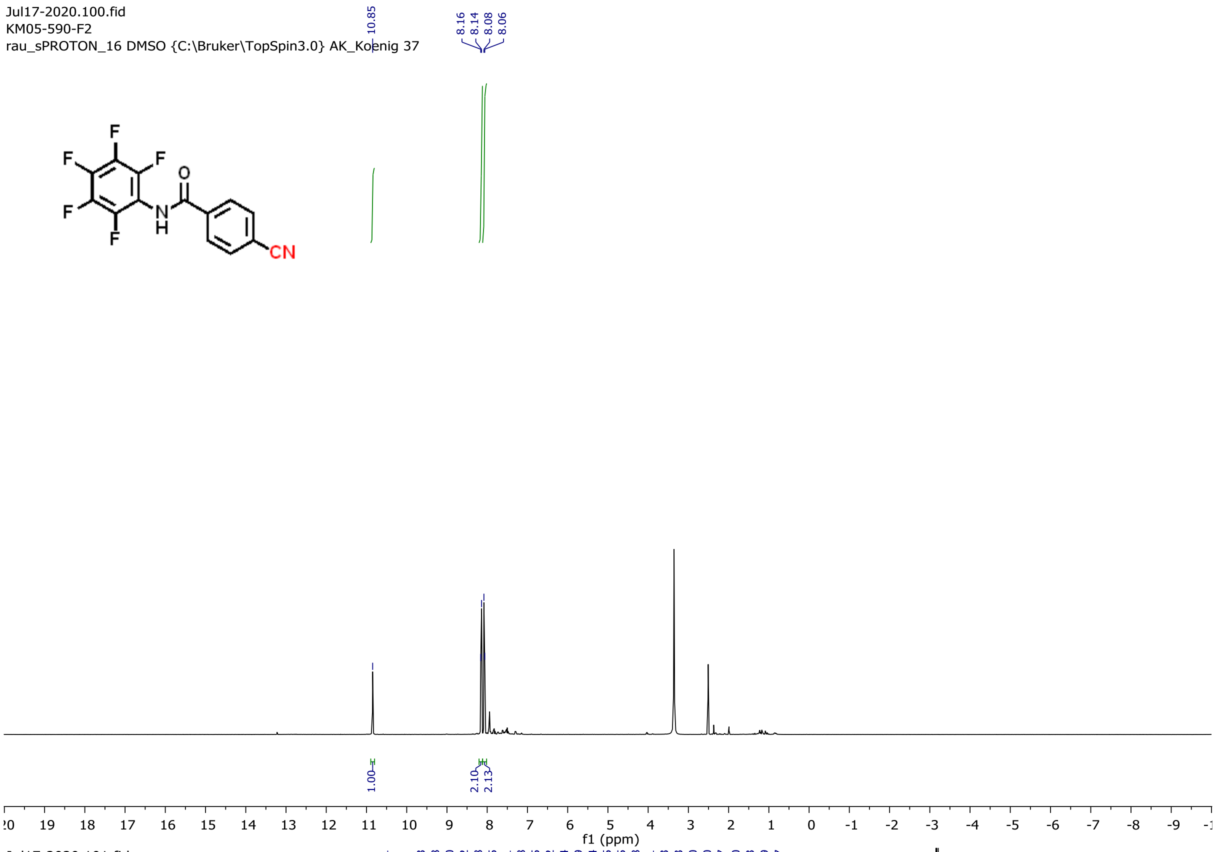
Jul17-2020.100.fid
KM05-590-F2
rau_sPROTON_16 DMSO {C:\Bruker\TopSpin3.0} AK_Koenig 37
10.85
8.16
8.14
8.08
8.06
1.00
2.10
2.13
f1 (ppm)

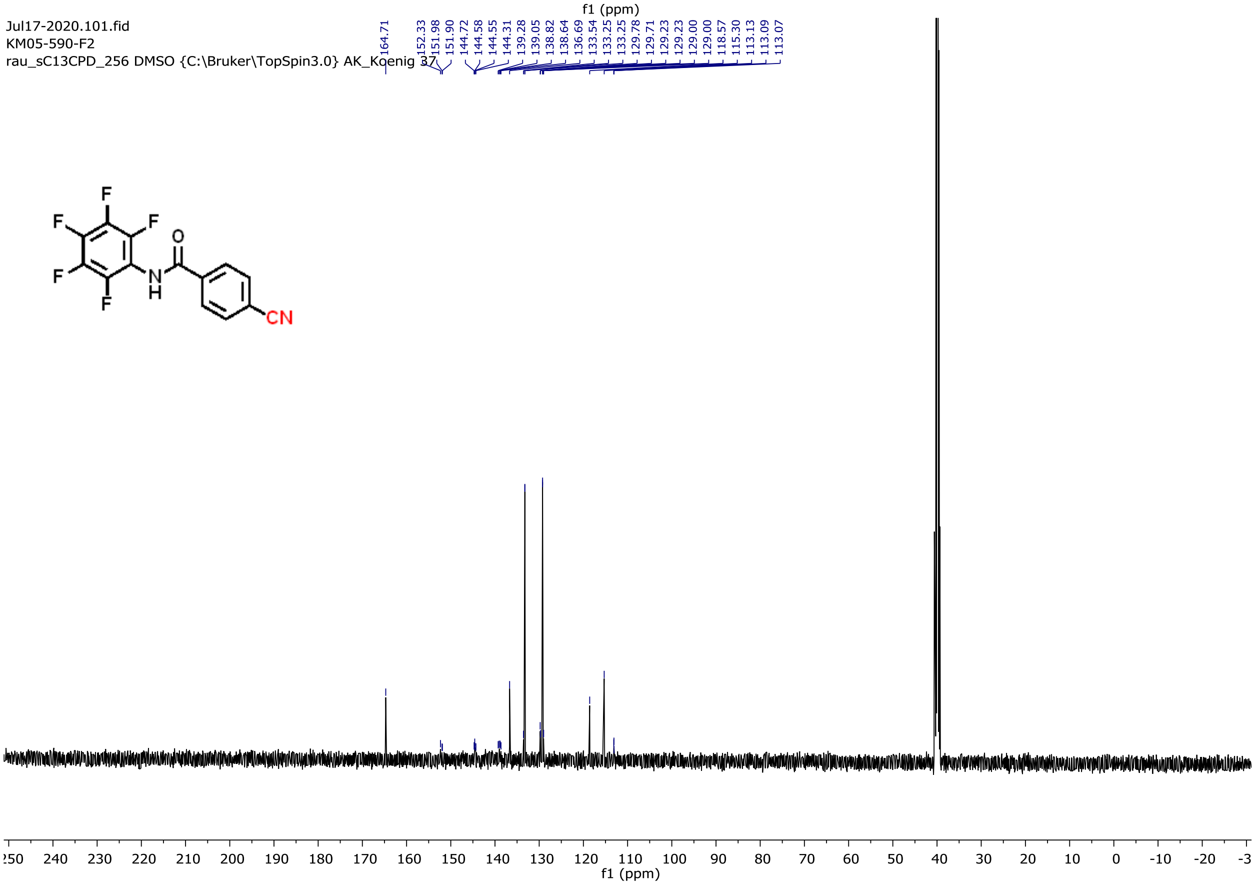
Jul17-2020.101.fid
KM05-590-F2
rau_sC13CPD_256 DMSO {C:\Bruker\TopSpin3.0} AK_Koenig 37
164.71
152.33
151.98
151.90
144.72
144.58
144.55
144.31
139.28
139.05
138.82
138.64
136.69
133.54
133.25
133.25
129.78
129.71
129.23
129.23
129.00
129.00
118.57
115.30
113.13
113.09
113.07
f1 (ppm)

Jul17-2020.102.fid
KM05-590-F2
rau_sF19CPD DMSO {C:\Bruker\TopSpin3.0} AK_Koenig 37

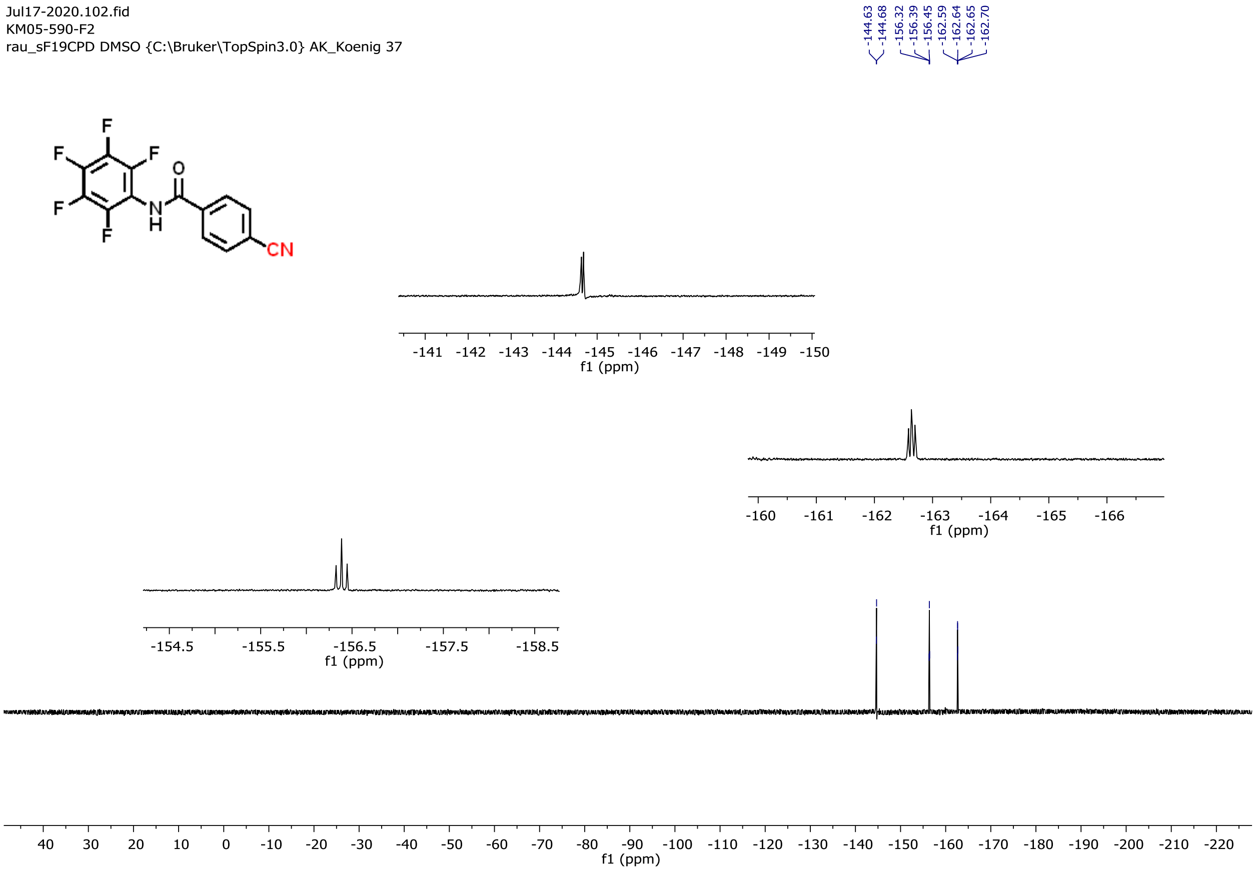

Jul28-2020.40.fid
KM05-501
rau_sPROTON_16 CDCl3 {C:\Bruker\TopSpin3.0} AK_Koenig 19

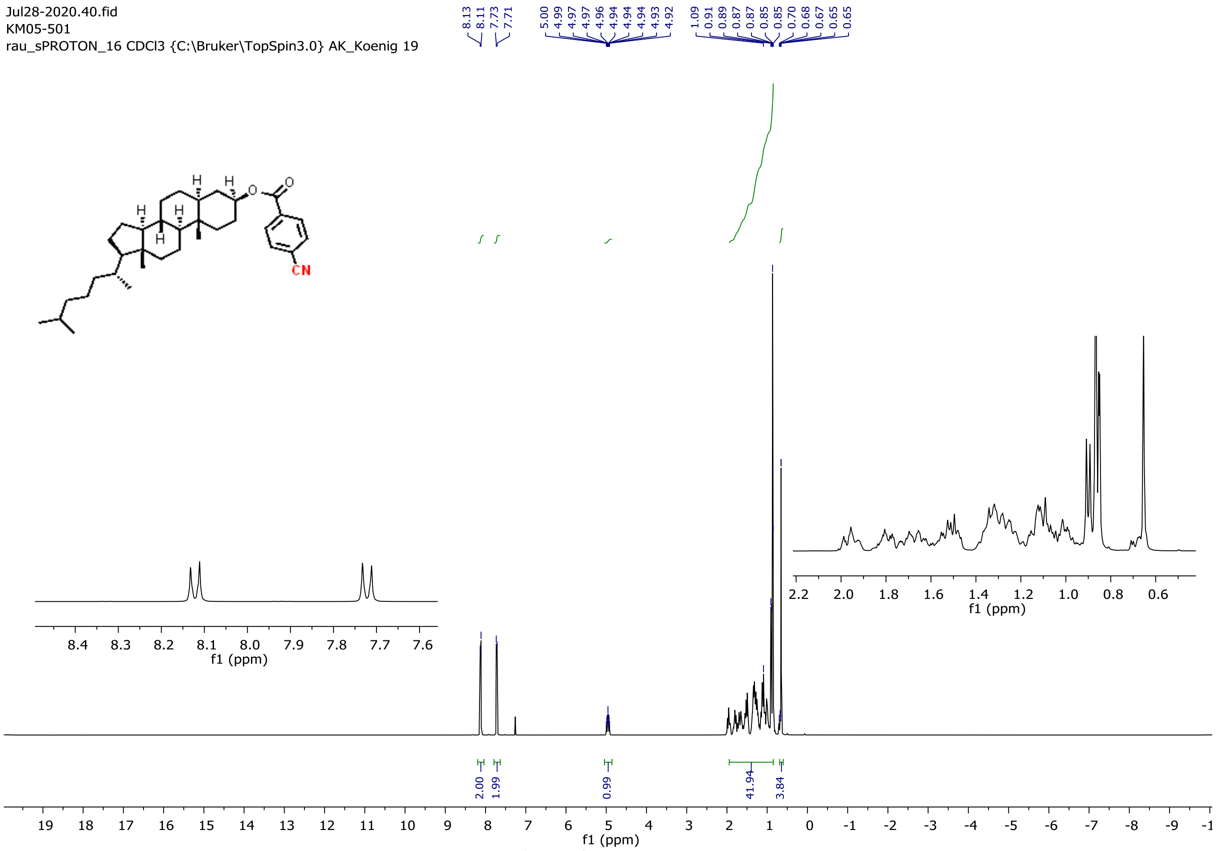

Jul28-2020.41.fid
KM05-501
rau_sC13CPD_256 CDCl3 {C:\Bruker\TopSpin3.0} AK_Koenig 19

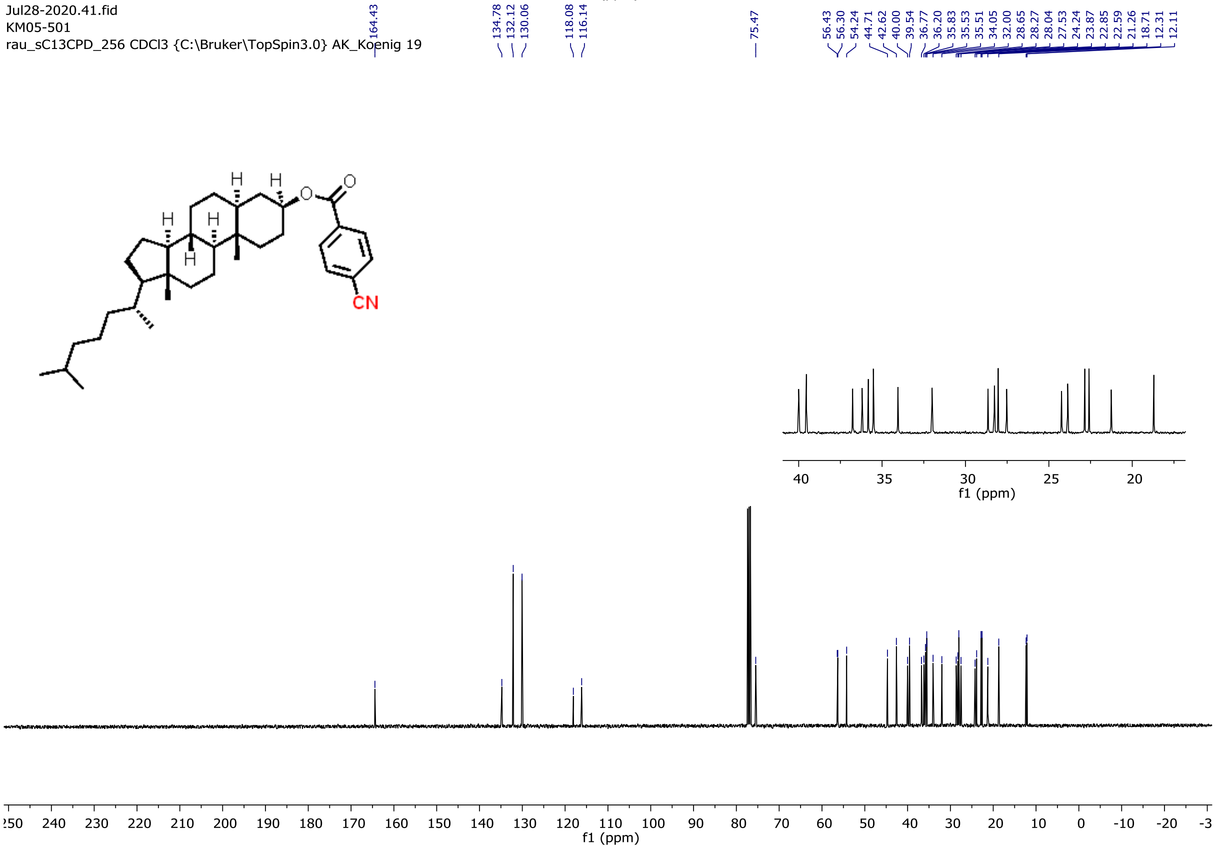

Jun30-2020.100.fid
KM05-513
rau_PROTONLF_16 CDCl3 {C:\Bruker\TOPSPIN2.1PL3} AK_Koenig 56

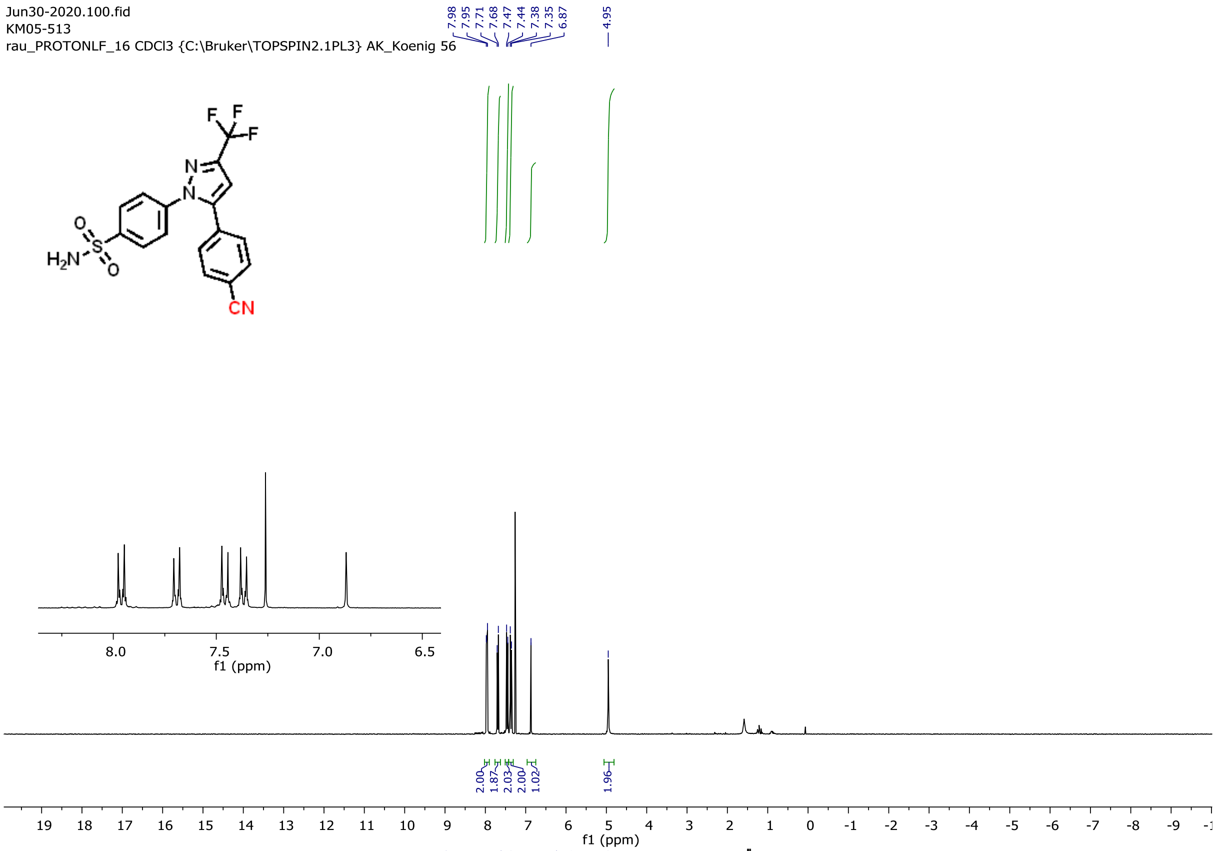

Jul28-2020.10.fid
KM05-513-CDCl3
rau_sC13CPD_1k CDCl3 {C:\Bruker\TopSpin3.0} AK_Koenig 7

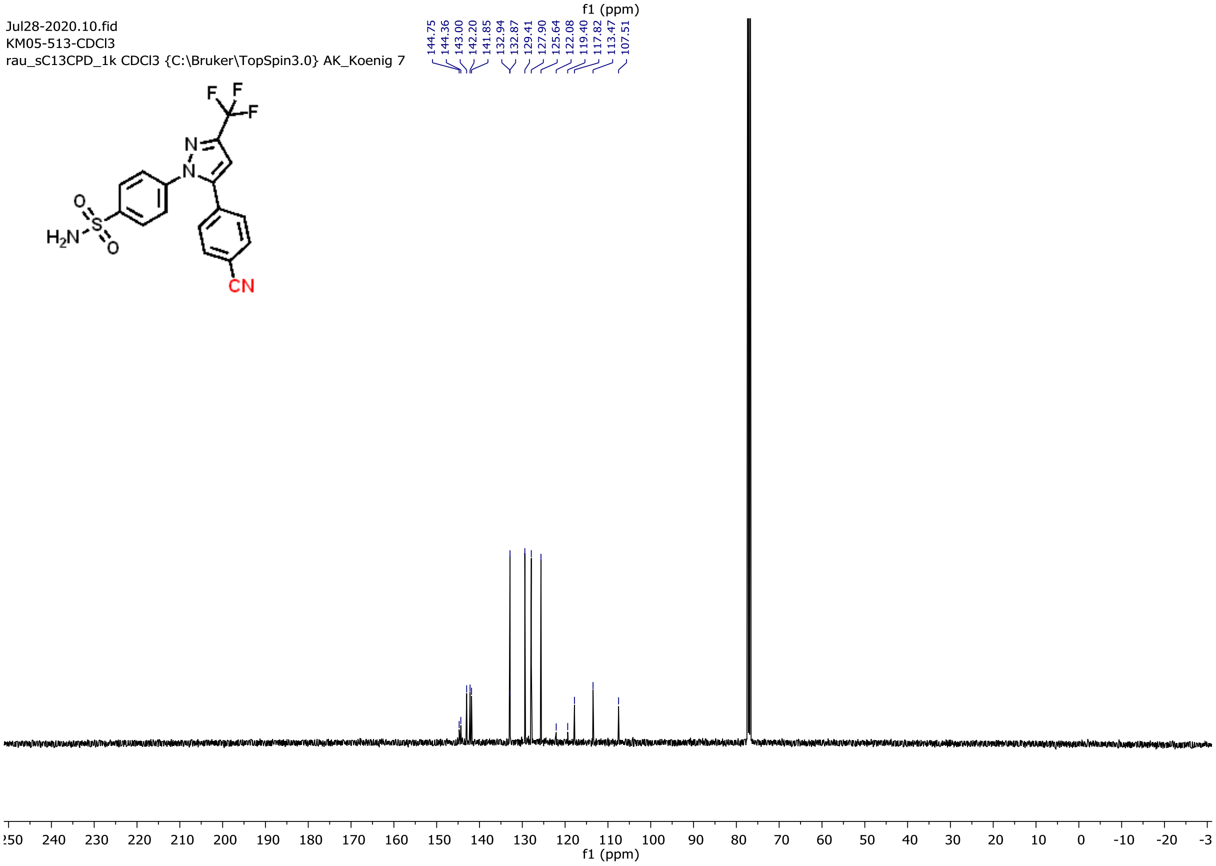

Jul01-2020.32.fid
KM05-513
rau_sF19CPD CDCl3 {C:\Bruker\TopSpin3.0} AK_Koenig 18

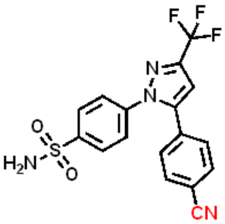

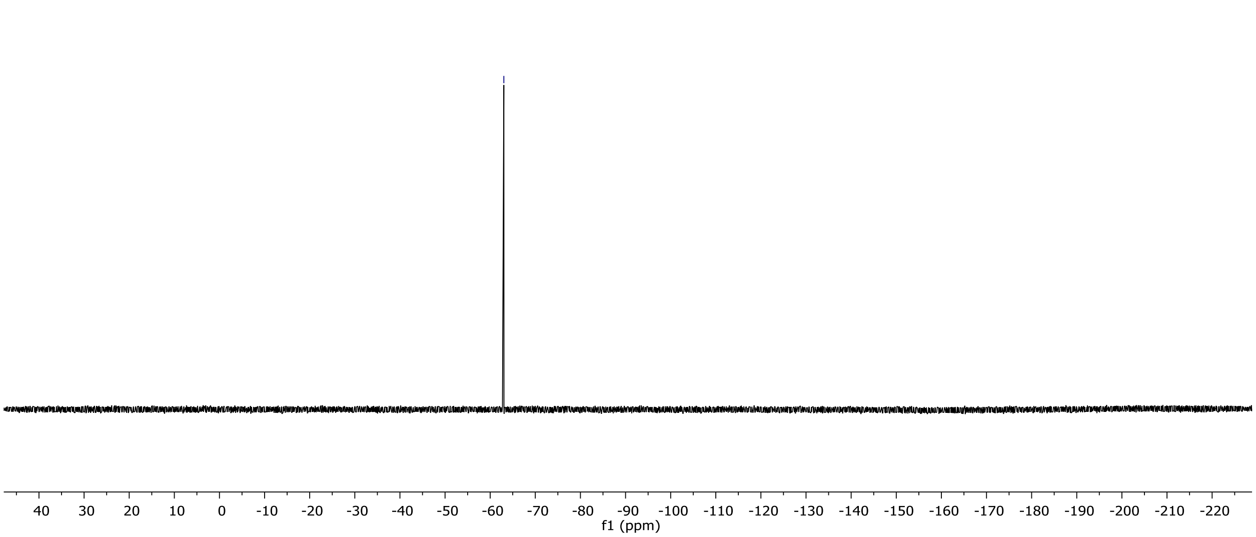

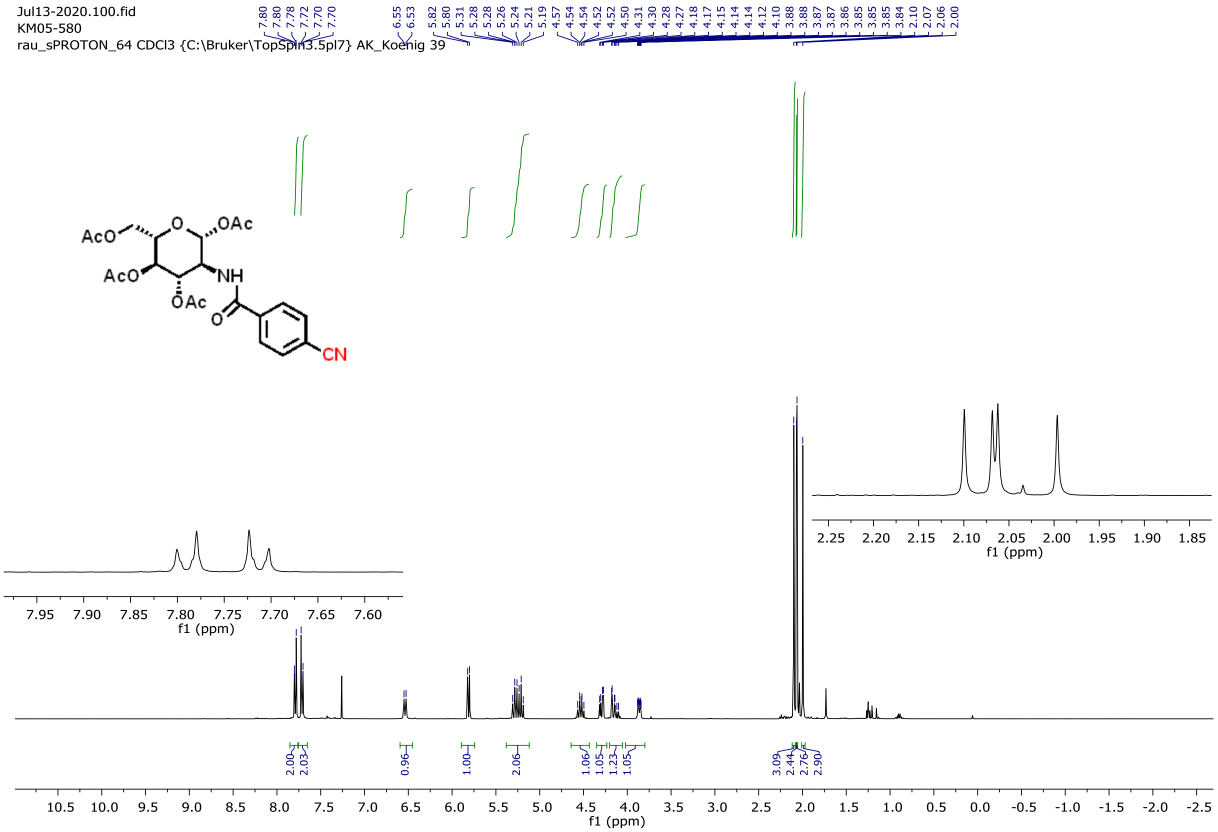
Jul13-2020.100.fid
KM05-580
rau_sPROTON_64 CDCl3 {C:\Bruker\TopSpin3.5pl7} AK_Koenig 39
7.80 7.80 7.78 7.72 7.70 7.70 6.55 6.53 5.82 5.80 5.31 5.28 5.28 5.26 5.24 5.21 5.19 4.57 4.54 4.54 4.52 4.52 4.50 4.31 4.30 4.28 4.27 4.18 4.17 4.15 4.14 4.14 4.12 4.10 3.88 3.88 3.87 3.87 3.86 3.85 3.85 3.85 3.84 2.10 2.07 2.06 2.00
AcO
OAc
AcO
NH
OAc
O
CN
2.00 2.03 0.96 1.00 2.06 1.06 1.05 1.23 1.05 3.09 2.44 2.76 2.90
f1 (ppm)

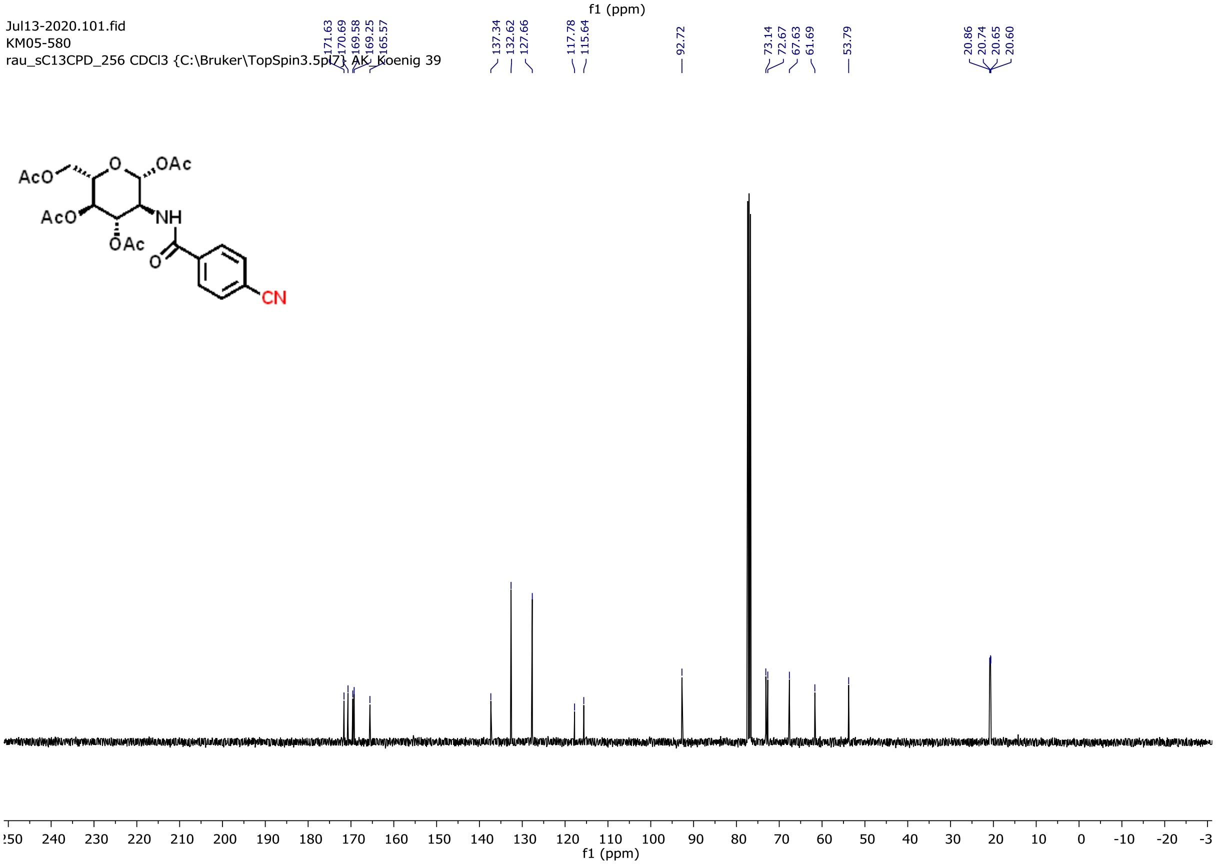
Jul13-2020.101.fid
KM05-580
rau_sC13CPD_256 CDCl3 {C:\Bruker\TopSpin3.5pl7} AK_Koenig 39
171.63 170.69 169.58 169.25 165.57 137.34 132.62 127.66 117.78 115.64 92.72 73.14 72.67 67.63 61.69 53.79 20.86 20.74 20.65 20.60
AcO
OAc
AcO
NH
OAc
O
CN
f1 (ppm)

Jul03-2020.60.fid
KM05-540
rau_sPROTON_64 CDCl3 {C:\Bruker\TopSpin3.5pl7} AK_Koenig 50

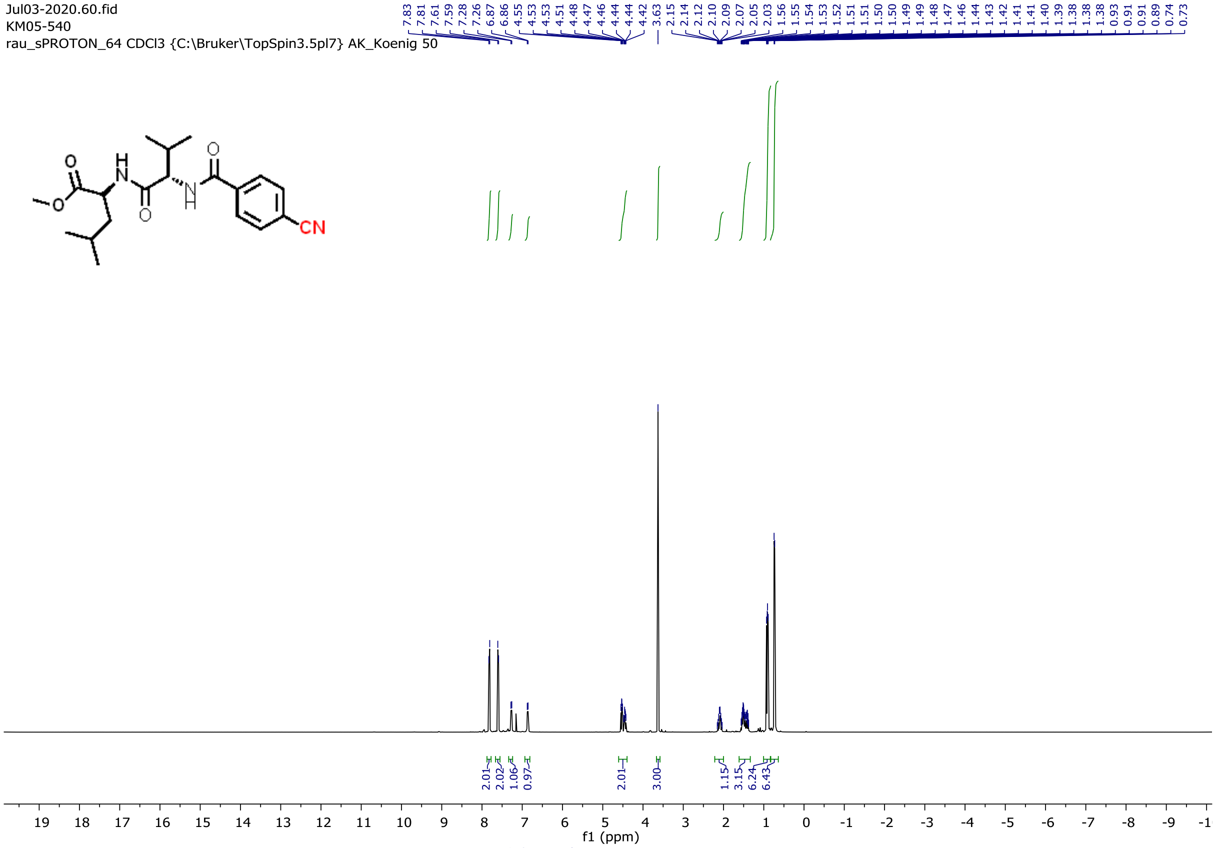

Jul03-2020.61.fid
KM05-540
rau_sC13CPD_256 CDCl3 {C:\Bruker\TopSpin3.5pl7} AK_Koenig 50

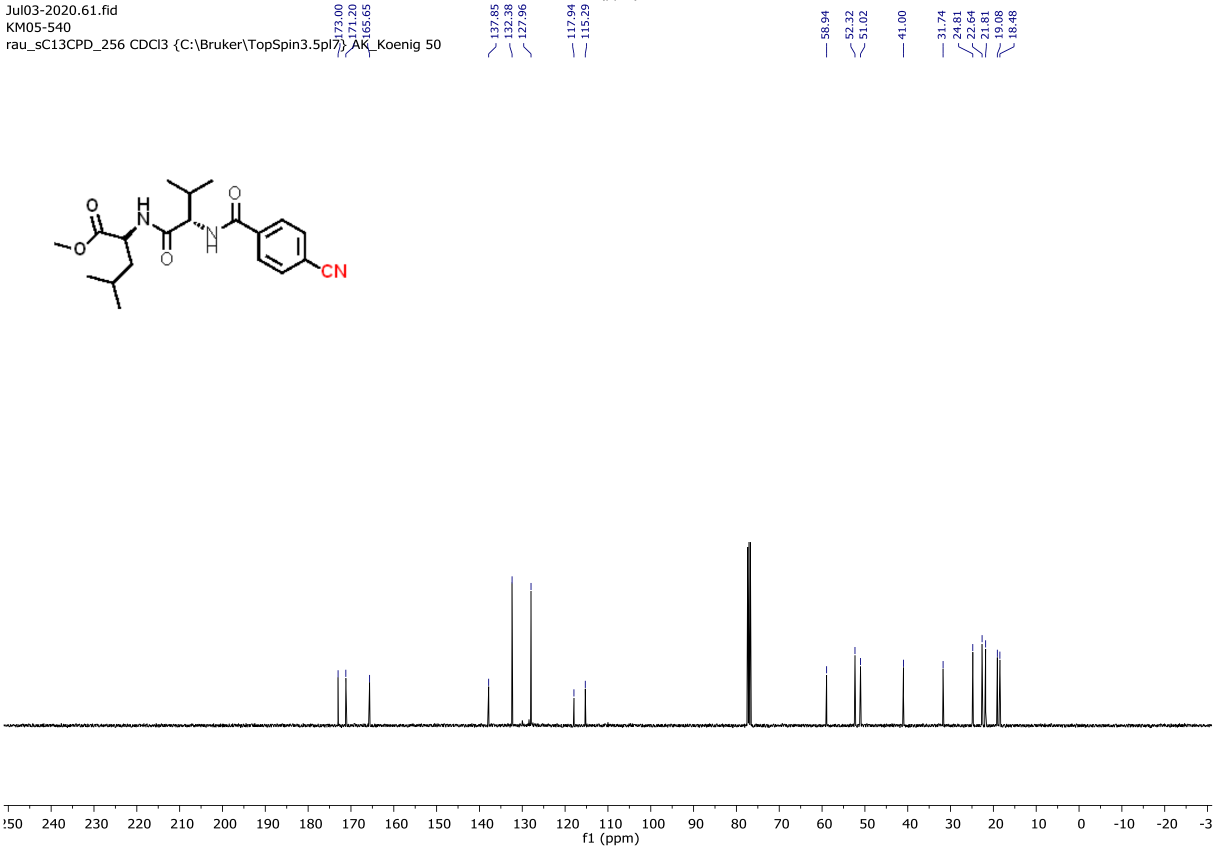

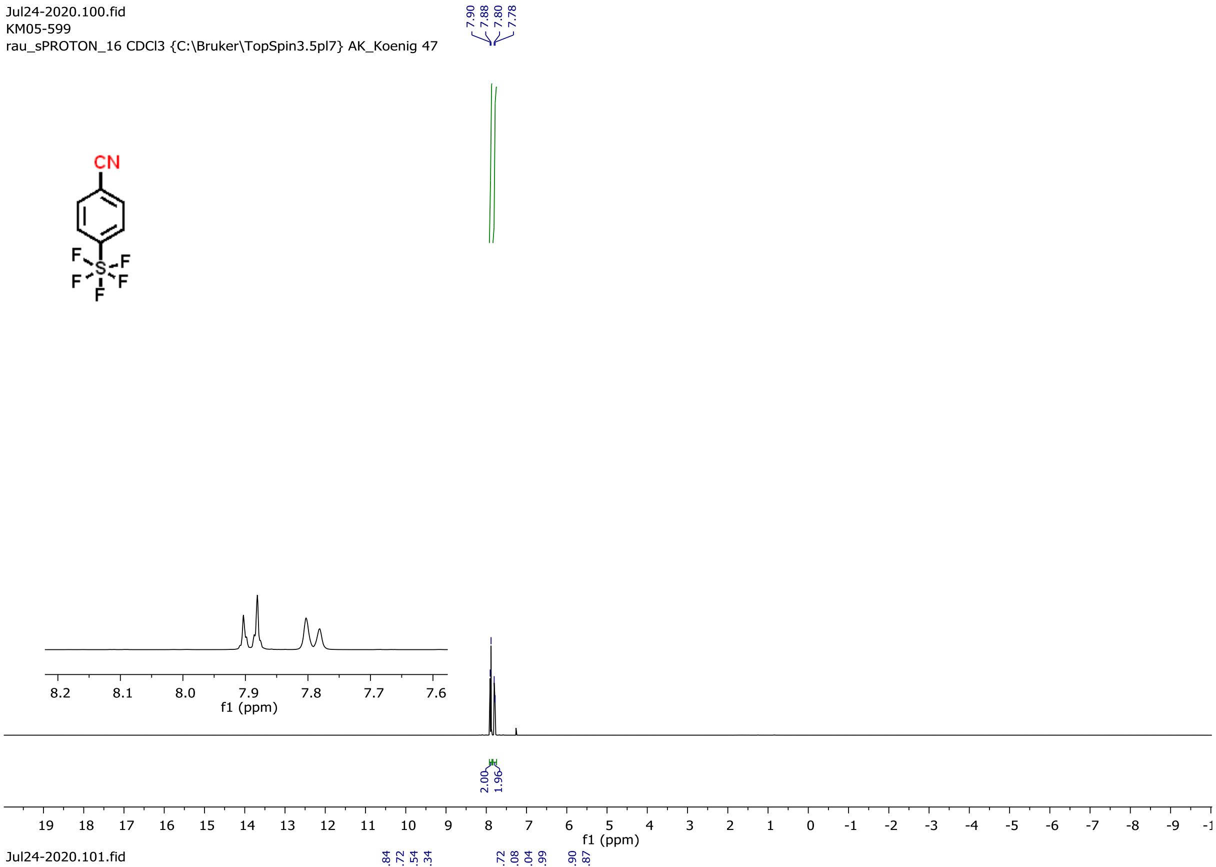
Jul24-2020.100.fid
KM05-599
rau_sPROTON_16 CDCl3 {C:\Bruker\TopSpin3.5pl7} AK_Koenig 47
7.90
7.88
7.80
7.78
CN
F
S
F
F
F
F
2.00
1.96
8.2 8.1 8.0 7.9 7.8 7.7 7.6
f1 (ppm)
19 18 17 16 15 14 13 12 11 10 9 8 7 6 5 4 3 2 1 0 -1 -2 -3 -4 -5 -6 -7 -8 -9
f1 (ppm)

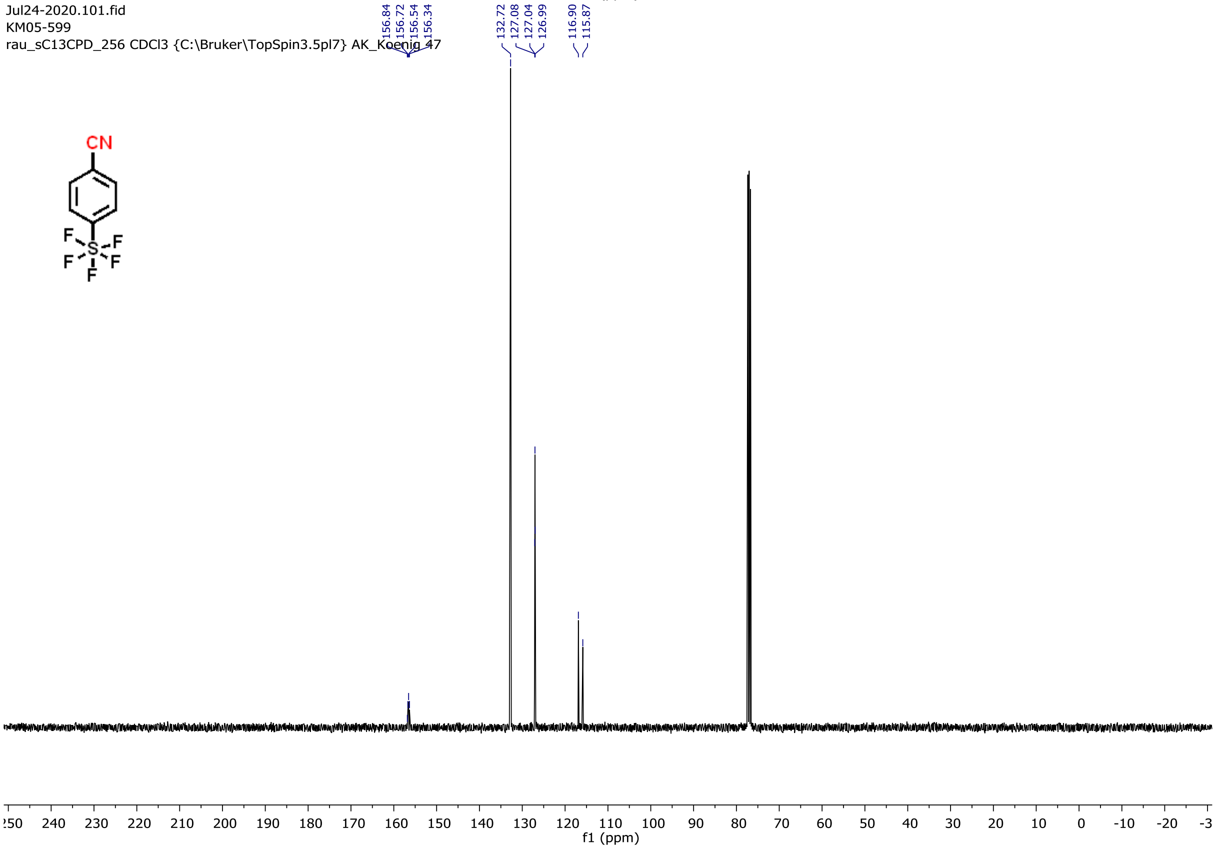
Jul24-2020.101.fid
KM05-599
rau_sC13CPD_256 CDCl3 {C:\Bruker\TopSpin3.5pl7} AK_Koenig 47
156.84
156.72
156.54
156.34
132.72
127.08
127.04
126.99
116.90
115.87
CN
F
S
F
F
F
F
240 230 220 210 200 190 180 170 160 150 140 130 120 110 100 90 80 70 60 50 40 30 20 10 0 -10 -20
f1 (ppm)

Jul23-2020.70.fid
KM05-597
rau_sPROTON_16 CDCl3 {C:\Bruker\TopSpin3.5pl7} AK_Koenig 41

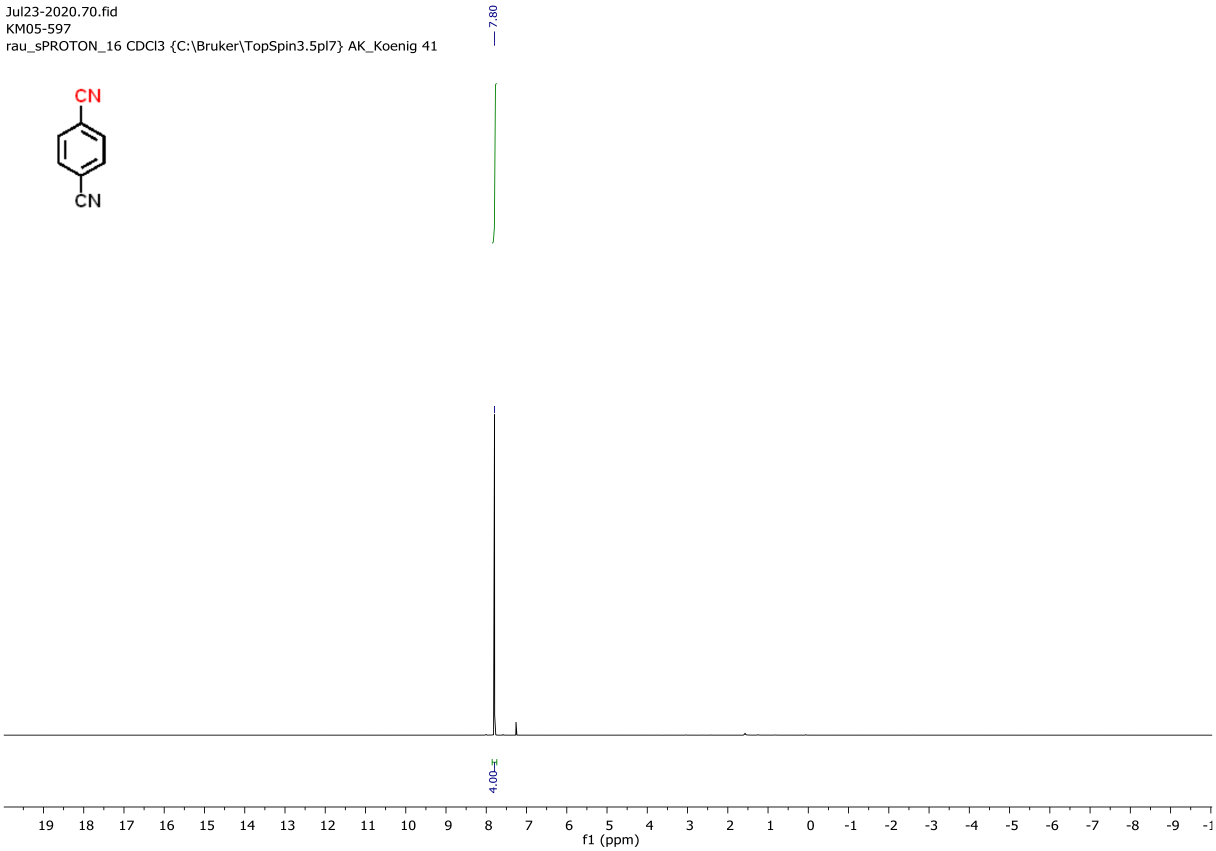

Jul23-2020.71.fid
KM05-597
rau_sC13CPD_256 CDCl3 {C:\Bruker\TopSpin3.5pl7} AK_Koenig 41

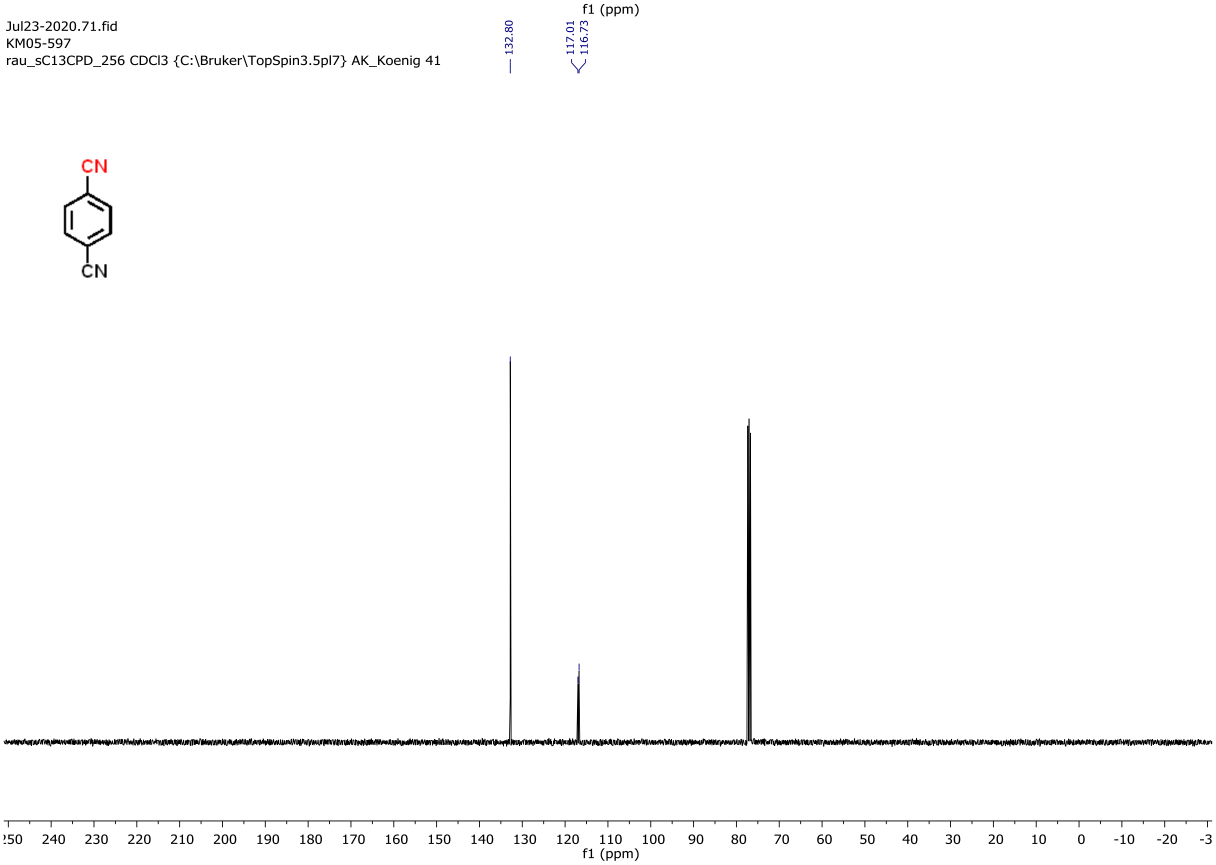

Jul21-2020.120.fid
KM05-612
rau_sPROTON_16 CDCl3 {C:\Bruker\TopSpin3.5pl7} AK_Koenig 28

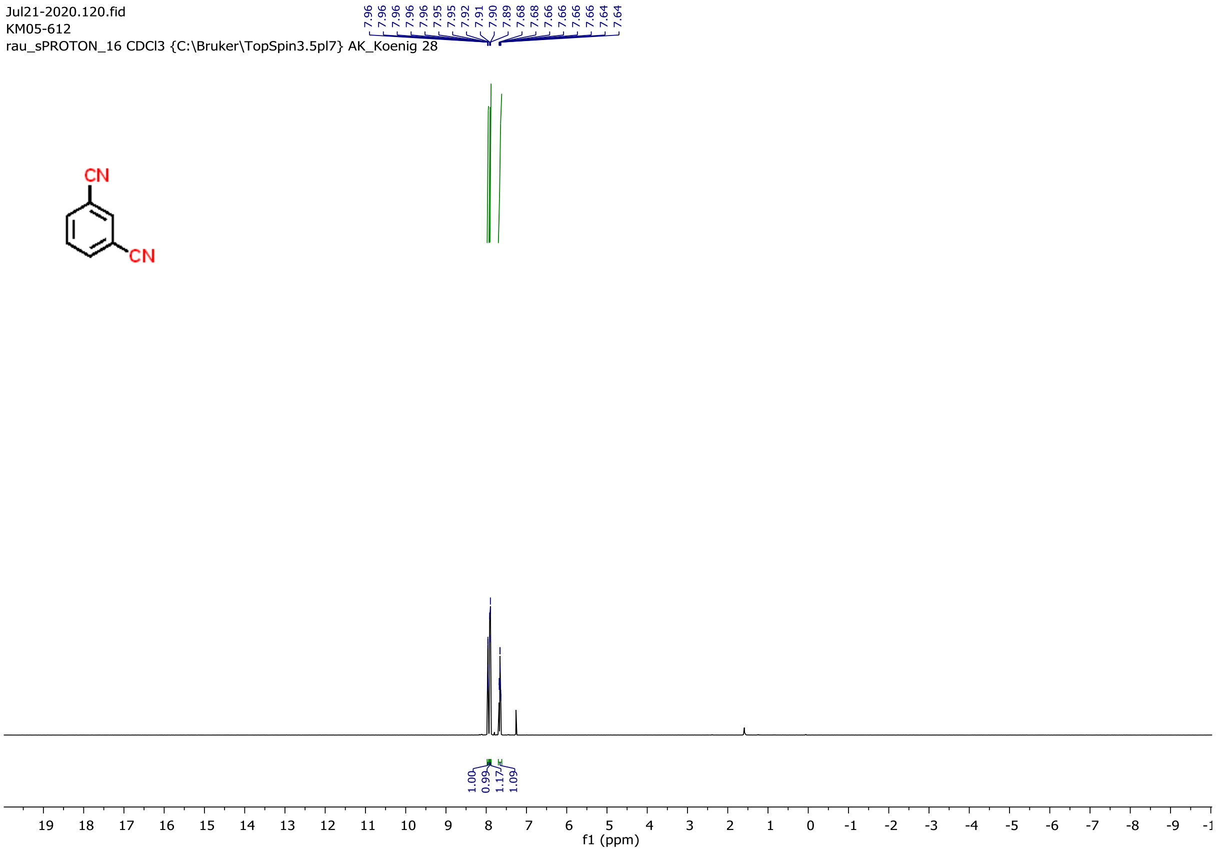

Jul21-2020.121.fid
KM05-612
rau_sC13CPD_256 CDCl3 {C:\Bruker\TopSpin3.5pl7} AK_Koenig 28

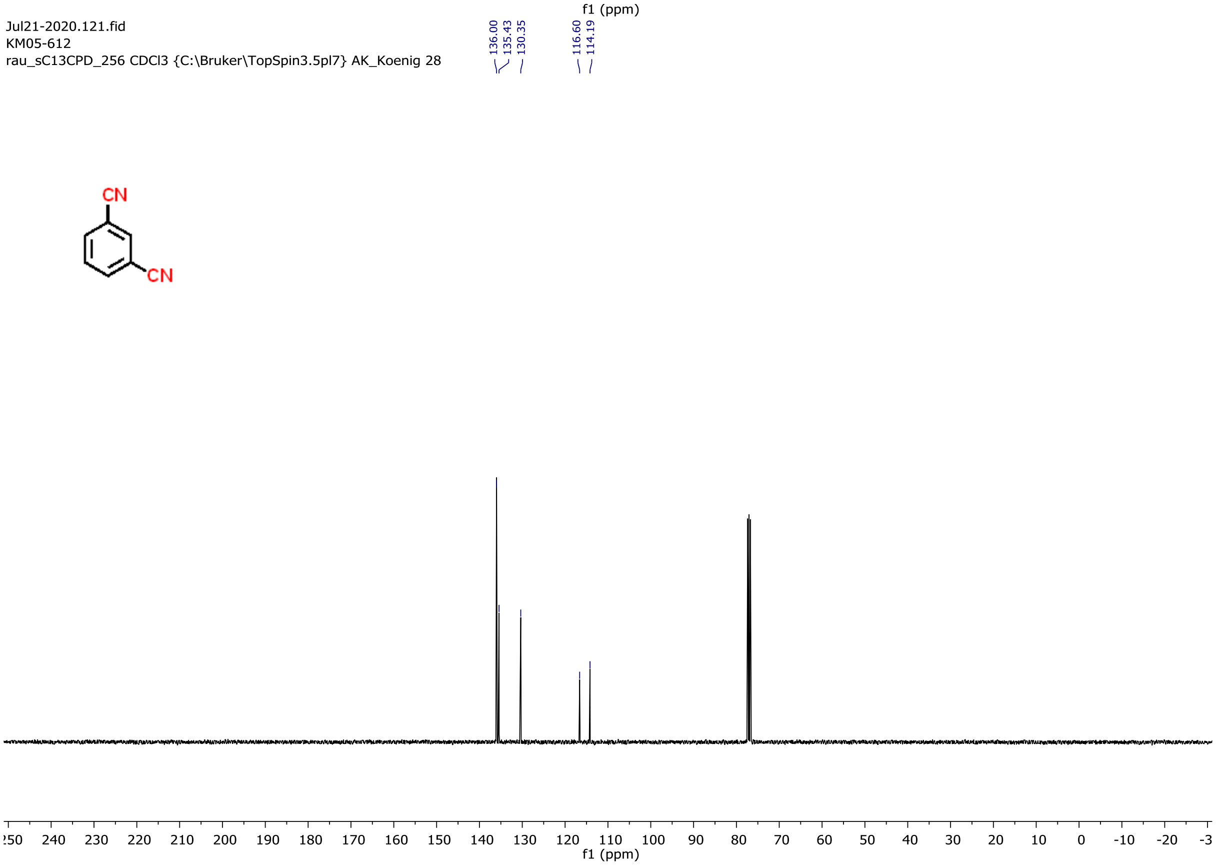

Jul21-2020.100.fid
KM05-609
rau_sPROTON_16 CDCl3 {C:\Bruker\TopSpin3.5pl7} AK_Koenig 26

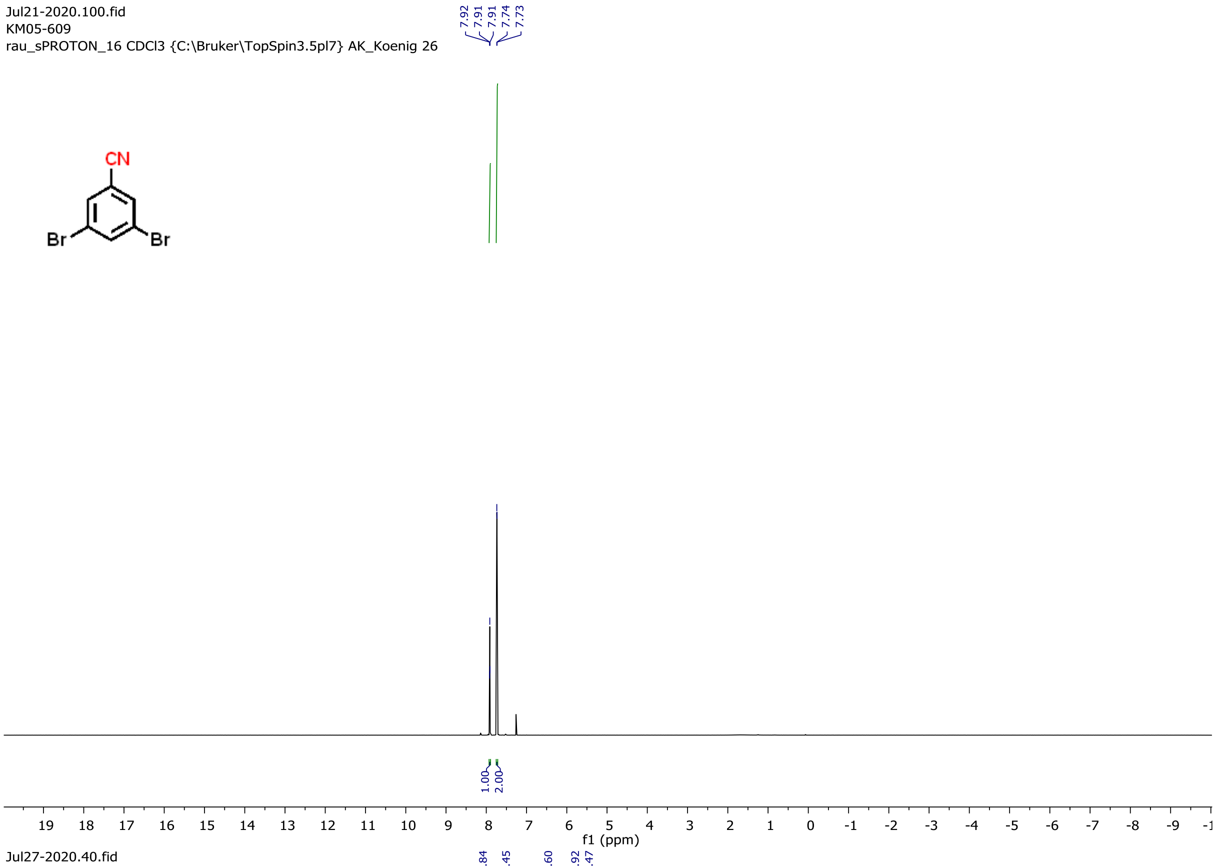

Jul27-2020.40.fid
KM05-609
rau_sC13CPD_256 CDCl3 {C:\Bruker\TopSpin3.5pl7} AK_Koenig 15

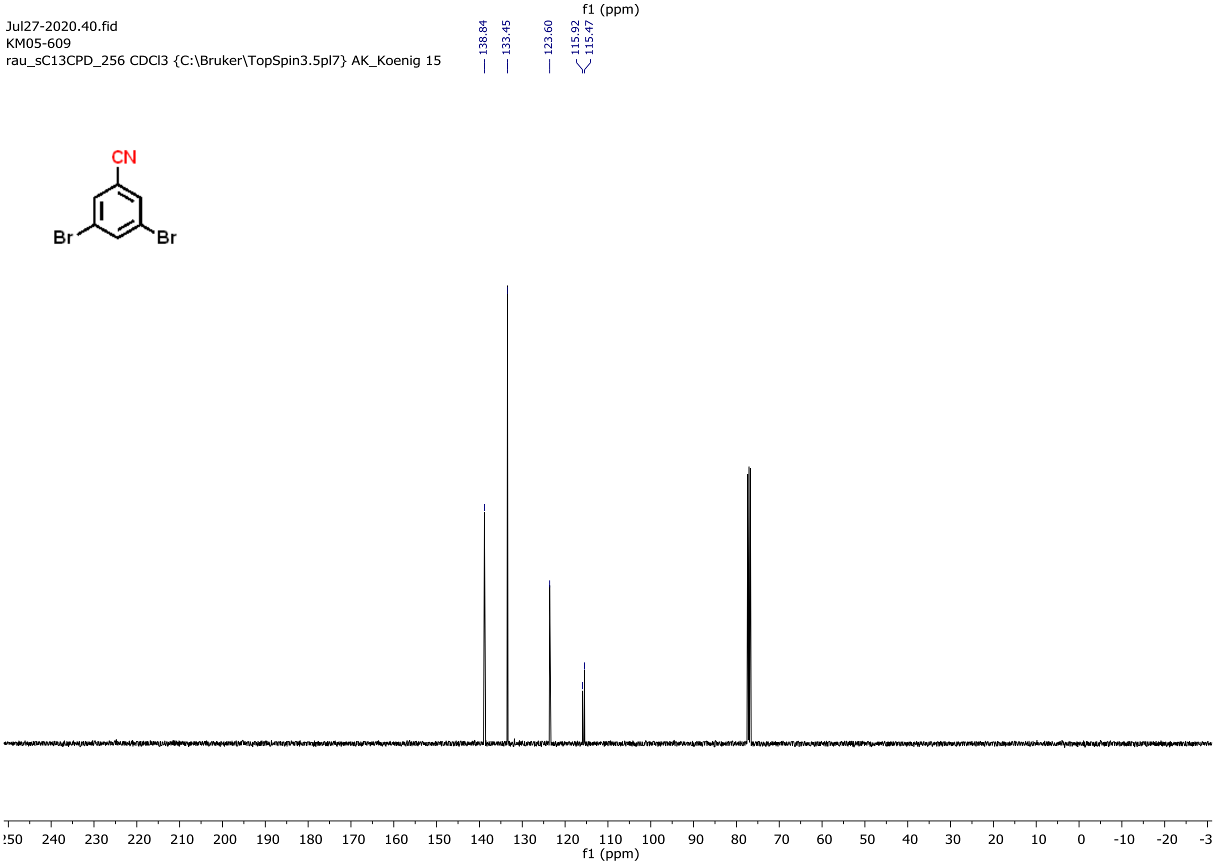

Jul21-2020.110.fid
KM05-610
rau_sPROTON_16 CDCl3 {C:\Bruker\TopSpin3.5pl7} AK_Koenig 27

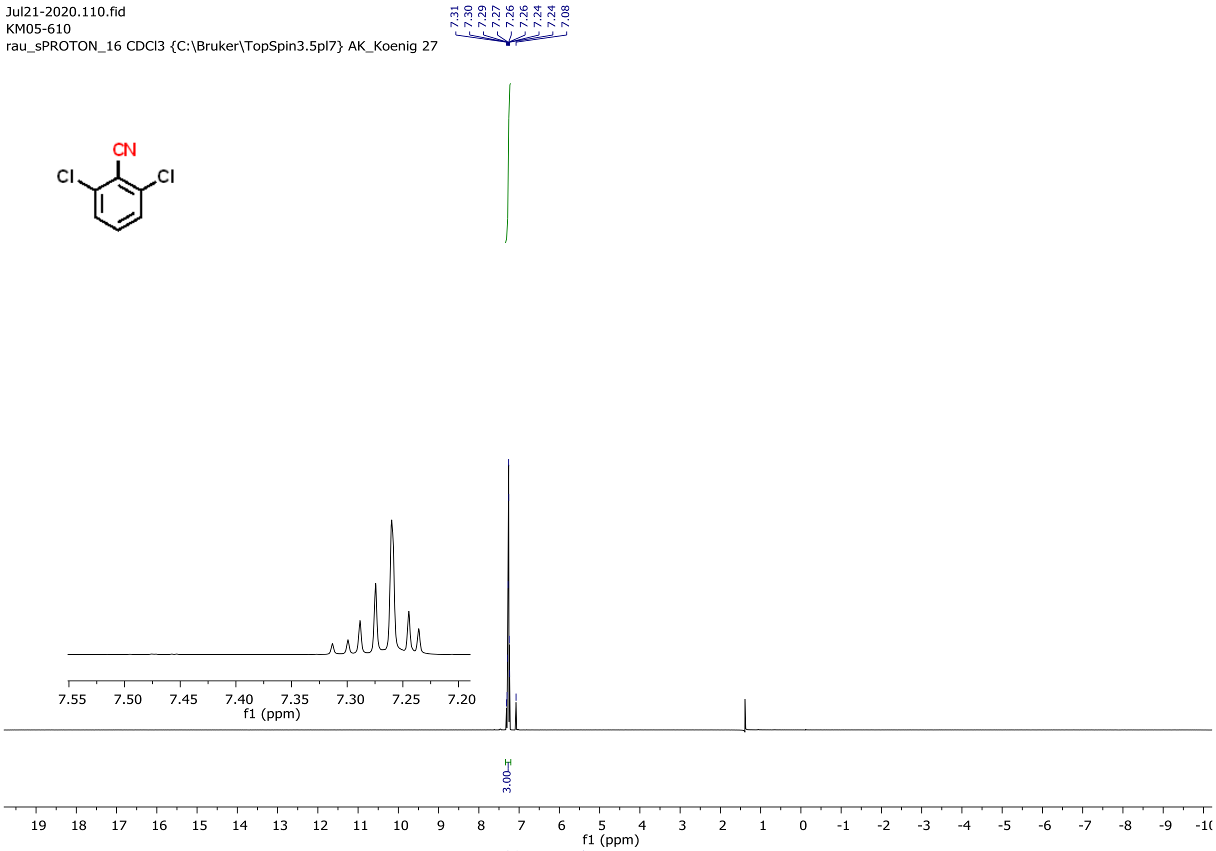

Jul27-2020.50.fid
KM05-610
rau_sC13CPD_256 CDCl3 {C:\Bruker\TopSpin3.5pl7} AK_Koenig 16

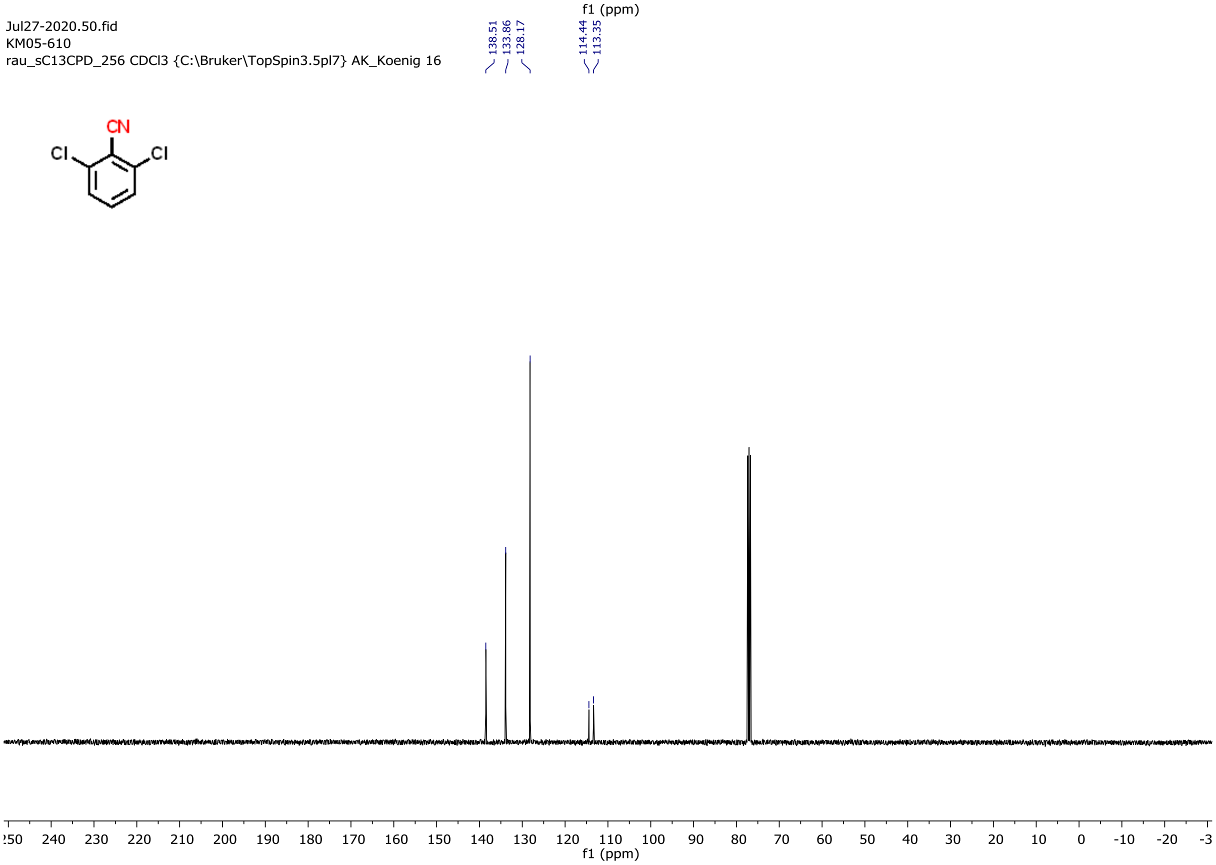

Jul23-2020.80.fid
KM05-604
rau_sPROTON_16 CDCl3 {C:\Bruker\TopSpin3.5pl7} AK_Koenig 42

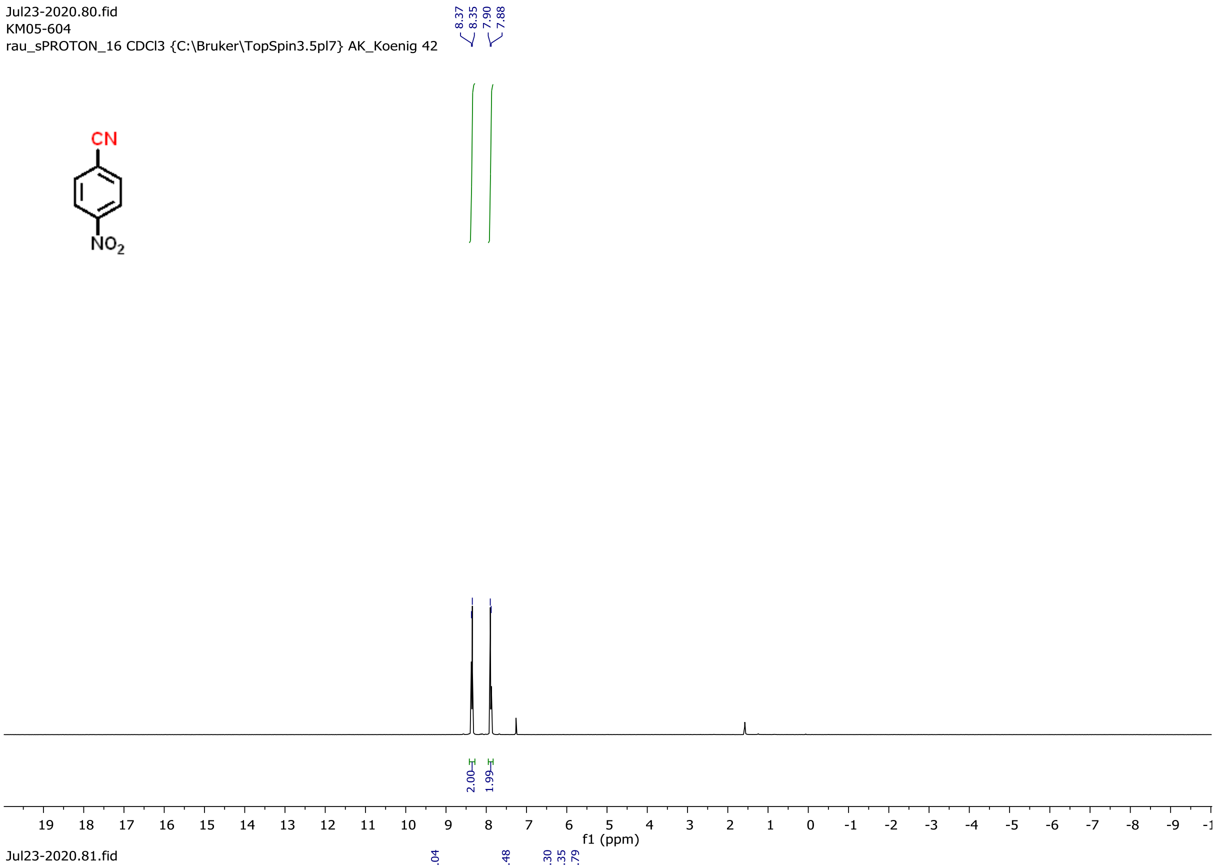

Jul23-2020.81.fid
KM05-604
rau_sC13CPD_256 CDCl3 {C:\Bruker\TopSpin3.5pl7} AK_Koenig 42

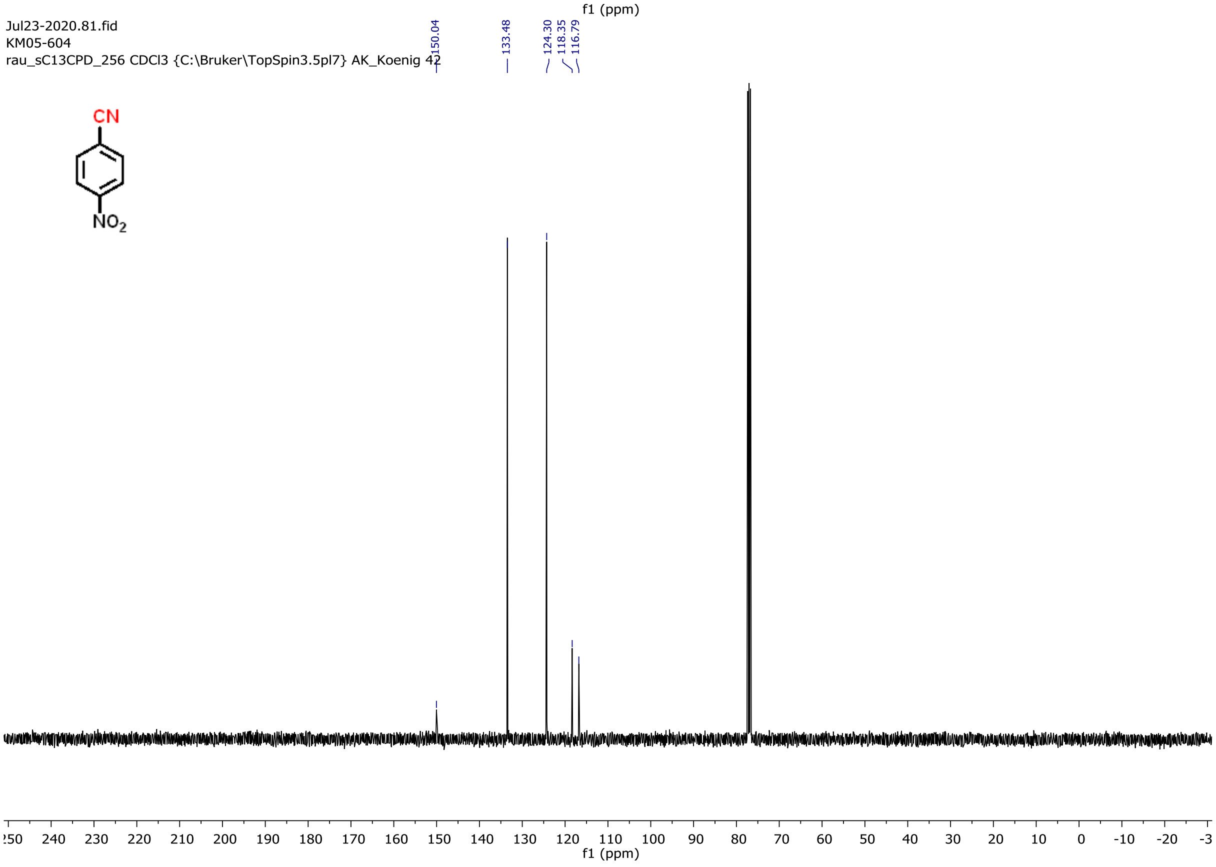

7. References

[1] H. Robin K., B. Edwin D., C. d. M. Sonia M., G. Robin, G. Pierre, *Magn. Reson. Chem.* **2002**, *40*, 622-622.

[2] G. R. Fulmer, A. J. M. Miller, N. H. Sherden, H. E. Gottlieb, A. Nudelman, B. M. Stoltz, J. E. Bercaw, K. I. Goldberg, *Organometallics* **2010**, *29*, 2176-2179.

[3] Y. Shen, Y. Gu, R. Martin, *J. Am. Chem. Soc.* **2018**, *140*, 12200-12209.

[4] D. Simoni, R. Rondanin, R. Baruchello, M. Rizzi, G. Grisolia, M. Eleopra, S. Grimaudo, A. D. Cristina, M. R. Pipitone, M. R. Bongiorno, M. Aricò, F. P. Invidiata, M. Tolomeo, *J. Med. Chem.* **2008**, *51*, 4796-4803.

[5] N. A. Romero, K. A. Margrey, N. E. Tay, D. A. Nicewicz, *Science* **2015**, *349*, 1326-1330.

[6] V. V. Pavlishchuk, A. W. Addison, *Inorg. Chim. Acta* **2000**, *298*, 97-102.

[7] J. M. Ovian, C. B. Kelly, V. A. Pistritto, N. E. Leadbeater, *Org. Lett.* **2017**, *19*, 1286-1289.

[8] P.-F. Dai, X.-S. Ning, H. Wang, X.-C. Cui, J. Liu, J.-P. Qu, Y.-B. Kang, *Angew. Chem. Int. Ed.* **2019**, *58*, 5392-5395.

[9] C. C. D. Wybon, C. Mensch, K. Hollanders, C. Gadais, W. A. Herrebout, S. Ballet, B. U. W. Maes, *ACS Catal.* **2018**, *8*, 203-218.

[10] G. Tu, Y. Yan, X. Chen, Q. Lv, J. Wang, S. Li, *Drug discoveries & therapeutics* **2013**, *7*, 58-65.

[11] K. Lam, I. E. Markó, *Org. Lett.* **2008**, *10*, 2773-2776.

[12] H. Kong, W. Chen, H. Lu, Q. Yang, Y. Dong, D. Wang, J. Zhang, *Carbohydr. Res.* **2015**, *413*, 135-144.

[13] A. Kasprzak, M. Bystrzejewski, M. Koszytkowska-Stawinska, M. Poplawska, *Green Chem.* **2017**, *19*, 3510-3514.

[14] Q. V. Vo, C. Trenerry, S. Rochfort, J. Wadeson, C. Leyton, A. B. Hughes, *Biorg. Med. Chem.* **2013**, *21*, 5945-5954.

[15] L. Schwarz, U. Girreser, B. Clement, *Eur. J. Org. Chem* **2014**, *2014*, 1961-1975.

[16] J. Li, Y. Okuda, J. Zhao, S. Mori, Y. Nishihara, *Org. Lett.* **2014**, *16*, 5220-5223.

[17] A. Sridhar, S. Swarnalakshmi, M. Selvaraj, *Synlett* **2016**, *27*, 1344-1348.

[18] W.-Y. Fang, H.-L. Qin, *J. Org. Chem.* **2019**, *84*, 5803-5812.

[19] Y.-Y. Liu, D. Liang, L.-Q. Lu, W.-J. Xiao, *Chem. Commun.* **2019**, *55*, 4853-4856.

[20] C. Ma, C.-Q. Zhao, X.-T. Xu, Z.-M. Li, X.-Y. Wang, K. Zhang, T.-S. Mei, *Org. Lett.* **2019**, *21*, 2464-2467.

[21] M. Liu, C.-J. Li, *Angew. Chem. Int. Ed.* **2016**, *55*, 10806-10810.

[22] R. S. Mane, B. M. Bhanage, *RSC Adv.* **2015**, *5*, 76122-76127.

[23] L. R. Mills, J. M. Graham, P. Patel, S. A. L. Rousseaux, *J. Am. Chem. Soc.* **2019**, *141*, 19257-19262.

[24] L. Wang, J. Li, X. Cui, Y. Wu, Z. Zhu, Y. Wu, *Adv. Synth. Catal.* **2010**, *352*, 2002-2010.

[25] J. Liu, H.-X. Zheng, C.-Z. Yao, B.-F. Sun, Y.-B. Kang, *J. Am. Chem. Soc.* **2016**, *138*, 3294-3297.

[26] Z. Shu, Y. Ye, Y. Deng, Y. Zhang, J. Wang, *Angew. Chem., Int. Ed.* **2013**, *52*, 10573-10576.

[27] N. Varga, I. Sutkeviciute, C. Guzzi, J. McGeagh, I. Petit-Haertlein, S. Gugliotta, J. Weiser, J. Angulo, F. Fieschi, A. Bernardi, *Chem. Eur. J.* **2013**, *19*, 4786-4797.

[28] A. Nagaki, K. Hirose, Y. Moriwaki, K. Mitamura, K. Matsukawa, N. Ishizuka, J. Yoshida, *Catal. Sci. Technol.* **2016**, *6*, 4690-4694.

[29] J. Ahmed, S. Chakraborty, A. Jose, S. P, S. K. Mandal, *J. Am. Chem. Soc.* **2018**, *140*, 8330-8339.

[30] T. Morioka, S. Nakatani, Y. Sakamoto, T. Kodama, S. Ogoshi, N. Chatani, M. Tobisu, *Chem. Sci.,* **2019**, *10*, 6666-6671.

[31] P. Krasik, *Tetrahedron Lett.* **1998**, *39*, 4223-4226.

[32] M. Shigeno, K. Hayashi, K. Nozawa-Kumada, Y. Kondo, *Chem. Eur. J.* **2019**, *25*, 6077-6081.

[33] L.-M. Jin, H. Lu, Y. Cui, C. L. Lizardi, T. N. Arzua, L. Wojtas, X. Cui, X. P. Zhang, *Chem. Sci.,* **2014**, *5*, 2422-2427.
[34] K. Ellis-Sawyer, R. A. Bragg, N. Bushby, C. S. Elmore, M. J. Hickey, *J Label Compd Radiopharm.* **2017**, *60*, 213-220.
[35] T. Okazaki, K. K. Laali, S. D. Bunge, S. K. Adas, *Eur. J. Org. Chem.* **2014**, *2014*, 1630-1644.
[36] J. Gurjar, J. Bater, V. V. Fokin, *Chem. Eur. J.* **2019**, *25*, 1906-1909.
[37] K. Murugesan, T. Senthamarai, M. Sohail, M. Sharif, N. V. Kalevaru, R. V. Jagadeesh, *Green Chem.* **2018**, *20*, 266-273.
